# Fluoromethylketone‐Fragment Conjugates Designed as Covalent Modifiers of *Ec*DsbA are Atypical Substrates

**DOI:** 10.1002/cmdc.202300684

**Published:** 2024-07-08

**Authors:** Bradley C. Doak, Rebecca L. Whitehouse, Kieran Rimmer, Martin Williams, Begoña Heras, Sofia Caria, Olga Ilyichova, Mansha Vazirani, Biswaranjan Mohanty, Jason B. Harper, Martin J. Scanlon, Jamie S. Simpson

**Affiliations:** ^1^ Medicinal Chemistry ARC Centre for Fragment-Based Design Monash Institute of Pharmaceutical Sciences Monash University 381 Royal Parade Parkville VIC 3052 Australia; ^2^ Department of Biochemistry and Genetics La Trobe La Trobe University Kingsbury Drive Bundoora Vic 3083 Australia; ^3^ School of Chemistry University of New South Wales Sydney NSW 2052 Australia; ^4^ Sydney Analytical Core Research Facility The University of Sydney Sydney New South Wales 2006 Australia

**Keywords:** Enzymes, Inhibitors, NMR spectroscopy, Protein Structure

## Abstract

Disulfide bond protein A (DsbA) is an oxidoreductase enzyme that catalyzes the formation of disulfide bonds in Gram‐negative bacteria. In *Escherichia coli*, DsbA (*Ec*DsbA) is essential for bacterial virulence, thus inhibitors have the potential to act as antivirulence agents. A fragment‐based screen was conducted against *Ec*DsbA and herein we describe the development of a series of compounds based on a phenylthiophene hit identified from the screen. A novel thiol reactive and “clickable” ethynylfluoromethylketone was designed for reaction with azide‐functionalized fragments to enable rapid and versatile attachment to a range of fragments. The resulting fluoromethylketone conjugates showed selectivity for reaction with the active site thiol of *Ec*DsbA, however unexpectedly, turnover of the covalent adduct was observed. A mechanism for this turnover was investigated and proposed which may have wider ramifications for covalent reactions with dithiol‐disulfide oxidoreducatases.

## Graphical abstract

We report that the oxidoreductase enzyme DsbA can turnover thiol‐reactive functional groups such as fluoromethyl ketones and maleimide.

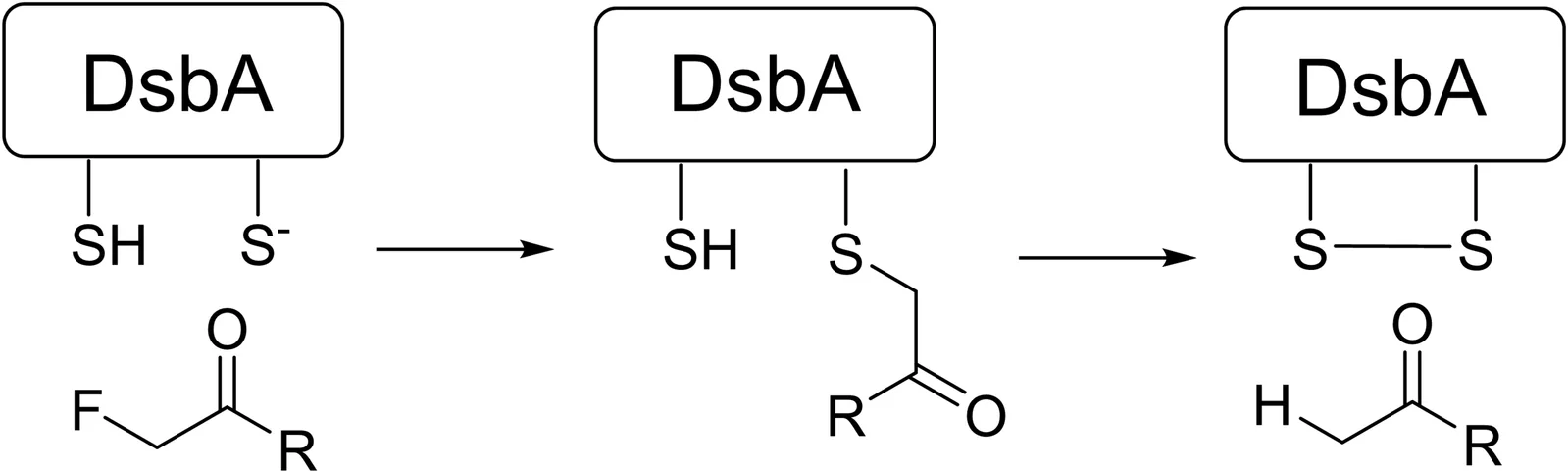

## Introduction

Disulfide bond protein A (DsbA) is a dithiol‐disulfide oxidoreductase enzyme that catalyzes the formation of disulfide bonds in bacterial proteins that enter the periplasm, including toxins and other virulence factors.[^1^, ^2^] Bacterial strains in which *dsbA* has been knocked out are avirulent and hence DsbA is a novel antivirulence target.[^2^, ^3^, ^4^, ^5^, ^6^, ^7^, ^8^, ^9^] We have previously reported the fragment‐based drug design (FBDD) strategy that we used to develop small molecule inhibitors of *Escherichia coli* DsbA (*Ec*DsbA).[^10^, ^11^, ^12^] Other reported inhibitors of DsbA include peptides based on endogenous sequences that bind to *Ec*DsbA, which have low μM potencies^[13]^ as well as covalent compounds that were targeted at *Ec*DsbB but were also found to bind to *Ec*DsbA.^[14]^ Herein we present our investigation of the development of a phenylthiophene fragment hit into a covalently binding compound. Although the compound initially formed a covalent adduct with *Ec*DsbA, it was unexpectedly found to be turned over. We report our characterization of the mechanism of turnover.

FBDD has also grown over the last two decades to become a widely accepted method for the development of drugs.[^15^, ^16^, ^17^, ^18^, ^19^, ^20^] While a number of advances in screening methods, library design and subsequent development of fragments have been reported, a major hurdle in the implementation of FBDD is that the initial fragments often have low potency for their target and fragments may have multiple modes of binding. Due to the lack of existing potent inhibitors for *Ec*DsbA, one of our initial approaches to improving potency and providing useful chemical probes was to attach a reactive functional group to a fragment hit to allow selective covalent binding to the target. Covalently modifying compounds has become the subject of increased interest due to their unique properties.[^21^, ^22^, ^23^, ^24^, ^25^, ^26^, ^27^] In addition, activity‐based protein profiling has provided numerous examples of selective covalent compounds being used to investigate the proteome.[^28^, ^29^, ^30^] To generate covalent inhibitors, a highly selective reaction with the target protein is desirable to avoid off‐target activity. We hypothesized that this could be achieved by judicious choice of a very low reactivity functional group. Attaching this to a fragment that bound to DsbA would be expected to accelerate the rate and selectivity of reaction with DsbA, by constraining the reactive functional group close to the active site. A similar approach has been successfully employed with high affinity ligands[^31^, ^32^, ^33^] as well as with reversible covalent linkages.[^34^, ^35^] A number of examples have also started with covalently reactive compounds for fragment screening strategies.[^36^, ^37^, ^38^, ^39^]

The thiol at the active site of DsbA enzymes possesses a uniquely low pKa. In *Ec*DsbA, this is the thiol of Cys30 which has a reported pKa ~3.5,^[40]^ which dictates that in the reduced protein it exists predominantly in the thiolate anion form at physiological pH. This makes Cys30 of *Ec*DsbA more nucleophilic than most other thiols, which typically have pKa values of 8–9. Hence a weakly thiolate‐reactive functional group has the potential to generate selectivity for DsbA over other cellular thiols. While a number of thiol selective probes are known,^[25]^ we chose to use fluoromethylketone as a covalent warhead for reaction with *Ec*DsbA. Non‐aryl and peptidyl fluoromethylketones (FMK) have been found to inhibit several proteolytic enzymes (reviewed in);^[41]^ for example FMKs have been reported as covalent inhibitors for many cysteine hydrolases (e. g. caspases),^[42]^ however the covalent inhibition of p90 ribosomal protein S6 kinase (RSK)^[31]^ by an aryl fluoromethylketone (AFMK) demonstrated the weak and selective reactivity that was desired for the current project. Design and synthesis of a fragment linked AFMK was expected to generate a compound that could selectively produce a covalently modified *Ec*DsbA in a cellular setting. To allow for rapid variation of fragment‐AFMK conjugates, the copper (I) catalyzed azide‐alkyne cycloaddition (CuAAC or click)[^43^, ^44^] reaction was used as the key conjugation step of the synthesis, whilst also forming the aryl unit of the AFMK.

## Results and Discussion

**Figure 1:**
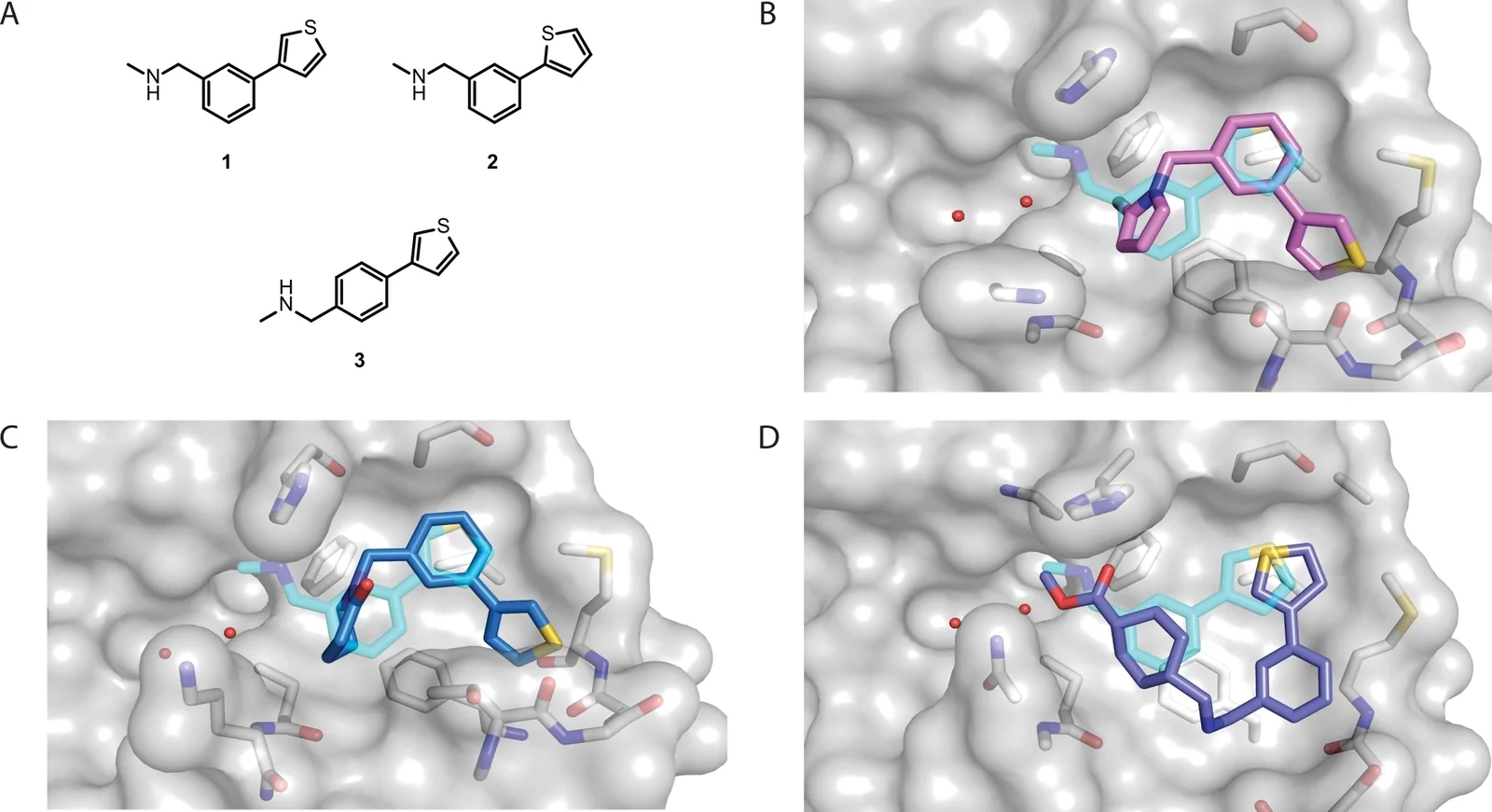
a) Chemical structures of the phenylthiophene fragment series identified via NMR‐based fragment screening. Binding modes determined using X‐ray crystallography of parent compound **1** (cyan: PDBID 7S1C), and analogues b) **38** (pink: PDBID 7S1F), c) 39 (blue: PDBID 7S1D) and d) 72 (purple: PDBID 7S1L) showing all atoms from residues in the protein that are within 5 Å of the ligand.

A prior fragment screen against *Ec*DsbA[^10^, ^45^] resulted in the identification of 8 clusters of structurally similar fragments comprising 26 compounds plus 11 singletons being classified as hits. These were analyzed using X‐ray crystallography, biochemical assay and other biophysical assays. One class for which X‐ray crystallography yielded structures of the fragment bound to *Ec*DsbA consisted of the amino phenylthiophenes **1**, **2** and **3** (Figure 1). Crystal structures were generated by soaking fragments into crystals of *Ec*DsbA in a similar way to our previously reported procedure.^[10]^ The binding mode for **1** and **2** (PDB ID: 6BR4)^[9]^ placed the fragment in a hydrophobic groove of *Ec*DsbA below the active site disulfide (Cys30‐Cys33).

^15^N‐^1^H HSQC NMR titration data for compound **1** did not reach saturation at the highest assayed concentration, suggesting a K_D_ of >1 mM and a ligand efficiency (LE) of <0.29 kcal mol^−1^ HAC^−1^. Despite the modest ligand efficiency, the fact that complexes of *Ec*DsbA bound to this core were amenable to structural characterization by X‐ray crystallography led us to attempt to develop more potent phenylthiophene compounds. Initial development was based on a structure‐activity relationships (SAR)‐by‐catalogue approach followed by synthetic elaboration to optimize and then grow the fragments to improve their affinity for *Ec*DsbA (see SI for full details). ^15^N‐^1^H HSQC spectra were used to characterize binding and compounds were ranked based on the number and extent of chemical shift perturbations (CSPs) observed in the spectra according to our previously reported approach.^[10]^ SAR for replacement of the amino substituent (**4**–**17**), the expansion of the methyl amino substituent (**18**–**49**) and optimization of the aromatic core (**50**–**65**) were explored and characterization of their binding using HSQC NMR spectroscopy and X‐ray crystallography was conducted. Based on the binding information obtained from this characterization two additional series were synthesized (see SI for full details). These were designed to attach a ring onto the amino substituent, to generate a cyclohexyl (**72**–**78**), and a pyridyl (**79**–**81**) series, as well as a series amide linked to the core (**66**–**71** and **82**–**90**). ^15^N‐^1^H HSQC data acquired in the presence of these compounds provided evidence of binding, with CSPs being observed for several residues in the hydrophobic groove of *Ec*DsbA (Supplementary Table 1). Analysis of the concentration dependence of the CSP indicated that the data were not consistent with 1 : 1 binding, with CSP for different residues appearing to saturate at different ligand concentrations (see SI for full details). Nonetheless, the compounds appeared to have residues which showed saturation at 1 mM, indicating an improvement in affinity over the starting phenylthiophenes where no residues had reached saturation. Several of the compounds that bound *Ec*DsbA were soaked into crystals of the protein, although X‐ray crystallography revealed different binding modes for compounds within the series (Figure 1). These results were consistent with the compound binding in multiple orientations giving SAR that was difficult to interpret and also a compound series that was difficult to optimize.

**Table 1:** ^19^F‐NMR investigations of triazolyl‐fluoromethylketone reactivity.

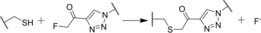					
Sample	AFMK	Thiol	TCEP	pH	Conversion (%) at time by ^19^F‐NMR
1	**98** (1 mM)	DTT (red) (1 mM)	–	7	45 % at 73 h
2	**98** (1 mM)	GSSG (1 mM)	1 mM	7	35 % at 73 h
3	**98** (1 mM)	GSSG (1 mM)	1 mM	12	100 % between 6–20 h
4	**98** (1 mM)	–	1 mM	7	Stable **98** for >51 h
5	**98** (0.1 mM)	DTT (red) (0.1 mM)	–	7	0 % at 44 h
6	**98** (0.1 mM)	DsbA (red) (0.1 mM)	0.2 mM	6.8	60 % at 24 h
7	**97** (0.1 mM)	DsbA (red) (0.1 mM)	0.2 mM	6.8	100 % at 22.5 h

To utilize the increase in binding affinity obtained from the analogue series and regain a consistent binding mode in X‐ray crystallography we adopted the approach of anchoring the phenylthiophenes in the hydrophobic groove through covalent attachment to the active site. We reasoned that the unusual reactivity of *Ec*DsbA′s thiol provided an opportunity to develop selective chemical probes despite the relatively low affinity of this series of fragments. Hence, the initial focus was on synthesis of a fragment‐FMK conjugate. To facilitate development of covalent inhibitors, we implemented a strategy of using the CuAAC reaction of an ethynylFMK and an azide linked fragment for the key linking step to provide a reliable chemistry that was tolerant of the presence of different functional groups and could therefore be used across multiple compound series.

**Scheme 1:**
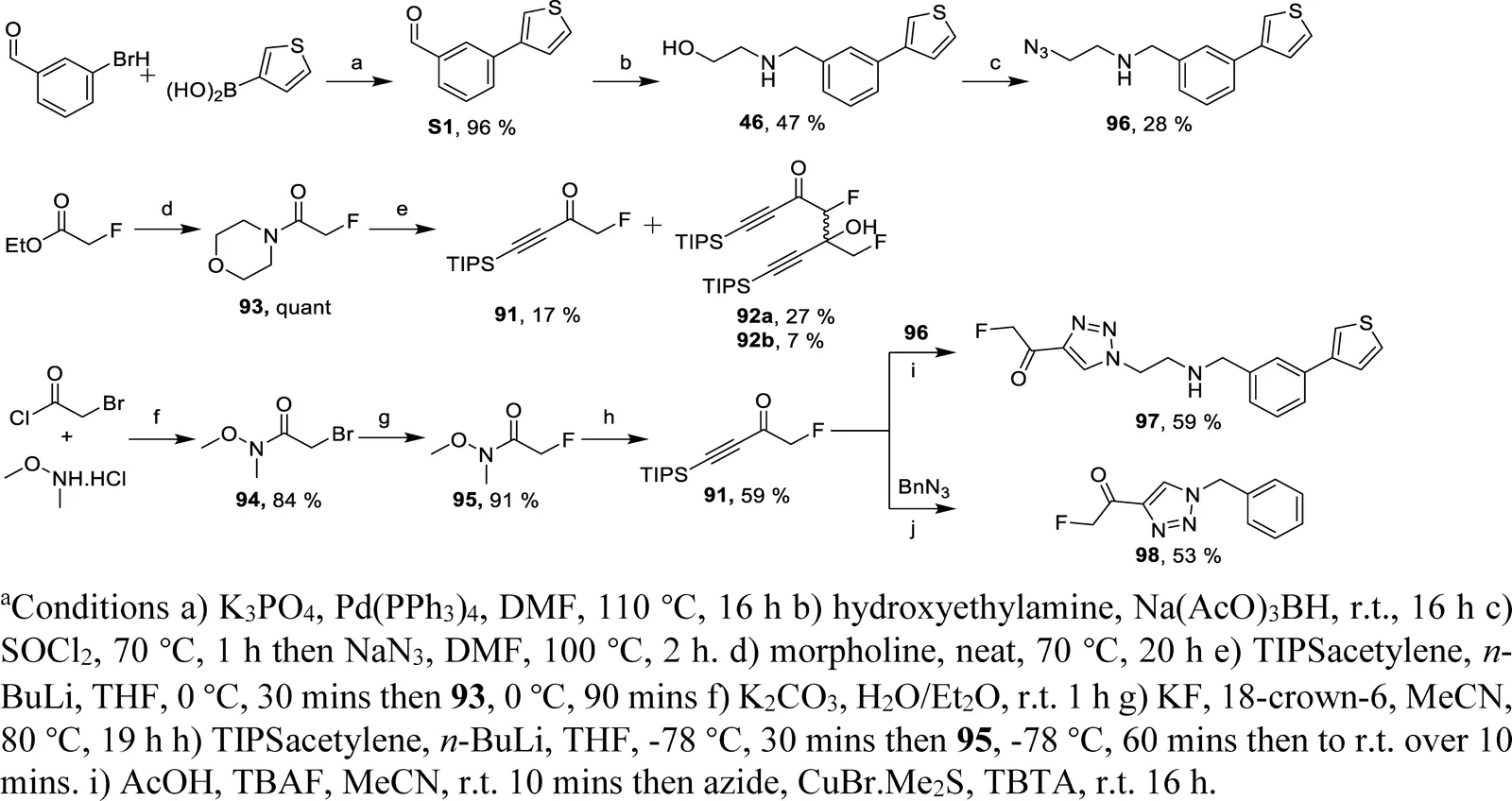
Synthesis of arylfluoromethylketones.^[a]^

A previously reported protocol for synthesis of the silyl protected ethynylFMK from ethyl fluoroacetate,^[46]^ gave low yields and aldol dimer byproducts **92 a** and **92 b** (Scheme 1). Attempts to optimize this reaction were unsuccessful; hence an alternative synthetic pathway using the Weinreb amide **95** was used to produce the TIPS‐protected ethynylFMK **91** in good overall yield (Scheme 1) and no evidence of byproducts **92 a** and **93 b** was found by NMR.

Attention was then turned to the synthesis of a suitable azido‐fragment. Based on analysis of the crystal structure of fragment **1** in complex with *Ec*DsbA (Figure 1) an azide linked by two carbons to the amino group of **1** was predicted to place the AFMK close to the active site Cys30. The azido‐fragment **96** was prepared using a similar synthetic route to phenylthiophene analogues **18**–**90** (Scheme 1). Synthesis of the conjugate fragment‐AFMK **97** and a control compound **98** having only a benzyl substituent rather than the fragment was achieved by reaction of the respective azido compounds **96** and benzyl azide with the ethynylFMK **91** using *in situ* TIPS deprotection and CuAAC conditions (Scheme 1).

The reactivity profile of the AFMKs **97** and **98** was investigated by 1D ^19^F‐NMR, by monitoring the disappearance of the ^19^F AFMK signal (−234 ppm) and appearance of the free fluoride signal (−119 ppm) (Table 1). Reaction of the control benzyl‐AFMK **98** (1 mM) with dithiothreitol (DTT, 1 mM, Sample 1 in Table 1) or glutathione (1 mM, Sample 2) revealed very slow production of fluoride in phosphate buffer (100 mM phosphate, pH 7.0) at 1 mM and the reaction did not reach completion after 73 h. At pH 12 the reaction with glutathione (1 mM, Sample 3) was complete in less than 20 h as expected due to the higher amount of free thiolate present in solution at higher pH. In the reactions of the AFMK compounds with *Ec*DsbA it was necessary to include a reducing agent (tris‐carboxyethylphosphine, TCEP) to prevent aerial oxidation of *Ec*DsbA, which was otherwise observed under the conditions used for the assay. Control experiments with TCEP confirmed the stability of the AFMK (Sample 4) to the phosphine reducing agent, indicating that TCEP did not interfere with the reaction. At 100 μM AFMK **98** and 100 μM DTT (100 mM phosphate, pH 7.0, Sample 5), no product was observed after 44 hours, indicating the desired weak reactivity of the AFMK. Conducting the reaction with *Ec*DsbA (100 μM) at pH 6.8 (50 mM HEPES, 50 mM NaCl) however showed ~60 % loss of the benzyl‐AFMK **98** in 24 h (Sample 6). This confirmed the increased reactivity of *Ec*DsbA to the AFMK compared to DTT suggesting that the compounds did indeed exhibit some degree of selectivity. The fragment‐AFMK **97** (Sample 7) showed complete conversion within 24 h when added to *Ec*DsbA under the same conditions indicating reaction rate was increased by attaching the phenythiophene fragment, concordant with the AFMK‐fragment conjugate binding to *Ec*DsbA and constraining the reactive AFMK group in proximity to the thiol of Cys30.

The progress of the reaction of *Ec*DsbA with the AFMK was monitored by recording a series of ^15^N‐HSQC spectra of reduced *Ec*DsbA after addition of fragment‐AFMK **97**. As expected, the spectra showed progressive loss of cross‐peaks consistent with reduced *Ec*DsbA and the appearance of a new set of resonances. However, the new cross peaks were observed at resonance frequencies of oxidized *Ec*DsbA. The addition of TCEP at this point reverted the resonance frequencies observed in the ^15^N‐HSQC spectrum to those of reduced *Ec*DsbA.

**Figure 2:**
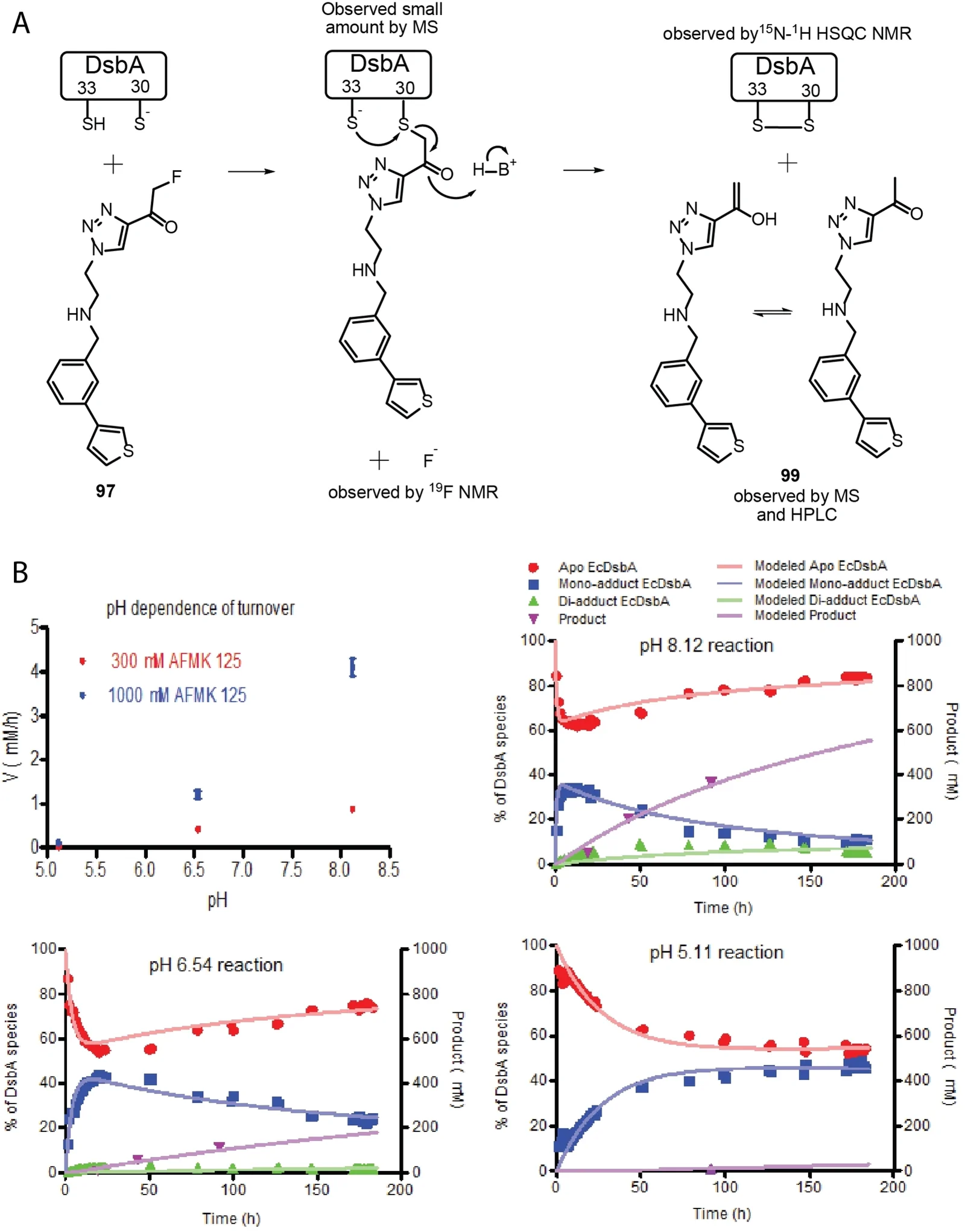
a) Proposed mechanism of turnover of Cys30 thioether labeled *Ec*DsbA b) pH dependence of fragment‐AFMK **97** turnover by *Ec*DsbA monitored by HPLC. Reaction conditions: 125 mM citrate‐phosphate buffer, 20 μM *Ec*DsbA, 2 mM TCEP, 41–360 mM NaCl, 2 % DMSO. LCMS and HPLC monitoring of *Ec*DsbA species and product **99** at pH 5.11, 6.54 and 8.12. Reaction conditions: 125 mM citrate‐phosphate buffer, 20 μM *Ec*DsbA, 2 mM TCEP, 41–360 mM NaCl, 2 % DMSO, 1000 μM fragment‐AFMK **97**.

The appearance of resonances consistent with oxidized *Ec*DsbA suggested that the covalent adduct of AFMK **97** with *Ec*DsbA was unexpectedly being turned over to release oxidized *Ec*DsbA. LCMS analysis of the reaction mixture also revealed a product having a mass consistent with the formation of the des‐fluoro phenylthiophene **99** in the reaction mixture (Figure 2). These observations led us to propose a mechanism for resolution of the covalent complex whereby the second thiol of *Ec*DsbA (Cys33) attacks the thioether to give an oxidized *Ec*DsbA species and desfluoro **99**. This requires the displacement of an enol group, in a process which we hypothesize is catalyzed by a nearby weakly acidic residue (possibly the proximal His32 imidazolium which is thought to stabilize the thiolate form of Cys33), or solvent (Figure 2).

Further support for this turnover mechanism was obtained by the synthesis of an authentic sample of the desfluoro **99** which had identical MS fragmentation and HPLC profiles. Desfluoro **99** was synthesized using the same methods as for fragment‐AFMK **97** with modified starting materials. The production of an authentic sample also enabled HPLC monitoring of the reaction to investigate the kinetics of the turnover. However, under conditions suitable for this analysis (20 μM *Ec*DsbA, 200–1600 μM fragment‐AFMK **97**, 50 mM HEPES, 50 mM NaCl, pH 6.8, 25 °C, 0–8–1.6 % DMSO) the rate was too slow to be determined accurately (estimated k_cat_ of 0.035±0.002 h^−1^ and a K_m_ of 110±30 μM). In an attempt to increase the rate of reaction we investigated the effect of pH. Reactions were monitored by HPLC at pH 5.11, 6.54 and 8.12 in citrate‐phosphate buffer (125 mM, 20 μM *Ec*DsbA, 2 mM TCEP, 41–360 mM NaCl, 2 % DMSO) at two concentrations of fragment‐AFMK **97** (300 and 1000 μM) and showed increased rates at higher pHs at both concentrations (Figure 2).

The increased rate of turnover between pH 6.54 and 8.12 could not be explained by a higher proportion of Cys30 existing in the thiolate form as at both pHs Cys30 is expected to be >99.9 % ionized. The data however could be explained by an increased amount of Cys33 thiolate, proposed as the nucleophile in turnover of the intermediate, although changes in pH may also affect the solubility of **97** and its affinity for *Ec*DsbA.

The pH dependence was further investigated by LCMS monitoring the relative amount of *Ec*DsbA species using the same reaction conditions at each pH with 1000 μM fragment‐AFMK **97** (Figure 2). The reaction profiles at pH 6.54 and 8.12 show the intermediate mono‐adduct accumulates relatively quickly, with significant buildup within a few hours. This is consistent with the initial labeling step being of a similar rate in each case, which is not surprising given the expected similar extent of ionization of Cys30 under these conditions. However, at pH 8.12 the adduct is turned over more rapidly to generate oxidized *Ec*DsbA, indicating an increased rate of turnover (relative to labeling) consistent with a greater extent of ionization of Cys33 thiolate at this pH. At pH 5.11 the rate of formation of the mono‐adduct is relatively slow compared to that observed at pH 6.54 and 8.12. This is also consistent with the expected extent of ionization of Cys30 and is concordant with the reduced rate of product formation observed, due to both a decrease in the rate of labeling as well as a decrease in the rate of turnover. In addition, a relatively low intensity species consistent with a di‐adduct was observed with increasing pH, suggesting labeling of Cys33 by the AFMK **97** or a further non‐thiol mediated reaction with *Ec*DsbA. The kinetic data fit well to a simple model in which the reduced *Ec*DsbA binds to the fragment‐AFMK **97** and undergoes covalent reaction (Figure 2, see SI for full details). This mono‐adduct can either turnover (as outlined in Figure 2) or *Ec*DsbA can react with a second molecule of AFMK **97** (to account for the observed di‐adduct). The oxidized *Ec*DsbA product release on turnover would be expected to react very rapidly with TCEP to regenerate reduced *Ec*DsbA.

To confirm the Cys33 mediated turnover mechanism and investigate the di‐adduct formation, a C33 A *Ec*DsbA mutant was prepared and monitored by LCMS which showed >80 % mono‐adduct formation after 5 hours and no change for a further 7 days. No di‐adduct was detected. This provides strong evidence that the second labeling site is Cys33 and further confirms the role of Cys33 in the proposed turnover mechanism as well as the thiol‐selective nature of the AFMK.

Due to the instability of the AFMK labeled *Ec*DsbA we also investigated the reaction with maleimide. Although the labeling mechanism is different (Michael addition) the covalent complex that is produced still has an α‐thiocarbonyl intermediate, which is the moiety proposed to be required for turnover. The reaction of maleimide with *Ec*DsbA was monitored by ^15^N‐^1^H HSQC; after 20 hours resonances consistent with the presence of oxidized *Ec*DsbA were observed. MS monitoring of a sample for 12 hours showed rapid and complete conversion to the covalent adduct followed by slow conversion back to an apo *Ec*DsbA species, consistent with a similar turnover mechanism to that observed for the AFMK **97**. The ability of *Ec*DsbA to turn over the maleimide labeled intermediate should be noted for future investigations of maleimide labeled DsbA enzymes and possibly other dithiol oxidoreductases, which are often presumed to create irreversible covalent linkages. The same mechanism may also exist for other well‐known thiol labeling reagents such as iodoacetamide and 1,4‐quinone based reactive functional groups.

## Conclusions

Herein we report the design and synthesis of a clickable alkynyl‐FMK **91** for use in production of selective, covalent, thiolate‐reactive compounds. Reaction of this species with an azido derivative of a low affinity fragment allowed for rapid synthesis of the fragment‐AFMK conjugate **97** reported in this study. Investigations by ^19^F NMR revealed an increased rate of formation of covalent adduct with *Ec*DsbA when the AFMK reactive functional group was attached to a low affinity fragment compared to a control benzyl group. In addition, selectivity for *Ec*DsbA over other thiols was observed for the AFMK. Further investigation by MS, ^15^N‐^1^H HSQC NMR and HPLC supports the conclusion that the covalent complex is not stable and can be turned over to give an oxidized *Ec*DsbA and a desfluoro product. Additional investigations suggest that a similar turnover mechanism may operate with maleimide. *Ec*DsbA therefore possesses an ability to turn over α‐thioether carbonyl intermediates leading to a formal reductive defluorination of AFMKs and reduction of maleimides to succinimides.

## Methods

### Protein Expression and Purification

^15^N isotopically enriched *Ec*DsbA was expressed as previously described.^[10]^ A plasmid, pC33A, for the expression of the C33A mutant of *Ec*DsbA (EcC33A), was created by site‐directed mutagenesis of the plasmid B0013, using the primers ′C33A_f2′ (ctctttcttctgcccgcacgcctatcagtttgaagaagtt) and ′C33A_rev2′ (aacttcttcaaactgataggcgtgcgggcagaagaaagag). The plasmid pC33A was used to transform BL21(DE3)‐Gold (Agilent) *E. coli* K12 cells and these were used for protein expression. *Ec*C33A was expressed and purified using the protocol described above for ^15^N isotopically‐enriched *Ec*DsbA, with the following exceptions: natural isotope abundance NH_4_Cl was used in the autoinduction broth, and at the final stage of purification, the protein was buffer exchanged into 50 mM Na_2_HPO_4_, 50 mM NaCl, pH 6.54.

### Crystallization and Structure Determination

*Ec*DsbA‐compound complexes were crystallized and structures were determined as previously described.^[10]^ Data collection and refinement statistics are summarized in Table S2.

### Macromolecular HSQC NMR Experiments

A reference ^1^H‐^15^N HSQC spectrum of uniformly labelled ^15^N oxidized *Ec*DsbA (~100 μM protein, 1 % *d_6_*‐DMSO, 50 mM HEPES, 50 mM NaCl, pH 6.8 in 10 % D_2_O) was acquired. A series of ^1^H‐^15^N HSQC spectra was acquired under the same conditions in the presence of a sample fragment (100–1000 μM).

### ^19^F NMR Experiments

Samples were prepared to desired concentration (100 or 1000 μM) in phosphate buffer (100 mM, pH 7), 10 % D_2_O and pH adjusted with NaOH_aq_ as required. Samples of thiol (*Ec*DsbA (100 μM), dithiothreitol (DTT) or oxidized glutathione (GSSG)) were treated with tris(carboxyethyl)phosphine (TCEP) if required and left at room temperature for 15 mins before the addition of FMK containing compounds. The reactions were then transferred to an NMR tube and monitored at the specified intervals by ^19^F NMR.

### HPLC Determination of Kinetic Parameters

Samples of 20 μM *Ec*DsbA, 200–1600 μM AFMK **97**, 2 mM TCEP, in 50 mM HEPES, 50 mM NaCl, pH 6.8, 0.8–1.6 % DMSO were allowed to react at 25 °C for up to 7 days. Samples were taken periodically and diluted into equal volumes of acetonitrile to quench the reaction. RP‐HPLC was then performed on a Phenomenex Luna 5 μ C8 (2) 100 A (150×4.60 mm ID) column monitoring at 254 nm. A gradient of 15–45 % Buffer B in Buffer A over 16 mins, followed by a gradient to 100 % Buffer B over 1 min, followed by a gradient to 15 % Buffer B over 1 min and a further 2 mins isocratic 15 % Buffer B, at a flow rate of 1.0 mL/min was used. Concentration of product in the reaction was then calculated based on area of peak with reference to a standard curve. The rates for the first 10–15 % of reaction were determined by linear regression and *K_m_* and V_max_ determined by non‐linear regression fitting to the Michaelis‐Menten equation using GraphPad Prism 5.

### LCMS Monitoring of Protein Species

Samples were analyzed on a Shimadzu LCMS‐20 system with an SGE ProteoCol C8 HQ1003 150 mm×2.0 mm ID 3 μm 1000 Å column or SGE ProteoCol C18 HQ303 150 mm×2.0 mm ID 3 μm 300 Å column monitoring at 214 nm. Buffer A: 99.9 % water, 0.1 % formic acid; buffer B: 99.9 % acetonitrile, 0.1 % formic acid. A gradient of 0–60 % buffer B over 16 mins followed by 1 min isocratic 60 % buffer B, 1 min gradient to 100 % buffer A and 2 min of isocratic 100 % buffer A at a flow rate of 0.2 mL/min was used as an elution profile. Data was analyzed using LabSolutions v5.31 and mMass v5.0.1. Samples of 20 μM *Ec*DsbA, 1000 μM AFMK **97** in 125 mM citrate‐phosphate buffer (at pH 5.11, 6.54 and 8.12), 2 mM TCEP, 41–360 mM NaCl, 2 % DMSO were allowed to react at 25 °C for 7 days. Samples were taken periodically and directly injected and the ratio of apo, mono‐adduct and di‐adduct determined.

### Accession Codes

The crystallographic coordinates were deposited in the Protein Data Bank under PDB codes 7S1D, 7S1F, 7S1L and 7S1C.

## Abbreviations

CuAAC: Copper (I) catalysed azide‐alkyne cycloaddition
*Ec*
: *Escherichia coli*
DTT: Dithiothreitol
FBDD: Fragment‐based drug design
AFMK: Aryl Fluoromethylketone
HEPES: 4‐(2‐Hydroxyethyl) piperazine‐1‐ethanesulfonic acid
HSQC: Heteronuclear single quantum coherence
HTS: High throughput screening
TBTA: Tris (*N*1‐Benzyltriazol‐4‐ylmethyl) amine
TCEP: Tris (2‐carboxyethyl) phosphine
TMS: Trimethylsilyl
TIPS: Triisopropylsilyl
GSSG: Glutathione (oxidized)

## Conflict of Interests

The authors declare no conflict of interest.

## Supporting information

As a service to our authors and readers, this journal provides supporting information supplied by the authors. Such materials are peer reviewed and may be re‐organized for online delivery, but are not copy‐edited or typeset. Technical support issues arising from supporting information (other than missing files) should be addressed to the authors.

Supporting Information

## Data Availability

Structural data reported in the manuscript have been submitted to the pdb. Other data are available from the authors on request.
