# Supplementary material for: Fluoromethylketone‐Fragment Conjugates Designed as Covalent Modifiers of EcDsbA are Atypical Substrates
Source: ChemMedChem. 2024 Jul 8;19(16):e202300684. doi: 10.1002/cmdc.202300684 (PMC13494810; doi:10.1002/cmdc.202300684)

ChemMedChem

Supporting Information

Fluoromethylketone-Fragment Conjugates Designed as Covalent Modifiers of *Ec*DsbA are Atypical Substrates

Bradley C. Doak, Rebecca L. Whitehouse, Kieran Rimmer, Martin Williams, Begoña Heras, Sofia Caria, Olga Ilyichova, Mansha Vazirani, Biswaranjan Mohanty, Jason B. Harper, Martin J. Scanlon,* and Jamie S. Simpson*

Fluoromethylketone-Fragment Conjugates Designed as Covalent Modifiers of *Ec*DsbA are Atypical Substrates

Bradley C. Doak[1‡], Rebecca L. Whitehouse[1‡], Kieran Rimmer[1], Martin Williams[1], Begoña Heras[2], Sofia Caria[1], Olga Ilyichova[1], Mansha Vazirani[1], Biswaranjan Mohanty[1], Jason B. Harper[3], Martin J. Scanlon[1,4*] and Jamie S. Simpson[1*]

[1]Medicinal Chemistry, [4]ARC Centre for Fragment-Based Design, Monash Institute of Pharmaceutical Sciences, Monash University, 381 Royal Parade, Parkville, VIC, 3052, Australia.[2] Department of Biochemistry and Genetics, La Trobe Institute for Molecular Science, La Trobe University, Kingsbury Drive, Bundoora, Vic 3083, Australia [3]School of Chemistry, University of New South Wales, Sydney, NSW 2052, Australia [‡]Authors share first authorship.

SUPPORTING INFORMATION

1. General experimental 2
2. Protein expression and purification 3
3. Macromolecular HSQC NMR experiments 4
4. HPLC determination of kinetic parameters 5
5. Spectra for ^{19}F NMR monitoring of reaction 6
6. LCMS monitoring of protein species 10
7. Modelling of reaction kinetics 11
8. Synthesis of compounds S1-S26 14
9. Synthesis of compounds 1 and 18-101 27
10. Comparative ranked screening results of compounds 1-90 87
11. X-ray crystallography of compounds 91
12. References 96
13. NMR and HPLC of compounds S1, 46, 91-100, ... 98
14. ^{15}N-^{1}H HSQC titration and residue K_D fits of compound 39 126

1. General experimental

NMR spectra were recorded at either 300 MHz for ^{1}H-NMR or 75 MHz for ^{13}C-NMR on a Bruker Avance 300 MHz NMR spectrometer, or at 400 MHz for ^{1}H-NMR, 100 MHz for ^{13}C-NMR and 367 MHz for ^{19}F-NMR on a Bruker Avance 400 MHz NMR spectrometer, or at 600 MHz for ^{1}H-NMR and 150 MHz for ^{13}C-NMR on a Varian Inova 600 MHz NMR spectrometer at 298 K. Data acquisition and processing was managed using TOPSPIN/ICONNMR (Bruker) software package and processing/plotting was managed using MestReNova 6.0.2. All chemical shifts for ^{1}H-NMR spectra were measured in parts per million (ppm) referenced to an internal standard of residual proteo-solvent, δ 7.26 for chloroform and δ 3.31 for methanol.[1] ^{13}C-NMR resonances were measured in parts per million (ppm) referenced to the residual proteo-solvent, δ 77.16 for $CDCl_3$ and δ 49.00 for d_4-methanol.[1] Multiplicity is quoted as a (apparent), b (broad), s (singlet), d (doublet), t (triplet), q (quartet), p (pentet) and m (multiplet). High Resolution Mass Spectra were collected on a Waters Micromass LCT Premier XE time-of-flight mass spectrometer fitted with an electrospray (ESI) ion source and controlled with MassLynx software version 4.5. Low Resolution Mass Spectrometry analyses were performed using a Micromass Platform II single quadrupole mass spectrometer equipped with an atmospheric pressure (ESI/APCI) ion source. Sample management was facilitated by an Agilent 1100 series HPLC system and the instrument was controlled using MassLynx software version 3.5. Analytical RP-High Performance Liquid Chromatography (RP-HPLC) was conducted on a Waters 2690 Separation Module coupled with a Waters 996 Photodiode Array Detector with a Phenomenex Luna 5μ C8 (2) 100A (150 × 4.60 mm ID) column monitoring at 254 and/or 214 nm. Buffer A: 99.9 % Water, 0.1 % TFA, Buffer B: 80 % Methanol, 19.9 % Water, 0.1 % TFA. A gradient of 20-100 % Buffer B in Buffer A over 10 mins, followed by isocratic 100 % Buffer B for a further 6 mins followed by a gradient of 100-20 % Buffer B over 1 min and isocratic 20 % Buffer B for 3 mins, at a flow rate of 1.0 mL/min was used.

Empower Pro software managed the running and processing of samples. Analytical NP-High Performance Liquid Chromatography (NP-HPLC) was conducted on an Agilent 1260 infinity with an Alltech Econosphere silica 5μ (250 × 4.6 mm ID) column at 254 nm. An isocratic method of 20 % ethanol in petroleum spirits was used. Agilent Chemstation software managed the running and processing of samples. Thin layer chromatography (TLC) was performed on Merck Silica Gel 60 F_{254} plates. TLC plates were visualized under UV illumination at 254 nm and/or with the aid of phosphomolybdic acid (PMA), vanillin, *p*-anisaldehyde, iodine or potassium permanganate solutions. Column chromatography was conducted using Davisil silica gel LC60A (40-63 microns). All commercially available chemicals were purchased from either Aldrich/Fluka or Merck, stored appropriately and used as required. Amino acid methyl esters were prepared from the amino acids according to the procedure of Gros *et al.*[8] and analytical data were concordant with literature values.[8] Palladium *tetrakis*(triphenylphosphine) was prepared according to the procedure of Dubreuil *et al.*[5] Hexane and ethyl acetate was distilled before use. HPLC-grade THF was dried using an Mbraun solvent purification system (SPS-800) according to manufacturer's instructions. All HPLC solvents were HPLC grade, all other solvents were reagent grade and used as required.

2. Protein expression and purification

^{15}N isotopically enriched *Ec*DsbA was overexpressed by autoinduction.[2] Briefly, minimal media containing $^{15}NH_4Cl$ as the sole nitrogen source; glycerol as the primary carbon source and glucose and lactose as sequential repressor and inducer of protein expression, respectively; trace metals, thiamine and Kanamycin, was inoculated with an overnight culture of *E. coli* strain BL21(DE3) carrying the plasmid B0013-(5644bb). After 6 hours cell growth at 37 °C with agitation at 200 rpm, the temperature was reduced to 28 °C and the expression culture was incubated for a further 24 hours before cell harvest by centrifugation at 3200 × g. For extraction

of the target protein from the cell periplasm, the cells were lysed by osmotic shock[3] and cell debris was removed by centrifugation (40000 × g). Uniformly ^{15}N labelled *Ec*DsbA was purified using Hydrophobic Interaction Chromatography followed by Anion-Exchange Chromatography (ÄKTA FPLC, GE Healthcare), oxidized using copper phenanthroline and buffer exchanged to 50 mM HEPES (pH 6.8), 50 mM NaCl. The protein was concentrated by diafiltration (Amicon, 3000 Da MWCO, Millipore), and 10 % v/v D_2O was added for a final protein concentration of 100 µM.

A plasmid, pC33A, for the expression of the C33A mutant of *Ec*DsbA (EcC33A), was created by site-directed mutagenesis of the plasmid B0013, using the primers 'C33A_f2' (ctctttcttctgcccgcacgcctatcagtttgaagaagtt) and 'C33A_rev2' (aacttcttcaaactgataggcgtgcgggcagaagaaagag). The plasmid pC33A was transformed into BL21(DE3)-Gold (Agilent) *E. coli* K12 cells and these were used for protein expression. *Ec*C33A was expressed and purified as per the protocol described above for ^{15}N isotopically-enriched *Ec*DsbA, with the following exceptions: natural isotope abundance NH_4Cl was used in the autoinduction broth, and at the final stage of purification, the protein was buffer exchanged into 50 mM Na_2HPO_4, 50 mM NaCl, pH 6.54.

3. Macromolecular HSQC NMR experiments

Final sample conditions were ~100 µM ^{15}N *Ec*DsbA, 50 mM HEPES, 50 mM NaCl, pH 6.8, 1 % d_6-DMSO, 10% D_2O, unless specified otherwise. ^{1}H-^{15}N HSQC spectra were acquired at 298 K on a Varian Inova 600 MHz spectrometer equipped with standard 5 mm bore, triple-resonance pulsed field gradient probe head and TCI Cryoprobe. Data were recorded with spectral widths of 14 ppm and 34 ppm in the ^{1}H and ^{15}N dimensions respectively, acquired over 8 transients with 64 complex points in the direct ^{1}H dimension, 128 increments in the indirect ^{15}N dimension and a 1 s recycle delay, for a total acquisition time of 20 minutes; or

spectral widths of 15 ppm and 28 ppm in the ^{1}H and ^{15}N dimensions respectively, acquired over 4 transients with 1098 complex points in the direct ^{1}H dimension, 128 increments in the indirect ^{15}N dimension and a recycle delay of 1.4 s for a total acquisition time of 27.5 minutes. All data were acquired with Varian Biopack, processed with Topspin 3.0 (Bruker) and analyzed with Sparky(UCSF)[4].

4. HPLC determination of kinetic parameters

Samples of 20 μM *Ec*DsbA, 200-1600 μM AFMK **97**, 2 mM TCEP, in 50 mM HEPES, 50mM NaCl, pH 6.8, 0.8-1.6 % DMSO were allowed to react at 25 °C for up to 7 days. Samples were taken periodically and diluted into equal volumes of acetonitrile to quench the reaction. RP-HPLC was then performed on a Phenomenex Luna 5μ C8 (2) 100A (150 × 4.60 mm ID) column monitoring at 254 nm. A gradient of 15-45 % Buffer B in Buffer A over 16 mins, followed by a gradient to 100 % Buffer B over 1 min, followed by a gradient to 15 % Buffer B over 1 min and a further 2 mins isocratic 15 % Buffer B, at a flow rate of 1.0 mL/min was used. Concentration of product in the reaction was then calculated based on area of peak with reference to a standard curve. The rates for the first 10-15 % of reaction were determined by linear regression and K_m and V_{max} determined by non-linear regression fitting to the Michaelis-Menten equation using GraphPad Prism 5.

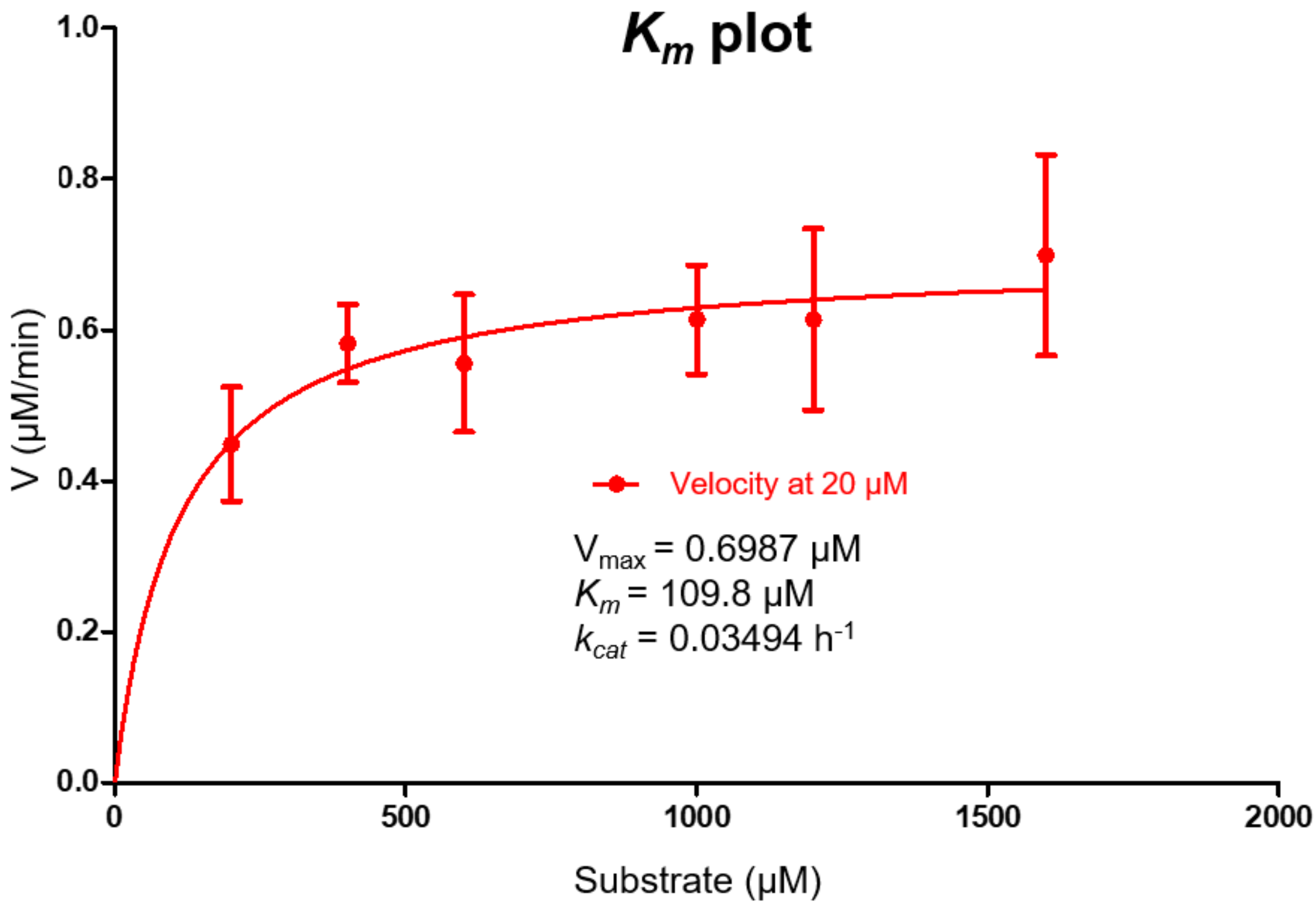

5. Spectra for ^{19}F NMR monitoring of reaction

Sample	AFMK	Thiol	TCEP	pH	Extent of conversion
1	**98** (1 mM)	DTT (red) (1 mM)	-	7	45 % at 73 h
2	**98** (1 mM)	GSSG (1 mM)	1 mM	7	35 % at 73 h
3	**98** (1 mM)	GSSG (1 mM)	1 mM	12	100 % between 6-20 h
4	**98** (1 mM)	-	1 mM	7	Stable **98** for >51 h
5	**98** (0.1 mM)	DTT (red) (0.1 mM)	-	7	0 % at 44 h
6	**98** (0.1 mM)	DsbA (red) (0.1 mM)	0.2 mM	6.8	60 % at 24 h
7	**97** (0.1 mM)	DsbA (red) (0.1 mM)	0.2 mM	6.8	100 % at 22.5 h

Samples were prepared in phosphate buffer (100 mM, pH 7), 10% D_2O, adjusted to pH 12 by addition of $NaOH_{aq}$ and acquired at a final volume of 500 µL.

Sample 1)

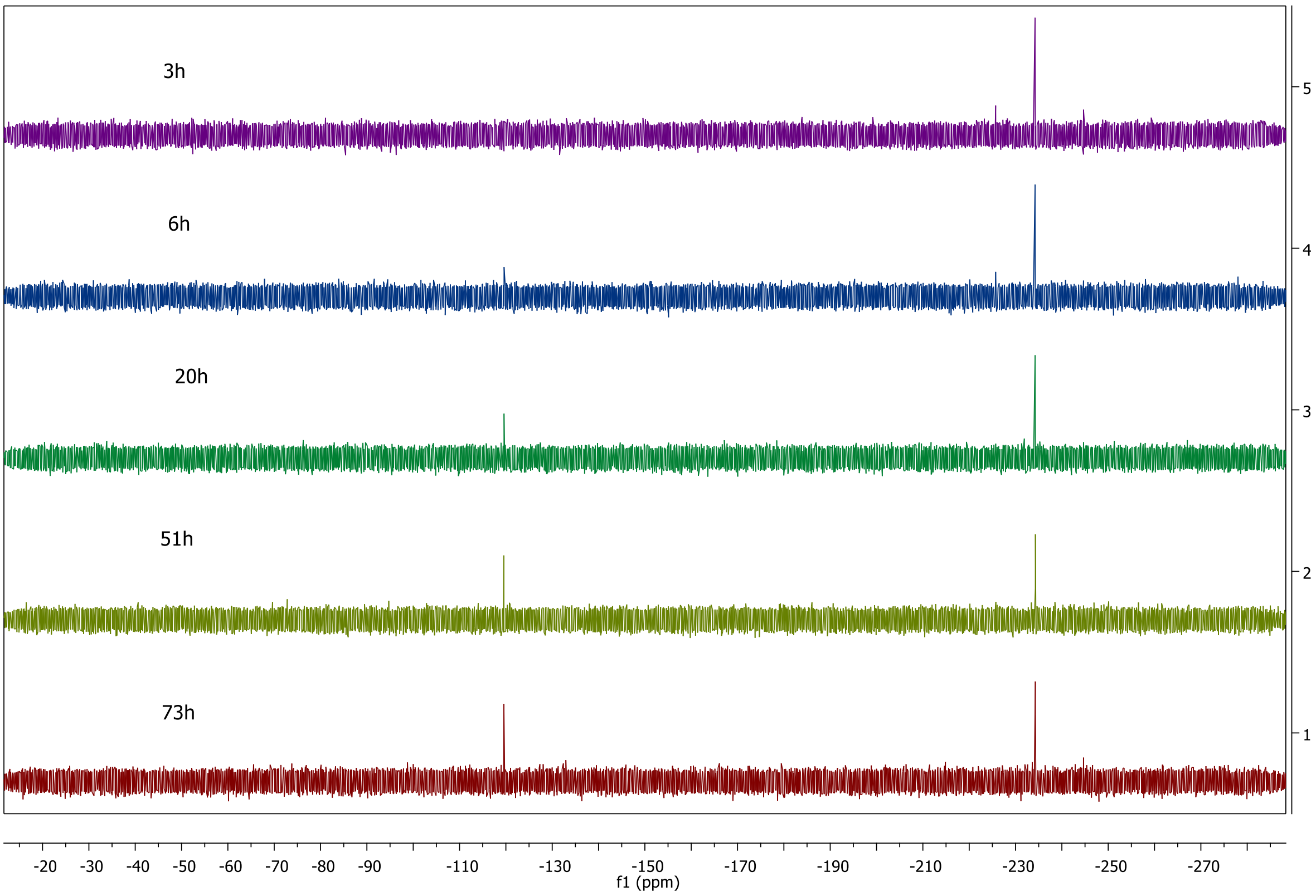

Sample 2)

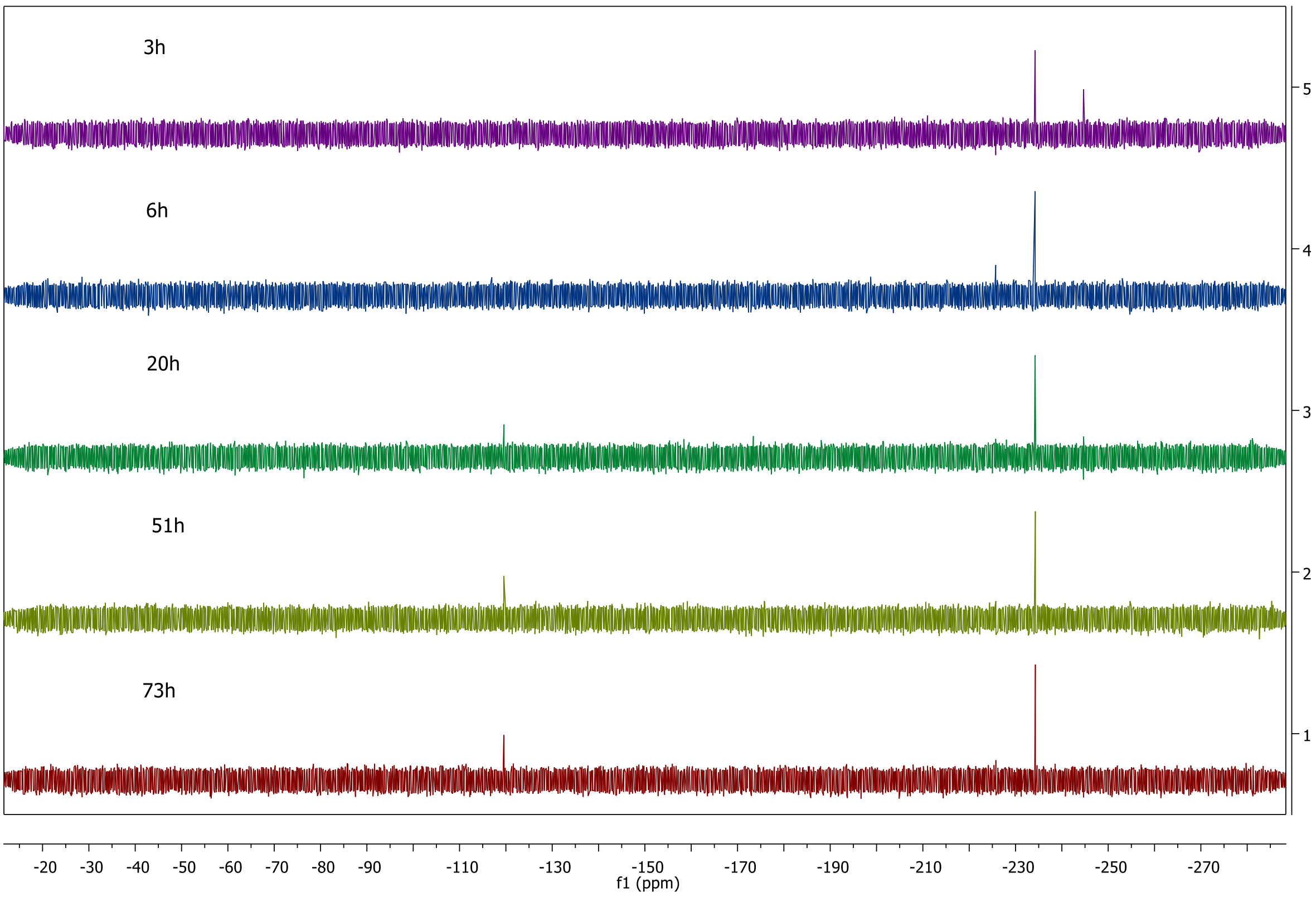

Sample 3)

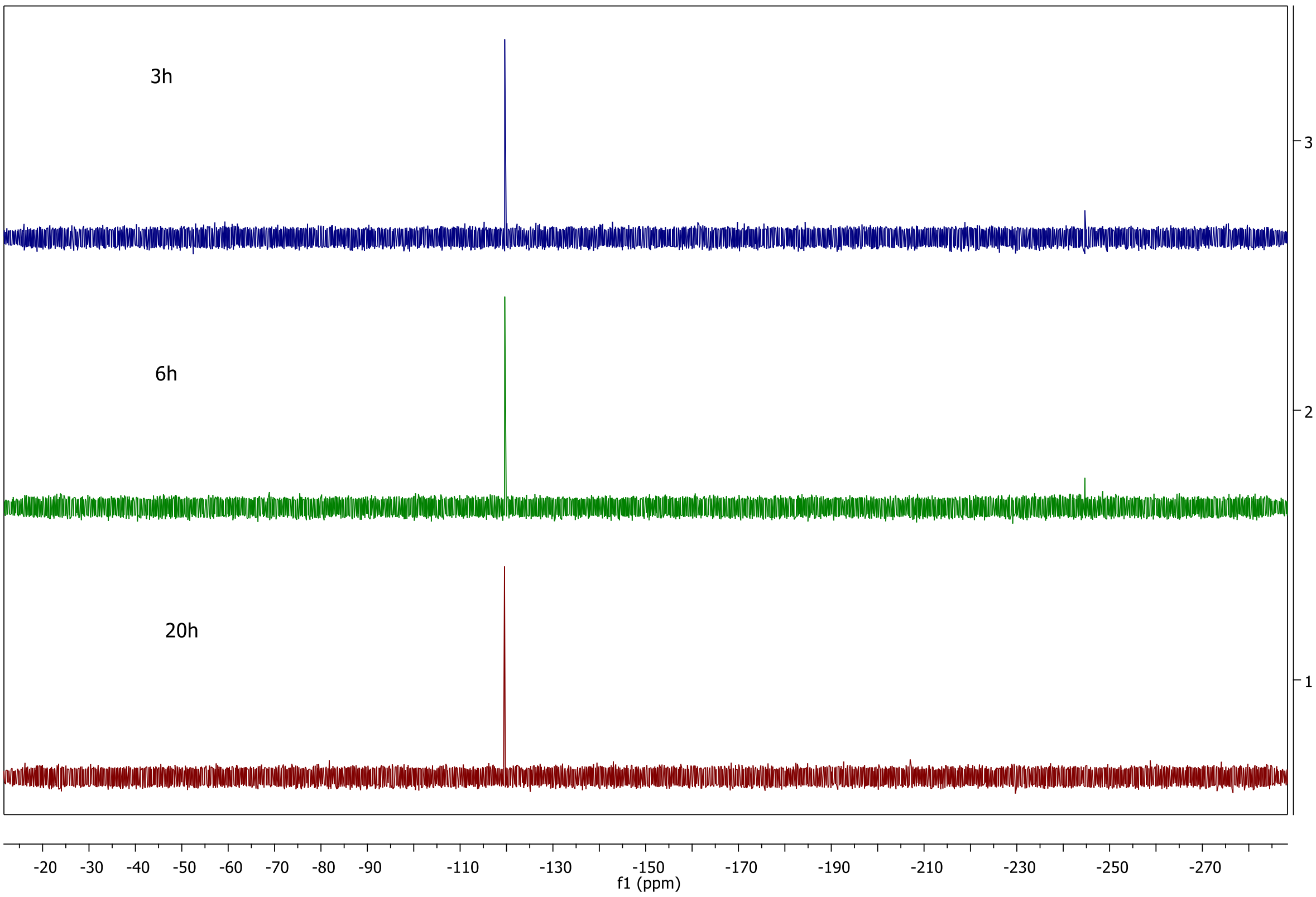

Sample 4)

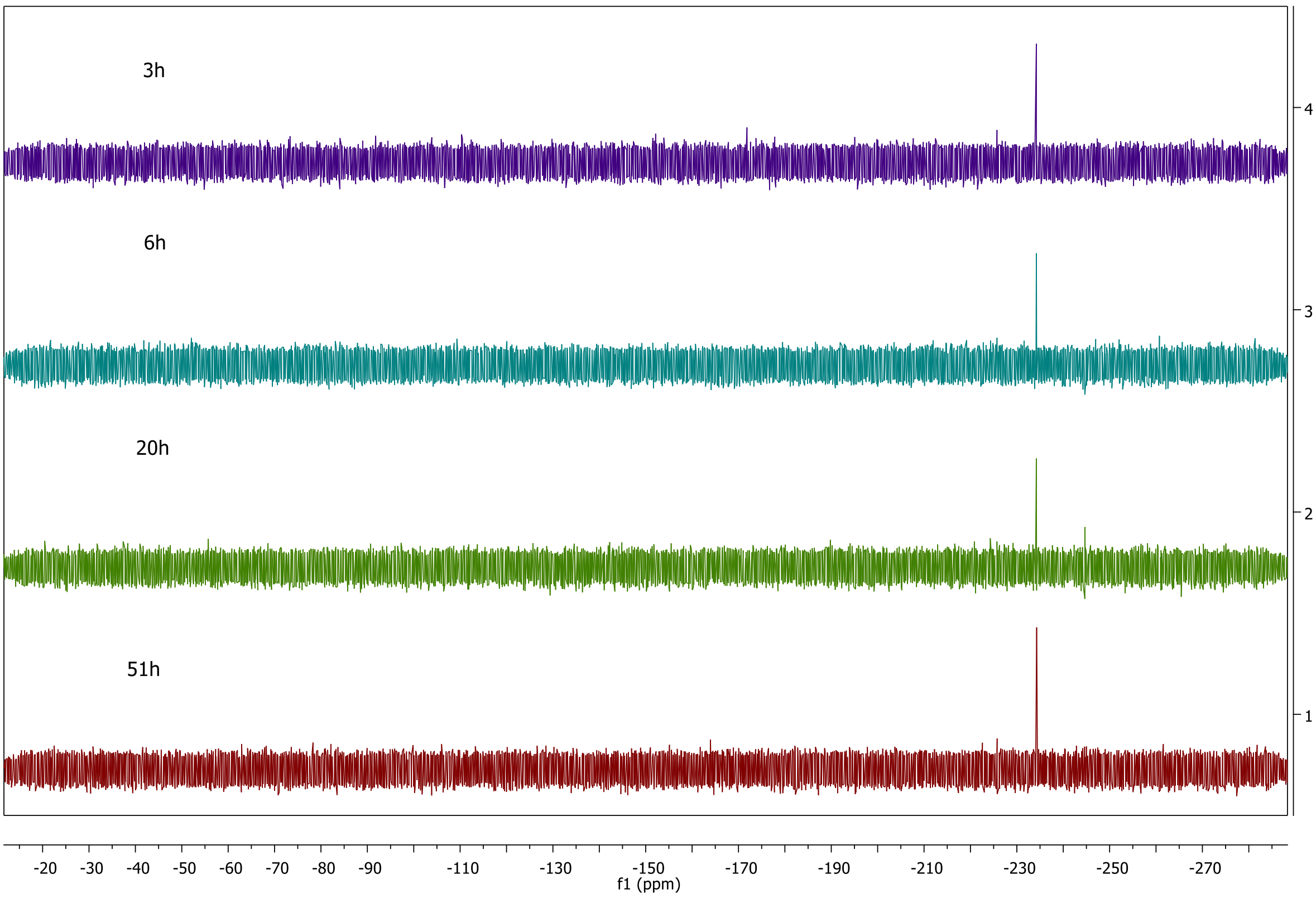

Sample 5)

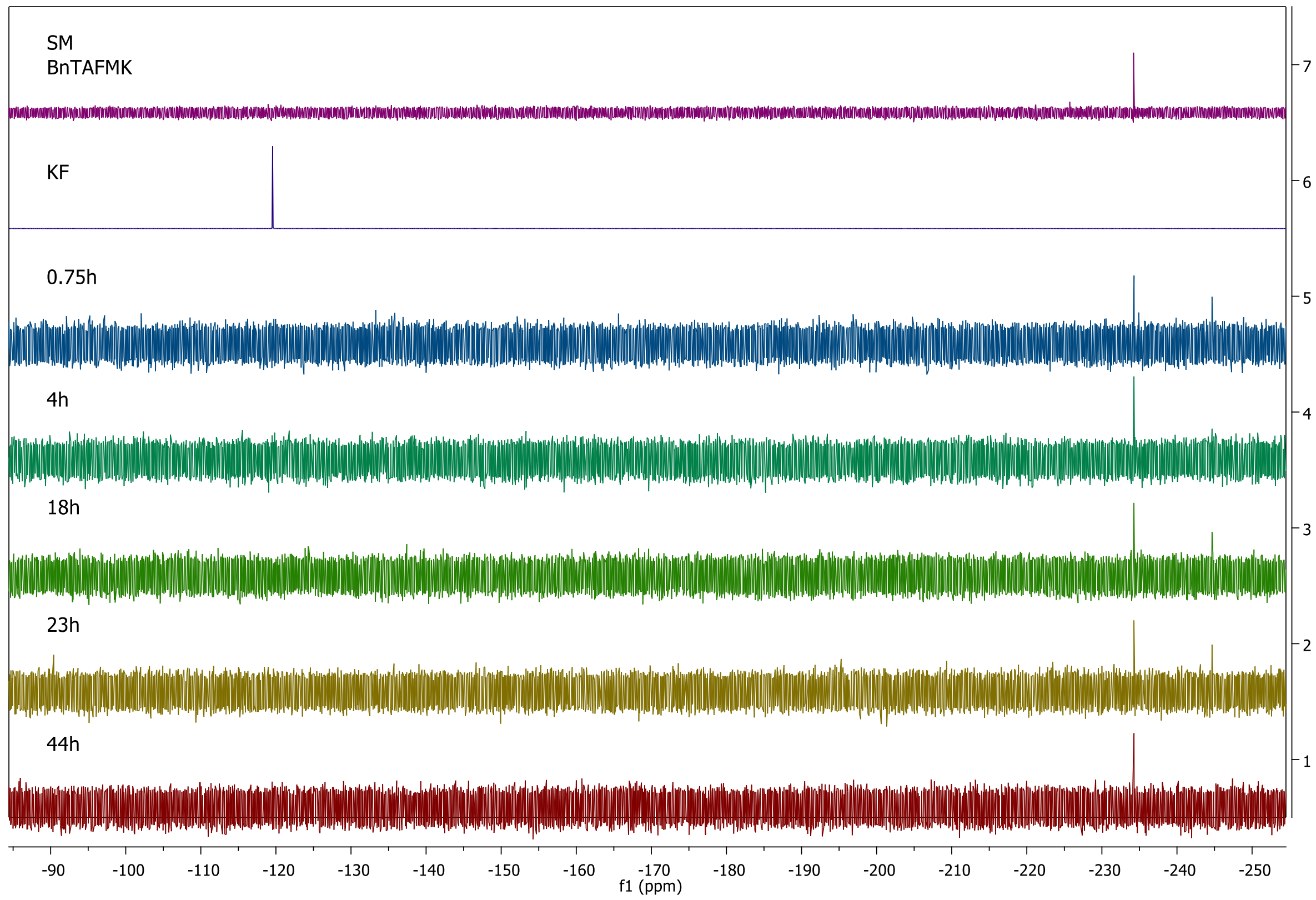

Sample 6)

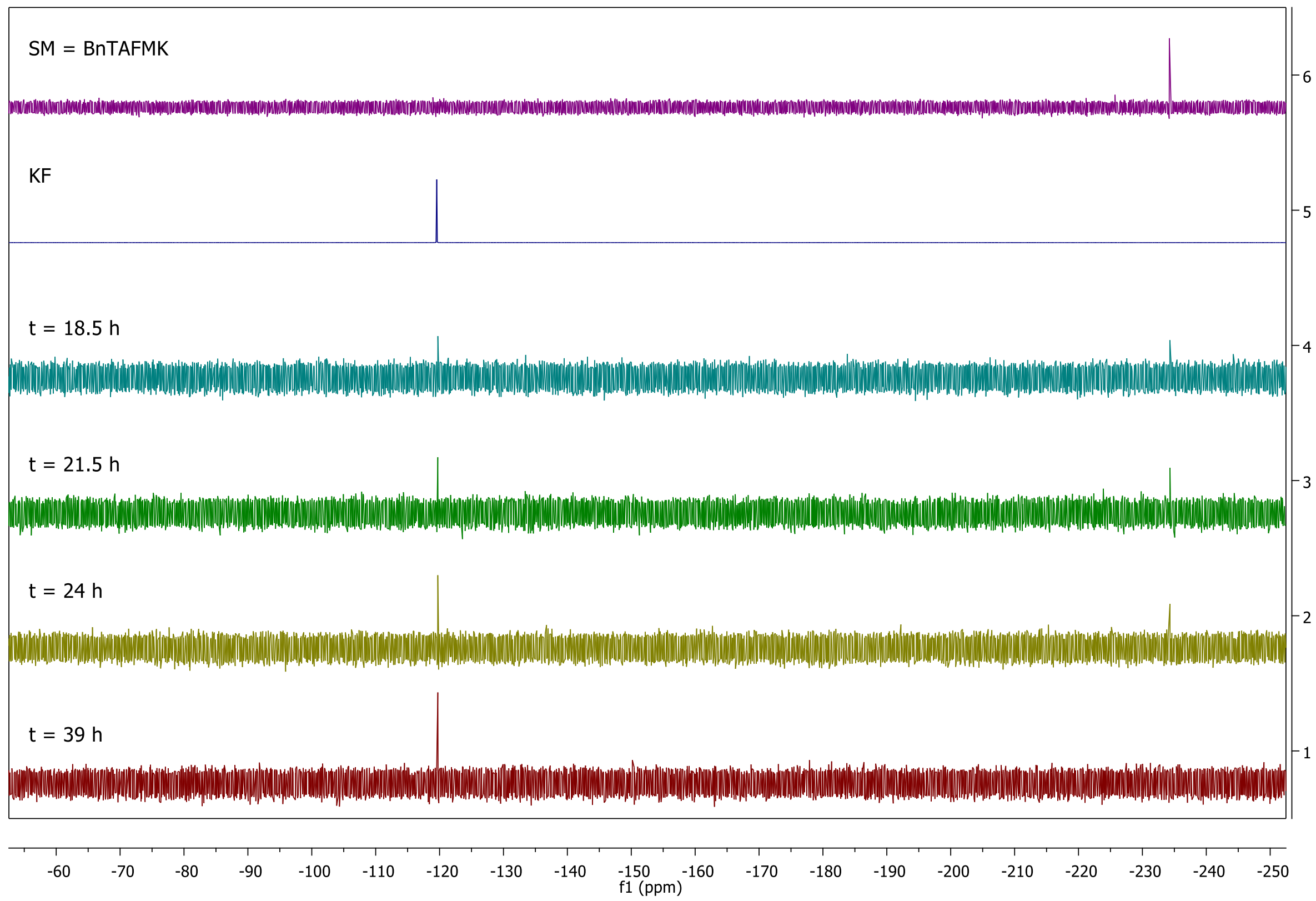

Sample 7)

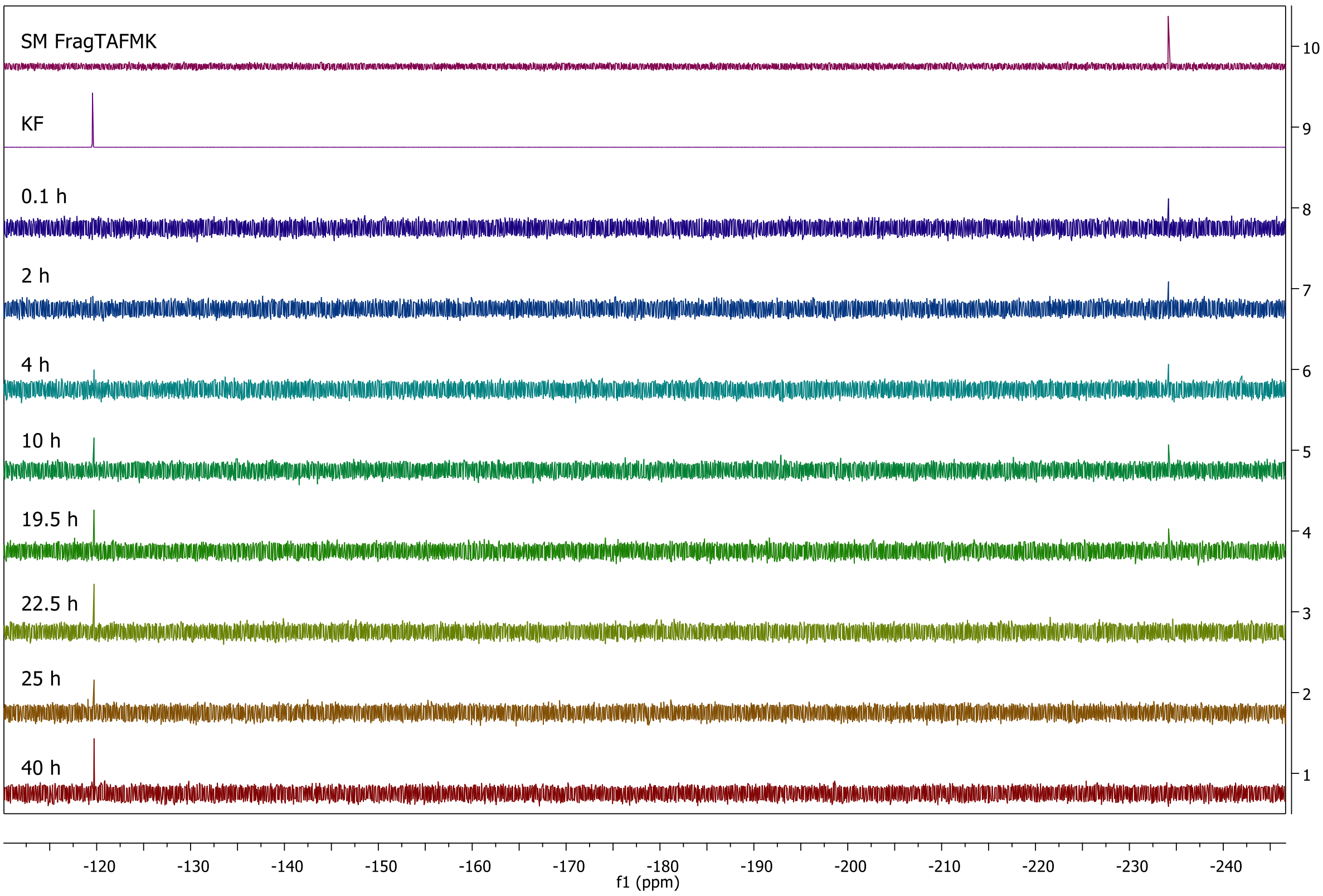

6. LCMS monitoring of protein species

Samples were analyzed on a Shimadzu LCMS-20 system with an SGE ProteoCol C8 HQ1003 150 mm X 2.0 mm ID 3 µm 1000 Å column or SGE ProteoCol C18 HQ303 150 mm X 2.0 mm ID 3 µm 300 Å column monitoring at 214 nm. Buffer A: 99.9 % water, 0.1 % formic acid; buffer B: 99.9 % acetonitrile, 0.1 % formic acid. A gradient of 0-60 % buffer B over 16 mins followed by 1 min isocratic 60 % buffer B, 1 min gradient to 100% buffer A and 2 min of isocratic 100% buffer A at a flow rate of 0.2 mL/min was used as an elution profile. Data were analyzed using LabSolutions v5.31 and mMass v5.0.1. Samples of 20 µM *Ec*DsbA, 1000 µM AFMK **97** in 125 mM citrate-phosphate buffer (at pH 5.11, 6.54 and 8.12), 2 mM TCEP, 41-360 mM NaCl, 2 % DMSO were allowed to react at 25 °C for 7 days. Samples were taken periodically and directly injected and the ratio of apo, mono-adduct and di-adduct determined.

7. Modelling of reaction kinetics

Reactions were modelled according to the scheme below,

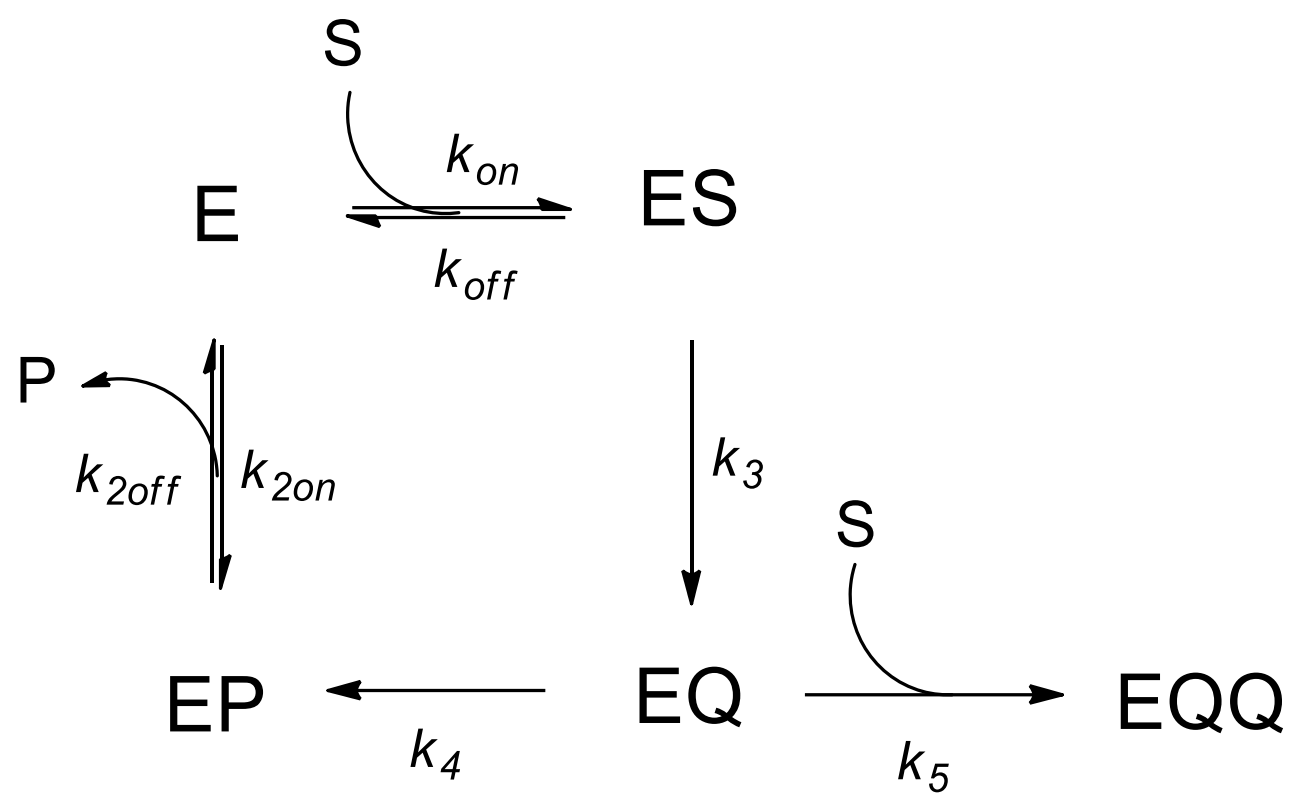

where: E = *Ec*DsbA, S = AFMK **97**, ES = non-covalent complex of *Ec*DsbA and AFMK **97**, EQ = Cys30 covalent complex of *Ec*DsbA and AFMK **97**, P = desfluoro product **99**, EQQ = Di-adduct of AFMK **97** with Cys30 and Cys33, ER = Cys33 covalent complex of *Ec*DsbA and AFMK **97**. Total Apo *Ec*DsbA was calculated from E + ES, total mono-adduct was calculated from EQ + ER and di-adduct was calculated from EQQ. The following constants were then used to model the reactions at different pH values and numerically integrated with a Δ t of 10 seconds and starting E = 0.00002 M and S = 0.001 M to give the modelled reaction kinetics with the respective RMSD (% of species).

**pH**	**8.12**	**6.54**	**5.11**	**Units**
k_{on}	1.83×10^5	1.83×10^5	1.83×10^5	$M^{-1}h^{-1}$
k_{off}	200	394	200	h^{-1}
k_{2on}	8.76×10^4	8.76×10^4	8.76×10^4	$M^{-1}h^{-1}$
k_{2off}	20	10	20	h^{-1}
k_3	0.9627	0.3895	0.0365	h^{-1}
k_4	0.7666	0.1524	0.0194	h^{-1}
k_5	2.8177	0.3878	0	$M^{-1}h^{-1}$
RMSD	2.83	2.01	2.30	% of species

8.General synthetic procedures

Suzuki coupling general procedure A:

Thiophene-3-boronic acid (2.00 g, 15.6 mmol, 1.125 eq.), 3-bromobenzaldehyde (1.62 mL, 13.9 mmol, 1 eq.) and K_3PO_4 (3.32 g, 15.6 mmol, 1.125 eq.) were added to DMF (10 mL) and the resulting mixture was degassed for 10 mins. Palladium *tetrakis*(triphenylphosphine) (401 mg, 0.347 mmol, 2.5 mol%) was then added and the solution placed under an atmosphere of nitrogen. The reaction was heated to 110 °C for 16 hours. The solution was cooled and diluted with water (20 mL) and extracted with diethyl ether (4 × 15 mL). The combined organic layers were dried over anhydrous magnesium sulfate, filtered, and evaporated to dryness to give the crude product.

Reductive amination general procedure B:

4-(Thiophen-3-yl)benzaldehyde (**S3**) (50 mg, 0.27 mmol, 1 eq.) and ethylamine (133 μL, 2 M in THF, 0.266 mmol, 1 eq.) were added to acetonitrile (2 mL) and stirred for 10 mins. Sodium triacetoxyborohydride (79 mg, 0.37 mmol, 1.4 eq.) was then added and the solution left stirring under a nitrogen atmosphere for 16 hours. The solution was then diluted with NaOH (aq., 1 M, 10 mL) and extracted with ethyl acetate (3 × 10 mL). The combined organic extracts were dried over anhydrous magnesium sulfate, filtered, and evaporated to give the crude product as an oil.

Reductive amination general procedure C:

4-(Thiophen-3-yl)benzaldehyde (**S3**) (200 mg, 1.06 mmol, 1 eq.) and hydroxylamine hydrochloride (148 mg, 2.13 mmol, 2 eq.) were added to methanol : water (5:1, 6 mL) and heated to reflux for 50 mins. The reaction was then cooled and evaporated to dryness and the residue was taken up in HCl (aq., 1 M, 20 mL) and extracted with ethyl acetate (3 × 20 mL). The combined organic layers were washed with saturated $NaHCO_3$ (1 × 20 mL) and brine (1 × 20 mL), dried over anhydrous magnesium sulfate, filtered, and evaporated to give 4-(thiophen-3-yl)benzaldehyde oxime (197 mg) as confirmed by MS.

4-(Thiophen-3-yl)benzaldehyde oxime (197 mg, 0.967 mmol, 1 eq.) and lithium aluminium hydride (73 mg, 1.9 mmol, 2 eq.) were added to diethyl ether (6 mL) and heated to reflux for 2.5 hours. The reaction was then cooled and diluted with NaOH (aq., 1 M, 20 mL) and extracted with ethyl acetate (3 × 20 mL). The combined organic layers were dried over anhydrous magnesium sulfate, filtered, and evaporated to give the crude product.

Suzuki coupling general procedure D:

3-Bromo-5-formyl-pyridine (150 mg, 0.806 mmol, 1 eq.), thiophene-3-boronic acid (116 mg, 0.907 mmol, 1.125 eq.) and K_3PO_4 (193 mg, 0.907 mmol, 1.125 eq.) were dissolved in dry DMF (6 mL) and degassed for 10 mins. Palladium *tetrakis*(triphenylphosphine) (47 mg, 0.04 mmol, 5 mol%) was then added and the reaction sealed under nitrogen and heated to 110 °C for 16 hours. The reaction was then cooled and diluted with NaOH (aq., 1 M, 20 mL) and extracted with ethyl acetate (3 × 15 mL). The combined organic layers were dried over anhydrous magnesium sulfate, filtered, and evaporated to give an oil.

Amidation general procedure E:

3-Iodobenzoic acid (407 mg, 1.64 mmol, 1 eq.) was added to thionyl chloride (4 mL). The solution was heated to 65 °C for 2 hours. The reaction was then cooled and evaporated to dryness and redissolved in toluene and evaporated to dryness (2 × 10 mL) to give a white solid. The residue was dissolved in chloroform and ammonium hydrogen carbonate (260 mg, 3.29 mmol, 2 eq. in water, 2 mL) and triethylamine (687 µL, 4.93 mmol, 3 eq.) were added sequentially and the reaction stirred at room temperature for 16 hours. The reaction was diluted with HCl (aq., 1M, 15 mL) and extracted with ethyl acetate (3 × 15 mL). The combined organic layers were washed with saturated $NaHCO_3$, dried over anhydrous magnesium sulfate, filtered, and evaporated to give an oil.

9.Synthesis of compounds S1-S26

3-(Thiophen-3-yl)benzaldehyde (S1)

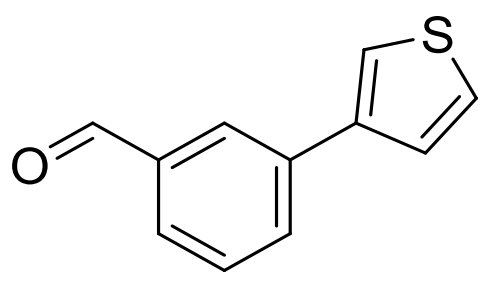

Prepared according to general procedure A with thiophene-3-boronic acid (2.00 g, 15.6 mmol, 1.125 eq.), 3-bromobenzaldehyde (1.62 mL, 13.9 mmol, 1 eq.), K_3PO_4 (3.32 g, 15.6 mmol, 1.125 eq.) and palladium *tetrakis*(triphenylphosphine) (401 mg, 0.347 mmol, 2.5 mol%). Purification by column chromatography using a stepped gradient from hexane to 80:20 hexane : ethyl acetate gave the title compound as a yellow solid (2.51 g, 96 %). The analytical data agreed with literature values.[7]

^{1}H NMR (600 MHz, $CDCl_3$) δ 10.07 (s, 1H), 8.10 (s, 1H), 7.86 (d, J = 7.6 Hz, 1H), 7.80 (d, J = 7.5 Hz, 1H), 7.60 – 7.52 (m, 2H), 7.47 – 7.38 (m, 2H). ^{13}C NMR (150 MHz, $CDCl_3$) δ 192.4, 140.9, 137.0, 136.8, 132.4, 129.6, 128.7, 127.3, 126.9, 126.2, 121.5. RP-HPLC R_t = 10.02 min, 98 %.

3-Bromo-*N*-methylbenzylamine (S2)

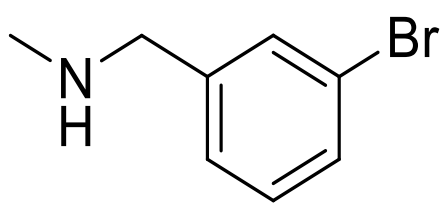

Prepared according to general procedure B with 3-bromobenzaldehyde (100 mg, 0.540 mmol, 1 eq.), methylamine hydrochloride (55 mg, 0.811 mmol, 1.5 eq.) and sodium triacetoxyborohydride (173 mg, 0.811 mmol, 1.5 eq.). Purification by column chromatography using a stepped gradient from 50:50:1 hexane : ethyl acetate : triethylamine to 90:10:1 ethyl acetate : methanol : triethylamine in gave the title compound as a clear oil (29 mg, 27 %). The analytical data agreed with authentic sample purchased from Aldrich.

^{1}H NMR (400 MHz, $CDCl_3$) δ 7.48 (at, *J* = 1.5 Hz, 1H), 7.39-7.36 (m, 1H), 7.25-7.22 (m, 1H), 7.18 (at, *J* = 7.7 Hz, 1H), 3.71 (s, 2H), 2.44 (s, 3H). ESI-MS *m/z* = 169.0 (100 %, M-$CH_3NH)^+$; 200.0 (19 %, $M+H)^+$. RP-HPLC R_t= 3.79 min, 92 %.

4-(Thiophen-3-yl)benzaldehyde (S3)

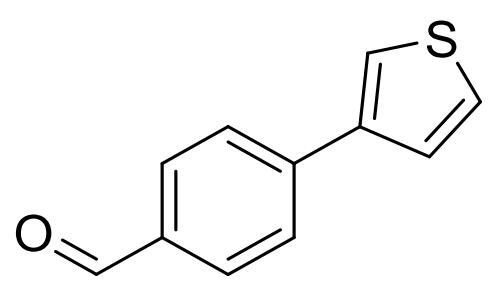

Prepared according to general procedure A with thiophene-3-boronic acid (500 mg, 3.91 mmol, 1.125 eq.), 4-bromobenzaldehyde (943 mg, 3.47 mmol, 1 eq.), K_3PO_4 (830 mg, 3.91 mmol, 1.125 eq.) and palladium tetrakis(triphenylphosphine) (201 mg, 0.174 mmol, 5 mol%). Purification by column chromatography using a stepped gradient from hexane to 80:20 hexane : ethyl acetate gave the title compound as a white solid (487 mg, 75 %). The analytical data agreed with literature values.[6]

^{1}H NMR (300 MHz, $CDCl_3$) δ 10.02 (s, 1H), 7.98 – 7.83 (m, 2H), 7.83 – 7.68 (m, 2H), 7.62 (dd, *J* = 2.5, 1.8 Hz, 1H), 7.45 (dd, *J* = 2.2, 0.7 Hz, 2H). ESI-MS *m/z* = 189.1 $(M+H)^+$. RP-HPLC R_t= 10.80 min, >99 %.

Methyl 2-(3-(thiophen-3-yl)benzylamino)ethanoate (S4)

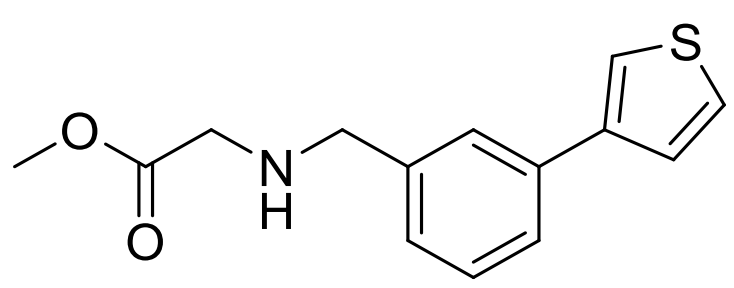

Prepared according to general procedure B with 3-(thiophen-3-yl)benzaldehyde (**S1**) (150 mg, 0.798 mmol, 1 eq.), glycine methyl ester (140 mg, 1.12 mmol, 1.4 eq.) and sodium triacetoxyborohydride (237 mg, 1.12 mmol, 1.4 eq.). Purification by column chromatography using a stepped gradient from 90:10:1 hexane : ethyl acetate : triethylamine to 50:50:1 hexane : ethyl acetate : triethylamine gave the title compound as a clear oil (135 mg, 65 %).

^1H NMR (400 MHz, $CDCl_3$) δ 7.59 – 7.56 (m, 1H), 7.50 (adt, J = 7.8, 1.4 Hz, 1H), 7.46 (dd, J = 2.8, 1.5 Hz, 1H), 7.42 – 7.34 (m, 3H), 7.28 – 7.24 (m, 1H), 3.85 (s, 2H), 3.73 (s, 3H), 3.45 (s, 2H). ^{13}C NMR (100 MHz, $CDCl_3$) δ 173.0, 142.3, 140.2, 136.2, 129.1, 127.2, 126.5, 126.5, 126.3, 125.5, 120.5, 53.4, 51.9, 50.1. ESI-MS *m/z* = 262.1 (20 %, M+H)$^+$, 173.0 (100 %, M-$CH_3O_2CCH_2HN$)$^+$. ESI-HRMS-TOF found 262.0909, calculated 262.0896 for (M+H)$^+$. RP-HPLC R_t= 6.75 min, 95 %.

Methyl 3-aminopropanoate hydrochloride (S5)

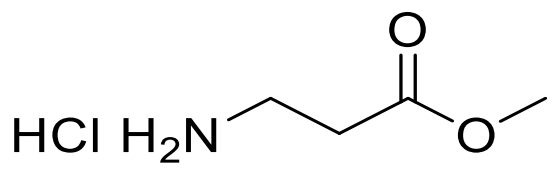

Was prepared according to the procedure of Gros *et al.*[8] and analytical data were concordant with literature values. [8]

Methyl 4-aminobutanoate hydrochloride (S6)

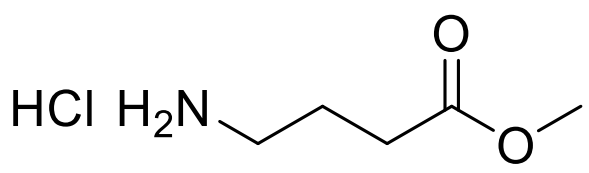

Was prepared according to the procedure of Gros *et al.*[8] and analytical data were concordant with literature values. [8]

Methyl 5-aminopentanoate hydrochloride (S7)

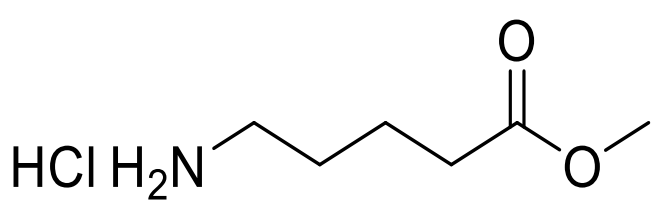

Was prepared according to the procedure of Gros *et al.*[8] and analytical data were concordant with literature values. [8]

Methyl 6-aminohexanoate hydrochloride (S8)

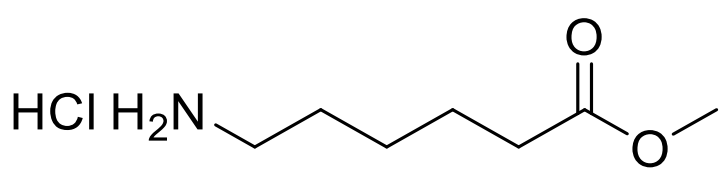

Was prepared according to the procedure of Gros *et al.*[8] and analytical data were concordant with literature values. [8]

***N*-Boc-*β*-alanine (S9)**

β-Alanine (1.00 g, 11.2 mmol, 1 eq.), sodium hydroxide (494 mg, 12.4 mmol, 1.1 eq.) and di-*tert*-butyl dicarbonate (2.69 g, 12.4 mmol, 1.1 eq.) were added to 1:1 *t*-BuOH:H_2O and the resulting mixture was stirred at room temp for 16 hours. The reaction was then diluted with HCl (aq., 1 M, 20 mL) and extracted with ethyl acetate (3 × 20 mL). The combined organic layers were dried over anhydrous magnesium sulfate, filtered, and evaporated to give *N*-Boc-*β*-alanine (**S9**) as a white solid (1.83 g, 86 %).

^{1}H NMR (400 MHz, $CDCl_3$) δ 5.11 (bs, 1H), 3.38 (bs, 2H), 2.56 (bs, 2H), 1.43 (s, 9H).

***N*-Boc-2-aminopropanamide (S10)**

N-Boc-*β*-alanine (751 mg, 3.97 mmol, 1 eq.) and triethylamine (553 µL, 3.97 mmol, 1 eq.) were added to dry THF (10 mL) and the reaction mixture was cooled to -10 °C under nitrogen. Ethyl chloroformate (380 µL, 3.97 mmol, 1 eq.) was then added dropwise. The reaction was stirred at -10 °C under nitrogen for a further 30 mins followed by slow addition of ammonium carbonate (784 mg, 9.92 mmol, 2.5 eq.) in H_2O (3 mL). The reaction was allowed to warm to room temperature and left stirring under nitrogen for a further 16 hours. The reaction mixture was then evaporated and taken up in H_2O (20 mL) and this solution was extracted with ethyl acetate (3 × 20 mL). The combined organic layers were dried over anhydrous magnesium sulfate, filtered, and evaporated to give *N*-Boc-2-aminopropanamide (**S10**) as a white solid (524 mg, 70 %).

^{1}H NMR (400 MHz, $CDCl_3$) δ 5.77 (bs, 1H), 5.52 (bs, 1H), 5.14 (bs, 1H), 3.41 (aq, *J* = 6.2 Hz, 2H), 2.45 (t, *J* = 6.0 Hz, 2H), 1.43 (s, 9H).

3-Aminopropanamide hydrochloride (S11)

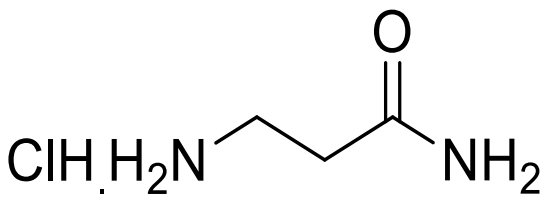
Was prepared according to procedures of McCubbin *et al.*[9] and Schneider *et al.*[10] and gave analytical data consistent with previous reports.[10-14]

N-Boc-2-aminopropanamide (152 mg, 0.797 mmol, 1 eq.) was added to a solution of HCl (2M in Et_2O, 1.20 mL, 2.39 mmol, 3 eq.) in methanol (5 mL) and stirred at room temperature for 1 hour then evaporated to dryness to give 3-aminopropanamide hydrochloride (**S11**) (98 mg, 98 %).

^{1}H NMR (400 MHz, CD_3OD) δ 3.18 (t, *J* = 6.3 Hz, 2H), 2.64 (t, *J* = 6.4 Hz, 2H). ^{13}C NMR (400 MHz, CD_3OD) δ 174.8, 37.0, 32.2.

5-(Thiophen-3-yl)nicotinaldehyde (S12)

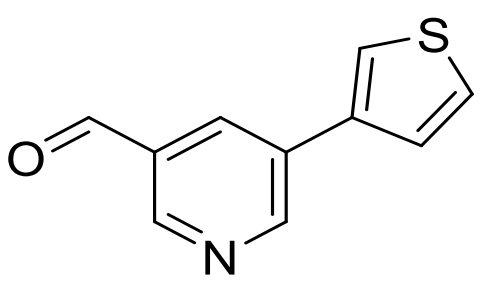
Prepared according to general procedure D with 3-bromo-5-formyl-pyridine (150 mg, 0.806 mmol, 1 eq.), thiophene-3-boronic acid (116 mg, 0.907 mmol, 1.125 eq.), K_3PO_4 (193 mg, 0.907 mmol, 1.125 eq.) and palladium *tetrakis*(triphenylphosphine) (47 mg, 0.04 mmol, 5 mol%). Purification by column chromatography using a stepped gradient from hexane to 70:30 hexane : ethyl acetate gave the title compound as a light yellow solid (49 mg, 32 %).

^{1}H NMR (400 MHz, $CDCl_3$) δ 10.16 (s, 1H), 9.09 (d, *J* = 2.3 Hz, 1H), 8.97 (d, *J* = 1.9 Hz, 1H), 8.31 (at, *J* = 2.1 Hz, 1H), 7.63 (dd, *J* = 2.9, 1.4 Hz, 1H), 7.49 (dd, *J* = 5.0, 2.9 Hz, 1H), 7.44 (dd, *J* = 5.0, 1.4 Hz, 1H). ^{13}C NMR (100 MHz, $CDCl_3$) δ 190.9, 152.7, 150.6, 137.4, 132.7, 132.3, 131.5, 127.7, 125.8, 122.8. ESI-MS *m/z* = 190.1 $(M+H)^+$. ESI-HRMS-TOF found 190.0312, calculated 190.0321 for $(M+H)^+$. RP-HPLC R_t= 4.27 min, 99 %.

6-(Thiophen-3-yl)picolinaldehyde (S13)

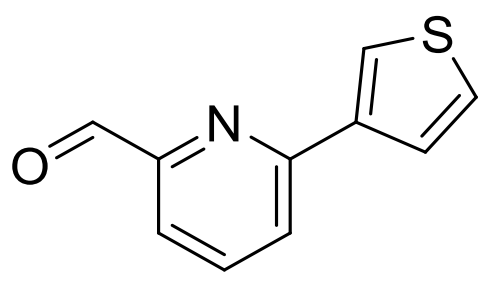

Prepared according to general procedure D with 2-bromo-6-formyl-pyridine (151 mg, 0.806 mmol, 1 eq.), thiophene-3-boronic acid (116 mg, 0.907 mmol, 1.125 eq.), K_3PO_4 (193 mg, 0.907 mmol, 1.125 eq.) and palladium *tetrakis*(triphenylphosphine) (47 mg, 0.04 mmol, 5 mol%). Purification by column chromatography using a stepped gradient from hexane to 70:30 hexane : ethyl acetate gave the title compound as a white solid (81 mg, 53 %).

^{1}H NMR (400 MHz, $CDCl_3$) δ 10.10 (d, *J* = 0.7 Hz, 1H), 8.00 (dd, *J* = 3.0, 1.3 Hz, 1H), 7.87 – 7.77 (m, 3H), 7.73 (dd, *J* = 5.1, 1.3 Hz, 1H), 7.42 (dd, *J* = 5.1, 3.0 Hz, 1H). ^{13}C NMR (100 MHz, $CDCl_3$) δ 194.0, 154.0, 152.7, 141.1, 137.8, 126.8, 126.2, 124.6, 124.2, 119.5. ESI-MS *m/z* = 190.1 $(M+H)^+$. ESI-HRMS-TOF found 190.0317, calculated 190.0321 for $(M+H)^+$. RP-HPLC R_t= 5.51 min, 96 %.

6-(Thiophen-3-yl)nicotinaldehyde (S14)

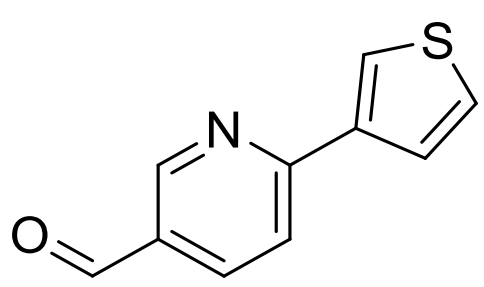

Prepared according to general procedure D with 2-bromo-5-formyl-pyridine (150 mg, 0.806 mmol, 1 eq.), thiophene-3-boronic acid (116 mg, 0.907 mmol, 1.125 eq.), K_3PO_4 (193 mg, 0.907 mmol, 1.125 eq.) and palladium *tetrakis*(triphenylphosphine) (47 mg, 0.04 mmol, 5 mol%). Purification by column chromatography using a stepped gradient from hexane to 70:30 hexane : ethyl acetate gave the title compound as a white solid (39 mg, 25 %).

^{1}H NMR (400 MHz, $CDCl_3$) δ 10.07 (s, 1H), 9.03 (dd, *J* = 2.1, 0.6 Hz, 1H), 8.15 (dd, *J* = 8.2, 2.2 Hz, 1H), 8.08 (dd, *J* = 3.0, 1.3 Hz, 1H), 7.74 (d, *J* = 8.2 Hz, 1H), 7.71 (dd, *J* = 5.1, 1.3 Hz,

1H), 7.42 (dd, *J* = 5.1, 3.0 Hz, 1H). ^{13}C NMR (100 MHz, $CDCl_3$) δ 190.4, 158.0, 152.7, 141.2, 136.6, 129.6, 127.4, 126.6, 126.4, 120.4. ESI-MS *m/z* = 190.1 $(M+H)^+$. ESI-HRMS-TOF found 190.0323, calculated 190.0321 for $(M+H)^+$. RP-HPLC R_t= 4.52 min, >99 %.

5-(Thiophen-3-yl)picolinaldehyde (S15)

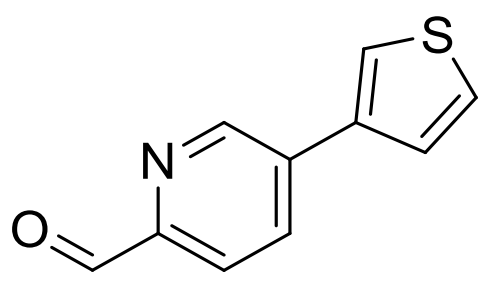

Prepared according to general procedure D with 5-bromo-2-formyl-pyridine (151 mg, 0.806 mmol, 1 eq.), thiophene-3-boronic acid (116 mg, 0.907 mmol, 1.125 eq.), K_3PO_4 (193 mg, 0.907 mmol, 1.125 eq.) and palladium *tetrakis*(triphenylphosphine) (47 mg, 0.04 mmol, 5 mol%). Purification by column chromatography using a stepped gradient from hexane to 70:30 hexane : ethyl acetate gave the title compound as a white solid (69 mg, 44 %).

^{1}H NMR (400 MHz, $CDCl_3$) δ 10.06 (d, *J* = 0.7 Hz, 1H), 9.00 (dd, *J* = 2.0, 0.8 Hz, 1H), 8.01 (ddd, *J* = 8.1, 2.1, 0.6 Hz, 1H), 7.96 (dd, *J* = 8.1, 0.8 Hz, 1H), 7.66 (dd, *J* = 2.9, 1.4 Hz, 1H), 7.47 (dd, *J* = 5.1, 2.9 Hz, 1H), 7.44 (dd, *J* = 5.1, 1.4 Hz, 1H). ^{13}C NMR (100 MHz, $CDCl_3$) δ 192.9, 151.3, 148.0, 137.7, 135.4, 134.1, 127.7, 125.9, 123.6, 122.1. ESI-MS *m/z* = 190.1 $(M+H)^+$. ESI-HRMS-TOF found 190.0316, calculated for 190.0321 $(M+H)^+$. RP-HPLC R_t= 4.61 min, > 99 %.

2-(Thiophene-3-yl)isonicotinaldehyde (S16)

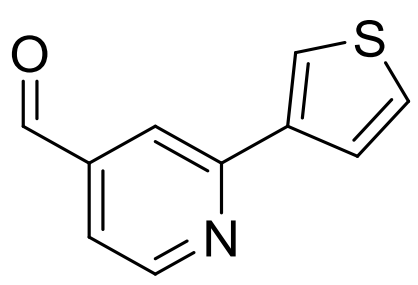

Prepared according to general procedure D with 2-bromo-4-formyl-pyridine (150 mg, 0.806 mmol, 1 eq.), thiophene-3-boronic acid (116 mg, 0.907 mmol, 1.125 eq.), K_3PO_4 (193 mg, 0.907 mmol, 1.125 eq.) and palladium *tetrakis*(triphenylphosphine) (47 mg,

0.04 mmol, 5 mol%). Purification by column chromatography using a stepped gradient from hexane to 70:30 hexane : ethyl acetate gave the title compound as a clear oil (94 mg, 62 %).

^{1}H NMR (400 MHz, $CDCl_3$) δ 10.10 (s, 1H), 8.85 (dd, *J* = 4.9, 0.7 Hz, 1H), 8.03 – 7.95 (m, 2H), 7.71 (dd, *J* = 5.1, 1.3 Hz, 1H), 7.56 (dd, *J* = 4.9, 1.4 Hz, 1H), 7.43 (dd, *J* = 5.1, 3.0 Hz, 1H). ^{13}C NMR (100 MHz, $CDCl_3$) δ 191.7, 155.2, 151.2, 142.5, 141.3, 126.9, 126.2, 124.8, 120.4, 118.6. RP-HPLC R_t= 6.38 min, 96 %.

2-Formyl-4-phenylthiophene (S17)

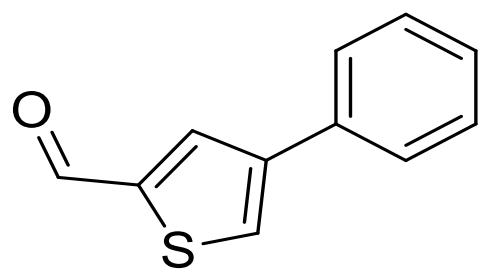

Prepared according to general procedure A with 2-formylthiophene-4-boronic acid (168 mg, 1.08 mmol, 1.125 eq.), bromobenzene (101 µL, 0.955 mmol, 1 eq.), K_3PO_4 (228 mg, 1.08 mmol, 1.125 eq.) and palladium *tetrakis*(triphenylphosphine) (55 mg, 0.05 mmol, 5 mol%). Purification by column chromatography using a stepped gradient from hexane to 92:8 hexane : ethyl acetate gave the title compound as a light red solid (74 mg, 41 %). The analytical data agreed with literature values.[15]

^{1}H NMR (400 MHz, $CDCl_3$) δ 9.98 (d, *J* = 1.3 Hz, 1H), 8.04 (d, *J* = 1.5 Hz, 1H), 7.86 (at, *J* = 1.4 Hz, 1H), 7.63 – 7.56 (m, 2H), 7.48 – 7.41 (m, 2H), 7.40 – 7.33 (m, 1H). ^{13}C NMR (100 MHz, $CDCl_3$) δ 183.1, 144.6, 143.8, 134.9, 134.6, 129.8, 129.3, 128.2, 126.5. ESI-MS *m/z* = 189.1 $(M+H)^+$. RP-HPLC R_t= 9.49 min, >99 %.

2-Formyl-5-phenylthiophene (S18)

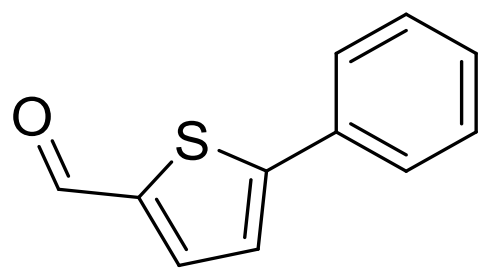

Prepared according to general procedure A with 2-formylthiophene-5-boronic acid (168 mg, 1.08 mmol, 1.125 eq.), bromobenzene (101 µL, 0.955 mmol, 1 eq.),

K_3PO_4 (228 mg, 1.08 mmol, 1.125 eq.) and palladium *tetrakis*(triphenylphosphine) (55 mg, 0.05 mmol, 5 mol%). Purification by column chromatography using a stepped gradient from hexane to 92:8 hexane : ethyl acetate gave the title compound as a yellow solid (63 mg, 35 %). The analytical data agreed with literature values.[15]

^{1}H NMR (400 MHz, $CDCl_3$) δ 9.90 (s, 1H), 7.74 (d, *J* = 3.9 Hz, 1H), 7.71 – 7.64 (m, 2H), 7.48 – 7.35 (m, 4H). ^{13}C NMR (100 MHz, $CDCl_3$) δ 183.0, 154.5, 142.6, 137.5, 133.2, 129.6, 129.4, 126.6, 124.3. ESI-MS *m/z* = 189.1 $(M+H)^+$. RP-HPLC R_t= 10.02 min, 99 %.

3-Iodobenzamide (S19)

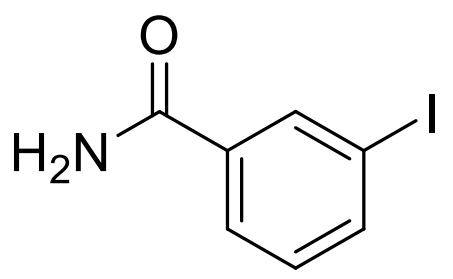

Prepared according to general procedure E with 3-iodobenzoic acid (407 mg, 1.64 mmol, 1 eq.), thionyl chloride (4 mL), ammonium hydrogen carbonate (260 mg, 3.29 mmol, 2 eq. in water, 2 mL) and triethylamine (687 μL, 4.93 mmol, 3 eq.). Purification by column chromatography using a stepped gradient from 70:30 hexane : ethyl acetate to 70:30 ethyl acetate : hexane to give the title compound as a white solid (79 mg, 19 %). ^{1}H NMR was consistent with rotamers of the amide. The analytical data agreed with literature values.[17]

^{1}H NMR (400 MHz, $CDCl_3$) δ 8.44 (s, 0.1H), 8.16 (at, *J* = 1.6 Hz, 0.8H), 8.07 – 8.03 (m, 0.1H), 7.91 (ddd, *J* = 7.9, 1.8, 1.1 Hz, 0.1H), 7.87 (ddd, *J* = 7.9, 1.7, 1.1 Hz, 0.9H), 7.76 (ddd, *J* = 7.8, 1.7, 1.1 Hz, 0.9H), 7.20 (m, 1H), 5.90 (bs, 2H). ESI-MS *m/z* = 247.9 $(M+H)^+$. RP-HPLC R_t= 7.10 min, 97 %.

***N*-Methyl-3-iodobenzamide (S20)**

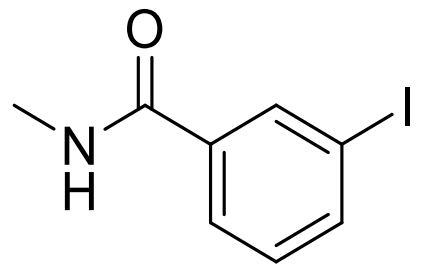
Prepared according to general procedure E with 3-iodobenzoic acid (403 mg, 1.62 mmol, 1 eq.) thionyl chloride (4 mL), methylamine hydrochloride (164 mg, 2.43 mmol, 1.5 eq.) and triethylamine (679 µL, 4.87 mmol, 3 eq.). Purification by column chromatography using a stepped gradient from 70:30 hexane : ethyl acetate to 70:30 ethyl acetate: hexane to give the title compound as a white solid (280 mg, 66 %). The analytical data agreed with literature values.[16]

^{1}H NMR (400 MHz, $CDCl_3$) δ 8.09 (at, *J* = 1.7 Hz, 1H), 7.80 (ddd, *J* = 7.9, 1.7, 1.1 Hz, 1H), 7.71 (ddd, *J* = 7.8, 1.7, 1.1 Hz, 1H), 7.15 (at, *J* = 7.8 Hz, 1H), 6.26 (bs, 1H), 2.99 (ad, *J* = 4.9 Hz, 3H). ^{13}C NMR (100 MHz, $CDCl_3$) δ 166.8, 140.4, 136.8, 136.1, 130.4, 126.2, 94.4, 27.1. ESI-MS *m/z* = 262.0 $(M+H)^+$. RP-HPLC R_t= 7.50 min, 99 %.

_N_-Cyclohexyl-3-iodobenzamide (S21)

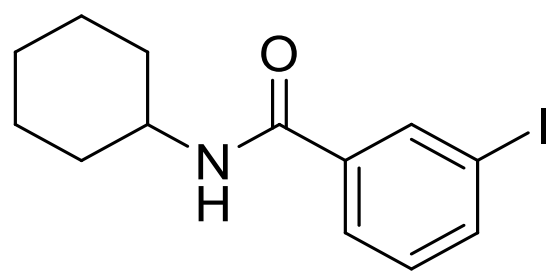
Prepared according to general procedure E with 3-iodobenzoic acid (416 mg, 1.68 mmol, 1 eq.), thionyl chloride (4 mL), cyclohexylamine (288 µL, 2.52 mmol, 1.5 eq.) and triethylamine (702 µL, 5.04 mmol, 3 eq.). Purification by column chromatography using a stepped gradient from hexane to 75:25 hexane : ethyl acetate to give the title compound as a white solid (234 mg, 42 %).

^{1}H NMR (400 MHz, $CDCl_3$) δ 8.06 (at, *J* = 1.7 Hz, 1H), 7.80 (ddd, *J* = 7.9, 1.7, 1.1 Hz, 1H), 7.70 (ddd, *J* = 7.8, 1.5, 1.1 Hz, 1H), 7.16 (at, *J* = 7.8 Hz, 1H), 5.92 (bs, 1H), 3.95 (ttd, *J* = 11.9, 8.0, 3.9 Hz, 1H), 2.07 – 1.97 (m, 2H), 1.81 – 1.71 (m, 2H), 1.70 – 1.63 (m, 1H), 1.49 – 1.35 (m, 2H), 1.30 – 1.17 (m, 3H). ^{13}C NMR (100 MHz, $CDCl_3$) δ 165.2, 140.3, 137.3, 136.0, 130.4,

126.2, 94.4, 49.1, 33.3, 25.7, 25.0. ESI-MS *m/z* = 330.0 $(M+H)^+$. ESI-HRMS-TOF found 330.0365, calculated 330.0349 for $(M+H)^+$. RP-HPLC R_t= 10.60 min, 99%.

Trans-4-(aminomethyl)cyclohexaneamide hydrochloride (S22)

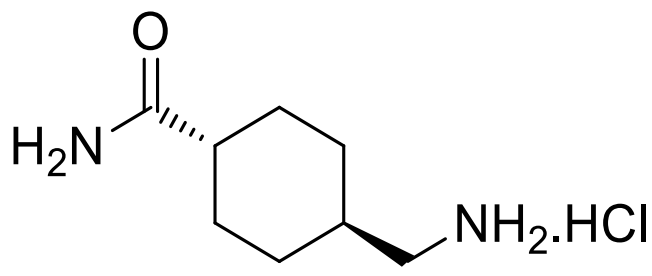

Prepared according to the procedures of McCubbin et al. [9] and Schneider et al. [10] and characterisation was in accordance with Yen *et al.*[18] and Li *et al.*[19]

Trans-(4-aminomethyl)cyclohexane carboxylic acid (2.00 g, 12.7 mmol, 1 eq.) and sodium hydroxide (560 mg, 14.0 mmol, 1.1 eq.) was added to *tert*-butanol : water (20 mL, 2:1) followed by di-*tert*-butyl dicarbonate (3.05 g, 14.0 mmol, 1.1 eq.). The reaction was stirred at room temperature for 18 hours, then diluted with HCl (aq., 1 M, 20 mL) and extracted with ethyl acetate (3 × 20 mL). The combined organic layers were dried over anhydrous magnesium sulfate, filtered, and evaporated to give trans-*N*-Boc-4-(aminomethyl)cyclohexanecarboxylic acid as a white solid (2.70 g, 82 %). The analytical data agreed with literature values.[18]

^{1}H NMR (400 MHz, $CDCl_3$) δ 4.59 (bs, 1H), 2.97 (d, *J* = 5.5 Hz, 2H), 2.25 (tt, *J* = 12.2, 3.5 Hz, 1H), 2.08 – 1.99 (m, 2H), 1.83 (dd, *J* = 13.4, 2.7 Hz, 2H), 1.50 – 1.33 (m, 12H), 0.96 (aqd, *J* = 13.1, 3.3 Hz, 2H). ^{13}C NMR (100 MHz, $CDCl_3$) δ 181.5, 156.3, 79.4, 46.7, 43.1, 37.8, 29.7, 28.6, 28.4.

Trans-*N*-Boc-4-(aminomethyl)cyclohexanecarboxylic acid (1.00 g, 3.89 mmol, 1 eq.) and triethylamine (543 µL, 3.89 mmol, 1 eq.) were added to THF (15 ml) and cooled to 0 °C followed by dropwise addition of ethyl chloroformate (372 µL, 3.89 mmol, 1 eq.) over 5 mins. The reaction was stirred under nitrogen at 0 °C for 30 mins, followed by addition of ammonium carbonate (769 mg, 9.72 mmol, 2.5 eq.) in water (3 mL). The reaction was warmed to room

temperature and stirring continued under nitrogen for 16 hours. The reaction was then diluted with NaOH (aq., 1 M, 20 mL) and extracted with ethyl acetate (3 × 20 mL) and the combined organic layers were dried over anhydrous magnesium sulfate, filtered, and evaporated to give trans-(*N*-Boc-4-aminomethyl)cyclohexaneamide as a white solid (836 mg, 84 %). The analytical data agreed with literature values.[18]

^{1}H NMR (400 MHz, CD_3OD) δ 2.91 – 2.85 (m, *J* = 5.7 Hz, 2H), 2.16 (tt, *J* = 12.3, 3.4 Hz, 1H), 1.92 – 1.76 (m, 4H), 1.51 – 1.36 (m, 12H), 1.04 – 0.90 (m, 2H). ^{13}C NMR (100 MHz, CD_3OD) δ 182.1, 158.7, 79.8, 48.4, 47.5, 45.9, 39.1, 31.0, 30.3, 28.8. ESI-MS *m/z* = 156.2 (100 %, M-$C_4H_7O_2$)$^+$; 257.2 (20 %, M+H)$^+$.

Trans-(*N*-Boc-4-Aminomethyl)cyclohexaneamide (700 mg, 2.73 mmol, 1 eq.) was added to methanol (10 mL) to give a white suspension. Ethereal HCl (2 M, 8.12 mmol, 3 eq.) was then added and the reaction stirred at room temperature for 1 hour, then evaporated to dryness to give the title compound **S22** as a white solid (500 mg, 95 %). The analytical data agreed with literature values.[19]

^{1}H NMR (400 MHz, CD_3OD) δ 2.81 (d, *J* = 6.7 Hz, 2H), 2.29 (at, *J* = 12.0 Hz, 1H), 1.93 (at, *J* = 12.5 Hz, 4H), 1.67 (s, 1H), 1.52 (dd, *J* = 23.1, 12.2 Hz, 2H), 1.18 – 1.04 (m, 2H). ^{13}C NMR (100 MHz, CD_3OD) δ 182.1, 46.2, 45.0, 36.7, 30.3, 29.7. ESI-MS *m/z* = 140.2 (100%, M-NH_2)$^+$; 157.2 (10%, M+H)$^+$.

Methyl trans-4-(aminomethyl)cyclohexanecarboxylate (S23)

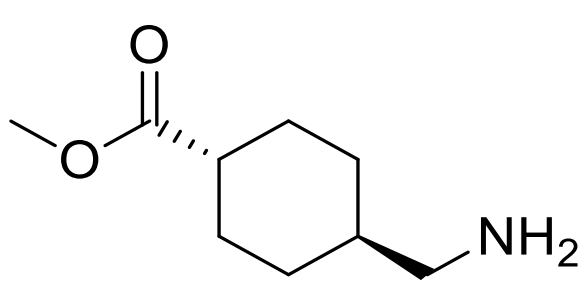

Was prepared according to the procedure of Gros *et al.*[8] and analytical data were concordant with literature values.[20]

Trans-4-(aminomethyl)cyclohexylmethanol (S24)

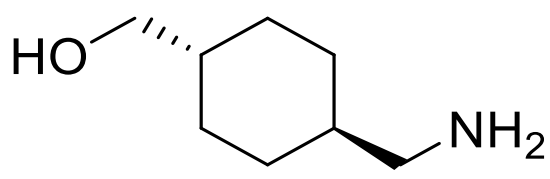
Was prepared according to the procedure of Moffat *et al.*[21] trans-(4-aminomethyl)cyclohexane carboxylic acid (1.00 g, 6.36 mmol, 1 eq.) was added to THF (20 mL) and cooled to 0 °C. Lithium aluminium hydride (724 mg, 19.1 mmol, 3 eq.) was then added and the reaction sealed under nitrogen and stirred at room temperature for 3 hours. The reaction was then cooled to 0 °C and quenched with water (3.0 mL), dried over anhydrous sodium sulfate, filtered, and evaporated to give the title compound as a clear oil (331 mg, 36 %). The analytical data agreed with literature values.[21]

^{1}H NMR (400 MHz, $CDCl_3$) δ 3.42 (d, J = 6.3 Hz, 2H), 2.51 (d, J = 6.5 Hz, 2H), 1.89 – 1.76 (m, 4H), 1.48 – 1.36 (m, 1H), 1.31 – 1.17 (m, 1H), 1.03 – 0.83 (m, 4H). ^{13}C NMR (100 MHz, $CDCl_3$) δ 68.6, 48.9, 41.5, 40.9, 30.3, 29.3. ESI-MS m/z = 144.3 $(M+H)^+$.

Methyl trans-4-(aminomethyl)cyclohexanecarboxylate (S25)

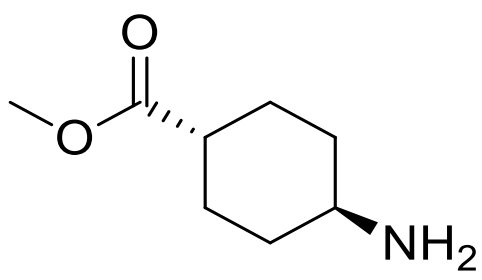
Was prepared according the procedure of Gros *et al.*[8] and analytical data were concordant with literature values.[22]

***N*-(2,2,6,6-Tetramethylpiperidin-4-yl)-3-(thiophen-3-yl)benzylamine (S26)**

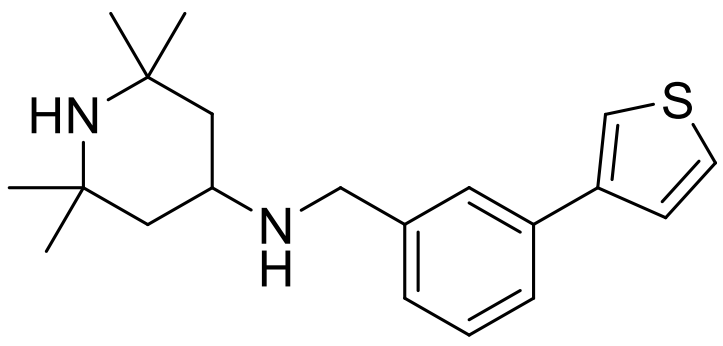
Prepared according to general procedure B with 3-(thiophen-3-yl)benzaldehyde (**S1**) (43 mg, 0.23 mmol, 1 eq.), 2,2,6,6-tetramethyl-4-aminopiperidine (34 µL, 0.32 mmol, 1.4 eq.) and sodium triacetoxyborohydride (67 mg, 0.32 mmol, 1.4 eq.). Purification by column chromatography using a stepped gradient from 50:50:1 ethyl acetate : hexane : triethylamine to 90:10:1 ethyl acetate : methanol : triethylamine gave

the title compound as a clear oil (44 mg, 59 %). ^{15}N-^{1}H HSQC and ^{15}N-^{1}H HMBC NMR confirmed regioselective reductive amination.

^{1}H NMR (400 MHz, $CDCl_3$) δ 7.58 – 7.55 (m, *J* = 1.6 Hz, 1H), 7.49 (adt, *J* = 8.0, 1.6 Hz, 1H), 7.46 (dd, *J* = 2.8, 1.5 Hz, 1H), 7.41 – 7.33 (m, 3H), 7.28 – 7.24 (m, 1H), 3.89 (s, 2H), 3.02 (tt, *J* = 11.6, 3.6 Hz, 1H), 1.92 (add, *J* = 12.7, 3.6 Hz, 2H), 1.19 (s, 6H), 1.14 (s, 6H), 0.93 (at, *J* = 12.0 Hz, 2H). ^{13}C NMR (100 MHz, $CDCl_3$) δ 142.5, 141.4, 136.2, 129.1, 127.1, 126.5, 126.4, 126.3, 125.3, 120.5, 51.2, 51.1, 49.7, 46.4, 35.3, 28.9. ESI-MS *m/z* = 329.2 $(M+H)^+$. ESI-HRMS-TOF found 329.2035, calculated 329.2046 for $(M+H)^+$. RP-HPLC R_t= 5.86 min, 99%.

10.Synthesis of compounds 1 and 18-101

N-Methyl-3-(thiophen-3-yl)benzylamine hydrochloride (1)

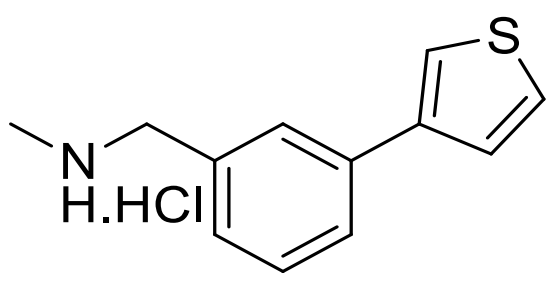

Method A:

3-(Thiophen-3-yl)benzaldehyde (**S1**) (800 mg, 4.25 mmol, 1 eq.) and methylamine hydrochloride (430 mg, 6.37 mmol, 1.5 eq.) were added to acetonitrile (20 mL) and stirred at room temperature for 1 hour followed by addition of sodium triacetoxyborohydride (1.35 g, 6.37 mmol, 1.5 eq.). The reaction was then sealed under nitrogen and stirred at room temperature for 17 hours. The solution was then diluted with NaOH (aq., 1 M, 40 mL) and extracted with ethyl acetate (3 × 40 mL) and the combined organic layers were dried over anhydrous magnesium sulfate, filtered, and evaporated to give the crude product. Purification by column chromatography using a stepped gradient from 100:1 ethyl acetate : triethylamine to 85:15:1 ethyl acetate : methanol : triethylamine gave a clear oil which was evaporated to

dryness then taken up in diethyl ether and precipitated with HCl (4 mL, 2 M in diethyl ether) to give the title compound as an off white solid (274 mg, 27 %).

Method B:

Prepared according to general procedure A with thiophene-3-boronic acid (300 mg, 2.35 mmol, 1.125 eq.), 3-bromo-*N*-methylbenzylamine (**S2**) (285 µL, 2.08 mmol, 1 eq.), K_3PO_4 (498 mg, 2.35 mmol, 1.125 eq.) and palladium *tetrakis*(triphenylphosphine) (120 mg, 0.104 mmol, 5 mol%). Purification by column chromatography using a stepped gradient from 90:10:1 ethyl acetate : hexane : triethylamine to 90:10:1 ethyl acetate : methanol : triethylamine gave a clear oil which was evaporated to dryness then taken up in diethyl ether and precipitated with HCl (2 mL, 2 M in diethyl ether). The solid was then recrystallized from ethanol : hexane (1:4, 25 mL) to give the title compound as an off white solid (177 mg, 35 %).

The analytical data agreed with the previously purchased Maybridge compound.

^{1}H NMR (400 MHz, CD_3OD) δ 7.82 (at, *J* = 1.6 Hz, 1H), 7.78 – 7.74 (m, 1H), 7.72 (dd, *J* = 2.6, 1.7 Hz, 1H), 7.54 – 7.48 (m, 3H), 7.42 – 7.38 (m, 1H), 4.24 (s, 2H), 2.75 (s, 3H). ^{13}C NMR (100 MHz, CD_3OD) δ 142.5, 138.3, 133.2, 130.9, 129.3, 128.7, 128.5, 127.7, 127.0, 122.2, 53.7, 33.2. ESI-MS *m/z* = 173.1 (100 %, M-CH_4N)$^+$; 204.1 (9 %, M+H)$^+$. RP-HPLC R_t= 5.65 min, 99 %.

***N*-Ethyl-4-(thiophen-3-yl)benzylamine (18)**

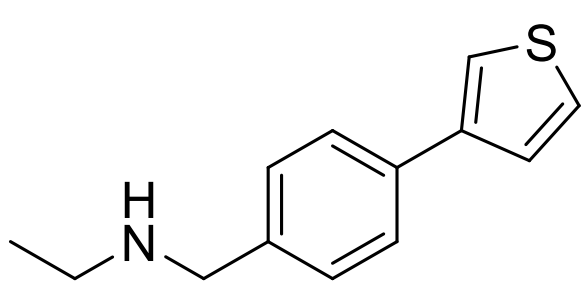

Prepared according to general procedure B with 4-(thiophen-3-yl)benzaldehyde (**S3**) (50 mg, 0.27 mmol, 1 eq.), ethylamine (133 µL, 2 M in THF, 0.266 mmol, 1 eq.) and sodium triacetoxyborohydride (79 mg, 0.37 mmol, 1.4 eq.). Purification by

column chromatography using a stepped gradient from ethyl acetate to 99:1 ethyl acetate : triethylamine gave the title compound as a white solid (22 mg, 38 %).

^{1}H NMR (300 MHz, CD_3OD) δ 7.66 – 7.54 (m, 3H), 7.49 – 7.30 (m, 4H), 3.73 (s, 2H), 2.64 (q, *J* = 7.1 Hz, 2H), 1.13 (t, *J* = 7.1 Hz, 3H). ^{13}C NMR (75 MHz, CD_3OD) δ 143.3, 139.4, 136.1, 130.1, 127.34, 127.27, 127.1, 121.1, 53.9, 44.0, 14.6 ESI-MS *m/z* = 218.4 $(M+H)^+$. ESI-HRMS-TOF found 218.0990, calculated 218.0998 for $(M+H)^+$. RP-HPLC R_t= 7.10 min, 98 %.

_N_-_t_-Butyl-4-(thiophen-3-yl)benzyl amine (19)

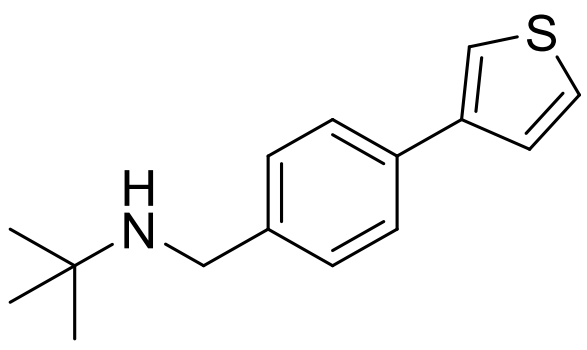

Prepared according to general procedure B with 4-(thiophen-3-yl)benzaldehyde (**S3**) (50 mg, 0.27 mmol, 1 eq.), *tert*-butylamine (28 µL, 0.27 mmol, 1 eq.) and sodium triacetoxyborohydride (79 mg, 0.37 mmol, 1.4 eq.). Purification by column chromatography using a stepped gradient from ethyl acetate to 97.5:2.5 ethyl acetate : triethylamine gave the title compound as a white solid (19 mg, 29 %).

^{1}H NMR (300 MHz, CD_3OD) δ 7.67 – 7.56 (m, 3H), 7.49 – 7.34 (m, 4H), 3.70 (s, 2H), 1.21 (s, 9H). ^{13}C NMR (75 MHz, CD_3OD) δ 143.2, 140.1, 136.0, 130.2, 127.4, 127.3, 127.1, 121.1, 52.1, 47.6, 28.7. ESI-MS *m/z* = 246.4 $(M+H)^+$. ESI-HRMS-TOF found 246.1315, calculated 246.1311 for $(M+H)^+$. RP-HPLC R_t= 7.48 min, 99 %.

_N_-Cyclohexyl-4-(thiophen-3-yl)benzyl amine (20)

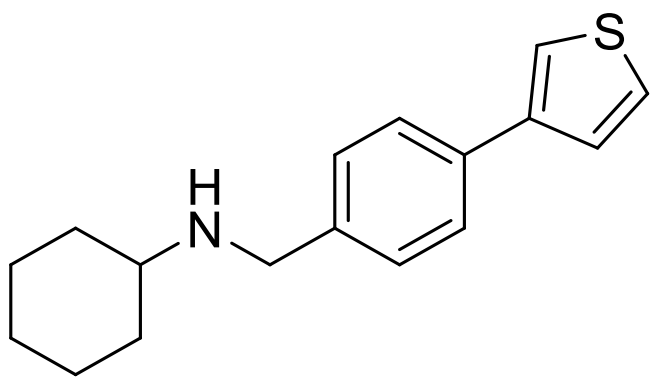

Prepared according to general procedure B with 4-(thiophen-3-yl)benzaldehyde (**S3**) (100 mg, 0.532 mmol, 1 eq.), cyclohexylamine (85 µL, 0.75 mmol, 1.4 eq.) and sodium triacetoxyborohydride (158 mg, 0.745 mmol, 1.4 eq.). Purification by column chromatography using a stepped gradient from 75:25:1 hexane : ethyl acetate : triethylamine to 50:50:1 hexane : ethyl acetate : triethylamine gave the title compound as a white solid (45 mg, 31%).

^{1}H NMR (300 MHz, CD_3OD) δ 7.69 – 7.51 (m, 3H), 7.51 – 7.29 (m, 4H), 3.76 (s, 2H), 2.46 (at, *J* = 10.0 Hz, 1H), 1.95 (ad, *J* = 11.0 Hz, 2H), 1.85 – 1.54 (m, 3H), 1.37 – 1.01 (m, 5H). ^{13}C NMR (75 MHz, CD_3OD) δ 143.3, 139.7, 136.1, 130.0, 127.4, 127.3, 127.1, 121.1, 57.0, 50.9, 33.7, 27.2, 26.2. ESI-MS *m/z* = 272.5 $(M+H)^+$. ESI-HRMS-TOF found 272.1481, calculated 272.1467 for $(M+H)^+$. RP-HPLC R_t= 8.82 min, 98 %.

_N_-Adamantyl-4-(thiophen-3-yl)benzyl amine (21)

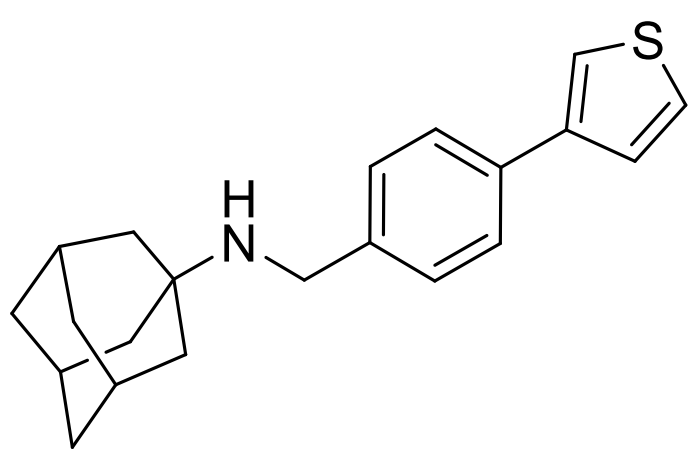

Prepared according to general procedure B with 4-(thiophen-3-yl)benzaldehyde (**S3**) (50 mg, 0.27 mmol, 1 eq.), adamantylamine (40 mg, 0.27 mmol, 1 eq.) and sodium triacetoxyborohydride (79 mg, 0.37 mmol, 1.4 eq.). Purification by column chromatography using a stepped gradient from ethyl acetate to 99:1 ethyl acetate : triethylamine gave the title compound as a white solid (3.6 mg, 5 %).

^{1}H NMR (300 MHz, CD_3OD) δ 7.66 – 7.58 (m, 3H), 7.49 – 7.42 (m, 2H), 7.38 (ad, *J* = 8.2 Hz, 2H), 3.75 (s, 2H), 2.11 (as, 3H), 1.83 – 1.78 (m, 6H), 1.78 – 1.64 (m, 6H). ^{13}C NMR (75 MHz,

CD$_3$OD) δ 143.2, 140.0, 136.1, 130.3, 127.4, 127.3, 127.1, 121.1, 52.7, 45.4, 42.7, 37.7, 31.1. ESI-MS m/z = 324.4 $(M+H)^+$. ESI-HRMS-TOF found 324.1794, calculated 324.1780 for $(M+H)^+$. RP-HPLC R_t= 9.57 min, 96 %.

***N*-Benzyl-4-(thiophen-3-yl)benzylamine (22)**

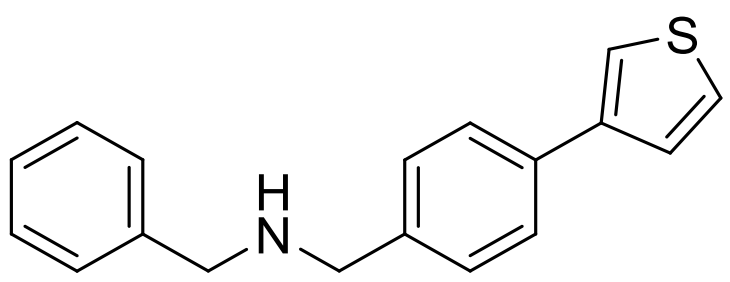

Prepared according to general procedure B with 4-(thiophen-3-yl)benzaldehyde (**S3**) (50 mg, 0.27 mmol, 1 eq.), benzylamine (29 µL, 0.27 mmol, 1 eq.) and sodium triacetoxyborohydride (79 mg, 0.37 mmol, 1.4 eq.). Purification by column chromatography using a stepped gradient from 60:40 hexane : ethyl acetate to 60:40 ethyl acetate : hexane gave the title compound as a white solid (22 mg, 30 %).

^{1}H NMR (300 MHz, CD$_3$OD) δ 7.67 – 7.52 (m, 3H), 7.44 (as, 2H), 7.40 – 7.20 (m, 7H), 3.74 (as, 4H). ^{13}C NMR (75 MHz, CD$_3$OD) δ 143.2, 140.3, 139.1, 136.2, 130.2, 129.7, 129.5, 128.3, 127.4, 127.3, 127.1, 121.1, 53.5, 53.2. ESI-MS m/z = 280.5 $(M+H)^+$. ESI-HRMS-TOF found 280.1163, calculated 280.1154 for $(M+H)^+$. RP-HPLC R_t= 8.69 min, 98 %.

***N*-Phenethyl-4-(thiophen-3-yl)benzylamine (23)**

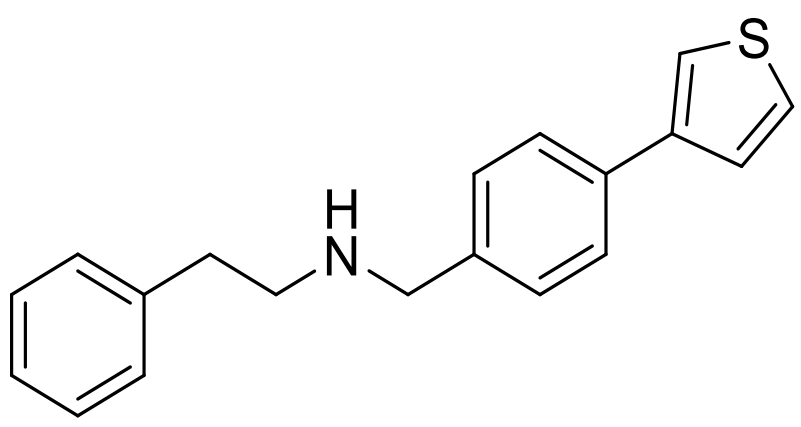

Prepared according to general procedure B with 4-(thiophen-3-yl)benzaldehyde (**S3**) (50 mg, 0.27 mmol, 1 eq.), phenethylamine (34 µL, 0.27 mmol, 1 eq.)

and sodium triacetoxyborohydride (79 mg, 0.37 mmol, 1.4 eq.). Purification by column chromatography in ethyl acetate gave the title compound as a white solid (29 mg, 37 %).

^{1}H NMR (300 MHz, CD_3OD) δ 7.64 – 7.50 (m, 3H), 7.42 (as, 2H), 7.34 – 7.21 (m, 4H), 7.21 – 7.12 (m, 3H), 3.74 (s, 2H), 2.80 (as, 4H). ^{13}C NMR (75 MHz, CD_3OD) δ 143.2, 141.0, 139.2, 136.2, 130.0 (2 carbons), 129.7, 129.6, 127.4, 127.3, 127.1, 121.1, 53.9, 51.4, 36.6. ESI-MS *m/z* = 294.4 $(M+H)^+$. ESI-HRMS-TOF found 294.1325, calculated 294.1311 for $(M+H)^+$. RP-HPLC R_t= 9.20 min, 99 %.

***N*-(4-Methoxybenzyl)-4-(thiophen-3-yl)benzyl amine (24)**

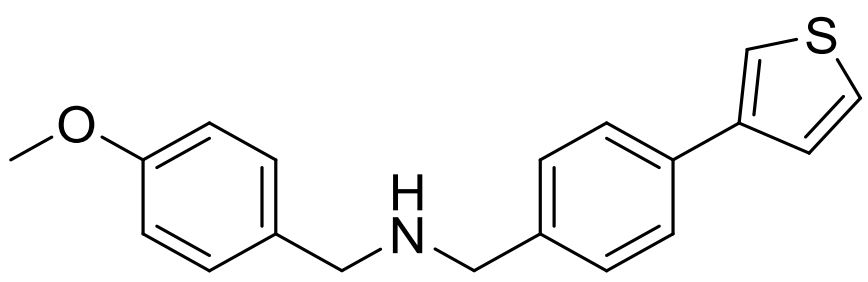

Prepared according to general procedure B with 4-(thiophen-3-yl)benzaldehyde (**S3**) (50 mg, 0.27 mmol, 1 eq.), p-methoxybenzylamine (35 µL, 0.27 mmol, 1 eq.) and sodium triacetoxyborohydride (79 mg, 0.37 mmol, 1.4 eq.). Purification by column chromatography using a stepped gradient from 75:25 hexane : ethyl acetate to 75:25 ethyl acetate : hexane gave the title compound as a white solid (59 mg, 72 %).

^{1}H NMR (300 MHz, CD_3OD) δ 7.66 – 7.57 (m, 3H), 7.49 – 7.41 (m, 2H), 7.35 (d, *J* = 7.9 Hz, 2H), 7.25 (ad, *J* = 8.3 Hz, 2H), 6.88 (ad, *J* = 8.3 Hz, 2H), 3.77 (s, 3H), 3.73 (s, 2H), 3.69 (s, 2H). ^{13}C NMR (75 MHz, CD_3OD) δ 160.4, 143.3, 139.2, 136.2, 132.4, 130.9, 130.2, 127.4, 127.3, 127.1, 121.1, 114.9, 55.7, 53.1, 52.9. ESI-MS *m/z* = 310.4 $(M+H)^+$. ESI-HRMS-TOF found 310.1273, calculated 310.1260 for $(M+H)^+$. RP-HPLC R_t= 8.97 min, 99 %.

***N*-(4-(Thiophen-3-yl)benzyl)piperidine (25)**

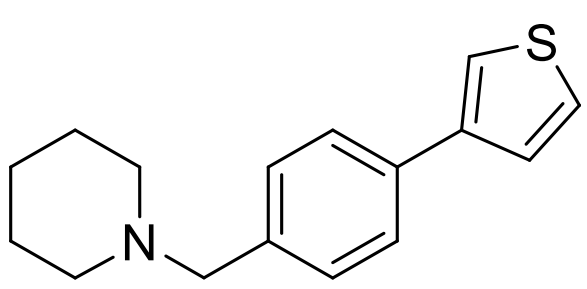

Prepared according to general procedure B with 4-(thiophen-3-yl)benzaldehyde (**S3**) (50 mg, 0.27 mmol, 1 eq.), piperidine (26 µL, 0.27 mmol, 1 eq.) and

sodium triacetoxyborohydride (79 mg, 0.37 mmol, 1.4 eq.). Purification by column chromatography in ethyl acetate gave the title compound as a white solid (28 mg, 41 %).

^{1}H NMR (300 MHz, CD_3OD) δ 7.67 – 7.56 (m, 3H), 7.48 – 7.41 (m, 2H), 7.33 (ad, J = 8.1 Hz, 2H), 3.50 (s, 2H), 2.43 (as, 4H), 1.65 – 1.52 (m, 4H), 1.52 – 1.38 (m, 2H). ^{13}C NMR (75 MHz, CD_3OD) δ 143.2, 136.8, 136.4, 131.5, 127.3, 127.1 (2 carbons), 121.2, 64.3, 55.2, 26.4, 25.1. ESI-MS m/z = 258.5 $(M+H)^+$. ESI-HRMS-TOF found 258.1311, calculated 258.1311 for $(M+H)^+$. RP-HPLC R_t= 8.12 min, 98 %.

***N*-(4-(Thiophen-3-yl)benzyl)morpholine (26)**

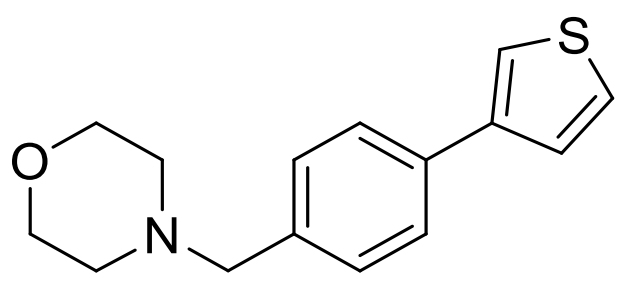

Prepared according to general procedure B with 4-(thiophen-3-yl)benzaldehyde (**S3**) (50 mg, 0.27 mmol, 1 eq.), morpholine (23 µL, 0.27 mmol, 1 eq.) and sodium triacetoxyborohydride (79 mg, 0.37 mmol, 1.4 eq.). Purification by column chromatography using a stepped gradient from 70:30 ethyl acetate : hexane to ethyl acetate gave the title compound as a white solid (36 mg, 52 %).

^{1}H NMR (600 MHz, CD_3OD) δ 7.66 – 7.52 (m, 3H), 7.52 – 7.38 (m, 2H), 7.34 (ad, J = 8.0 Hz, 2H), 3.73 – 3.59 (m, 4H), 3.50 (s, 2H), 2.45 (as, 4H). ^{13}C NMR (150 MHz, CD_3OD) δ 143.3, 137.1, 136.6, 131.3, 127.4, 127.4, 127.2, 121.3, 67.8, 64.1, 54.7. ESI-MS m/z = 260.3 $(M+H)^+$. ESI-HRMS-TOF found 260.1107, calculated 260.1104 for $(M+H)^+$. RP-HPLC R_t= 7.12 min, 98 %.

***N,N*-Dimethyl-4-(thiophen-3-yl)benzylamine (27)**

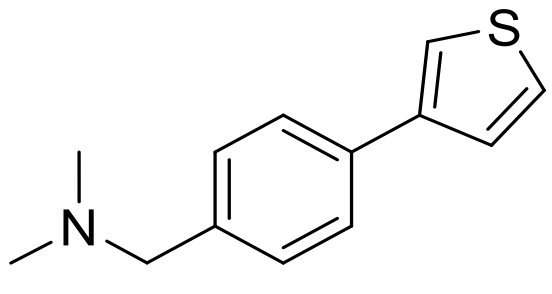

Prepared according to general procedure B with 4-(thiophen-3-yl)benzaldehyde (**S3**) (50 mg, 0.27 mmol, 1 eq.), dimethylamine (33 µL, 40 % in H_2O,

0.27 mmol, 1 eq.) and sodium triacetoxyborohydride (113 mg, 0.531 mmol, 2.0 eq.). Purification by column chromatography using a stepped gradient from ethyl acetate to 90:10:1 ethyl acetate : methanol : triethylamine gave the title compound as a white solid (20 mg, 35%). ^{1}H NMR (600 MHz, CD_3OD) δ 7.65 – 7.56 (m, 3H), 7.48 – 7.40 (m, 2H), 7.32 (ad, J = 8.1 Hz, 2H), 3.46 (s, 2H), 2.24 (s, 6H). ^{13}C NMR (150 MHz, CD_3OD) δ 143.2, 137.4, 136.5, 131.2, 127.3, 127.2, 127.1, 121.3, 64.6, 45.2. ESI-MS m/z = 218.3 $(M+H)^+$. ESI-HRMS-TOF found 218.0990, calculated 218.0998 for $(M+H)^+$. RP-HPLC R_t= 7.28 min, 99 %.

4-(Thiophen-3-yl)benzyl amine (28)

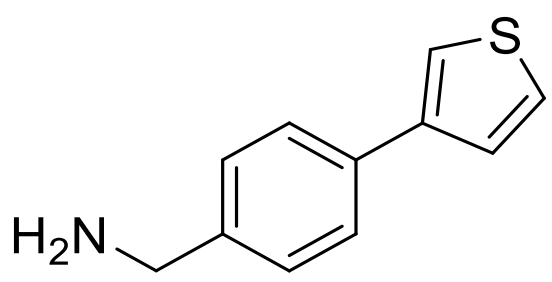

Prepared according to general procedure C with 4-(thiophen-3-yl)benzaldehyde (**S3**) (200 mg, 1.06 mmol, 1 eq.) and hydroxylamine hydrochloride (148 mg, 2.13 mmol, 2 eq.). Purification by column chromatography using a stepped gradient from 100:1 ethyl acetate : triethylamine to 85:15:1 ethyl acetate : methanol : triethylamine gave the title compound as an orange solid (110 mg, 55 %). The analytical data agreed with the purchased Maybridge compound.

^{1}H NMR (600 MHz, CD_3OD) δ 7.66 – 7.62 (m, 2H), 7.60 (dd, J = 2.8, 1.4 Hz, 1H), 7.46 (dd, J = 5.0, 2.9 Hz, 1H), 7.44 (dd, J = 5.0, 1.4 Hz, 1H), 7.38 (ad, J = 8.3 Hz, 2H), 3.88 (s, 2H). ^{13}C NMR (150 MHz, CD_3OD) δ 143.1, 139.7, 136.5, 129.4, 127.6, 127.4, 127.1, 121.3, 45.7. ESI-MS m/z = 173.2 $(M-NH_2)^+$. RP-HPLC R_t= 6.29 min, 99 %.

***N*-(2-Hydroxyethyl)-4-(thiophen-3-yl)benzylamine (29)**

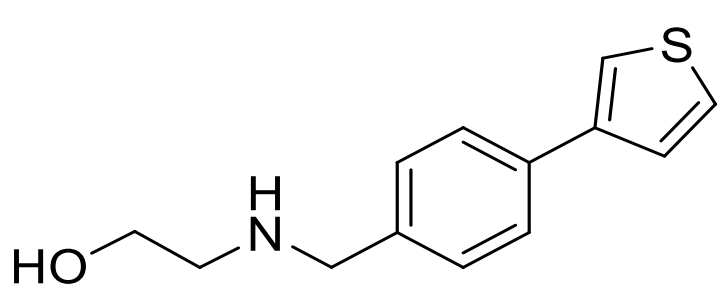

Prepared according to general procedure B with 4-(thiophen-3-yl)benzaldehyde (**S3**) (69 mg, 0.37 mmol, 1 eq.), ethanolamine (22 µL, 0.37 mmol, 1 eq.) and

sodium triacetoxyborohydride (108 mg, 0.512 mmol, 1.4 eq.). Purification by column chromatography using a stepped gradient from ethyl acetate to 85:15:1 ethyl acetate : methanol : triethylamine gave the title compound as a white solid (24 mg, 28 %).

^{1}H NMR (400 MHz, CD_3OD) δ 7.62 (ad, *J* = 8.1 Hz, 2H), 7.58 (dd, *J* = 2.8, 1.5 Hz, 1H), 7.44 (m, 2H), 7.36 (ad, *J* = 8.2 Hz, 2H), 3.79 (s, 2H), 3.68 (abs, 2H), 2.74 (abs, 2H). ^{13}C NMR (100 MHz, CD_3OD) δ 143.2, 139.2, 136.3, 130.1, 127.4, 127.3, 127.1, 121.2, 61.5, 53.9, 51.6. ESI-MS *m/z* = 234.3 $(M+H)^+$. ESI-HRMS-TOF found 234.0957, calculated 234.0947 for $(M+H)^+$. RP-HPLC R_t= 6.78 min, 96 %.

Ethyl 2-(4-(thiophene-3-yl)benzylamino)acetate (30)

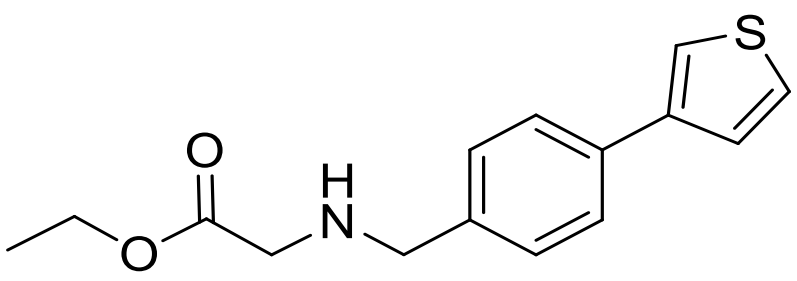

Prepared according to general procedure B with 4-(thiophen-3-yl)benzaldehyde (**S3**) (50 mg, 0.27 mmol, 1 eq.), glycine ethyl ester hydrochloride (27 mg, 0.27 mmol, 1 eq.) and sodium triacetoxyborohydride (79 mg, 0.37 mmol, 1.4 eq.). Purification by column chromatography using a stepped gradient from 80:20 hexane : ethyl acetate to 50:50 hexane : ethyl acetate gave the title compound as a white solid (22 mg, 31 %).

^{1}H NMR (400 MHz, CD_3OD) δ 7.65 – 7.60 (m, 2H), 7.59 (dd, *J* = 2.8, 1.5 Hz, 1H), 7.45 (m, 2H), 7.38 – 7.33 (m, 2H), 4.18 (q, *J* = 7.1 Hz, 2H), 3.78 (s, 2H), 3.38 (s, 2H), 1.26 (t, *J* = 7.1 Hz, 3H). ^{13}C NMR (100 MHz, CD_3OD) δ 173.2, 143.2, 138.8, 136.4, 130.2, 127.4, 127.3, 127.1, 121.2, 61.9, 53.4, 50.2, 14.5. ESI-MS *m/z* = 276.3 $(M+H)^+$. ESI-HRMS-TOF found 276.1059, calculated 276.1053 for $(M+H)^+$. RP-HPLC R_t= 7.79 min, 95 %.

1-(Pyridin-3-yl)-*N*-(4-(thiophen-3-yl)benzyl)methanamine (31)

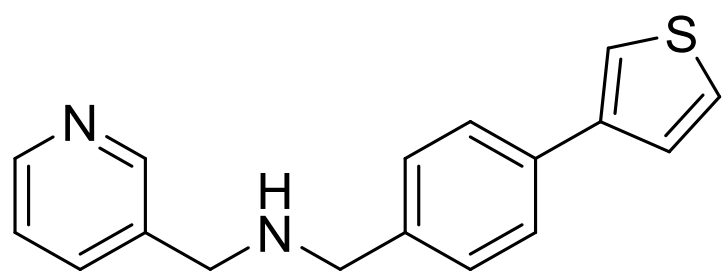 Prepared according to general procedure B with 4-(thiophen-3-yl)benzaldehyde (**S3**) (50 mg, 0.27 mmol, 1 eq.), 3-picolylamine (27 µL, 0.27 mmol, 1 eq.) and sodium triacetoxyborohydride (79 mg, 0.37 mmol, 1.4 eq.). Purification by column chromatography using a stepped gradient from 70:30:1 ethyl acetate : hexane : triethylamine to 90:10:1 ethyl acetate : methanol : triethylamine gave the title compound as a clear oil (50 mg, 67 %).

^{1}H NMR (400 MHz, CD_3OD) δ 8.49 (s, 1H), 8.41 (d, *J* = 4.1 Hz, 1H), 7.81 (dt, *J* = 7.8, 1.8 Hz, 1H), 7.63 – 7.57 (m, 2H), 7.56 (dd, *J* = 2.8, 1.5 Hz, 1H), 7.46 – 7.40 (m, 2H), 7.40 – 7.31 (m, 3H), 3.76 (s, 2H), 3.74 (s, 2H). ^{13}C NMR (100 MHz, CD_3OD) δ 150.3, 148.7, 143.2, 139.2, 138.4, 137.2, 136.2, 130.1, 127.4, 127.3, 127.1, 125.2, 121.1, 53.4, 50.7. ESI-MS *m/z* = 281.2 $(M+H)^+$. ESI-HRMS-TOF found 281.1117, calculated 281.1107 for $(M+H)^+$. RP-HPLC R_t= 6.02 min, 99 %.

3-(Thiophen-3-yl)benzylamine (32)

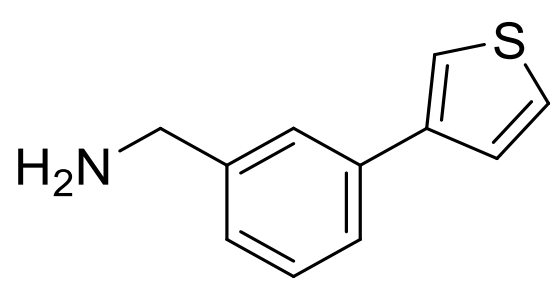 Prepared according to general procedure C with 3-(thiophen-3-yl)benzaldehyde (**S1**) (100 mg, 5.03 mmol, 1 eq.) and hydroxylamine hydrochloride (74 mg, 1.1 mmol, 2 eq.) to give 3-(thiophen-3-yl)benzaldehyde oxime (94 mg, 0.46 mmol) and lithium aluminium hydride (35 mg, 0.93 mmol, 2 eq.). Purification using a stepped gradient from 100:1 ethyl acetate : triethylamine to 85:15:1 ethyl acetate : methanol : triethylamine gave the title compound as an orange solid (35 mg, 34 %).

^{1}H NMR (400 MHz, CD_3OD) δ 7.65 (atd, *J* = 1.7, 0.5 Hz, 1H), 7.62 (dd, *J* = 2.4, 1.9 Hz, 1H), 7.54 (adt, *J* = 7.7, 1.5 Hz, 1H), 7.48 – 7.44 (m, 2H), 7.35 (t, *J* = 7.7 Hz, 1H), 7.26 – 7.22 (m,

1H), 3.84 (s, 2H). ^{13}C NMR (100 MHz, CD_3OD) δ 143.7, 143.5, 137.5, 130.1, 127.24 (2 carbons), 127.18, 126.5, 126.0, 121.3, 46.6. ESI-MS *m/z* = 190.2 $(M+H)^+$. ESI-HRMS-TOF found 190.0687, calculated 190.0685 for $(M+H)^+$. RP-HPLC R_t= 6.34 min, 99 %.

N-Cyclohexyl-3-(thiophen-3-yl)benzyl amine (33)

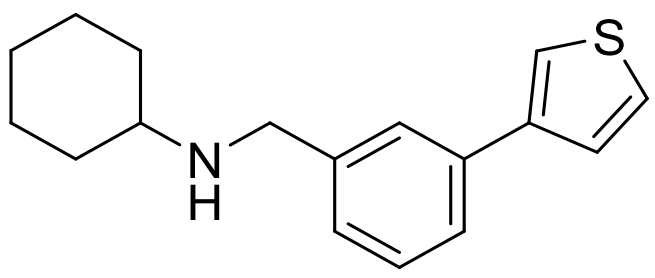

Prepared according to general procedure B with 3-(thiophen-3-yl)benzaldehyde (**S1**) (86 mg, 0.46 mmol, 1 eq.), cyclohexylamine (52 µL, 0.46 mmol, 1 eq.) and sodium triacetoxyborohydride (136 mg, 0.640 mmol, 1.4 eq.). Purification by column chromatography using a stepped gradient from 100:1 hexane : triethylamine to 70:30:1 hexane : ethyl acetate : triethylamine gave the title compound as a clear oil (92 mg, 74 %).

^{1}H NMR (400 MHz, CD_3OD) δ 7.64 (at, *J* = 1.5 Hz, 1H), 7.60 (dd, *J* = 2.7, 1.6 Hz, 1H), 7.53 (adt, *J* = 8.0, 1.6 Hz, 1H), 7.47 – 7.43 (m, 2H), 7.33 (at, *J* = 7.6 Hz, 1H), 7.23 (ad, *J* = 7.8 Hz, 1H), 3.77 (s, 2H), 2.54 – 2.39 (m, 1H), 2.03 – 1.89 (m, 2H), 1.81 – 1.70 (m, 2H), 1.68 – 1.56 (m, 1H), 1.33 – 1.04 (m, 5H). ^{13}C NMR (100 MHz, CD_3OD) δ 143.5, 141.6, 137.6, 130.0, 128.2, 127.5, 127.3, 127.2, 126.1, 121.3, 57.2, 51.4, 33.7, 27.2, 26.2. ESI-MS *m/z* = 272.5 $(M+H)^+$. ESI-HRMS-TOF found 272.1477, calculated 272.1467 for $(M+H)^+$. RP-HPLC R_t= 8.69 min, 98 %.

2-(3-(Thiophen-3-yl)benzylamino)acetamide (34)

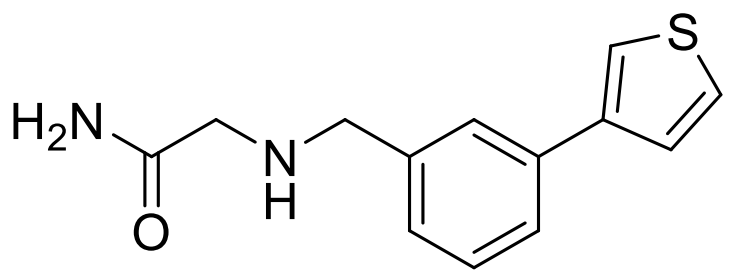 Prepared according to general procedure B with 3-(thiophen-3-yl)benzaldehyde (**S1**) (121 mg, 0.643 mmol, 1 eq.), glycinamide (167 mg, 0.964 mmol, 1.5 eq.) and sodium triacetoxyborohydride (206 mg, 0.964 mmol, 1.5 eq.). Purification by column chromatography using a stepped gradient from 75:25:1 hexane : ethyl acetate : triethylamine to 90:10:1 ethyl acetate : methanol : triethylamine gave the title compound as a clear oil (65 mg, 30%).

^{1}H NMR (400 MHz, $CDCl_3$) δ 7.54 – 7.49 (m, 2H), 7.46 (dd, *J* = 2.7, 1.6 Hz, 1H), 7.41 – 7.35 (m, 3H), 7.24 (bd, *J* = 7.8 Hz, 1H), 7.02 (bs, 1H), 5.51 (bs, 1H), 3.85 (s, 2H), 3.35 (s, 2H). ^{13}C NMR (100 MHz, $CDCl_3$) δ 174.5, 142.2, 140.0, 136.4, 129.3, 127.0, 126.5 (2 carbons), 126.3, 125.7, 120.7, 54.1, 52.0. ESI-MS *m/z* = 247.1 $(M+H)^+$. ESI-HRMS-TOF found 247.0901, calculated 247.0900 for $(M+H)^+$. RP-HPLC R_t= 6.08 min, 99 %.

1-(4-(Thiophen-3-yl)benzyl)azetidine (35)

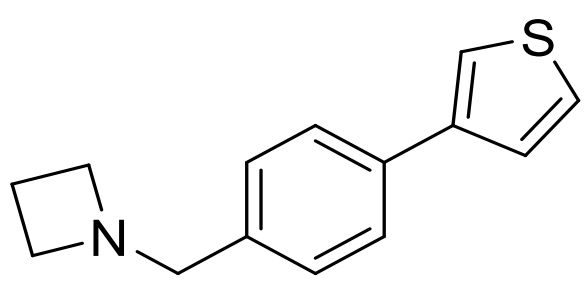 4-(Thiophen-3-yl)benzaldehyde (**S3**) (103 mg, 0.547 mmol, 1 eq.) and 3-bromopropylamine hydrobromide (120 mg, 0.547 mmol, 1 eq.) were added to acetonitrile (2 mL) followed by addition of sodium triacetoxyborohydride (162 mg, 0.766 mmol, 1.4 eq.). The solution was sealed under a nitrogen atmosphere and stirred for 2 hours at room temperature. The solution was then diluted with sodium hydroxide (1 M, 15 mL) and extracted with ethyl acetate (3 × 15 mL). The combined organic layers were dried over anhydrous magnesium sulfate, filtered, and concentrated to 2 mL, the solution was left at room temperature for 30 mins then diluted with DMSO (4 ml). Sodium azide (178 mg, 2.74 mmol, 5 eq.) was then added to the solution and sealed under a nitrogen atmosphere. The solution was

left stirring at room temperature for 22 hours then diluted with NaOH (aq., 1 M, 15 mL) and extracted with ethyl acetate (3 × 15 mL). The combined organic layers were dried over anhydrous magnesium sulfate, filtered, and evaporated to give the crude product. Purification by column chromatography using a stepped gradient from 100:1 hexane : triethylamine to 60:40:1

hexane : ethyl acetate : triethylamine gave the azetidine product as a white solid (46 mg, 37%). ^{1}H NMR (600 MHz, CD_3OD) δ 7.61 – 7.53 (m, 3H), 7.45 – 7.39 (m, 2H), 7.26 (ad, *J* = 8.3 Hz, 2H), 3.54 (s, 2H), 3.23 (t, *J* = 7.2 Hz, 4H), 2.06 (p, *J* = 7.1 Hz, 2H). ^{13}C NMR (150 MHz, CD_3OD) δ 143.1, 137.2, 136.3, 130.4, 128.5, 127.3, 127.1, 121.2, 63.9, 55.6, 18.3. ESI-MS *m/z* = 230.2 $(M+H)^+$. ESI-HRMS-TOF found 173.0410, calculated 173.0425 for $(M-(CH_2)_3N)^+$. RP-HPLC R_t= 7.04 min, 96 %.

Methyl 2-(3-(thiophen-3-yl)benzylamino)propanoate (36)

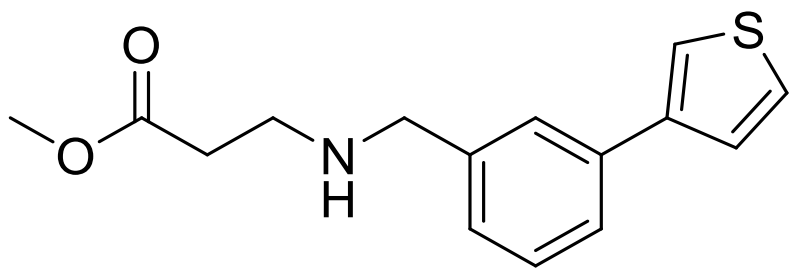

Prepared according to general procedure B with 3-(thiophen-3-yl)benzaldehyde (**S1**) (150 mg, 0.797 mmol, 1 eq.), β-alanine methyl ester hydrochloride (**S5**) (156 mg, 1.12 mmol, 1.4 eq.) and sodium triacetoxyborohydride (236 mg, 1.12 mmol, 1.4 eq.). Purification by column chromatography using a stepped gradient from 100:1 hexane : triethylamine to 80:20:1 ethyl acetate : hexane : triethylamine gave the title compound as a yellow oil (158 mg, 72 %).

^{1}H NMR (400 MHz, $CDCl_3$) δ 7.57-7.56 (m, 1H), 7.51 – 7.45 (m, 2H), 7.42 – 7.33 (m, 3H), 7.26 – 7.23 (m, 1H), 3.85 (s, 2H), 3.68 (s, 3H), 2.93 (t, *J* = 6.5 Hz, 2H), 2.55 (t, *J* = 6.5 Hz, 2H). ^{13}C NMR (100 MHz, $CDCl_3$) δ 173.3, 142.4, 140.8, 136.1, 129.0, 127.0, 126.5, 126.3, 126.3, 125.3, 120.5, 53.9, 51.7, 44.6, 34.7. ESI-MS *m/z* = 276.1 $(M+H)^+$. ESI-HRMS-TOF found 276.1055, calculated 276.1053 for $(M+H)^+$. RP-HPLC R_t= 7.00 min, 97 %.

Methyl 6-(3-(thiophen-3-yl)benzylamino)hexanoate (37)

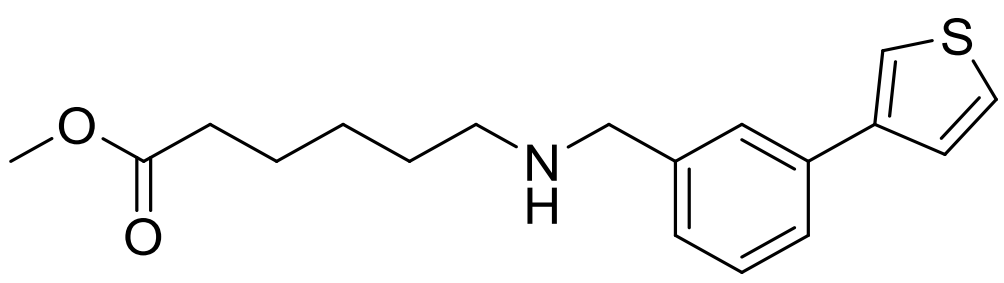
Prepared according to general procedure B with 3-(thiophen-3-yl)benzaldehyde (**S1**) (150 mg, 0.797 mmol, 1 eq.), 6-aminocaproic acid methyl ester hydrochloride (**S8**) (174 mg, 0.956 mmol, 1.2 eq.) and sodium triacetoxyborohydride (236 mg, 1.12 mmol, 1.4 eq.). Purification by column chromatography using a stepped gradient from 100:1 hexane : triethylamine to 50:50:1 hexane : ethyl acetate : triethylamine gave the title compound as a clear oil (69 mg, 27 %).

^{1}H NMR (400 MHz, $CDCl_3$) δ 7.55 (bs, 1H), 7.48 (adt, *J* = 7.8, 1.4 Hz, 1H), 7.46 (dd, *J* = 2.8, 1.4 Hz, 1H), 7.40 (dd, *J* = 1.4, 5.0 Hz, 1H), 7.38 (dd, *J* = 2.8, 5.0 Hz, 1H), 7.35 (at, *J* = 7.7 Hz, 1H), 7.24 (bd, *J* = 7.6 Hz, 1H), 3.83 (s, 2H), 3.66 (s, 3H), 2.65 (t, *J* = 7.0 Hz, 2H), 2.31 (t, *J* = 7.5 Hz, 2H), 1.64 (ap, *J* = 7.6 Hz, 2H), 1.54 (ap, *J* = 7.6 Hz, 2H), 1.33-1.41 (m, 2H). ^{13}C NMR (100 MHz, $CDCl_3$) δ 174.1, 142.3, 140.9, 135.9, 128.8, 126.9, 126.3, 126.2, 126.1, 125.0, 120.3, 54.0, 51.4, 49.2, 33.9, 29.7, 26.8, 24.8. ESI-MS *m/z* = 318.1 $(M+H)^+$. ESI-HRMS-TOF found 318.1530, calculated 318.1522 for $(M+H)^+$. RP-HPLC R_t= 8.35 min, 97 %

***N*-(3-(Thiophen-3-yl)benzyl)pyrrolidin-2-one (38)**

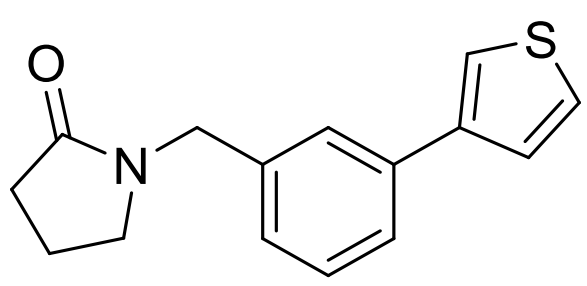
Prepared according to general procedure B with 3-(thiophen-3-yl)benzaldehyde (**S1**) (150 mg, 0.806 mmol, 1 eq.), 4-aminobutanoic acid methyl ester hydrochloride (**S6**) (173 mg, 1.13 mmol, 1.4 eq.) and sodium triacetoxyborohydride (239 mg, 1.13 mmol, 1.4 eq.). Purification by column chromatography using a stepped gradient from

100:1 hexane : triethylamine to 70:30:1 ethyl acetate: hexane: triethylamine gave the title compound as a clear oil (8 mg, 4 %) as well as a 3.5:1 mixture of lactam/methyl ester (98 mg, 46 %) which was used in the next step for hydrolysis. *The methyl ester slowly converted to the lactam at room temperature. Lactam **38** characterisation:

^{1}H NMR (400 MHz, $CDCl_3$) δ 7.54 – 7.50 (m, 1H), 7.48 – 7.45 (m, 2H), 7.40 – 7.33 (m, 3H), 7.17 (d, *J* = 7.6 Hz, 1H), 4.49 (s, 2H), 3.33 – 3.24 (m, 2H), 2.46 (t, *J* = 8.1 Hz, 2H), 2.06 – 1.94 (m, 2H). ^{13}C NMR (100 MHz, $CDCl_3$) δ 175.1, 142.1, 137.4, 136.5, 129.3, 127.1, 126.5 (2carbons), 126.4, 125.9, 120.8, 46.8 (2 carbons), 31.1, 17.9. ESI-MS *m/z* = 258.1 $(M+H)^+$. ESI-HRMS-TOF found 258.0952, calculated 258.0947 for $(M+H)^+$. RP-HPLC R_t= 10.14 min, 97 %.

***N*-(3-(Thiophen-3-yl)benzyl)piperidin-2-one (39)**

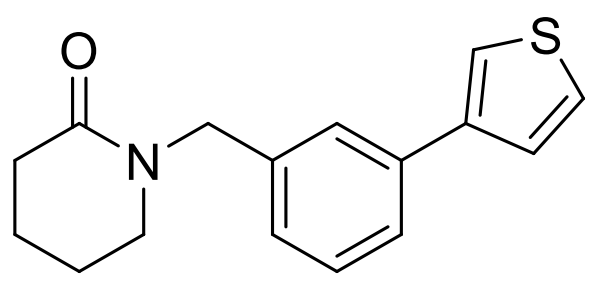

Prepared according to general procedure B with 3-(thiophen-3-yl)benzaldehyde (**S1**) (150 mg, 0.797 mmol, 1 eq.), 5-aminovaleric acid methyl ester hydrochloride (**S7**) (187 mg, 1.12 mmol, 1.4 eq.) and sodium triacetoxyborohydride (236 mg, 1.12 mmol, 1.4 eq.). Purification by column chromatography using a stepped gradient from 100:1 hexane : triethylamine to 50:50:1 hexane : ethyl acetate : triethylamine gave the title compound as a clear oil (166 mg, 77 %).

^{1}H NMR (400 MHz, $CDCl_3$) δ 7.52 – 7.46 (m, 2H), 7.45 (at, *J* = 2.2 Hz, 1H), 7.38 (d, *J* = 2.2 Hz, 2H), 7.35 (at, *J* = 7.6 Hz, 1H), 7.18 (bd, *J* = 7.6 Hz, 1H), 4.64 (s, 2H), 3.22 (t, *J* = 6.0 Hz, 2H), 2.48 (t, *J* = 6.4 Hz, 2H), 1.85 – 1.71 (m, 4H). ^{13}C NMR (100 MHz, $CDCl_3$) δ 170.0, 142.2, 138.1, 136.3, 129.2, 127.0, 126.5, 126.4, 126.3, 125.6, 120.6, 50.2, 47.4, 32.6, 23.3, 21.5. ESI-MS *m/z* = 272.1 $(M+H)^+$. ESI-HRMS-TOF found 272.1099, calculated 272.1104for $(M+H)^+$. RP-HPLC R_t= 10.6 min, 98 %.

Lithium 2-(3-(thiophen-3-yl)benzylamino)acetate (40)

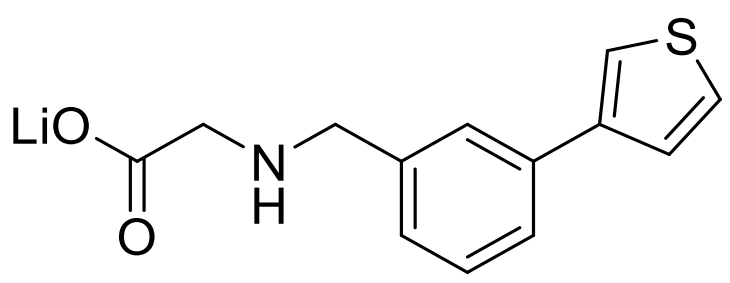

Methyl 2-(3-(thiophen-3-yl)benzylamino)methanoate (**S4**) (69 mg, 0.26 mmol, 1 eq.) and lithium hydroxide (9 mg, 0.394 mmol, 1.5 eq.) were dissolved in 3:1 methanol : water (4 mL) and stirred at room temperature for 4 hours. The reaction was evaporated to dryness and purified by reversed-phase C18 column chromatography using a stepped gradient from water to methanol to give the title compound as a white solid (47 mg, 72 %).

^{1}H NMR (400 MHz, D_2O/CD_3OD, 1:1) δ 7.70 – 7.65 (m, 2H), 7.62 (bd, *J* = 7.8 Hz, 1H), 7.53 (dd, *J* = 5.0, 2.9 Hz, 1H), 7.50 (dd, *J* = 5.0, 1.4 Hz, 1H), 7.43 (at, *J* = 7.7 Hz, 1H), 7.32 (bd, *J* = 7.6 Hz, 1H), 3.87 (s, 2H), 3.26 (s, 2H). ^{13}C NMR (100 MHz, D_2O/CD_3OD) δ 178.4, 142.6, 139.5, 136.9, 130.3, 128.5, 127.8, 127.4, 127.0, 126.4, 121.7, 53.1, 52.1. ESI-MS *m/z* = 248.1 $(M+H)^+$. ESI-HRMS-TOF found 248.0731, calculated 248.0740 for $(M+H)^+$. RP-HPLC R_t= 6.24 min, >99 %.

Lithium 3-(3-(thiophen-3-yl)benzylamino)propanoate (41)

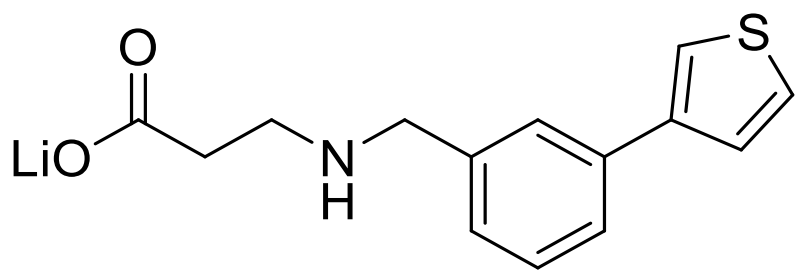

Methyl 2-(3-(thiophen-3-yl)benzylamino)propanoate (**36**) (60 mg, 0.22 mmol, 1 eq.) and lithium hydroxide (8 mg, 0.325 mmol, 1.5 eq.) were dissolved in 3:1 methanol : water (4 mL) and stirred at room temperature for 4 hours. The reaction was evaporated to dryness and purified by reversed-phase C18 column chromatography using a stepped gradient from water to 75:25 methanol : water to give the title compound as a white solid (53 mg, 91 %).

^{1}H NMR (400 MHz, D_2O/CD_3OD, 1:1) δ 7.69 (dd, *J* = 2.9, 1.4 Hz, 1H), 7.65 (bs, 1H), 7.61 (bd, *J* = 7.9 Hz, 1H), 7.54 (dd, *J* = 5.0, 2.9 Hz, 1H), 7.51 (dd, *J* = 5.0, 1.4 Hz, 1H), 7.43 (at, *J* = 7.7 Hz, 1H), 7.30 (bd, *J* = 7.7 Hz, 1H), 3.80 (s, 2H), 2.82 (t, *J* = 7.2 Hz, 2H), 2.40 (t, *J* = 7.2 Hz, 2H). ^{13}C NMR (100 MHz, D_2O/CD_3OD) δ 181.6, 142.6, 140.5, 136.7, 130.2, 128.4, 127.9, 127.2, 127.1, 126.1, 121.8, 53.3, 46.0, 37.8. ESI-MS *m/z* = 262.1 $(M+H)^+$. ESI-HRMS-TOF found 262.0894, calculated 262.0896 for $(M+H)^+$. RP-HPLC R_t= 6.53 min, >99 %.

Potassium 4-(3-(Thiophen-3-yl)benzylamino)butanoate (42)

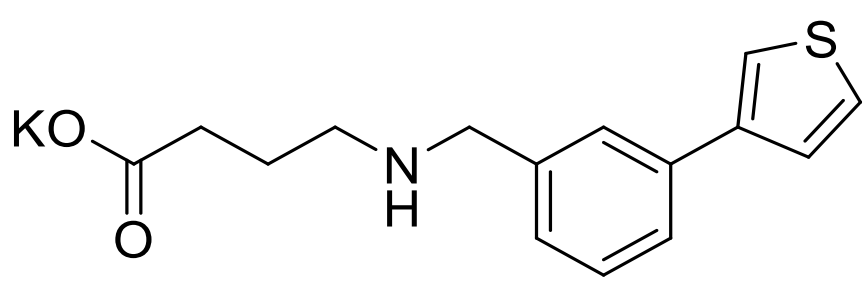

N-(3-(Thiophen-3-yl)benzyl)pyrrolidinone (**38**) (40 mg, 0.15 mmol, 1 eq.) was dissolved in KOH (5 mL, 2.5 M in 40:60 ethanol : water) and heated to reflux for 17 h. The reaction was cooled and evaporated to dryness and purified by reversed-phase C18 column chromatography using a stepped gradient from water to 75:25 methanol : water to give the title compound as a white solid (16 mg, 38 %).

^{1}H NMR (400 MHz, CD_3OD) δ 7.79 (at, *J* = 6.5 Hz, 1H), 7.72 – 7.67 (m, 2H), 7.52 – 7.48 (m, *J* = 2.6 Hz, 2H), 7.45 (at, *J* = 7.7 Hz, 1H), 7.37 (bd, *J* = 7.7 Hz, 1H), 4.15 (s, 2H), 3.06 (t, *J* = 6.3 Hz, 2H), 2.37 (t, *J* = 6.5 Hz, 2H), 1.88 (ap, *J* = 6.5 Hz, 2H). ^{13}C NMR (100 MHz, CD_3OD) δ 181.2, 142.7, 138.1, 134.3, 130.7, 129.1, 128.5, 128.0, 127.6, 127.1, 122.1, 52.1, 49.3, 37.2, 23.6. ESI-MS *m/z* = 276.1 $(M+H)^+$. ESI-HRMS-TOF found 276.1062, calculated 276.1053 for $(M+H)^+$. RP-HPLC R_t= 6.47 min, 99 %.

Potassium 5-(3-(thiophen-3-yl)benzylamino)valerate (43)

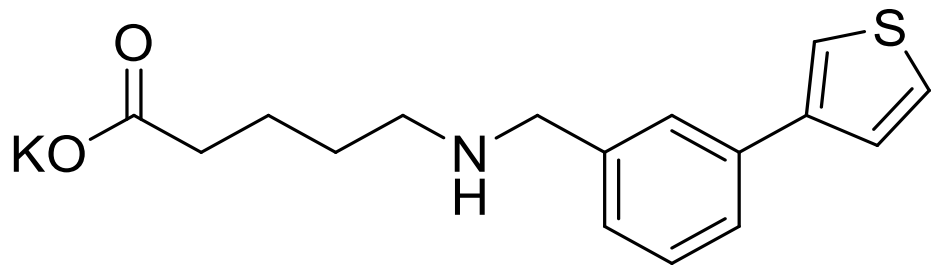

N-(3-(Thiophen-3-yl)benzyl)piperidineone (**39**) (109 mg, 0.400 mmol, 1 eq.) was dissolved in KOH (10 mL, 2.5 M in 40:60 ethanol : water) and heated to reflux for 17 h. The reaction was cooled and evaporated to dryness and purified by reversed-phase C18 column chromatography using a stepped gradient from water to 75:25 methanol : water to give the title compound as a white solid (119 mg, 91 %).

^{1}H NMR (400 MHz, 2:1 D_2O:CD_3CN) δ 7.60 (dd, *J* = 2.8, 1.4 Hz, 1H), 7.56 (s, 1H), 7.53 (d, *J* = 7.8 Hz, 1H), 7.47 (dd, *J* = 5.0, 2.9 Hz, 1H), 7.44 (dd, *J* = 5.0, 1.4 Hz, 1H), 7.35 (at, *J* = 7.6 Hz, 1H), 7.21 (d, *J* = 7.6 Hz, 1H), 3.68 (s, 2H), 2.47 (t, *J* = 7.0 Hz, 2H), 2.04 (t, *J* = 7.1 Hz, 2H), 1.49 – 1.33 (m, 4H). ^{13}C NMR (100 MHz, 2:1 D_2O:CD_3CN) δ 183.5, 142.6, 141.0, 136.4, 130.1, 128.4, 128.0, 127.2, 127.1, 125.9, 121.8, 53.3, 48.7, 38.4, 29.5, 24.7. ESI-MS *m/z* = 290.1 $(M+H)^+$. RP-HPLC R_t= 6.82 min, 99 %.

Lithium 6-(3-(Thiophen-3-yl)benzylamino)caproate (44)

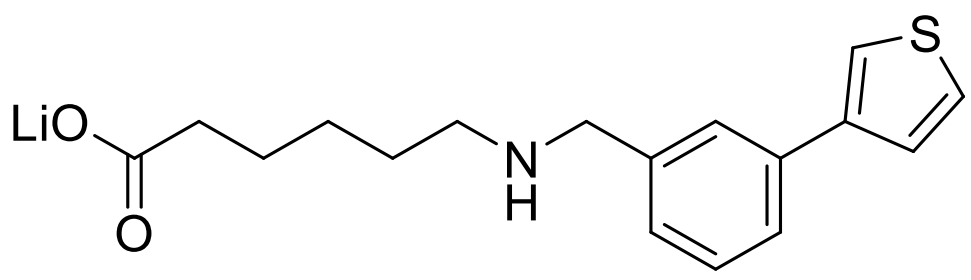

Methyl 6-(3-(thiophen-3-yl)benzylamino)hexanoate (**37**) (54 mg, 0.17 mmol, 1 eq.) and lithium hydroxide (12 mg, 0.51 mmol, 3 eq.) were added sequentially to methanol : water (3:1, 4 mL) and stirred at room temperature for 4 hours. The reaction mixture was then evaporated to dryness and purified by reversed-phase C18 column chromatography using a stepped gradient from water to 75:25 methanol : water to give the title compound as a white solid (47 mg, 89 %)

^{1}H NMR (400 MHz, D_2O:CD_3CN, 1:1) δ 7.90 – 7.82 (m, 3H), 7.76 – 7.68 (m, 2H), 7.65 (at, *J* = 7.6 Hz, 1H), 7.53 (d, *J* = 7.7 Hz, 1H), 4.16 (s, 2H), 2.95 (t, *J* = 7.6 Hz, 2H), 2.32 (t, *J* = 7.5 Hz, 2H), 1.83 – 1.65 (m, 4H), 1.55 – 1.42 (m, 2H). ^{13}C NMR (100 MHz, D_2O:CD_3CN) δ 183.7,

142.1, 136.7, 136.6, 130.5, 128.9, 128.1, 127.8, 127.0 (2 carbons), 122.1, 52.4, 48.2, 38.3, 27.6, 27.0, 26.4. ESI-MS *m/z* = 304.1 $(M+H)^+$. ESI-HRMS-TOF found 304.1374, calculated 304.1366 for $(M+H)^+$. RP-HPLC R_t= 7.34 min, 99 %.

3-(3-(Thiophen-3-yl)benzylamino)propanamide (45)

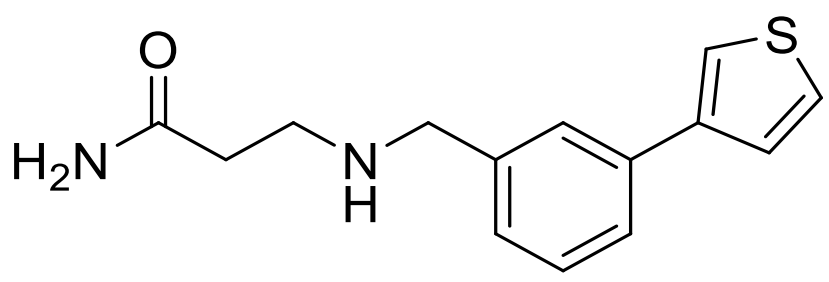

Prepared according to general procedure B with 3-(thiophen-3-yl)benzaldehyde (**S1**) (136 mg, 0.725 mmol, 1 eq.), 3-aminopropylamide hydrochloride (97 mg, 0.78 mmol, 1.1 eq.) and sodium triacetoxyborohydride (215 mg, 1.01 mmol, 1.4 eq.). Purification by column chromatography using a stepped gradient from 75:25:1 ethyl acetate : hexane : triethylamine to 85:15:1 ethyl acetate : methanol : triethylamine gave the title compound as a white solid (53 mg, 28 %).

^{1}H NMR (400 MHz, $CDCl_3$) δ 7.67 – 7.63 (m, 1H), 7.62 (dd, *J* = 2.4, 1.9 Hz, 1H), 7.58 – 7.53 (m, 1H), 7.48 – 7.44 (m, 2H), 7.35 (at, *J* = 7.6 Hz, 1H), 7.27 – 7.22 (m, 1H), 3.82 (s, 2H), 2.89 (t, *J* = 6.8 Hz, 2H), 2.46 (t, *J* = 6.8 Hz, 2H). ^{13}C NMR (100 MHz, $CDCl_3$) δ 177.3, 143.4, 140.7, 137.5, 130.1, 128.3, 127.5, 127.3, 127.2, 126.3, 121.4, 54.1, 45.8, 35.4. ESI-MS *m/z* = 261.1 $(M+H)^+$. ESI-HRMS-TOF found 261.1064, calculated 261.1056 for $(M+H)^+$. RP-HPLC R_t= 6.05 min, 98 %.

2-(3-(Thiophen-3-yl)benzylamino)ethanol (46)

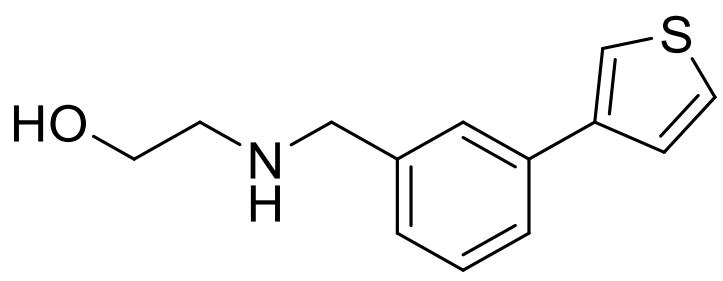

Prepared according to general procedure B with 3-(thiophen-3-yl)benzaldehyde (**S1**) (400 mg, 2.13 mmol, 1 eq.), ethanolamine (180 µL, 2.98 mmol, 1.4 eq.) and sodium triacetoxyborohydride (632 mg, 2.98 mmol, 1.4 eq.). Purification by column

chromatography using a stepped gradient from 100:1 ethyl acetate : triethylamine to 80:20:1 ethyl acetate : methanol : triethylamine gave the title compound as a clear oil (232 mg, 47 %). ^{1}H NMR (400 MHz, CD_3OD) δ 7.65 (at, *J* = 1.5 Hz, 1H), 7.60 (dd, *J* = 2.5, 1.8 Hz, 1H), 7.56 – 7.51 (m, 1H), 7.47 – 7.43 (m, 2H), 7.34 (at, *J* = 7.6 Hz, 1H), 7.27 – 7.22 (m, 1H), 3.80 (s, 2H), 3.68 (t, *J* = 5.6 Hz, 2H), 2.74 (t, *J* = 5.6 Hz, 2H). ^{13}C NMR (100 MHz, CD_3OD) δ 143.4, 141.0, 137.4, 130.0, 128.2, 127.4, 127.3, 127.2, 126.2, 121.4, 61.5, 54.3, 51.7. ESI-MS *m/z* = 234.3 $(M+H)^+$. ESI-HRMS-TOF found 234.0951, calculated 234.0947 for $(M+H)^+$. RP-HPLC R_t= 6.40 min, 98 %.

3-(3-(Thiophen-3-yl)benzylamino)propanol (47)

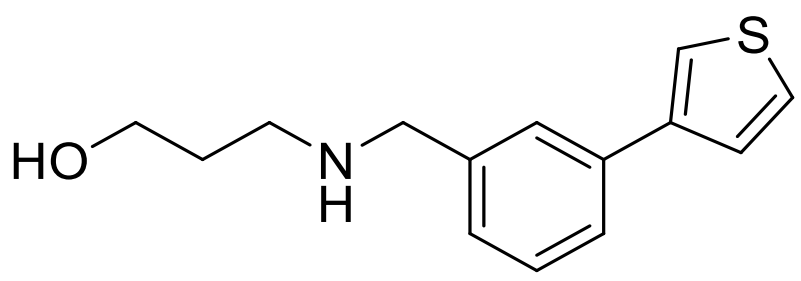

Prepared according to general procedure B with 3-(thiophen-3-yl)benzaldehyde (**S1**) (429 mg, 2.28 mmol, 1 eq.), 3-aminopropanol (236 µL, 3.19 mmol, 1.4 eq.) and sodium triacetoxyborohydride (675 mg, 3.19 mmol, 1.4 eq.). Purification by column chromatography using a stepped gradient from 100:1 ethyl acetate : triethylamine to 80:20:1 ethyl acetate : methanol : triethylamine gave the title compound as a clear oil (362 mg, 64 %).

^{1}H NMR (400 MHz, $CDCl_3$) δ 7.53 (bs, 1H), 7.50 (abdt, *J* = 7.7, 1.3 Hz, 1H), 7.46 (dd, *J* = 2.7, 1.6 Hz, 1H), 7.41 – 7.33 (m, 3H), 7.25 – 7.20 (m, 1H), 3.86 – 3.79 (m, 4H), 2.92 (t, *J* = 5.9 Hz, 2H), 1.79 – 1.70 (m, 2H). ^{13}C NMR (100 MHz, $CDCl_3$) δ 142.3, 140.2, 136.2, 129.1, 127.1, 126.5, 126.4 (2 carbons), 125.5, 120.6, 64.4, 54.1, 49.5, 31.0. ESI-MS *m/z* = 248.1 $(M+H)^+$. ESI-HRMS-TOF found 248.1112, calculated 248.1104 for $(M+H)^+$. RP-HPLC R_t= 6.34 min, 99 %.

4-(3-(Thiophen-3-yl)benzylamino)butanol (48)

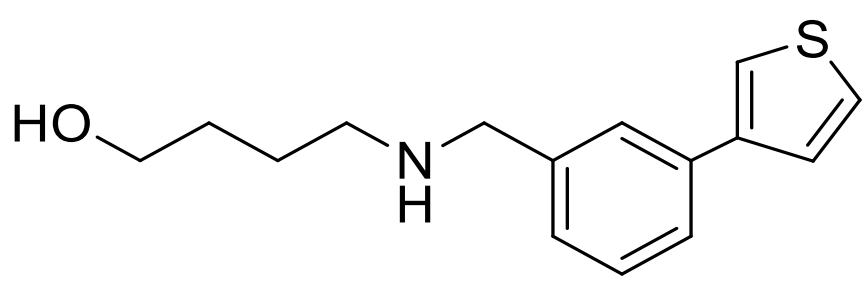

Prepared according to general procedure B with 3-(thiophen-3-yl)benzaldehyde (**S1**) (403 mg, 2.14 mmol, 1 eq.), 4-aminobutanol (276 µL, 2.99 mmol,

1.4 eq.) and sodium triacetoxyborohydride (635 mg, 2.99 mmol, 1.4 eq.). Purification by column chromatography using a stepped gradient from 100:1 ethyl acetate : triethylamine to 80:20:1 ethyl acetate : methanol : triethylamine gave the title compound as a clear oil (379 mg, 68 %).

^{1}H NMR (400 MHz, $CDCl_3$) δ 7.56 – 7.53 (m, 1H), 7.53 – 7.48 (m, 1H), 7.47 (dd, *J* = 2.8, 1.5 Hz, 1H), 7.42 – 7.33 (m, 3H), 7.27 – 7.23 (m, 1H), 3.83 (s, 2H), 3.61 (t, *J* = 4.4 Hz, 2H), 2.73 (t, *J* = 4.8 Hz, 2H), 1.75 – 1.57 (m, 4H). ^{13}C NMR (100 MHz, $CDCl_3$) δ 142.3, 140.0, 136.3, 129.2, 127.2, 126.6, 126.5, 126.4, 125.5, 120.6, 62.9, 54.1, 49.4, 32.6, 28.8. ESI-MS *m/z* = 262.2 $(M+H)^+$. ESI-HRMS-TOF found 262.1265, calculated 262.1260 for $(M+H)^+$. RP-HPLC R_t= 6.71 min, 99 %.

5-(3-(Thiophen-3-yl)benzylamino)pentanol (49)

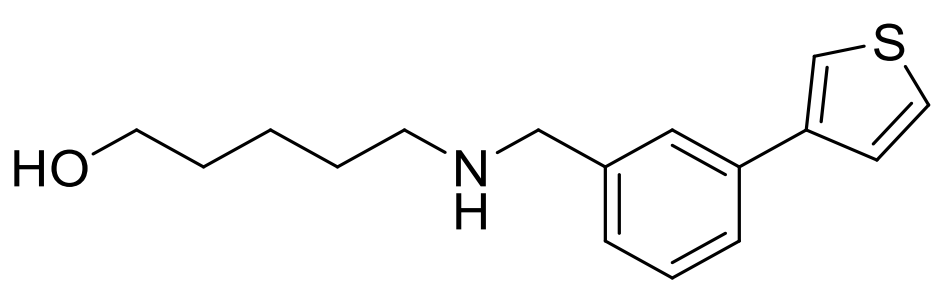

Prepared according to general procedure B with 3-(thiophen-3-yl)benzaldehyde (**S1**) (171 mg, 0.909 mmol, 1 eq.), 5-aminopentanol (131 mg, 1.27 mmol, 1.4 eq.) and sodium triacetoxyborohydride (270 mg, 1.27 mmol, 1.4 eq.). Purification by column chromatography using a stepped gradient from 100:1 ethyl acetate : triethylamine to 85:15:1 ethyl acetate : methanol : triethylamine gave the title compound as a clear oil (132 mg, 53 %).

^1H NMR (400 MHz, $CDCl_3$) δ 7.56 (bs, 1H), 7.51 – 7.45 (m, 2H), 7.42 – 7.32 (m, 3H), 7.27 – 7.22 (m, 1H), 3.83 (s, 2H), 3.64 (t, *J* = 7.4 Hz, 2H), 2.68 (t, *J* = 7.0 Hz, 2H), 1.61 – 1.52 (m, 4H), 1.49 – 1.36 (m, 2H). ^{13}C NMR (100 MHz, $CDCl_3$) δ 142.4, 141.0, 136.1, 129.0, 127.1, 126.5, 126.4, 126.3, 125.3, 120.5, 62.7, 54.2, 49.4, 32.6, 29.8, 23.5. ESI-MS *m/z* = 276.1 $(M+H)^+$. ESI-HRMS-TOF found 276.1421, calculated 276.1417 for $(M+H)^+$. RP-HPLC R_t= 7.07 min, 99 %.

_N_-Methyl-3-(_N_-Methylpyrrol-2-yl)benzylamine (50)

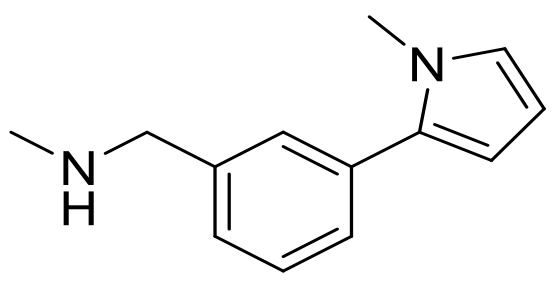

Prepared according to general procedure D with *N*-methyl-3-bromobenzylamine (**S2**) (35 µL, 0.26 mmol, 1 eq.), *N*-methylpyrrole-2-boronic acid (60 mg, 0.29 mmol, 1.125 eq.), K_3PO_4 (61 mg, 0.29 mmol, 1.125 eq.) and palladium *tetrakis*(triphenylphosphine) (15 mg, 0.01 mmol, 5 mol%). Purification by column chromatography using a stepped gradient from 100:1 ethyl acetate : triethylamine to 80:20:1 ethyl acetate : methanol : triethylamine gave the title compound as a white solid (21 mg, 41%). ^1H NMR (400 MHz, CD_3OD) δ 7.39 – 7.33 (m, 2H), 7.30 (adt, *J* = 7.7, 1.5 Hz, 1H), 7.24 (adt, *J* = 7.4, 1.5 Hz, 1H), 6.72 (dd, *J* = 2.6, 1.9 Hz, 1H), 6.14 (dd, *J* = 3.6, 1.8 Hz, 1H), 6.09 (dd, *J* = 3.6, 2.7 Hz, 1H), 3.74 (s, 2H), 3.64 (s, 3H), 2.40 (s, 3H). ^{13}C NMR (100 MHz, CD_3OD) δ 140.2, 135.5, 135.3, 129.7, 129.6, 128.4, 127.8, 124.9, 109.6, 108.6, 56.2, 35.4, 35.3. ESI-MS *m/z* = 170.2 (100%, $M-CH_3NH)+$; 201.2 (10%, $M+H)^+$. ESI-HRMS-TOF found 201.1389, calculated 201.1386 for $(M+H)^+$. RP-HPLC R_t= 5.69 min, 96 %.

N-Methyl-3-(furan-3-yl)benzylamine (51)

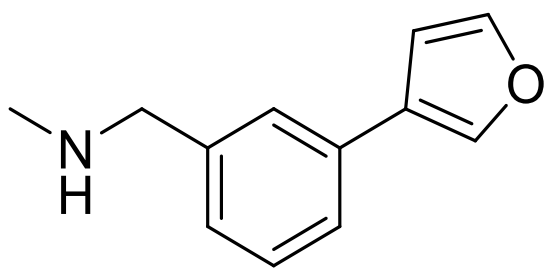

Prepared according to general procedure D with *N*-methyl-3-bromobenzylamine (**S2**) (35 µL, 0.26 mmol, 1 eq.), furan-3-boronic acid (32 mg, 0.29 mmol, 1.125 eq.), K_3PO_4 (61 mg, 0.29 mmol, 1.125 eq.) and palladium *tetrakis*(triphenylphosphine) (15 mg, 0.01 mmol, 5 mol%). Purification by column chromatography using a stepped gradient from 100:1 ethyl acetate : triethylamine to 80:20:1 ethyl acetate : methanol : triethylamine gave the title compound as a white solid (25 mg, 52 %).

^{1}H NMR (400 MHz, CD_3OD) δ 7.89 (dd, *J* = 1.5, 0.9 Hz, 1H), 7.55 – 7.54 (at, *J* =1.6 Hz, 1H), 7.53 (dd, *J* = 1.7, 1.3 Hz, 1H), 7.47 – 7.42 (adt, *J* = 7.6, 1.2 Hz, 1H), 7.32 (at, *J* = 7.6 Hz, 1H), 7.20 (ddd, *J* = 7.6, 1.6, 1.2 Hz, 1H), 6.81 (dd, *J* = 1.9, 0.9 Hz, 1H), 3.71 (s, 2H), 2.38 (s, 3H). ^{13}C NMR (100 MHz, CD_3OD) δ 145.0, 140.8, 140.1, 134.1, 130.0, 128.1, 127.7, 126.9, 125.7, 109.6, 56.3, 35.5. ESI-MS 157.2 (100%, M-CH_3NH)$^+$; 188.2 (10%, M+H)$^+$. ESI-HRMS-TOF found 188.1076, calculated 188.1070 for $(M+H)^+$. RP-HPLC R_t= 5.67 min, >99 %.

N-Methyl-3-(furan-2-yl)benzylamine (52)

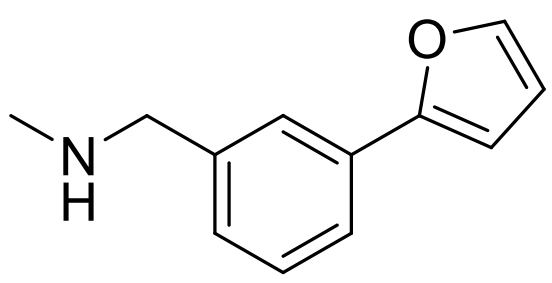

Prepared according to general procedure D with *N*-methyl-3-bromobenzylamine (**S2**) (35 µL, 0.26 mmol, 1 eq.), furan-2-boronic acid (32 mg, 0.29 mmol, 1.125 eq.), K_3PO_4 (61 mg, 0.29 mmol, 1.125 eq.) and palladium *tetrakis*(triphenylphosphine) (15 mg, 0.01 mmol, 5 mol%). Purification by column chromatography using a stepped gradient from 100:1 ethyl acetate : triethylamine to 90:10:1 ethyl acetate : methanol : triethylamine gave the title compound as a yellow oil (25 mg, 52 %).

^{1}H NMR (400 MHz, CD_3OD) δ 7.67 (bs, 1H), 7.59 (adt, *J* = 8.0, 1.2 Hz, 1H), 7.54 (dd, *J* = 1.8, 0.7 Hz, 1H), 7.34 (at, *J* = 7.7 Hz, 1H), 7.24 – 7.19 (m, 1H), 6.76 (dd, *J* = 3.4, 0.7 Hz, 1H), 6.50 (dd, *J* = 3.4, 1.8 Hz, 1H), 3.71 (s, 2H), 2.37 (s, 3H). ^{13}C NMR (100 MHz, CD_3OD) δ 155.2, 143.4, 140.9, 132.5, 129.9, 128.5, 124.8, 123.6, 112.7, 106.2, 56.3, 35.5. ESI-MS *m/z* = 157.1 (100 %, M-$CH_3NH)^+$; 188.1 (4 %, M+H)$^+$. ESI-HRMS-TOF found 188.1077, calculated 188.1070 for (M+H)$^+$. RP-HPLC R_t= 5.94 min, 99 %.

***N*-Methyl-3-(3,5-dimethylisoxazol-4-yl)benzylamine (53)**

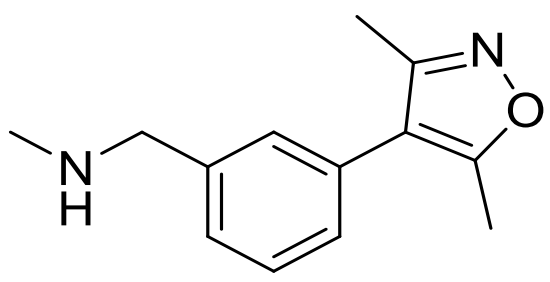

Prepared according to general procedure D with *N*-methyl-3-bromobenzylamine (**S2**) (35 µL, 0.26 mmol, 1 eq.), 3,5-dimethylisoxazole4-boronic acid pinacol ester (64 mg, 0.29 mmol, 1.125 eq.) and K_3PO_4 (61 mg, 0.29 mmol, 1.125 eq.) and palladium *tetrakis*(triphenylphosphine) (15 mg, 0.01 mmol, 5 mol%). Purification by column chromatography using a stepped gradient from 100:1 ethyl acetate : triethylamine to 80:20:1 ethyl acetate : methanol : triethylamine gave the title compound as a white solid (18 mg, 33%). ^{1}H NMR (400 MHz, CD_3OD) δ 7.45 (at, *J* = 7.6 Hz, 1H), 7.38 – 7.34 (m, 1H), 7.33 – 7.30 (m, 1H), 7.27 – 7.22 (m, 1H), 3.78 (s, 2H), 2.41 (s, 3H), 2.40 (s, 3H), 2.25 (s, 3H). ^{13}C NMR (100 MHz, CD_3OD) δ 167.0, 160.0, 140.9, 131.7, 130.4, 130.1, 129.2, 129.1, 117.9, 56.0, 35.4, 11.4, 10.7. ESI-MS *m/z* = 186.1 (100 %, M-$CH_3NH)^+$; 217.1 (14 %, M+H)$^+$. ESI-HRMS-TOF found 217.1343, calculated 217.1335 for (M+H)$^+$. RP-HPLC R_t= 5.30 min, 97 %.

***N*-Methyl-3-(pyrazol-4-yl)benzylamine (54)**

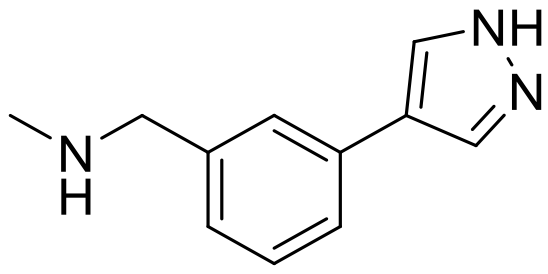

Prepared according to general procedure D with *N*-methyl-3-bromobenzylamine (**S2**) (35 µL, 0.26 mmol, 1 eq.), 1-Boc-pyrazole-4-boronic acid (61 mg, 0.29 mmol, 1.125 eq.), K_3PO_4 (61 mg, 0.29 mmol, 1.125 eq.) and palladium *tetrakis*(triphenylphosphine) (15 mg, 0.01 mmol, 5 mol%). Purification by column chromatography using a stepped gradient from 100:1 ethyl acetate : triethylamine to 80:20:1 ethyl acetate : methanol : triethylamine gave the title compound as a white solid (7 mg, 14 %).

^{1}H NMR (400 MHz, CD_3OD) δ 7.97 (s, 2H), 7.61 (at, *J* = 1.5 Hz, 1H), 7.54 (adt, *J* = 7.6, 1.6 Hz, 1H), 7.36 (at, *J* = 7.7 Hz, 1H), 7.21 (adt, *J* = 7.6, 1.6 Hz, 1H), 3.86 (s, 2H), 2.49 (s, 3H).

^{13}C NMR (100 MHz, $CDCl_3$) δ 138.9, 133.1, 131.3, 129.2, 127.0, 125.9, 125.0, 122.4, 55.4, 35.1. ESI-MS *m/z* = 157.2 (100 %, M-CH_3NH)$^+$; 188.2 (10 %, M+H)$^+$. ESI-HRMS-TOF found 188.1182, calculated 188.1182 for (M+H)$^+$. RP-HPLC R_t= 3.61 min, 96 %.

N-Methyl-3-(pyridin-4-yl)benzylamine (55)

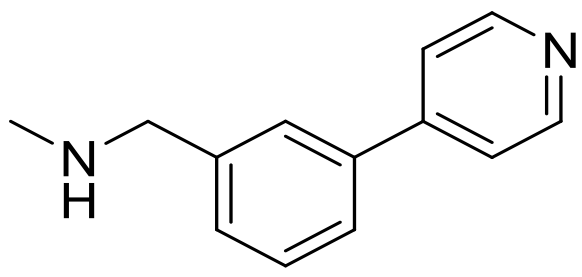

Prepared according to general procedure D with *N*-methyl-3-bromobenzylamine (**S2**) (35 µL, 0.26 mmol, 1 eq.), 4-pyridine boronic acid (59 mg, 0.29 mmol, 1.125 eq.), K_3PO_4 (61 mg, 0.29 mmol, 1.125 eq.) and palladium *tetrakis*(triphenylphosphine) (15 mg, 0.01 mmol, 5 mol%). Purification by column chromatography using a stepped gradient from 100:1 ethyl acetate : triethylamine to 80:20:1 ethyl acetate : methanol : triethylamine gave the title compound as a white solid (29 mg, 58 %).

^{1}H NMR (400 MHz, CD_3OD) δ 8.60 – 8.54 (m, 2H), 7.79 – 7.59 (m, 4H), 7.52 – 7.44 (m, 2H), 3.84 (s, 2H), 2.44 (s, 3H). ^{13}C NMR (100 MHz, CD_3OD) δ 150.6, 150.5, 140.9, 139.1, 130.9,

130.5, 128.4, 127.2, 123.2, 55.9, 35.3. ESI-MS *m/z* = 199.2 $(M+H)^+$. ESI-HRMS-TOF found 199.1236, calculated199.1230 for $(M+H)^+$. RP-HPLC R_t= 2.21 min, 97 %.

***N*-Methyl-3-(p-phenol)benzylamine (56)**

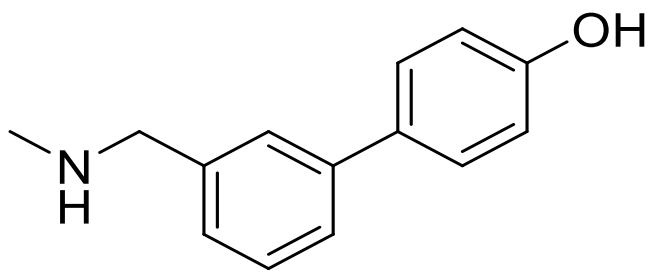

Prepared according to general procedure D with *N*-methyl-3-bromobenzylamine (**S2**) (35 µL, 0.26 mmol, 1 eq.), p-hydroxyphenyl boronic acid (40 mg, 0.29 mmol, 1.125 eq.), K_3PO_4 (61 mg, 0.29 mmol, 1.125 eq.) and palladium *tetrakis*(triphenylphosphine) (15 mg, 0.01 mmol, 5 mol%). Purification by column chromatography using a stepped gradient from 100:1 ethyl acetate : triethylamine to 80:20:1 ethyl acetate : methanol : triethylamine gave the title compound as a white solid (21 mg, 39%). ^{1}H NMR (400 MHz, CD_3OD) δ 7.53 (at, *J* = 1.5 Hz, 1H), 7.49 – 7.44 (m, 3H), 7.36 (at, *J* = 7.6 Hz, 1H), 7.23 (d, *J* = 7.6 Hz, 1H), 6.88 – 6.82 (m, 2H), 3.78 (s, 2H), 2.42 (s, 3H). ^{13}C NMR (100 MHz, CD_3OD) δ 158.4, 142.7, 140.0, 133.5, 129.9, 129.1, 127.8, 127.7, 126.6, 116.7, 56.2, 35.3. ESI-MS *m/z* = 183.2 (100%, $M-CH_3NH)^+$; 214.2 (10%, $M+H)^+$. ESI-HRMS-TOF found 214.1236, calculated 214.1226 for $(M+H)^+$. RP-HPLC R_t= 5.27 min, 98 %.

***N*-Methyl-3-(m-phenol)benzylamine (57)**

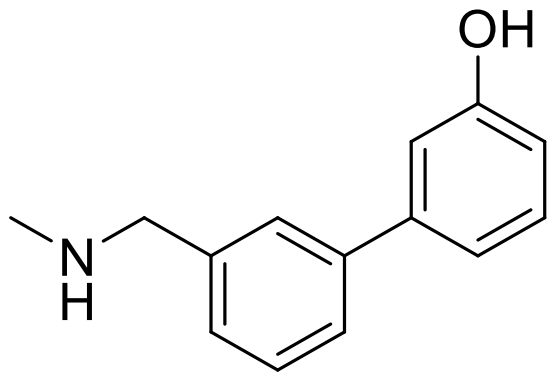

Prepared according to general procedure D with *N*-methyl-3-bromobenzylamine (**S2**) (35 µL, 0.26 mmol, 1 eq.), *m*-hydroxyphenyl boronic acid (40 mg, 0.29 mmol, 1.125 eq.), K_3PO_4 (61 mg, 0.29 mmol, 1.125 eq.) and palladium *tetrakis*(triphenylphosphine) (15 mg, 0.01 mmol, 5 mol%). Purification by column

chromatography using a stepped gradient from 100:1 ethyl acetate : triethylamine to 80:20:1 ethyl acetate : methanol : triethylamine gave the title compound as a white solid (14 mg, 26%). ^{1}H NMR (400 MHz, CD_3OD) δ 7.46 (bs, 1H), 7.38 (bd, *J* = 7.8 Hz, 1H), 7.28 (at, *J* = 7.6 Hz, 1H), 7.19 (bd, *J* = 7.6 Hz, 1H), 7.13 (at, *J* = 7.9 Hz, 1H), 6.98 (ddd, *J* = 7.7, 1.5, 1.0 Hz, 1H), 6.94 (at, *J* = 2.0 Hz, 1H), 6.66 (ddd, *J* = 8.1, 2.4, 0.9 Hz, 1H), 3.68 (bs, 2H), 2.31 (bs, 3H). ^{13}C NMR (100 MHz, CD_3OD) δ 159.0, 143.7, 142.8, 140.3, 130.8, 129.9, 128.6, 128.3, 127.0, 119.3, 115.4, 114.9, 56.2, 35.4. ESI-MS *m/z* = 183.1 (100 %, M-$CH_3NH)^+$; 214.2 (10 %, $M+H)^+$. ESI-HRMS-TOF found 214.1223, calculated 214.1226 for $(M+H)^+$. RP-HPLC R_t= 5.71 min, 99 %.

***N*-Methyl-3-(3-trifluoromethylphenyl)benzylamine (58)**

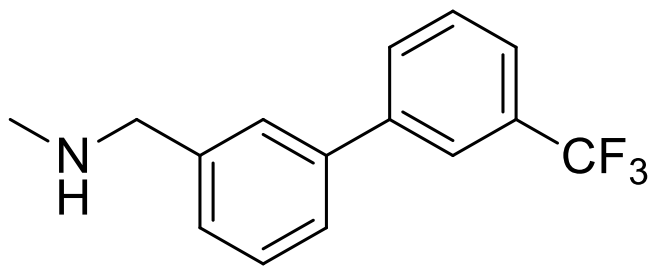

Prepared according to general procedure D with *N*-methyl-3-bromobenzylamine (**S2**) (50 µL, 0.37 mmol, 1 eq.), 3-(trifluoromethyl)phenyl boronic acid (78 mg, 0.41 mmol, 1.125 eq.), K_3PO_4 (87 mg, 0.41 mmol, 1.125 eq.) and palladium *tetrakis*(triphenylphosphine) (21 mg, 0.02 mmol, 5 mol%). Purification by column chromatography using a stepped gradient from 100:1 ethyl acetate : triethylamine to 80:20:1 ethyl acetate : methanol : triethylamine gave the title compound as a light yellow oil (26 mg, 27 %).

^{1}H NMR (400 MHz, $CDCl_3$) δ 7.76 (bs, 1H), 7.70 (bd, *J* = 7.6 Hz, 1H), 7.54 – 7.43 (m, 3H), 7.41 (adt, *J* = 7.6, 1.5 Hz, 1H), 7.35 (at, *J* = 7.5 Hz, 1H), 7.28 (bd, *J* = 7.5 Hz, 1H), 3.76 (s, 2H), 2.42 (s, 3H). ^{13}C NMR (100 MHz, $CDCl_3$) δ 142.1, 140.9, 140.1, 131.3 (q, *J* = 32.1 Hz), 130.6, 129.3, 129.2, 128.1, 127.2, 126.1, 124.2 – 123.9 (m, 2 carbons), 123.0 (q, *J* = 272.5 Hz), 56.1, 36.1. ESI-MS *m/z* = 235.1 (100 %, M-$CH_3NH)^+$, found 266.2 (67 %, $M+H)^+$. ESI-HRMS-TOF found 266.1154, calculated 266.1151 for $(M+H)^+$. RP-HPLC R_t= 8.86 min, 97 %.

N-Methyl-6-(thiophen-3-yl)-2-picolylamine (59)

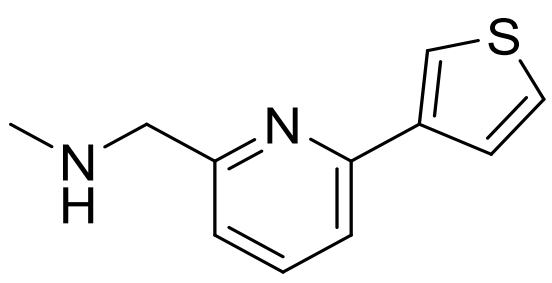

Prepared according to general procedure B with 6-(thiophen-3-yl)picolinaldehyde (**S13**) (50 mg, 0.26 mmol, 1 eq.), methylamine hydrochloride (27 mg, 0.40 mmol, 1.4 eq.) and sodium triacetoxyborohydride (84 mg, 0.340 mmol, 1.4 eq.). Purification by column chromatography using a stepped gradient from 100:1 ethyl acetate: triethylamine to 80:20:1 ethyl acetate : methanol : triethylamine gave the title compound as a clear oil (33 mg, 61 %).

^{1}H NMR (400 MHz, CD_3OD) δ 8.03 (dd, *J* = 3.0, 1.3 Hz, 1H), 7.76 – 7.71 (m, 2H), 7.65 – 7.60 (m, 1H), 7.47 (dd, *J* = 5.1, 3.0 Hz, 1H), 7.23 – 7.19 (m, 1H), 3.83 (s, 2H), 2.44 (s, 3H). ^{13}C NMR (100 MHz, CD_3OD) δ 159.5, 154.6, 143.4, 138.8, 127.4, 127.2, 124.9, 121.7, 120.0, 57.1, 35.7. ESI-MS *m/z* = 205.1 $(M+H)^+$. ESI-HRMS-TOF found 205.0784, calculated 205.0794 for $(M+H)^+$. RP-HPLC R_t= 5.16 min, 98 %.

N-Methyl-6-(thiophen-3-yl)nicotinamine (60)

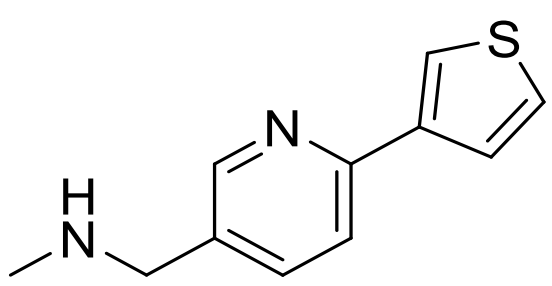

Prepared according to general procedure B with 2-(thiophen-3-yl)nicotinaldehyde (**S14**) (26 mg, 0.14 mmol, 1 eq.), methylamine hydrochloride (14 mg, 0.21 mmol, 1.4 eq.) and sodium triacetoxyborohydride (45 mg, 0.21 mmol, 1.4 eq.). Purification by column chromatography using a stepped gradient from 100:1 ethyl acetate : triethylamine to 80:20:1 ethyl acetate : methanol : triethylamine gave the title compound as a white solid (12 mg, 40 %).

^{1}H NMR (400 MHz, CD_3OD) δ 8.52 – 8.49 (m, 1H), 7.99 (dd, *J* = 3.0, 1.3 Hz, 1H), 7.83 (dd, *J* = 8.2, 2.2 Hz, 1H), 7.79 (dd, *J* = 8.2, 0.9 Hz, 1H), 7.68 (dd, *J* = 5.1, 1.3 Hz, 1H), 7.51 (dd, *J* = 5.1, 3.0 Hz, 1H), 3.79 (s, 2H), 2.44 (s, 3H). ^{13}C NMR (100 MHz, CD_3OD) δ 154.1, 150.4, 142.6, 139.2, 133.9, 127.6, 127.3, 125.0, 121.9, 53.0, 35.3. ESI-MS *m/z* = 205.2 $(M+H)^+$. ESI-HRMS-TOF found 205.0789, calculated 205.0794 for $(M+H)^+$. RP-HPLC R_t= 2.94 min, 96 %.

_N_-Methyl-5-(thiophen-3-yl)-2-picolylamine (61)

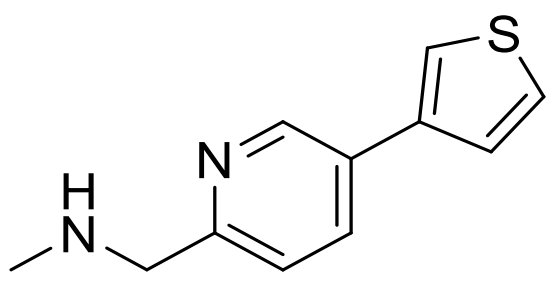

Prepared according to general procedure B with 5-(thiophen-3-yl)picolinaldehyde (**S15**) (47 mg, 0.25 mmol. 1 eq.), methylamine hydrochloride (25 mg, 0.37 mmol, 1.4 eq.) and sodium triacetoxyborohydride (79 mg, 0.37 mmol, 1.4 eq.). Purification by column chromatography using a stepped gradient from 100:1 ethyl acetate: triethylamine to 80:20:1 ethyl acetate : methanol : triethylamine gave the title compound as a white solid (33 mg, 65 %).

^{1}H NMR (400 MHz, CD_3OD) δ 8.79 (d, *J* = 1.6 Hz, 1H), 8.04 (dd, *J* = 8.1, 2.3 Hz, 1H), 7.75 (dd, *J* = 2.9, 1.4 Hz, 1H), 7.53 (dd, *J* = 5.1, 2.9 Hz, 1H), 7.49 (dd, *J* = 5.1, 1.4 Hz, 1H), 7.44 (d, *J* = 8.1 Hz, 1H), 3.83 (s, 2H), 2.41 (s, 3H). ^{13}C NMR (100 MHz, CD_3OD) δ 158.2, 147.4, 139.4, 135.9, 132.0, 128.2, 126.7, 124.1, 122.8, 56.7, 35.6. ESI-MS *m/z* = 205.2 $(M+H)^+$. ESI-HRMS-TOF found 205.0788, calculated 205.0794 for $(M+H)^+$. RP-HPLC R_t= 5.13 min, 98 %.

_N_-Methyl-5-(thiophen-3-yl)nicotinamine (62)

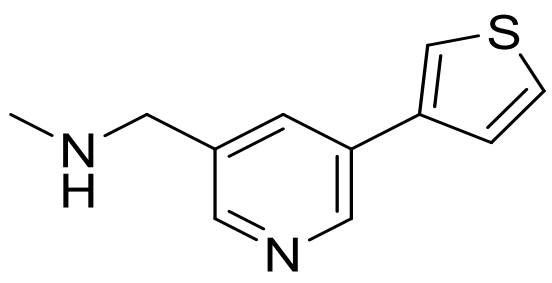
Prepared according to general procedure B with 5-(thiophen-3-yl)nicotinaldehyde (**S12**) (36 mg, 0.19 mmol, 1 eq.), methylamine hydrochloride (19 mg, 0.29 mmol, 1.4 eq.) and sodium triacetoxyborohydride (61 mg, 0.29 mmol, 1.4 eq.). Purification by column chromatography using a stepped gradient from 75:25:1 ethyl acetate : hexane : triethylamine to 80:20:1 ethyl acetate : methanol : triethylamine gave the title compound as a clear oil (18 mg, 47 %).

^{1}H NMR (400 MHz, CD_3OD) δ 8.76 (d, *J* = 2.1 Hz, 1H), 8.41 (d, *J* = 2.0 Hz, 1H), 8.10 (at, *J* = 2.1 Hz, 1H), 7.81 (dd, *J* = 2.9, 1.4 Hz, 1H), 7.56 (dd, *J* = 5.1, 2.9 Hz, 1H), 7.53 (dd, *J* = 5.1, 1.4 Hz, 1H), 3.80 (s, 2H), 2.42 (s, 3H). ^{13}C NMR (100 MHz, CD_3OD) δ 148.5, 146.6, 139.3, 136.8, 135.7, 133.4, 128.3, 126.8, 123.2, 53.3, 35.6. ESI-MS *m/z* = 205.2 $(M+H)^+$. ESI-HRMS-TOF found 205.0785, calculated 205.0794 for $(M+H)^+$. RP-HPLC R_t= 2.74 min, 99 %.

***N*-Methyl-2-(thiophen-3-yl)isonicotinamine (63)**

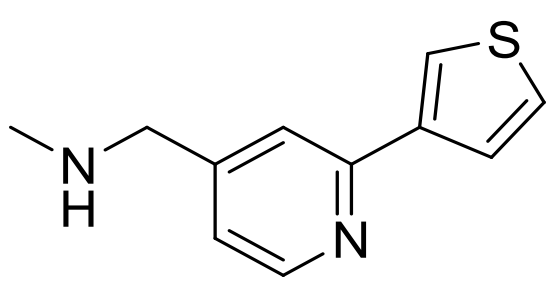
Prepared according to general procedure B with 2-(thiophene-3-yl)isonicotinaldehyde (**S16**) (34 mg, 0.18 mmol, 1 eq.), methylamine hydrochloride (18 mg, 0.27 mmol, 1.4 eq.) and sodium triacetoxyborohydride (58 mg, 0.27 mmol, 1.4 eq.). Purification by column chromatography using a stepped gradient from 50:50:1 ethyl acetate : hexane : triethylamine to 80:20:1 ethyl acetate : methanol : triethylamine gave the title compound as a clear oil (17 mg, 45 %).

^{1}H NMR (400 MHz, CD_3OD) δ 8.47 (dd, *J* = 5.1, 0.7 Hz, 1H), 8.00 (dd, *J* = 3.0, 1.3 Hz, 1H), 7.79 (dd, *J* = 1.5, 0.7 Hz, 1H), 7.69 (dd, *J* = 5.1, 1.3 Hz, 1H), 7.51 (dd, *J* = 5.1, 3.0 Hz, 1H),

7.26 (dd, J = 5.2, 1.6 Hz, 1H), 3.79 (s, 2H), 2.42 (s, 3H). ^{13}C NMR (100 MHz, CD_3OD) δ 155.0, 151.5, 150.2, 142.8, 127.6, 127.3, 125.1, 122.9, 121.6, 55.1, 35.7. ESI-MS m/z = 205.1 $(M+H)^+$. ESI-HRMS-TOF found 205.0784, calculated 205.0794 for $(M+H)^+$. RP-HPLC R_t= 2.43 min, 97 %.

***N*-Methyl-1-(4-phenylthiophen-2-yl)methanamine (64)**

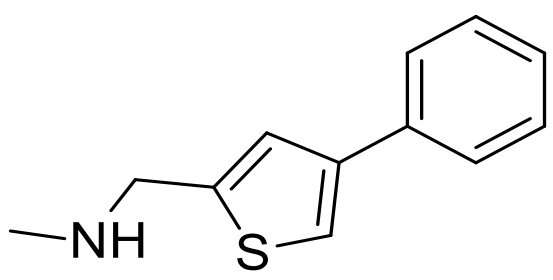

Prepared according to general procedure B with 2-formyl-4-phenylthiophene (**S17**) (48 mg, 0.26 mmol, 1 eq.), methylamine hydrochloride (26 mg, 0.38 mmol, 1.5 eq.) and sodium triacetoxyborohydride (82 mg, 0.38 mmol, 1.5 eq.). Purification by column chromatography using a stepped gradient from 100:1 hexane : triethylamine to 60:40:1 hexane : ethyl acetate: triethylamine gave the title compound as a clear oil (14 mg, 26 %).

^{1}H NMR (400 MHz, $CDCl_3$) δ 7.59 – 7.55 (m, 2H), 7.41 – 7.36 (m, 2H), 7.33 (d, J = 1.5 Hz, 1H), 7.30 – 7.25 (m, 1H), 7.24 – 7.22 (m, 1H), 3.97 (ad, J = 0.8 Hz, 2H), 2.51 (s, 3H). ^{13}C NMR (100 MHz, $CDCl_3$) δ 144.9, 141.9, 136.1, 128.9, 127.2, 126.4, 124.4, 119.4, 50.8, 36.0. ESI-HRMS-TOF found 204.0836, calculated 204.0841 for $(M+H)^+$. RP-HPLC R_t= 6.39 min, 95 %.

***N*-Methyl-1-(5-phenylthiophen-2-yl)methanamine (65)**

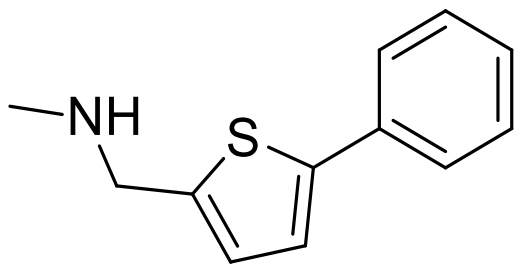 Prepared according to general procedure B with 2-formyl-5-phenylthiophene (**S18**) (41 mg, 0.26 mmol, 1 eq.), methylamine hydrochloride (22 mg, 0.33 mmol, 1.5 eq.) and sodium triacetoxyborohydride (69 mg, 0.33 mmol, 1.5 eq.). Purification by column chromatography using a stepped gradient from 100:1 hexane : triethylamine to 80:20:1 hexane : ethyl acetate: triethylamine gave the title compound as a clear oil (11 mg, 25 %).

^{1}H NMR (400 MHz, $CDCl_3$) δ 7.60 – 7.55 (m, 2H), 7.39 – 7.33 (m, 2H), 7.26 (tt, *J* = 6.6, 1.2 Hz, 1H), 7.16 (d, *J* = 3.6 Hz, 1H), 6.89 (dt, *J* = 3.6, 0.8 Hz, 1H), 3.95 (d, *J* = 0.7 Hz, 2H), 2.51 (s, 3H). ^{13}C NMR (100 MHz, $CDCl_3$) δ 143.6, 143.5, 134.7, 129.0, 127.4, 126.1, 125.8, 122.7, 50.8, 35.9. ESI-HRMS-TOF found 173.0413, calculated 173.0419 for $(M\text{-}CH_3NH)^+$. RP-HPLC R_t= 6.88 min, 98 %.

3-(Thiophen-3-yl)benzamide (66)

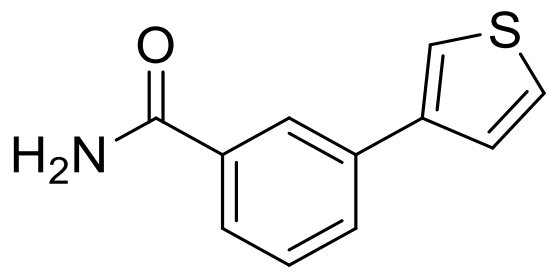 Prepared according to general procedure A with 3-iodobenzamide (**S19**) (64 mg, 0.26 mmol, 1 eq.), thiophene-3-boronic acid (37 mg, 0.22 mmol, 1.125 eq.), K_3PO_4 (62 mg, 0.29 mmol, 1.125 eq.) and palladium *tetrakis*(triphenylphosphine) (15 mg, 0.01 mmol, 5 mol%). Purification by column chromatography using a stepped gradient from 70:30 hexane : ethyl acetate to 30:70 ethyl acetate : hexane gave the title compound as a white solid (27 mg, 51 %).

^{1}H NMR (400 MHz, CD_3OD) δ 8.18 (atd, *J* = 1.8, 0.4 Hz, 1H), 7.85 (ddd, *J* = 7.8, 1.8, 1.1 Hz, 1H), 7.79 (ddd, *J* = 7.8, 1.8, 1.1 Hz, 1H), 7.72 (dd, *J* = 2.7, 1.6 Hz, 1H), 7.54 – 7.47 (m, 3H). ^{13}C NMR (100 MHz, CD_3OD) δ 172.3, 142.6, 137.6, 135.6, 130.6, 130.1, 127.6, 127.2, 127.1,

126.5, 122.1. ESI-MS *m/z* = 204.1 $(M+H)^+$. ESI-HRMS-TOF found 204.0472, calculated 204.0478 for $(M+H)^+$. RP-HPLC R_t= 8.18 min, 99 %.

N-Methyl-3-(thiophen-3-yl)benzamide (67)

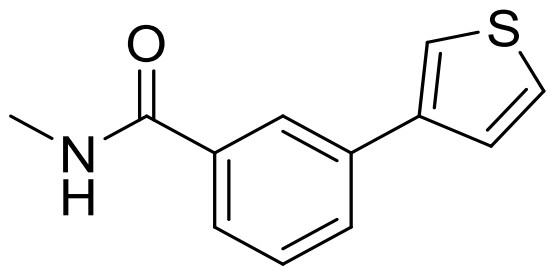

Prepared according to general procedure A with *N*-methyl-3-iodobenzamide (**S20)** (107 mg, 0.410 mmol, 1 eq.), thiophene-3-boronic acid (59 mg, 0.46 mmol, 1.125 eq.), K_3PO_4 (98 mg, 0.46 mmol, 1.125 eq.) and palladium *tetrakis*(triphenylphosphine) (24 mg, 0.02 mmol, 5 mol%). Purification by column chromatography using a stepped gradient from 75:25 hexane : ethyl acetate to 75:25 ethyl acetate : hexane gave the title compound as a white solid (73 mg, 82 %).

^{1}H NMR (400 MHz, $CDCl_3$) δ 8.01 (at, *J* = 1.6 Hz, 1H), 7.70 (ddd, *J* = 7.7, 1.8, 1.1 Hz, 1H), 7.63 (ddd, *J* = 7.7, 1.7, 1.2 Hz, 1H), 7.50 (dd, *J* = 2.6, 1.7 Hz, 1H), 7.46 – 7.37 (m, 3H), 6.34 (bs, 1H), 3.02 (d, *J* = 4.9 Hz, 3H). ^{13}C NMR (100 MHz, $CDCl_3$) δ 168.4, 141.5, 136.4, 135.4, 129.4, 129.1, 126.7, 126.3, 125.3, 125.3, 121.2, 27.0. ESI-MS *m/z* = 218.1 $(M+H)^+$. ESI-HRMS-TOF found 218.0632, calculated 218.0634 for $(M+H)^+$. RP-HPLC R_t= 8.47 min, 95 %.

N-Cyclohexyl-3-(thiophen-3-yl)benzamide (68)

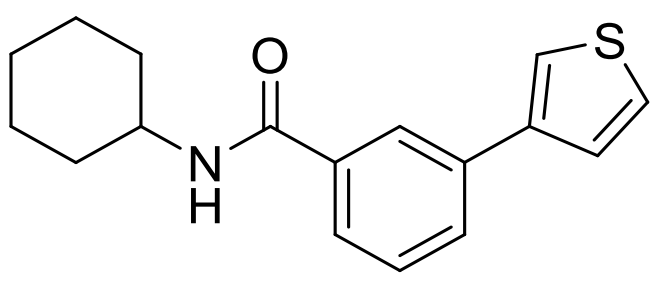

Prepared according to general procedure A with *N*-cyclohexyl-3-iodobenzamide (**S21**) (150 mg, 0.456 mmol, 1 eq.), thiophene-3-boronic acid (66 mg, 0.51 mmol, 1.125 eq.), K_3PO_4 (109 mg, 0.513 mmol, 1.125 eq.) and palladium *tetrakis*(triphenylphosphine) (26 mg, 0.02 mmol, 5 mol%). Purification by column

chromatography using a stepped gradient from hexane to 75:25 hexane : ethyl acetate gave the title compound as a white solid (73 mg, 56 %).

^{1}H NMR (400 MHz, $CDCl_3$) δ 8.00 (at, *J* = 1.7 Hz, 1H), 7.69 (ddd, *J* = 7.7, 1.8, 1.1 Hz, 1H), 7.66 – 7.59 (m, 1H), 7.50 (dd, *J* = 2.7, 1.6 Hz, 1H), 7.46 – 7.37 (m, 3H), 6.11 (bd, *J* = 7.4 Hz, 1H), 4.08 – 3.89 (m, 1H), 2.15 – 1.97 (m, 2H), 1.81 – 1.70 (m, 2H), 1.70 – 1.61 (m, 1H), 1.49 – 1.35 (m, 2H), 1.32 – 1.13 (m, 3H). ^{13}C NMR (100 MHz, $CDCl_3$) δ 166.7, 141.6, 136.4, 135.9, 129.3, 129.1, 126.6, 126.4, 125.3, 125.3, 121.2, 48.9, 33.4, 25.7, 25.1. ESI-MS *m/z* = 286.1 $(M+H)^+$. ESI-HRMS-TOF found 286.1262, calculated 286.1260 for $(M+H)^+$. RP-HPLC R_t= 11.13 min, 99 %.

_N_-Acetyl-3-(thiophen-3-yl)benzylamine (69)

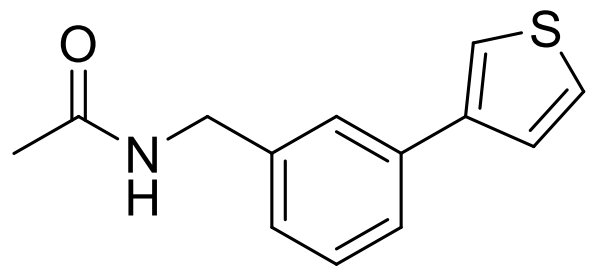

3-(Thiophen-3-yl)benzylamine hydrochloride (**32**) (92 mg, 0.41 mmol, 1 eq.), acetic anhydride (44 µL, 0.45 mmol, 1.1 eq.) and triethylamine (114 µL, 0.815 mmol, 2 eq.) was added sequentially to chloroform (5 mL) and the reaction stirred at room temperature for 16 hours. The reaction mixture was then evaporated to dryness and diluted with HCl (aq., 1 M, 10 mL) and extracted with ethyl acetate (3 × 10 mL). The combined organic layers were dried over anhydrous magnesium sulfate, filtered, and evaporated to give an oil. Purification by column chromatography using a stepped gradient from 70:30 ethyl acetate : hexane to ethyl acetate to give the title compound as a white solid (79 mg, 70 %).

^{1}H NMR (400 MHz, $CDCl_3$) δ 7.53 – 7.49 (m, 2H), 7.45 (dd, *J* = 2.8, 1.5 Hz, 1H), 7.41 – 7.34 (m, 3H), 7.23 – 7.19 (m, 1H), 5.79 (bs, 1H), 4.47 (d, *J* = 5.7 Hz, 2H), 2.04 (s, 3H). ^{13}C NMR (100 MHz, $CDCl_3$) δ 170.0, 142.1, 139.0, 136.5, 129.4, 126.8, 126.5, 126.4, 126.2, 125.9, 120.8, 44.0, 23.5. ESI-MS *m/z* = 232.1 $(M+H)^+$. ESI-HRMS-TOF 232.0792 found, calculated 232.0791 for $(M+H)^+$. RP-HPLC R_t= 8.90 min, >99 %.

N-Acetyl-N-methyl-3-(thiophen-3-yl)benzylamine (70)

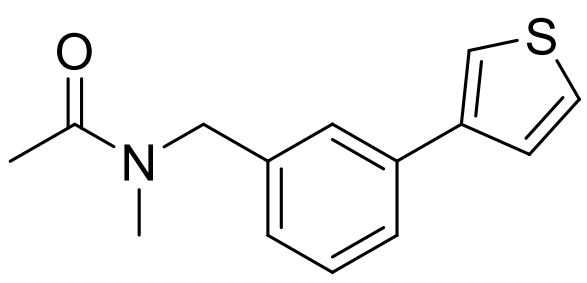

N-Methyl-3-(thiophen-3-yl)benzylamine hydrochloride (**1**) (100 mg, 0.42 mmol, 1 eq.), acetic anhydride (44 µL, 0.46 mmol, 1.1 eq.) and triethylamine (117 µL, 0.84 mmol, 2 eq.) was added sequentially to chloroform (5 mL) and the reaction stirred at room temperature for 16 hours. The reaction mixture was then evaporated to dryness and diluted with HCl (aq., 1 M, 10 mL) and extracted with ethyl acetate (3 × 10 mL). The combined organic layers were dried over anhydrous magnesium sulfate, filtered, and evaporated to give an oil. Purification by column chromatography using a stepped gradient from 50:50 ethyl acetate: hexane to ethyl acetate gave the title compound as a white solid (71 mg, 69 %). ^{1}H NMR was consistent with the presence of amide rotamers.

^{1}H NMR (400 MHz, $CDCl_3$) δ 7.54 – 7.43 (m, 2.6H), 7.42 – 7.32 (m, 3.4H), 7.16 (bd, J = 0.6 Hz, 1H), 7.09 (bd, J = 7.6 Hz, 0.4H), 4.62 (s, 1.2H), 4.54 (s, 0.8H), 2.97 (s, 1.3H), 2.93 (s, 1.7H), 2.17 (s, 1.3H), 2.16 (s, 1.7H). ^{13}C NMR (100 MHz, $CDCl_3$) δ 171.1, 170.8, 142.1, 141.8, 138.1, 137.3, 136.7, 136.3, 129.5, 129.1, 126.9, 126.6, 126.4, 126.3, 126.3, 126.2, 125.9, 125.6, 125.0, 124.4, 120.8, 120.6, 54.3, 50.6, 35.6, 33.8, 21.9, 21.6. ESI-MS *m/z* = 246.1 $(M+H)^+$. ESI-HRMS-TOF found 246.0958, calculated 246.0947 for $(M+H)^+$. RP-HPLC R_t= 9.84 min, >99 %.

N-Acetyl-N-cyclohexyl-3-(thiophen-3-yl)benzylamine (71)

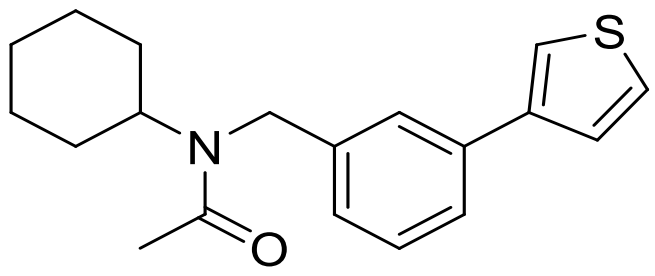

N-Cyclohexyl-3-(thiophen-3-yl)benzylamine (**33**) (53 mg, 0.20 mmol, 1 eq.), acetic anhydride (20 µL, 0.22 mmol, 1.1 eq.) and triethylamine (55 µL,

0.39 mmol, 2 eq.) was added sequentially to chloroform (5 mL) and the reaction stirred at room temperature for 16 hours. The reaction mixture was then evaporated to dryness and diluted with HCl (aq., 1 M, 10 mL) and extracted with chloroform (3 × 10 mL). The combined organic layers were dried over anhydrous magnesium sulfate, filtered, and evaporated to give an oil. Purification by column chromatography using a stepped gradient from hexane to 55:45 ethyl acetate : hexane to give the title compound as a clear oil (53 mg, 86 %). ^{1}H NMR was consistent with the presence of amide rotamers.

^{1}H NMR (400 MHz, $CDCl_3$) δ 7.48 (d, *J* = 7.7 Hz, 0.6H), 7.46 – 7.40 (m, 2.3H), 7.37 (atdd, *J* = 7.2, 4.9, 2.5 Hz, 2.4H), 7.29 (at, *J* = 7.6 Hz, 0.4H), 7.14 (d, *J* = 7.6 Hz, 1.0H), 4.69 – 4.43 (m, 2.5H), 3.63 (tt, *J* = 11.7, 3.5 Hz, 0.5H), 2.27 (s, 1.3H), 2.04 (s, 1.9H), 1.79-1.60 (m, 5.0H), 1.52 – 1.17 (m, 4.2H), 1.13 – 0.92 (m, 1.0H). ^{13}C NMR (100 MHz, $CDCl_3$) δ 171.5, 170.9, 142.5, 141.5, 140.4, 139.4, 136.3, 135.9, 129.2, 128.8, 126.52, 126.50, 126.3, 126.1, 125.9, 125.3, 125.1, 124.8, 124.6, 123.8, 120.7, 120.4, 58.8, 53.6, 47.6, 44.6, 32.1, 30.8, 26.0, 25.8, 25.6, 25.3, 22.7, 22.1. ESI-MS *m/z* = 314.1 $(M+H)^+$. ESI-HRMS-TOF found 314.1576, calculated 314.1573 for $(M+H)^+$. RP-HPLC R_t= 11.97 min, >99 %.

Methyl trans-4-((3-(thiophen-3-yl)benzylamino)methyl)cyclohexanecarboxylate (72)

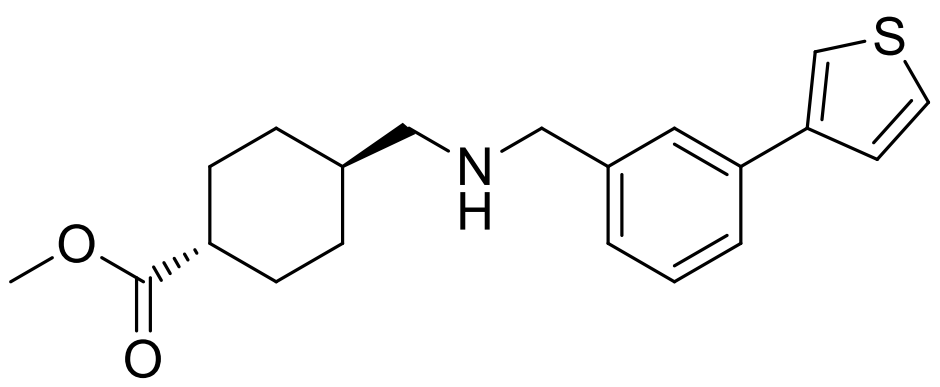

Prepared according to general procedure B with 3-(thiophen-3-yl)benzaldehyde (**S1**) (152 mg, 0.809 mmol, 1 eq.), trans-4-carboxycyclohexylmethylamine methyl ester hydrochloride (**S23**) (235 mg, 1.13 mmol, 1.4 eq.) and sodium triacetoxyborohydride (240 mg, 1.13 mmol, 1.4 eq.). Purification by column chromatography using a stepped gradient from 100:1 hexane : triethylamine to 50:50:1 hexane : ethyl acetate : triethylamine gave the title compound as a clear oil (198 mg, 71 %).

^{1}H NMR (400 MHz, $CDCl_3$) δ 7.57 – 7.53 (m, 1H), 7.50 – 7.46 (m, 1H), 7.46 (dd, *J* = 2.8, 1.5 Hz, 1H), 7.42 – 7.32 (m, 3H), 7.26 – 7.22 (m, 1H), 3.81 (s, 2H), 3.66 (s, 3H), 2.50 (d, *J* = 6.6 Hz, 2H), 2.24 (tt, *J* = 12.3, 3.6 Hz, 1H), 2.03 – 1.84 (m, 4H), 1.54 – 1.37 (m, 3H), 0.96 (aqd, *J* = 13.1, 3.4 Hz, 2H). ^{13}C NMR (100 MHz, $CDCl_3$) δ 176.6, 142.5, 141.3, 136.1, 129.0, 127.0, 126.5, 126.3 (2 carbons), 125.2, 120.4, 56.0, 54.3, 51.6, 43.6, 37.6, 30.6, 28.8. ESI-MS *m/z* = 344.1 $(M+H)^+$. ESI-HRMS-TOF found 344.1682, calculated 344.1679 for $(M+H)^+$. RP-HPLC R_t= 8.54 min, 99 %.

N-(Trans-4-(aminocarbonyl)cyclohexylmethyl)-3-(thiophen-3-yl)benzylamine (73)

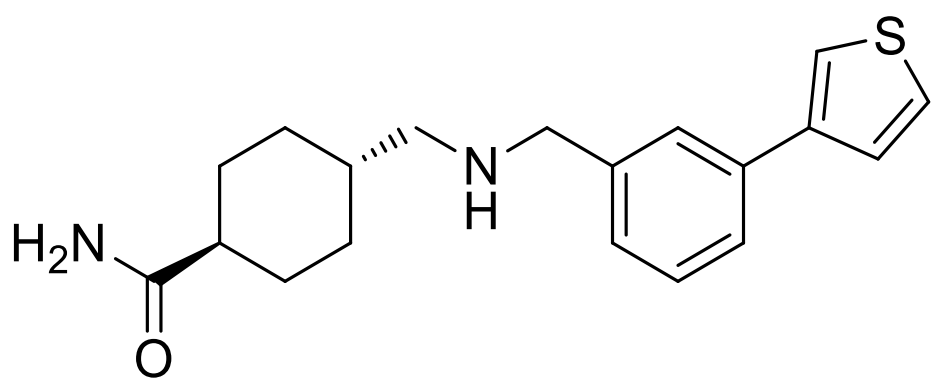

Prepared according to general procedure B with 3-(thiophen-3-yl)benzaldehyde (**S1**) (200 mg, 1.06 mmol, 1 eq.), trans-4-(aminomethyl)cyclohexaneamide hydrochloride (**S22**) (287 mg, 1.49 mmol, 1.4 eq.) and sodium triacetoxyborohydride (315 mg, 1.49 mmol, 1.4 eq.). Purification by column chromatography using a stepped gradient from 75:25:1 ethyl acetate : hexane : triethylamine to 90:10:1 ethyl acetate : methanol : triethylamine gave the title compound as a white solid (115 mg, 31 %).

^{1}H NMR (400 MHz, $CDCl_3$) δ 7.55 (s, 1H), 7.50 – 7.46 (m, 1H), 7.45 (dd, *J* = 2.8, 1.5 Hz, 1H), 7.41 – 7.31 (m, 3H), 7.24 (bd, *J* = 7.6 Hz, 1H), 5.85 (bs, 1H), 5.54 (bs, 1H), 3.81 (s, 2H), 2.49 (d, *J* = 6.6 Hz, 2H), 2.13 – 2.02 (m, 1H), 2.01 – 1.84 (m, 4H), 1.56 – 1.38 (m, 3H), 1.05 – 0.88 (m, 2H). ^{13}C NMR (100 MHz, CD_3OD) δ 178.8, 142.4, 141.1, 136.0, 128.9, 127.0, 126.5, 126.3, 126.2, 125.2, 120.4, 55.9, 54.2, 45.0, 37.5, 30.6, 29.4. ESI-MS *m/z* = 329.1 $(M+H)^+$.

ESI-HRMS-TOF found 329.1684, calculated 329.1682 for $(M+H)^+$. RP-HPLC R_t= 7.35 min, >99 %.

Lithium trans-4-((3-(thiophen-3-yl)benzylamino)methyl)cyclohexanecarboxylate (74)

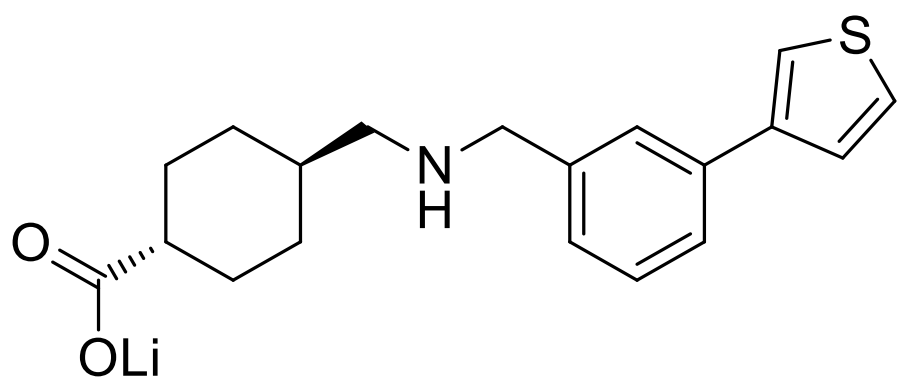

Methyl trans-4-((3-(thiophen-3-yl)benzylamino)methyl)-cyclohexanecarboxylate (**72**) (78 mg, 0.23 mmol, 1 eq.) and lithium hydroxide (16 mg, 0.68 mmol, 3 eq.) were added sequentially to methanol : water (3:1, 6 mL) and stirred at room temperature for 4 hours. The reaction mixture was then evaporated to dryness and purified by reversed-phase C18 column chromatography using a stepped gradient from 75:25 water : methanol to 75:25 methanol : water to give the title compound as a white solid (70 mg, 92 %). ^{1}H NMR (400 MHz, CD_3OD) δ 7.75 (s, 1H), 7.69 – 7.64 (m, 2H), 7.52 – 7.47 (m, 2H), 7.43 (at, J = 7.7 Hz, 1H), 7.34 (d, J = 7.6 Hz, 1H), 4.04 (s, 2H), 2.71 (d, J = 6.8 Hz, 2H), 2.07 (tt, J = 12.1, 3.4 Hz, 1H), 1.96 (add, J = 13.8, 3.0 Hz, 2H), 1.86 (add, J = 9.4, 4.2 Hz, 2H), 1.69 – 1.59 (m, 1H), 1.44 (aqd, J = 13.1, 3.3 Hz, 2H), 1.00 (aqd, J = 12.9, 3.3 Hz, 2H). ^{13}C NMR (100 MHz, CD_3OD) δ 184.9, 143.0, 137.8, 130.4, 128.9, 128.3, 127.5, 127.3, 127.3, 127.1, 121.7, 55.6, 53.6, 48.2, 37.2, 31.6, 30.7. ESI-MS *m/z* = 330.1 $(M+H)^+$. ESI-HRMS-TOF found 330.1955, calculated 330.1522 for $(M+H)^+$. RP-HPLC R_t= 7.90 min, >99 %.

***N*-((Trans-4-(hydroxymethyl)cyclohexyl)methyl)-3-(thiophen-3-yl)benzylamine (75)**

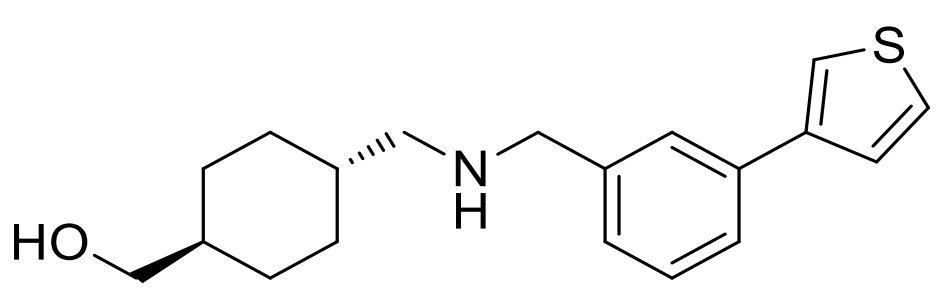

Prepared according to general procedure B with 3-(thiophen-3-yl)benzaldehyde (**S1**) (150 mg, 0.797 mmol, 1 eq.), trans-4-(aminomethyl)cyclohexylmethanol (**S24**) (160 mg, 1.12 mmol, 1.4 eq.) and sodium

triacetoxyborohydride (236 mg, 1.12 mmol, 1.4 eq.). Purification by column chromatography using a stepped gradient from 75:25:1 hexane : ethyl acetate : triethylamine to 100:1 ethyl acetate : triethylamine gave the title compound as a clear oil (133 mg, 52 %).

^{1}H NMR (400 MHz, $CDCl_3$) δ 7.56 (bs, 1H), 7.50 – 7.47 (m, 1H), 7.46 (dd, *J* = 2.9, 1.4 Hz, 1H), 7.40 (dd, *J* = 5.0, 1.4 Hz, 1H), 7.39 – 7.32 (m, 2H), 7.26 – 7.22 (m, 1H), 3.81 (s, 2H), 3.40 (d, *J* = 6.3 Hz, 2H), 2.50 (d, *J* = 6.7 Hz, 2H), 1.91 – 1.74 (m, 4H), 1.53 – 1.33 (m, 2H), 1.02 – 0.86 (m, 4H). ^{13}C NMR (100 MHz, $CDCl_3$) δ 142.4, 141.0, 136.0, 128.9, 127.0, 126.4, 126.2, 126.2, 125.1, 120.4, 68.4, 56.2, 54.2, 40.7, 38.1, 30.8, 29.2. ESI-MS *m/z* = 316.1 $(M+H)^+$. ESI-HRMS-TOF found 316.1735, calculated 316.1730 for $(M+H)^+$. RP-HPLC R_t= 8.05 min, >99 %.

1-Cyclohexyl-*N*-(3-(thiophen-3-yl)benzyl)methanamine (76)

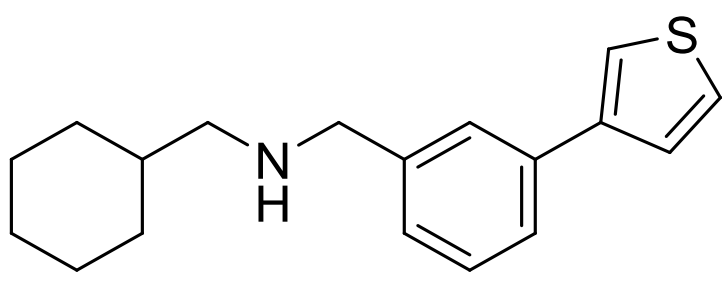

Prepared according to general procedure B with 3-(thiophen-3-yl)benzaldehyde (**S1**) (122 mg, 0.650 mmol), aminomethylcyclohexane (70 µL, 0.91 mmol, 1.4 eq.) and sodium triacetoxyborohydride (193 mg, 0.910 mmol, 1.4 eq.). Purification by column chromatography using a stepped gradient from 100:1 hexane : triethylamine to 50:50:1 hexane : ethyl acetate: triethylamine gave the title compound as a clear oil (86 mg, 47 %).

^{1}H NMR (400 MHz, $CDCl_3$) δ 7.56 (bs, 1H), 7.51 – 7.45 (m, 2H), 7.43 – 7.33 (m, 3H), 7.28 – 7.23 (m, 1H), 3.82 (s, 2H), 2.49 (d, *J* = 6.7 Hz, 2H), 1.82 – 1.62 (m, 5H), 1.56 – 1.43 (m, 1H), 1.32 – 1.09 (m, 3H), 0.99 – 0.85 (m, 2H). ^{13}C NMR (100 MHz, $CDCl_3$) δ 142.5, 141.4, 136.1, 128.9, 127.0, 126.6, 126.3, 126.2, 125.1, 120.4, 56.5, 54.3, 38.2, 31.6, 26.8, 26.2. ESI-MS *m/z* = 286.1 $(M+H)^+$. ESI-HRMS-TOF found 286.1635, calculated 286.1624 for $(M+H)^+$. RP-HPLC R_t= 9.23 min, 97 %.

Methyl trans-4-(3-(thiophen-3-yl)benzylamino)cyclohexanecarboxylate (77)

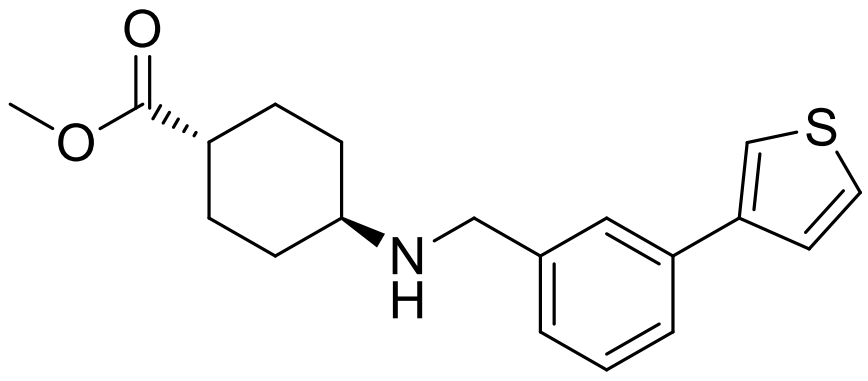

Prepared according to general procedure B with 3-(thiophen-3-yl)benzaldehyde (**S1**) (104 mg, 0.553 mmol, 1 eq.), trans-4-carboxycyclohexylamine methyl ester hydrochloride (**S25**) (150 mg, 0.775 mmol, 1.4 eq.) and sodium triacetoxyborohydride (164 mg, 0.775 mmol, 1.4 eq.). Purification by column chromatography using a stepped gradient from 75:25:1 hexane : ethyl acetate : triethylamine to 75:25:1 ethyl acetate : hexane : triethylamine to give the title compound as a white solid (45 mg, 25 %).

^{1}H NMR (400 MHz, $CDCl_3$) δ 7.55 (bs, 1H), 7.50 – 7.47 (m, 1H), 7.46 (dd, *J* = 2.7, 1.6 Hz, 1H), 7.41 – 7.32 (m, 3H), 7.24 (bd, *J* = 7.6 Hz, 1H), 3.86 (s, 2H), 3.66 (s, 3H), 2.53 (tt, *J* = 11.1, 3.7 Hz, 1H), 2.34 – 2.23 (m, 1H), 2.09 – 1.96 (m, 4H), 1.54 – 1.41 (m, 2H), 1.23 – 1.10 (m, 2H). ^{13}C NMR (100 MHz, $CDCl_3$) δ 176.4, 142.5, 141.4, 136.2, 129.1, 127.0, 126.6, 126.3, 126.3, 125.2, 120.5, 55.9, 51.7, 51.4, 43.11, 32.7, 27.9. ESI-MS *m/z* = 330.1 $(M+H)^+$. ESI-HRMS-TOF found 330.1530, calculated 330.1522 for $(M+H)^+$. RP-HPLC R_t= 8.11 min, 99 %.

***N*-(Trans-4-hydroxycyclohexyl)-3-(thiophen-3-yl)benzylamine (78)**

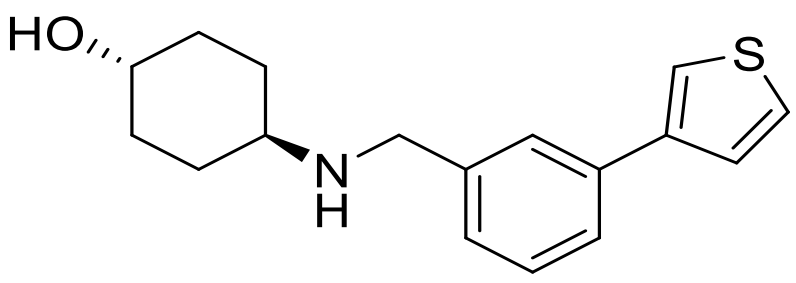

Prepared according to general procedure B with 3-(thiophen-3-yl)benzaldehyde (**S1**) (205 mg, 1.09 mmol, 1 eq.), trans-4-hydroxycyclohexylamine hydrochloride (231 mg, 1.52 mmol, 1.4 eq.) and sodium triacetoxyborohydride (323 mg, 1.52 mmol, 1.4 eq.). Purification by column chromatography using a stepped gradient from 75:25:1 ethyl acetate : hexane : triethylamine to 85:15:1 ethyl acetate : methanol : triethylamine gave the title compound as a white solid (182 mg, 58 %).

^{1}H NMR (400 MHz, CD_3OD) δ 7.65 (at, *J* = 1.5 Hz, 1H), 7.62 (dd, *J* = 2.4, 1.9 Hz, 1H), 7.57 – 7.53 (m, 1H), 7.48 – 7.44 (m, 2H), 7.35 (at, *J* = 7.6 Hz, 1H), 7.24 (bd, *J* = 7.8 Hz, 1H), 3.79 (s, 2H), 3.57 – 3.46 (m, 1H), 2.54 – 2.44 (m, 1H), 2.06 – 1.87 (m, 4H), 1.31 – 1.15 (m, 4H). ^{13}C NMR (100 MHz, CD_3OD) δ 143.4, 141.4, 137.4, 130.0, 128.2, 127.5, 127.3, 127.2, 126.1, 121.4, 71.0, 56.5, 51.7, 34.7, 31.4. ESI-MS *m/z* = 288.1 $(M+H)^+$. ESI-HRMS-TOF found 288.1414, calculated 288.1417 for $(M+H)^+$. RP-HPLC R_t= 6.54 min, >99 %.

***N*-(Pyridine-2-ylmethyl)-3-(thiophen-3-yl)benzylamine (79)**

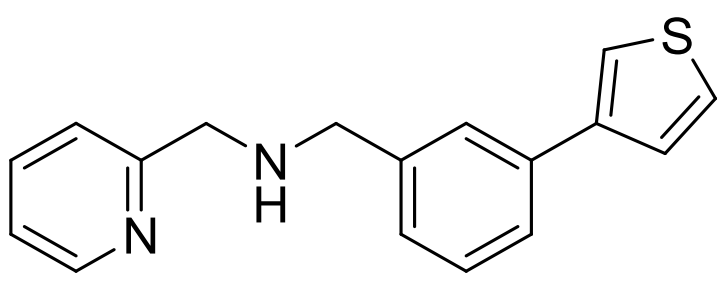

Prepared according to general procedure B with 3-(thiophen-3-yl)benzaldehyde (**S1**) (54 mg, 0.28 mmol, 1 eq.), 2-aminomethylpyridine (41 µL, 0.40 mmol, 1.4 eq.) and sodium triacetoxyborohydride (84 mg, 0.40 mmol, 1.4 eq.). Purification by column chromatography using a stepped gradient from 75:25:1 ethyl acetate : hexane : triethylamine to 85:15:1 ethyl acetate : methanol : triethylamine gave the title compound as a clear oil (52 mg, 66 %).

^{1}H NMR (400 MHz, CD_3OD) δ 8.47 (ddd, *J* = 5.0, 1.7, 0.9 Hz, 1H), 7.75 (atd, *J* = 7.7, 1.8 Hz, 1H), 7.65 – 7.62 (m, 1H), 7.58 (at, *J* = 2.2 Hz, 1H), 7.54 – 7.49 (m, 1H), 7.45 – 7.40 (m, 3H), 7.32 (at, *J* = 7.6 Hz, 1H), 7.28 – 7.20 (m, 2H), 3.87 (s, 2H), 3.79 (s, 2H). ^{13}C NMR (100 MHz, CD_3OD) δ 160.1, 149.8, 143.4, 141.0, 138.6, 137.4, 130.0, 128.3, 127.5, 127.3, 127.2, 126.2, 124.1, 123.7, 121.4, 54.5, 53.9. ESI-MS *m/z* = 281.1 $(M+H)^+$. ESI-HRMS-TOF found 281.1104, calculated 281.1107 for $(M+H)^+$. RP-HPLC R_t= 7.74 min, >99 %.

***N*-(Pyridin-3-ylmethyl)-3-(thiophen-3-yl)benzylamine (80)**

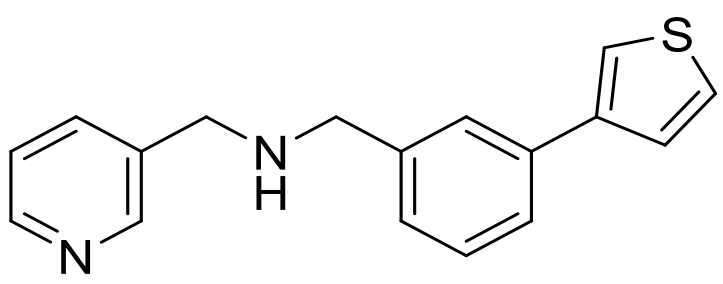

Prepared according to general procedure B with 3-(thiophen-3-yl)benzaldehyde (**S1**) (54 mg, 0.28 mmol, 1 eq.), 3-aminomethylpyridine (41 µL, 0.40 mmol, 1.4 eq.) and sodium triacetoxyborohydride (84 mg, 0.40 mmol, 1.4 eq.). Purification by column chromatography using a stepped gradient from 75:25:1 ethyl acetate : hexane : triethylamine to 85:15:1 ethyl acetate : methanol : triethylamine gave the title compound as a clear oil (51 mg, 64 %).

^{1}H NMR (400 MHz, CD_3OD) δ 8.49 (d, *J* = 1.6 Hz, 1H), 8.40 (dd, *J* = 4.9, 1.6 Hz, 1H), 7.83 – 7.76 (m, 1H), 7.62 (at, *J* = 1.4 Hz, 1H), 7.58 (at, *J* = 2.2 Hz, 1H), 7.56 – 7.49 (m, 1H), 7.43 (ad, *J* = 2.2 Hz, 2H), 7.39 – 7.30 (m, 2H), 7.23 (bd, *J* = 7.6 Hz, 1H), 3.77 (as, 4H). ^{13}C NMR (100 MHz, CD_3OD) δ 150.3, 148.7, 143.4, 141.0, 138.5, 137.4, 137.1, 130.0, 128.3, 127.5, 127.3, 127.2, 126.2, 125.1, 121.4, 53.8, 50.8. ESI-MS *m/z* = 281.1 $(M+H)^+$. ESI-HRMS-TOF found 281.1105, calculated 281.1107 for $(M+H)^+$. RP-HPLC R_t= 5.80 min, 99 %.

***N*-(Pyridine-4-ylmethyl)-3-(thiophen-3-yl)benzylamine (81)**

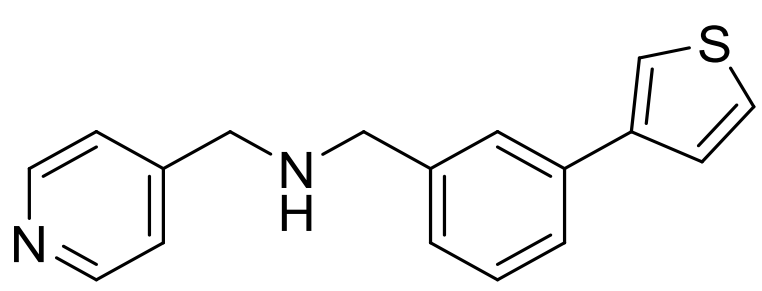

Prepared according to general procedure B with 3-(thiophen-3-yl)benzaldehyde (**S1**) (57 mg, 0.30 mmol, 1 eq.), 2-aminomethylpyridine (43 µL, 0.42 mmol, 1.4 eq.) and sodium triacetoxyborohydride (89 mg, 0.42 mmol, 1.4 eq.). Purification by column chromatography using a stepped gradient from 75:25:1 ethyl acetate : hexane : triethylamine

to 90:10:1 ethyl acetate : methanol : triethylamine gave the title compound as a clear oil (65 mg, 77 %).

^{1}H NMR (400 MHz, CD_3OD) δ 8.44 – 8.41 (m, 2H), 7.61 (at, *J* = 1.5 Hz, 1H), 7.57 (at, *J* = 2.2 Hz, 1H), 7.54 – 7.49 (m, 1H), 7.43 (ad, *J* = 2.2 Hz, 2H), 7.39 – 7.35 (m, 2H), 7.32 (at, *J* = 7.6 Hz, 1H), 7.24 – 7.20 (m, 1H), 3.77 (s, 2H), 3.75 (s, 2H). ^{13}C NMR (100 MHz, CD_3OD) δ 151.6, 150.0, 143.4, 141.1, 137.3, 130.0, 128.2, 127.4, 127.3, 127.2, 126.2, 124.9, 121.4, 53.8, 52.3. ESI-MS *m/z* = 281.1 $(M+H)^+$. ESI-HRMS-TOF found 28.1101, calculated 281.1107 for $(M+H)^+$. RP-HPLC R_t= 5.28 min, 95 %.

_N_-(_N_-Boc-2-aminoacetyl)-_N_-methyl-3-thiophen-3-ylbenzylamine (82)

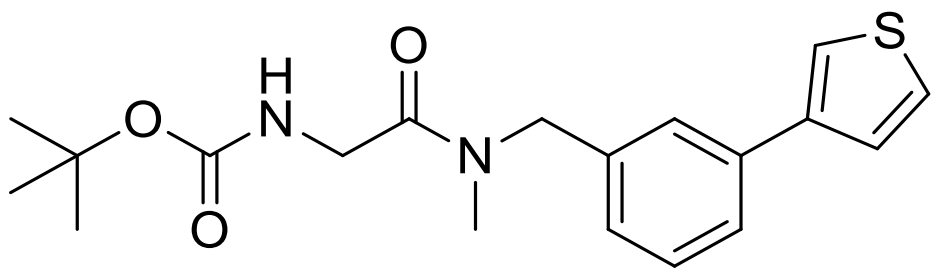

N-Boc-Glycine (15 mg, 0.08 mmol, 1 eq.), triethylamine (29 μL, 0.21 mmol, 2.5 eq.) and ethyl chloroformate (8.0 μL, 0.083 mmol, 1 eq.) were added sequentially to dry THF (3 mL) at -10 °C under a nitrogen atmosphere and stirred at -5 °C for 30 mins. *N*-Methyl-3-thiophen-3-ylbenzyl amine hydrochloride (**1**) (20 mg, 0.08 mmol, 1 eq.) in DMF (1 mL) was then added and the reaction was allowed to stir at room temperature for 16 hours. The reaction was then diluted with NaOH (aq., 1 M, 15 mL) and extracted with ethyl acetate (3 × 15 mL). The combined organic layers were dried over anhydrous magnesium sulfate filtered, and evaporated to give a clear oil. Purification by column chromatography using a stepped gradient from 90:10 hexane : ethyl acetate to 60:40 ethyl acetate : hexane gave the title compound as a clear oil (7 mg, 21 %). ^{1}H NMR was consistent with rotamers of the amide in a ratio of ~2:1.

^{1}H NMR (400 MHz, $CDCl_3$): δ 7.55 – 7.49 (m, 1.5H), 7.48 – 7.43 (m, 2.5H), 7.41 – 7.33 (m, 5H), 7.15 (bd, *J* = 7.7 Hz, 1H), 7.07 (bd, *J* = 7.6 Hz, 0.5H), 4.65 (s, 2H), 4.50 (s, 1H), 4.06 (d, *J* = 4.2 Hz, 1H), 4.02 (d, *J* = 4.2 Hz, 2H), 3.01 (s, 1.5H), 2.90 (s, 3H), 1.46 (s, 9H), 1.44 (s, 4.5H). ^{13}C NMR (100 MHz, $CDCl_3$): δ 168.9, 168.8, 142.1, 141.8, 137.4, 136.9, 136.5, 136.4, 129.7, 129.3, 127.0, 126.6, 126.49, 126.47, 126.3, 126.2, 126.0, 125.2, 124.6, 121.0, 120.8, 79.8, 52.4, 51.4, 42.6, 42.4, 34.2, 33.6, 28.5. ESI-MS *m/z* = 173.1 (100 %, M-$C_8H_{12}O_3N_2)^+$; 261.2 (55 %, M-Boc+2H)$^+$. ESI-HRMS-TOF found 361.1591, calculated 361.1580 for $(M+H)^+$. RP-HPLC R_t= 10.68 min, 98 %.

***N*-(*N*-Boc-3-aminopropanoyl)-*N*-methyl-3-thiophen-3-ylbenzylamine (83)**

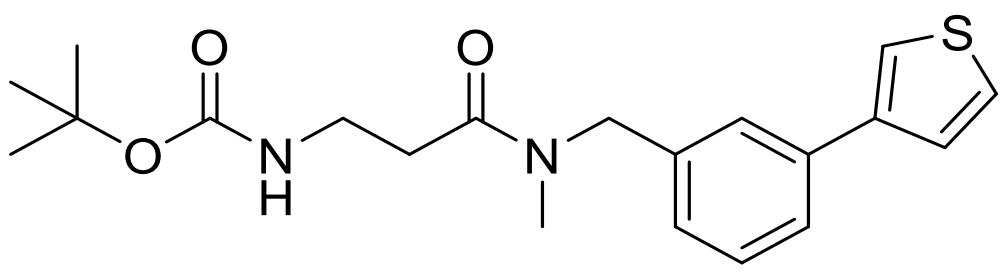

N-Boc-*β*-Alanine (16 mg, 0.08 mmol, 1 eq.), triethylamine (29 µL, 0.21 mmol, 2.5 eq.) and ethyl chloroformate (8.0 µL, 0.08 mmol, 1 eq.) were added sequentially to dry THF (3 mL) at -10 °C under a nitrogen atmosphere and stirred at -5 °C for 30 mins. *N*-Methyl-3-thiophen-3-ylbenzyl amine hydrochloride (**1**) (20 mg, 0.08 mmol, 1 eq.) in DMF (1 mL) was then added and the reaction was allowed to stir at room temperature for 16 hours. The reaction was then diluted with NaOH (aq., 1 M, 15 mL) and extracted with ethyl acetate (3 × 15 mL). The combined organic layers were dried over anhydrous magnesium sulfate filtered, and evaporated to give a clear oil. Purification by column chromatography using a stepped gradient from 90:10 hexane : ethyl acetate to 50:50 hexane : ethyl acetate gave the title compound as a clear oil (20 mg, 64%). ^{1}H NMR was consistent with rotamers of the amide in a ratio of ~3:2.

^{1}H NMR (400 MHz, $CDCl_3$): δ 7.53 – 7.48 (m, 1.6H), 7.45 – 7.43 (m, 2.4H), 7.41 – 7.32 (m, 5.5H), 7.14 (bd, *J* = 7.6 Hz, 1H), 7.06 (bd, *J* = 7.6 Hz, 0.6H), 4.63 (s, 2H), 4.54 (s, 1.3H), 3.51 – 3.41 (m, 3.4H), 2.98 (s, 2H), 2.91 (s, 3H), 2.62 – 2.54 (m, 3.3H), 1.43 (s, 9H), 1.41 (s, 5.8H). ^{13}C NMR (100 MHz, $CDCl_3$): δ 172.2, 172.0, 156.21, 156.16, 142.1, 141.9, 137.9, 137.0, 136.8, 136.4, 129.6, 129.3, 126.8, 126.6, 126.5, 126.42, 126.38, 126.2, 126.0, 125.8, 125.1, 124.5, 120.9, 120.7, 79.2, 53.3, 50.9, 36.5, 34.8, 34.0, 33.8, 33.5, 28.6, 28.5. ESI-MS *m/z* = 173.1 (26 %, M-$C_9H_{14}O_3N_2$)$^+$; 275.2 (100 %, M-Boc+2H)$^+$. ESI-HRMS-TOF found 375.1742, calculated 375.1737 for (M+H)$^+$. RP-HPLC R_t= 10.91 min, >99 %.

_N_-Methanesulfonyl-_N_-methyl-3-thiophen-3-ylbenzylamine (84)

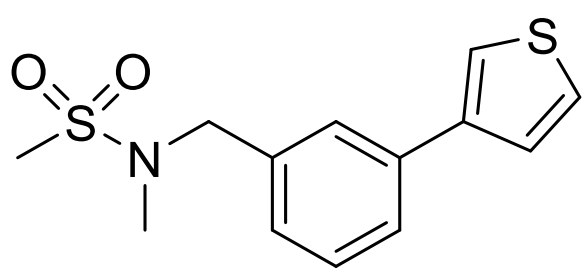

N-Methyl-3-thiophen-3-ylbenzylamine hydrochloride (**1**) (23 mg, 0.09 mmol, 1 eq.), triethylamine (33 µL, 0.23 mmol, 2.5 eq.) and methanesulfonyl chloride (9.0 µL, 0.11 mmol, 1.2 eq.) were added sequentially to chloroform (3 mL). The reaction was sealed under a nitrogen atmosphere and stirred at room temperature for 16 hours. The reaction was then diluted with NaOH (aq., 1 M, 15 mL) and extracted with chloroform (3 × 15 mL) and the combined organic layers were dried over anhydrous magnesium sulfate filtered, and evaporated to give a clear oil. Purification by column chromatography using a stepped gradient from 90:10 hexane : ethyl acetate to 60:40 hexane : ethyl acetate gave the title compound as a clear oil (20 mg, 77 %).

^{1}H NMR (400 MHz, $CDCl_3$): δ 7.59 – 7.56 (m, 1H), 7.56 – 7.54 (m, 1H), 7.48 (at, *J* = 2.2 Hz, 1H), 7.42 – 7.37 (m, 3H), 7.28 (ddd, *J* = 7.6, 1.6, 1.1 Hz, 1H), 4.35 (s, 2H), 2.86 (s, 3H), 2.80

(s, 3H). ^{13}C NMR (100 MHz, $CDCl_3$): δ 141.9, 136.5, 136.3, 129.4, 127.2, 126.5, 126.5, 126.4, 126.3, 120.8, 54.1, 36.1, 34.5. ESI-MS *m/z* = 173.1 $(M-C_2H_6O_2N_1S_1)^+$. RP-HPLC R_t= 9.20 min, 99 %.

***N*-Toluenesulfonyl-*N*-methyl-3-thiophen-3-ylbenzyl amine (85)**

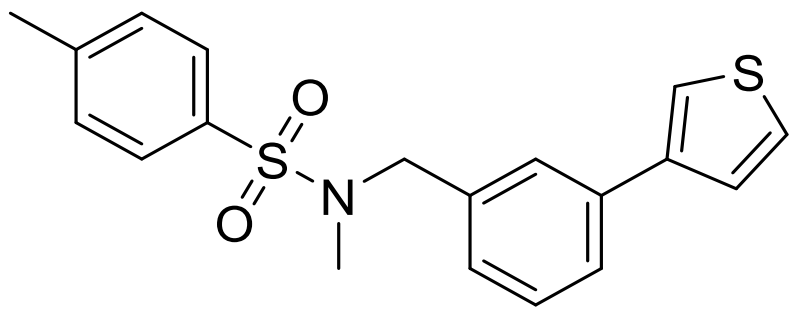

N-Methyl-3-thiophen-3-ylbenzyl amine hydrochloride (**1**) (28 mg, 0.12 mmol, 1.05 eq.), triethylamine (39 μL, 0.28 mmol, 2.5 eq.) and toluenesulfonyl chloride (22 mg, 0.11 mmol, 1.0 eq.) were added sequentially to chloroform (3 mL). The reaction was sealed under a nitrogen atmosphere and stirred at room temperature for 16 hours. The reaction was then diluted with NaOH (aq., 1 M, 15 mL) and extracted with chloroform (3 × 15 mL) and the combined organic layers were dried over anhydrous magnesium sulfate filtered, and evaporated to give a clear oil. Purification by column chromatography using a stepped gradient from 95:5 hexane : ethyl acetate to 80:20 hexane : ethyl acetate gave the title compound as a clear oil (24 mg, 76 %).

^{1}H NMR (400 MHz, $CDCl_3$): δ 7.77 – 7.73 (m, 2H), 7.55 – 7.49 (m, 2H), 7.44 (dd, *J* = 2.9, 1.5 Hz, 1H), 7.40 – 7.33 (m, 5H), 7.22 (d, *J* = 7.6 Hz, 1H), 4.18 (s, 2H), 2.62 (s, 3H), 2.46 (s, 3H). ^{13}C NMR (100 MHz, $CDCl_3$): δ 143.6, 142.0, 136.4, 136.4, 134.6, 129.9, 129.3, 127.7, 127.3, 126.4, 126.4, 126.1, 120.7, 54.3, 34.6, 21.7. An additional resonance is expected, and presumably coincident with a reported signal, however ^{13}C-^{1}H HSQC and ^{13}C-^{1}H HMBC NMR were unable to identify the overlapped peak. ESI-HRMS-TOF found 358.0934, calculated 358.0930 for $(M+H)^+$. RP-HPLC R_t= 11.56 min, 99 %.

***N*-(Trifluoroacetyl)-*N*-methyl-3-(thiophen-3-yl)benzylamine (86)**

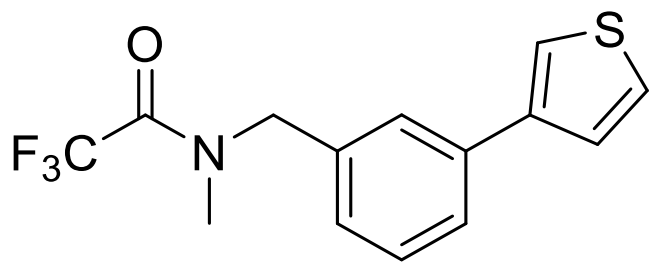

N-Methyl-3-(thiophen-3-yl)benzylamine hydrochloride (**1**) (50 mg, 0.21 mmol, 1 eq.), trifluoroacetic anhydride (36 µL, 0.25 mmol, 1.2 eq.) and triethylamine (59 µL, 0.42 mmol, 2 eq.) were added sequentially to chloroform (5 mL) and the reaction was stirred at room temperature for 16 hours. The reaction mixture was then evaporated to dryness and diluted with HCl (aq., 1 M, 10 mL) and the resulting mixture was extracted with chloroform (3 × 15 mL). The combined organic layers were dried over anhydrous magnesium sulfate, filtered, and evaporated to give an oil. Purification by column chromatography using a stepped gradient from hexane to 90:10 hexane : ethyl acetate gave the title compound as a clear oil (15 mg, 24 %). ^{1}H NMR was consistent with rotamers of the amide.

1H NMR (400 MHz, CDCl3) δ 7.60 – 7.54 (m, 1H), 7.51 – 7.43 (m, 2H), 7.43 – 7.36 (m, 3H), 7.21 – 7.12 (m, 1H), 4.70 – 4.65 (m, 2H), 3.08 (q, *J* = 1.5 Hz, 2H), 2.96 (s, 1H). ^{13}C NMR (100 MHz, CDCl3) δ 157.7, 157.3, 141.8, 141.7, 136.9, 136.7, 135.9, 135.4, 129.7, 129.6, 127.0, 126.7, 126.61, 126.56, 126.41, 126.39, 126.33, 126.0, 125.4, 121.0, 120.9, 116.83 (q, *J* = 287.7 Hz), 116.75 (q, *J* = 288.0 Hz), 53.0 (q, *J* = 3.4 Hz), 52.5, 34.5 (q, *J* = 3.9 Hz) 34.3. ESI-MS *m/z* = 173.1 (100 %, M-N(CH_3)$COCF_3$)$^+$; 299.9 (6.1 %, M+H)$^+$. ESI-HRMS-TOF found 173.0409, calculated 173.0419 for (M-N(CH_3)$COCF_3$)$^+$. RP-HPLC Rt= 10.9 min, 96 %.

_N_-Pivaloyl-_N_-methyl-3-thiophen-3-ylbenzyl amine (87)

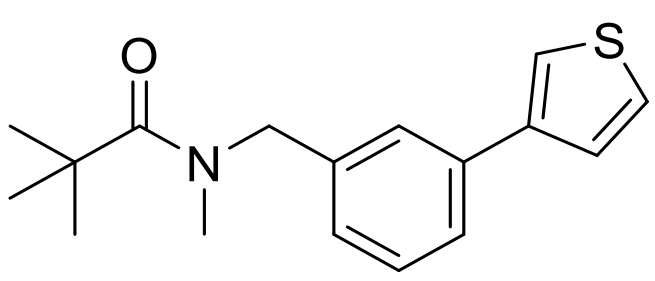

N-Methyl-3-thiophen-3-ylbenzyl amine hydrochloride (**1**) (22 mg, 0.09 mmol, 1 eq.), triethylamine (32 µL, 0.23 mmol, 2.5 eq.) and pivaloyl chloride (14 µL, 0.11 mmol, 1.2 eq.) were added sequentially to chloroform (3 mL). The reaction was sealed under a nitrogen atmosphere and stirred at room temperature for 16 hours. The reaction was

then diluted with NaOH (aq., 1 M, 15 mL) and extracted with chloroform (3 × 15 mL), the combined organic layers were dried over anhydrous magnesium sulfate filtered, and evaporated to give a clear oil. Purification by column chromatography using a stepped gradient from 80:20 hexane : ethyl acetate to 60:40 hexane : ethyl acetate gave the title compound as a clear oil (22 mg, 82 %).

^{1}H NMR (400 MHz, $CDCl_3$): δ 7.50 (bd, *J* = 7.7 Hz, 1H), 7.44 (dd, *J* = 2.8, 1.5 Hz, 1H), 7.41 (bs, 1H), 7.40 – 7.34 (m, 3H), 7.13 (bdd, *J* = 7.6, 0.6 Hz, 1H), 4.69 (s, 2H), 3.01 (s, 3H), 1.35 (s, 9H). ^{13}C NMR (100 MHz, $CDCl_3$): δ 177.9, 142.2, 138.3, 136.3, 129.3, 126.4 (2 carbons), 126.2, 125.5, 125.4, 120.6, 53.3, 39.1, 36.3, 28.6. ESI-MS *m/z* = 288.2 $(M+H)^+$. ESI-HRMS-TOF found 288.1424, calculated 288.1417 for $(M+H)^+$. RP-HPLC R_t= 11.00 min, 99 %.

_N_-Benzoyl-_N_-methyl-3-thiophen-3-ylbenzyl amine (88)

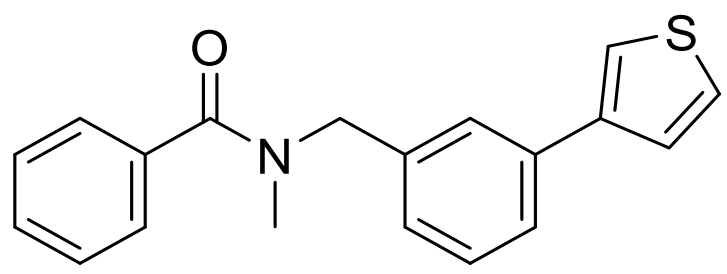

N-Methyl-3-thiophen-3-ylbenzyl amine hydrochloride (**1**) (23 mg, 0.10 mmol, 1 eq.), triethylamine (34 μL, 0.24 mmol, 2.5 eq.) and benzoyl chloride (13 μL, 0.12 mmol, 1.2 eq.) were added sequentially to chloroform (3 mL). The reaction was sealed under a nitrogen atmosphere and stirred at room temperature for 16 hours. The reaction was then diluted with NaOH (aq., 1 M, 15 mL) and extracted with chloroform (3 × 15 mL), the combined organic layers were washed with HCl (aq., 1 M, 2 × 15 mL) and the organic layer dried over anhydrous magnesium sulfate filtered, and evaporated to give a clear oil. Purification by column chromatography using a stepped gradient from hexane to 60:40 hexane : ethyl acetate to give the title compound as a clear oil (20 mg, 66 %). ^{1}H NMR was consistent with rotamers of the amide.

^{1}H NMR (400 MHz, $CDCl_3$): δ 7.64 – 7.24 (m, 22H), 7.11 (d, *J* = 7.1 Hz, 1H), 4.81 (s, 2H), 4.56 (s, 1.8 H), 3.08 (s, 2.7 H), 2.89 (s, 3H). ^{13}C NMR (100 MHz, $CDCl_3$): δ 172.6, 171.9, 142.2, 142.0, 137.9, 137.5, 136.7, 136.5, 136.4, 136.4, 129.9, 129.6, 129.4, 128.8, 128.7, 127.2, 127.0, 126.7, 126.5, 125.9, 125.6, 125.0, 120.9, 120.8, 55.4, 51.0, 37.3, 33.5. ^{1}H NMR (400 MHz, CD_3OD): δ 7.67 – 7.55 (m, 4.8H), 7.50 – 7.35 (m, 16H), 7.29 (bd, *J* = 7.4 Hz, 1H), 7.09 (bd, *J* = 7.2 Hz, 0.8H), 4.79 (s, 2H), 4.56 (s, 1.6H), 3.04 (s, 2.4H), 2.90 (s, 3H). ^{13}C NMR (100 MHz, CD_3OD): δ 174.6, 173.9, 143.2, 143.0, 138.8, 138.5, 137.8, 137.8, 137.3, 131.0, 130.5, 130.4, 129.7, 127.9, 127.7, 127.5, 127.1, 126.7, 126.7, 126.5, 125.9, 121.7, 121.6, 56.1, 51.8, 37.7, 33.8. ESI-MS *m/z* = 308.2 $(M+H)^+$. ESI-HRMS-TOF found 308.1111, calculated 308.1104 for $(M+H)^+$. RP-HPLC R_t= 10.64 min, 99 %.

N-(Phenylacetyl)-N-methyl-3-(thiophen-3-yl)benzylamine (89)

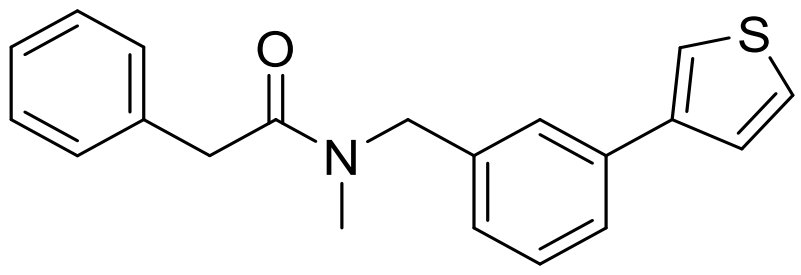

Phenylacetic acid (23 mg, 0.17 mmol, 1 eq.), *N*,*N*-dimethyl-4-aminopyridine (26 mg, 0.21 mmol, 1.25 eq.) and 1-ethyl-3-(3-dimethylaminopropyl) carbodiimide hydrochloride (40 mg, 0.21 mmol, 1.25 eq.) were added sequentially to dry dichloromethane (1.5 mL) and sealed under nitrogen and stirred at room temperature for 2 hours. *N*-Methyl-3-(thiophen-3-yl)benzylamine hydrochloride (**1**) (50 mg, 0.21 mmol, 1.25 eq.) was then added and the reaction continued stirring at room temperature under nitrogen for 48 hours. The reaction was then diluted with HCl (aq., 1 M, 15 mL) and the resulting mixture was extracted with chloroform (4 × 15 mL). The combined organic layers were dried over anhydrous magnesium sulfate, filtered, and evaporated to give a clear oil. Purification by column chromatography using a stepped gradient from 60:40 hexane : ethyl acetate to 60:40

ethyl acetate: hexane gave the title compound as a clear oil (35 mg, 65 %). ^{1}H NMR was consistent with rotamers of the amide in a ratio of ~3:2.

^{1}H NMR (400 MHz, $CDCl_3$): δ 7.51 (dd, *J* = 12.9, 7.8 Hz, 1H), 7.43 – 7.22 (m, 10H), 7.15 (d, *J* = 7.6 Hz, 0.6H), 7.03 (d, *J* = 7.6 Hz, 0.4H), 4.67 (s, 1.2H), 4.58 (s, 0.8H), 3.83 – 3.78 (m, 2H), 2.98 (s, 1.2H), 2.94 (s, 1.8H). ^{13}C NMR (100 MHz, $CDCl_3$): δ 171.6, 171.4, 142.1, 141.8, 138.0, 137.2, 136.7, 136.3, 135.2, 135.0, 129.5, 129.1, 128.89, 128.86, 127.0, 126.9, 126.9, 126.5, 126.4, 126.3, 126.3, 125.9, 125.9, 125.5, 125.2, 124.5, 120.9, 120.6, 53.8, 51.0, 41.3, 41.0, 35.4, 34.1. ESI-MS *m/z* = 322.2 $(M+H)^+$. ESI-HRMS-TOF found 322.1256, calculated 323.1260 for $(M+H)^+$. RP-HPLC R_t= 10.94 min, 99 %.

***N*-(Pyridin-2-ylacetyl)-*N*-methyl-3-(thiophen-3-yl)benzylamine (90)**

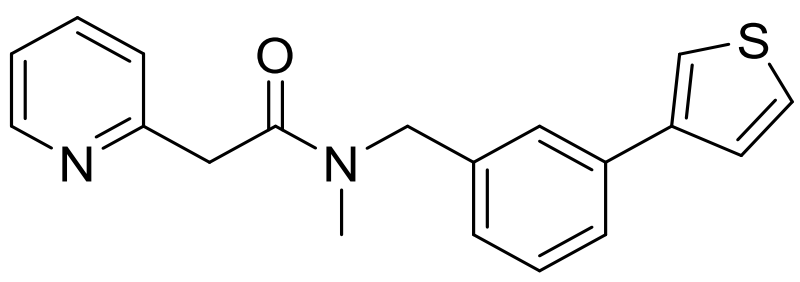

Pyridin-2-ylacetic acid (29 mg, 0.17 mmol, 1 eq.), *N*,*N*-dimethyl-4-aminopyridine (26 mg, 0.21 mmol, 1.25 eq.) and 1-ethyl-3-(3-dimethylaminopropyl) carbodiimide hydrochloride (40 mg, 0.21 mmol, 1.25 eq.) were added sequentially to dry dichloromethane (1.5 mL) and sealed under nitrogen and stirred at room temperature for 2 hours. *N*-Methyl-3-(thiophen-3-yl)benzylamine hydrochloride (50 mg, 0.21 mmol, 1.25 eq.) was then added and the reaction continued stirring at room temperature under nitrogen for 48 hours. The reaction was then diluted with HCl (aq., 1 M, 15 mL) and the resulting mixture was extracted with chloroform (4 × 15 mL). The combined organic layers were dried over anhydrous magnesium sulfate, filtered, and evaporated to give a clear oil. Purification by column chromatography using a stepped gradient from ethyl acetate to 94:6

ethyl acetate: methanol gave the title compound as a clear oil (20 mg, 37 %). ^{1}H NMR was consistent with rotamers of the amide in a ratio of ~3:2.

^{1}H NMR (400 MHz, $CDCl_3$): δ 8.55 – 8.46 (m, 1H), 7.64 (atdd, *J* = 7.8, 6.0, 1.8 Hz, 1H), 7.52 – 7.30 (m, 7H), 7.21 – 7.11 (m, 1.6H), 7.05 (bd, *J* = 7.6 Hz, 0.4H), 4.75 (s, 0.8H), 4.66 (s, 1.2H), 4.04 – 3.98 (m, 2H), 3.02 (s, 1.8H), 2.97 (s, 1.2H). ^{13}C NMR (100 MHz, $CDCl_3$): δ 170.7, 170.4, 155.9, 149.4, 149.3, 142.2, 141.9, 137.9, 137.3, 136.9, 136.8, 136.6, 136.3, 129.4, 129.1, 126.9, 126.50, 126.47, 126.4, 126.3, 126.1, 125.8, 125.6, 125.3, 124.6, 124.0, 123.9, 122.0, 120.8, 120.6, 54.0, 51.1, 43.9, 43.8, 35.6, 34.1. ESI-MS *m/z* = 323.2 $(M+H)^+$. ESI-HRMS-TOF found 323.1227, calculated 323.1213 for $(M+H)^+$. RP-HPLC R_t= 7.75 min, 98 %.

1-Fluoro-4-(triisopropylsilyl)but-3-yn-2-one (**91**)

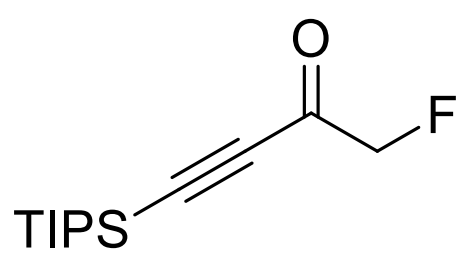

Method A:

To a solution of triisopropylsilylacetylene (220 µL, 0.968 mmol, 1 eq.) in dry THF (2 mL) at -20 ºC (dry ice/acetone bath) was added *n*-butyl lithium in hexane (1.4 M, 692 µL, 0.968 mmol, 1 eq.) over 3 mins, maintaining the temperature below 0 ºC. The solution was allowed to warm to 0 ºC and then stirred for 30 mins at 0 ºC. The solution was then cooled to -20 ºC and 2-fluoro-1-morpholinoethanone (**93**) (150 mg, 1.019 mmol, 1.05 eq.) was added dropwise over 5 mins. The solution was allowed to warm to 0 ºC and stirred for 1.5 hours. The reaction was quenched with acetic acid : water (2:1, 7.5 mL) and separated. The organic layer was further washed with water (3 mL), dried over anhydrous magnesium sulfate, filtered, and evaporated

to give a yellow oil which was purified by column chromatography in a gradient of hexane to 94:6 hexane : ethyl acetate to give the desired fluoromethylketone as a clear oil (41 mg, 17 %). ^{1}H NMR (400 MHz, $CDCl_3$) δ 4.96 (d, $J_{H,F}$ = 47.2 Hz, 2H), 1.24 – 1.01 (m, 21H). ^{13}C NMR (100 MHz, $CDCl_3$) δ 181.4 (d, $J_{C,F}$ = 22.4 Hz), 102.6, 100.5, 85.0 (d, $J_{C,F}$ = 189 Hz), 18.6, 11.1. ESI-HRMS-TOF found 243.1573, calculated 243.1575 for $(M+H)^+$. RP-HPLC R_t= 14.14 min, 98 %.

Method B:

Triisopropylsilylacetylene (300 µL, 0.148 mmol, 1 eq.) was added to dry THF (4 mL) and cooled to -78 ^{o}C under nitrogen, followed by addition of *n*-butyl lithium (1.45 M, 1.04 mL, 1 eq.). The mixture was stirred at -78 ^{o}C for 30 mins. 2-Fluoro-*N*-methoxy-*N*-methylacetamide (**95**) (200 mg, 1.65 mmol, 1.1 eq.) was added to dry THF (3mL) and cooled to -78 ^{o}C under nitrogen followed by dropwise addition of the acetylide mixture over 10 mins. The reaction was left stirring at -78 ^{o}C for 1 hour then warmed at room temp. for 10 mins. The reaction was then quenched with HCl (aq., 1 M, 20 mL) and the resulting mixture was extracted with diethyl ether (3 × 20 mL). The combined organic layers were dried over anhydrous magnesium sulfate, filtered, and evaporated to give a yellow oil (383 mg) which was immediately diluted with methanol. Concentration was calculated from the ^{1}H-NMR integrations of signals at 4.96, 4.91, 3.75, 2.35 ppm indicative of 1-fluoro-4-(triisopropylsilyl)but-3-yn-2-one (**152**), 2-fluoro-*N*-methoxy-*N*-methylacetamide (**155**), tetrahydrofuran and triisopropylacetylene respectively corresponding to a yield of 59 %. The solution was used without further purification in subsequent reactions.

4-Fluoro-5-(fluoromethyl)-5-hydroxy-1,7-bis(triisopropylsilyl)hepta-1,6-diyn-3-one (92a)

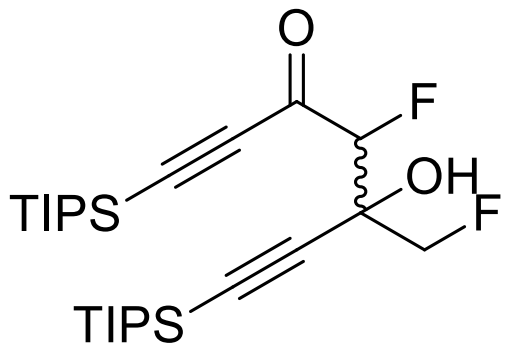

Also isolated from above method A (90 mg, 27 %).

^{1}H-NMR (400 MHz CDCl$_3$): δ 4.87 (d, $J_{H,F}$ = 46.9 Hz, 1H), 4.70 – 4.38 (m, 2H), 3.95 (at, J = 1.6 Hz, 1H), 1.15 – 1.01 (m, 42H). ^{13}C-NMR (100 MHz, CDCl$_3$): δ 185.1 (d, $J_{C,F}$ = 29.5 Hz), 106.2 (d, $J_{C,F}$ = 3.1 Hz), 101.3 (d, $J_{C,F}$ = 1.3 Hz), 100.8 (d, $J_{C,F}$ = 8.9 Hz), 91.2 (d, $J_{C,F}$ = 1.3 Hz), 90.5 (dd, $J_{C,F}$ = 200.6, 3.3 Hz), 83.9 (dd, $J_{C,F}$ = 185.9, 2.3 Hz), 71.2 (at, $J_{C,F}$ = 21.4 Hz), 18.6, 18.5, 11.09, 11.06. ^{19}F-NMR (376 MHz CDCl$_3$): δ -190.41 (d, $J_{H,F}$ = 3.4 Hz), -227.23 (d, $J_{H,F}$ = 3.3 Hz). ESI-HRMS-TOF found 485.3072, calculated 485.3077 for $(M+H)^+$. NP-HPLC R_t= 2.98 min, 98 %.

4-Fluoro-5-(fluoromethyl)-5-hydroxy-1,7-bis(triisopropylsilyl)hepta-1,6-diyn-3-one (92b)

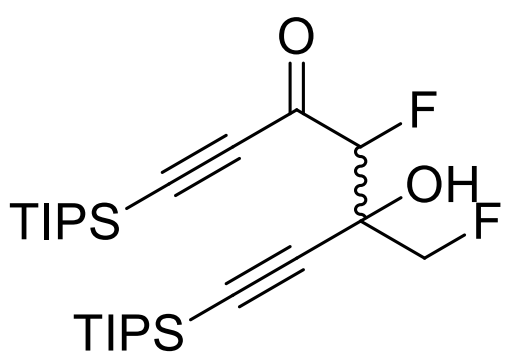

Also isolated from above method A (24 mg, 7 %).

^{1}H-NMR (400 MHz CDCl$_3$): δ 4.91 (d, $J_{H,F}$ = 47.5 Hz, 1H), 4.72 (dd, $J_{H,F}$ = 46.6, 9.5 Hz, 1H), 4.56 (ddd, $J_{H,F}$ = 47.2, 9.5, $J_{H,H}$ = 3.1 Hz, 1H), 3.22 (s, 1H), 1.23 – 1.00 (m, 42H). ^{13}C-NMR (100 MHz, CDCl$_3$): δ 181.4 (d, $J_{C,F}$ = 29.7 Hz), 104.2 (d, $J_{C,F}$ = 2.9 Hz), 101.8, 100.6, 92.7 (dd, $J_{C,F}$ = 197.2, 2.2 Hz), 91.7 (s), 84.0 (dd, $J_{C,F}$ = 183.2, 3.0 Hz), 71.2 (at, $J_{C,F}$ = 21.2 Hz), 18.61, 18.55, 11.11, 11.10. ^{19}F-NMR (376 MHz CDCl$_3$): δ -196.1, -224.1. ESI-HRMS-TOF found 485.3075, calculated 485.3077 for $(M+H)^+$. NP-HPLC R_t= 3.03 min, 96 %.

2-Fluoro-1-morpholinoethanone (**93**)

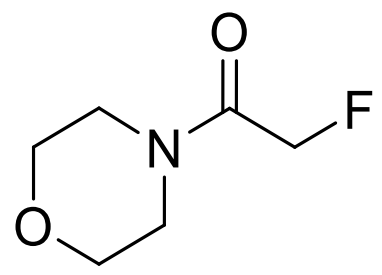

Morpholine (1.80 mL, 20.6 mmol, 2 eq.) and ethylfluoroacetate (1.00 mL, 10.3 mmol, 1 eq.) were heated together to 70 °C for 24 hours then the reaction mixture was cooled and diluted with HCl (aq., 4 M, 5 mL) and the resulting mixture was extracted with dichloromethane (3 × 10 mL). The combined organic layers were dried over anhydrous magnesium sulfate, filtered, and evaporated to give the product as a yellow oil (1.50 g, 99 %). The analytical data agreed with literature values.[23]

^{1}H-NMR (400 MHz, $CDCl_3$) δ 4.98 (d, $J_{H,F}$ = 47.2 Hz, 2H), 3.74 – 3.66 (m, 4H), 3.66 – 3.59 (m, 2H), 3.51 – 3.40 (m, 2H). ^{13}C-NMR (100 MHz $CDCl_3$) δ 165.5 (d, $J_{C,F}$ = 17.7 Hz), 80.2 (d, $J_{C,F}$ = 180.7 Hz), 66.9, 66.7, 45.4. 42.4. ESI-MS *m/z* = 148.2 $(M+H)^+$.

2-Bromo-*N*-methoxy-*N*-methylacetamide (**94**)

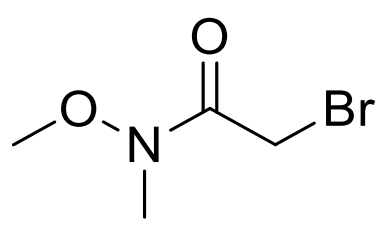

Bromoacetyl chloride (2.0 mL, 24.0 mmol, 1.2 eq.) was added dropwise to a solution of potassium carbonate (6.09 g, 44.0 mmol, 2.2 eq.) and hydroxlyamine hydrochloride (1.95 g, 20.0 mmol, 1 eq.) in water : diethyl ether (1:1, 40 mL). The reaction was stirred at room temperature for 1 hour then separated and the aqueous layer was further extracted with diethyl ether (3 × 20 mL). The combined organic layers were dried over anhydrous sodium sulfate, filtered, and evaporated to give the product as a clear oil (3.05 g, 84 %). The analytical data agreed with literature values.[24]

^{1}H-NMR (400 MHz $CDCl_3$): δ 3.98 (s, 2H), 3.75 (s, 3H), 3.20 (s, 3H). ^{13}C-NMR (100 MHz, $CDCl_3$): δ 167.7, 61.7, 40.9, 32.6, 25.2. ESI-MS *m/z* = 182.0 (100%, $M+H)^+$, 184.0 (87 %, $M+H)^+$. RP-HPLC R_t= 3.72 min, 99 %.

2-Fluoro-*N*-methoxy-*N*-methylacetamide (**95**)

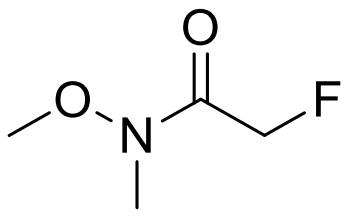

2-Bromo-*N*-methoxy-*N*-methylacetamide (**94**) (200 mg, 1.10 mmol, 1 eq.), potassium fluoride (255 mg, 4.40 mmol, 4 eq.) and 18-crown-6 (145 mg, 0.55 mmol, 0.5 eq.) were added to acetonitrile and the resulting mixture was heated to 80 °C for 19 hours. The reaction was then cooled and diluted with water (20 mL) and the resulting mixture was extracted with ethyl acetate (4 × 15 mL). The combined organic layers were dried over anhydrous magnesium sulfate, filtered, and evaporated to give the product as a yellow oil (122 mg, 91 %).

^{1}H-NMR (400 MHz $CDCl_3$): δ 5.06 (d, $J_{H,F}$ = 47.5 Hz, 2H), 3.68 (s, 3H), 3.20 (s, 3H). ^{13}C-NMR (100 MHz, $CDCl_3$): δ 168.6 (d, $J_{C,F}$ = 17.1 Hz), 78.8 (d, $J_{C,F}$ = 175.6 Hz), 61.7, 32.4. ESI-MS *m/z* = 122.2 $(M+H)^+$. ESI-HRMS-TOF found 122.0616, calculated 122.0612 for $(M+H)^+$.

RP-HPLC R_t= 2.84 min, 95 %.

***N*-(2-Azidoethyl)-3-(Thiophen-3-yl)benzylamine (96)**

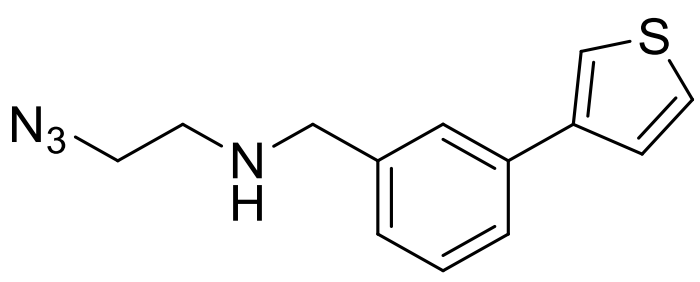

3-(3-(Thiophen-3-yl)benzylamino)ethanol (**46**) (213 mg, 0.913 mmol, 1 eq.) was added to thionyl chloride (2.5 mL) and the resulting mixture was heated to 70 °C for 1 hour. The reaction was then cooled and evaporated to dryness and co-evaporated with toluene to give an off white solid which was diluted with DMF (4 mL). Sodium azide (594 mg, 9.13 mmol, 10 eq.) was added and the solution was heated to 100 °C and stirred for 2 hours. The solution was then diluted with NaOH (aq., 1 M, 20 mL) and the resulting mixture was extracted with ethyl acetate (3 × 20 ml). The combined organic layers were dried over anhydrous magnesium sulfate, filtered, and evaporated to give a residue which was purified by

column chromatography in 100:1 hexane : triethylamine to 50:50:1 hexane : ethyl acetate : triethylamine to give the product as a clear oil (67 mg, 28 %).

^{1}H-NMR (400 MHz, $CDCl_3$) δ 7.60 – 7.57 (m, 1H), 7.52 – 7.48 (m, 1H), 7.47 (dd, *J* = 2.8, 1.5 Hz, 1H), 7.43 – 7.34 (m, 3H), 7.28 – 7.24 (m, 1H), 3.87 (s, 2H), 3.48 – 3.41 (m, 2H), 2.90 – 2.83 (m, 2H). ^{13}C-NMR (100 MHz, $CDCl_3$) δ 142.4, 140.6, 136.2, 129.1, 127.0, 126.5, 126.3, 126.3, 125.4, 120.5, 53.7, 51.6, 48.1. ESI-MS *m/z* = 259.1 (16 %, M+H)$^+$; 173.1 (100 %, M-$N_4C_2H_5$)$^+$. ESI-HRMS-TOF found 259.1021, calculated 259.1012 for (M+H)$^+$. RP-HPLC R_t= 6.43 min, 95 %.

***N*1-(*N*-(3-(Thiophen-3-yl)benzyl)-2-aminoethyl)-4-(fluoroacetyl)-1,2,3-triazole (97)**

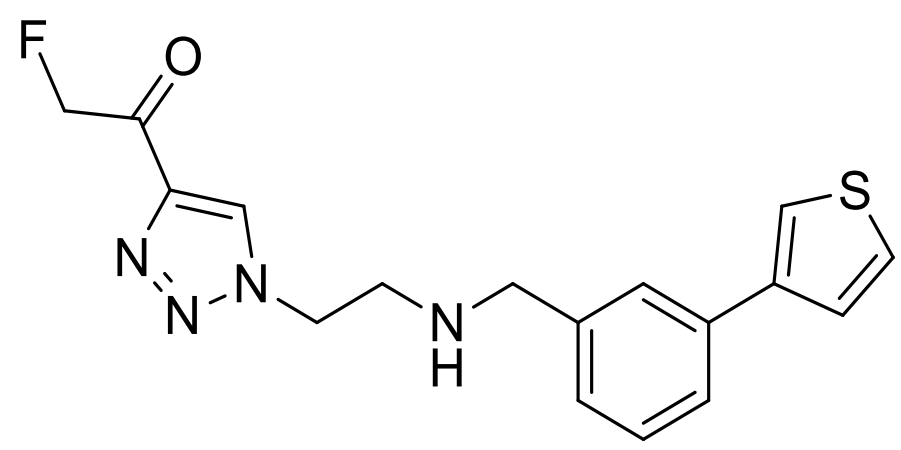

To a solution of 1-fluoro-4-(triisopropylsilyl)but-3-yn-2-one (**152**) (0.22 M in MeOH, 0.52 mL, 0.12 mmol, 1 eq.) in MeCN (5 mL) under nitrogen at room temperature was added acetic acid (7 μL, 0.13 mmol, 1.1 eq.) and tetrabutylammonium fluoride (1 M, 130 μL, 0.13 mmol, 1.1 eq.) sequentially and the reaction was stirred at room temperature t for 15 mins. Copper(I) bromide.dimethylsulfide complex (2 mg, 0.01 mmol, 0.1 eq.), TBTA (~1 mg, cat.) and *N*-(2-azidoethyl)-3-(thiophen-3-yl)benzylamine (**96**) (30 mg, 0.12 mmol, 1 eq.) were then added in order and the reaction was stirred at room temperature for a further 16 hours. The reaction was then diluted with water/brine (2:1, 15 mL) and the resulting mixture was extracted with ethyl acetate (3 × 15 mL). The combined organic layers were then dried over anhydrous magnesium sulfate, filtered, and evaporated to give the crude

as a brown solid. Purification by column chromatography in a gradient of 50:50:1 ethyl acetate : hexane : triethylamine to 95:5:1 ethyl acetate : methanol : triethylamine gave the product as a white solid (24 mg, 59 %).

^{1}H-NMR (400 MHz $CDCl_3$): δ 8.30 (s, 1H), 7.51 – 7.46 (m, 2H), 7.45 (dd, J = 2.9, 1.4 Hz, 1H), 7.41 – 7.32 (m, 3H), 7.18 (d, J = 7.2 Hz, 1H), 5.63 (d, $J_{H,F}$ = 47.0 Hz, 2H), 4.54 – 4.46 (m, 2H), 3.84 (s, 2H), 3.20 – 3.12 (m, 2H). ^{13}C-NMR (100 MHz, $CDCl_3$): δ 187.7 (d, $J_{C,F}$ = 16.7 Hz), 144.5, 142.1, 140.1, 136.3, 129.2, 127.0, 126.9, 126.5, 126.4, 126.2, 125.6, 120.6, 83.56 (d, $J_{C,F}$ = 181.2 Hz), 53.5, 51.0, 48.1. ESI-MS m/z = 345.1 $(M+H)^+$. ESI-HRMS-TOF found 345.1176, calculated 345.1180 for $(M+H)^+$. RP-HPLC R_t= 6.88 min, 95 %.

***N*1-Benzyl-4-(fluoroacetyl)-1,2,3-triazole (98)**

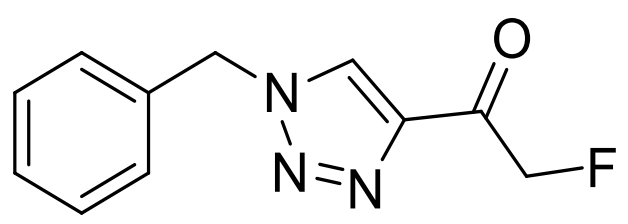

To a solution of 1-fluoro-4-(triisopropylsilyl)but-3-yn-2-one (**91**) (0.22 M in MeOH, 1.00 mL, 0.22 mmol, 1 eq.) in MeCN (5 mL) under nitrogen at room temperature was added acetic acid (14 μL, 0.24 mmol, 1.1 eq.) and tetrabutylammonium fluoride (1 M, 240 μL, 0.24 mmol, 1.1 eq.) in order and the reaction was stirred at room temperature for 15 mins. Copper(I) bromide.dimethylsulfide complex (5 mg, 0.02 mmol, 0.1 eq.), TBTA (~1 mg, cat.) and benzyl azide (30 mg, 0.22 mmol, 1 eq.) were then added in order and the reaction was stirred at room temperature for a further 16 hours. The reaction was then diluted with water/brine (2:1, 15 mL) and the resulting mixture was extracted with ethyl acetate (3 × 15 mL). The combined organic layers were then dried over anhydrous magnesium sulfate, filtered, and evaporated to give the

crude as a brown solid. Purification by column chromatography in a gradient of 75:25 hexane : ethyl acetate to 65:35 hexane : ethyl acetate gave the product as a white solid (26 mg, 53 %). ^{1}H-NMR (400 MHz $CDCl_3$): δ 8.06 (s, 1H), 7.45 – 7.38 (m, 3H), 7.34 – 7.27 (m, 2H), 5.66 (d, $J_{H,F}$ = 47.0 Hz, 2H), 5.58 (s, 2H). ^{13}C-NMR (100 MHz, $CDCl_3$): δ 187.7 (d, $J_{C,F}$ = 16.6 Hz), 145.0, 133.4, 129.6, 129.5, 128.5, 126.0 (d, $J_{C,F}$ = 3.6 Hz), 83.6 (d, $J_{C,F}$ = 181.4 Hz), 54.8. ESI-MS *m/z* = 220.1 $(M+H)^+$. ESI-HRMS-TOF found 220.0887, calculated 220.0881 for $(M+H)^+$. RP-HPLC R_t= 6.55 min, 98 %.

N1-(N-(3-(Thiophen-3-yl)benzyl)-2-aminoethyl)-4-(acetyl)-1,2,3-triazole (99)

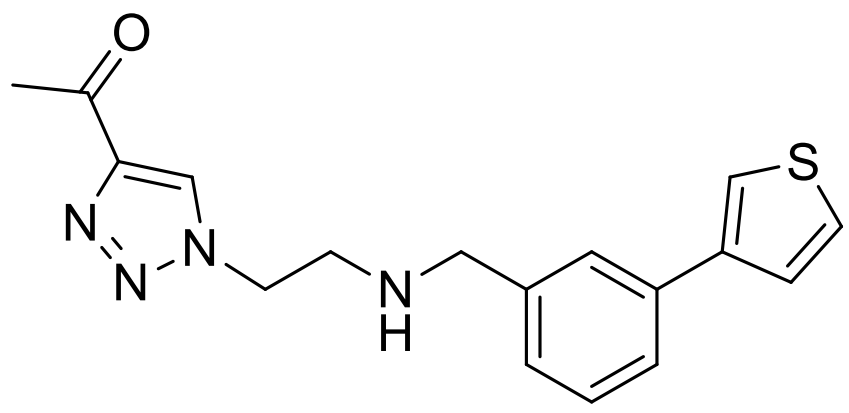

To a solution of 2-acetyl(triisopropylsilyl)acetylene (**101**) (0.74 mL, 0.17 M in MeOH, 0.12 mmol, 1 eq.) in MeCN (5 mL) under nitrogen at room temperature was added acetic acid (8 μL, 0.14 mmol, 1.1 eq.) and tetrabutylammonium fluoride (1 M, 140 μL, 0.14 mmol, 1.1 eq.) in order and the reaction was stirred at room temperature for 15 mins. Copper(I) bromide.dimethylsulfide complex (3 mg, 0.01 mmol, 0.1 eq.), TBTA (~1 mg, cat.) and *N*-(2-azidoethyl)-3-(thiophen-3-yl)benzylamine (**96**) (32 mg, 0.12 mmol, 1 eq.) were then added in order and the reaction was stirred at room temperature for a further 16 hours. The reaction was then diluted with water/brine (2:1, 15 mL) and the resulting mixture was extracted with ethyl acetate (3 × 15 mL). The combined organic layers were then dried over anhydrous magnesium sulfate, filtered, and evaporated to give the crude as a brown solid. Purification by column chromatography in a gradient of 75:25 hexane : ethyl acetate to 65:35 hexane : ethyl acetate gave the product as a white solid (26 mg, 53 %). Purification by column chromatography in a gradient of 70:30 ethyl acetate : hexane to 95:5 ethyl acetate : methanol gave the product as a clear oil (25 mg, 62 %).

^{1}H-NMR (400 MHz $CDCl_3$): δ 8.17 (s, 1H), 7.53 – 7.42 (m, 3H), 7.42 – 7.31 (m, 3H), 7.18 (bd, J = 7.6 Hz, 1H), 4.49 (t, J = 5.7 Hz, 2H), 3.84 (s, 2H), 3.17 (t, J = 5.8 Hz, 2H), 2.67 (s, 3H). ^{13}C-NMR (100 MHz, $CDCl_3$): δ 193.0, 148.2, 142.1, 139.9, 136.3, 129.2, 127.0, 126.4, 126.4, 126.3, 126.3, 125.6, 120.7, 53.5, 50.7, 48.1, 27.3. ESI-MS *m/z* = 173.1 (100 %, M-$C_6H_9N_4O_1$)$^+$, 327.1 (66 %, M+H)$^+$. ESI-HRMS-TOF found 327.1284, calculated 327.1274 for $(M+H)^+$. RP-HPLC R_t= 6.45 min, 95 %.

_N_-Methoxy-_N_-methylacetamide (100)

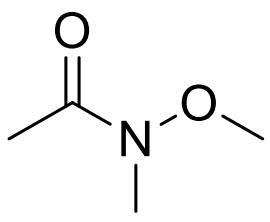

Acetyl chloride (874 µL, 12.3 mmol, 1.2 eq.) was added dropwise to a solution of potassium carbonate (3.12 g, 22.6 mmol, 2.2 eq.) and hydroxlyamine hydrochloride (1.00 g, 10.3 mmol, 1 eq.) in water : diethyl ether (1:1, 20 mL) at 0 °C. The reaction was then warmed to room temperature and stirred for 1 hour, the phases were separated and the aqueous layer further extracted with diethyl ether (2 × 10 mL). The combined organic layers were dried over anhydrous sodium sulfate, filtered, and evaporated to give the product as a clear oil (536 mg, 51 %). The analytical data agreed with literature values.[25]

^{1}H-NMR (400 MHz $CDCl_3$): δ 3.67 (s, 3H), 3.16 (s, 3H), 2.11 (s, 3H). ^{13}C-NMR (100 MHz, $CDCl_3$): δ 172.1, 77.2, 61.2, 32.1, 19.9. ESI-MS *m/z* = 104.1 $(M+H)^+$. RP-HPLC R_t= 3.14 min, >99 %

2-Acetyl(triisopropylsilyl)acetylene (101)

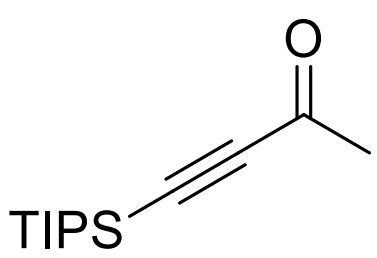

Triisopropylsilylacetylene (446 µL, 2.20 mmol, 1 eq.) was added to dry THF (5 mL) and the resulting solution was cooled to -78 °C under nitrogen, followed by addition of *n*-butyl lithium (1.45 M, 1.51 mL, 2.20 mmol, 1 eq.). The mixture was stirred at -78 °C for 30

mins. *N*-Methoxy-*N*-methylacetamide (**100**) (250 mg, 2.42 mmol, 1.1 eq.) was added to dry THF (2 mL) and the resulting solution was cooled to -78 °C under nitrogen followed by dropwise cannula addition of the acetylide mixture over 10 mins. The reaction was stirred at -78 °C for 1 hour then warmed at room temp. for 10 mins. The reaction was then quenched with HCl (aq., 1 M, 10 mL) and the resulting mixture was extracted with diethyl ether (3 × 15 mL). The combined organic layers were dried over anhydrous magnesium sulfate, filtered, and evaporated to give a yellow oil (470 mg) which was diluted with methanol and used without further purification. Concentration was calculated from the ^{1}H-NMR integrations of signals at 2.36, 3.18 and 1.85 ppm indicative of 4-(triisopropylsilyl)but-3-yn-2-one (**101**), *N*-methoxy-*N*-methylacetamide (**100**) and tetrahydrofuran respectively corresponding to a yield of 76%. Signals were consistent with literature values.[26] The solution was used without further purification in subsequent reactions.

11.Comparative ranked screening results of compounds 1-90

Screening was carried out via HSQC NMR spectroscopy by recording ^{15}N-^{1}H HSQC spectra of ^{15}N *Ec*DsbA in apo state as well as 1 mM compound. Compounds were then assessed by chemical shift perturbations of *Ec*DsbA amides, similar to that of our previous reports.[27] Fragment library screening hits and initial purchased compounds:

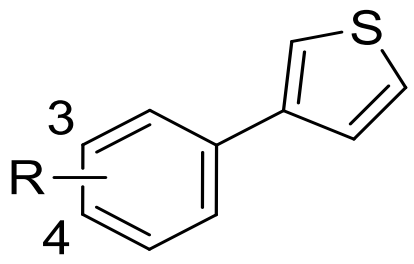

3-		4-	
**1**[a]	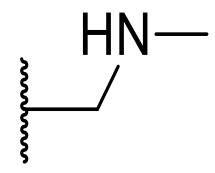	**3**[a]	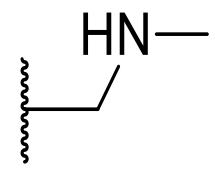
**4**	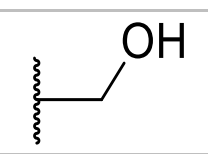		
**8**	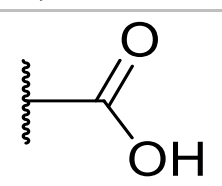	**9**	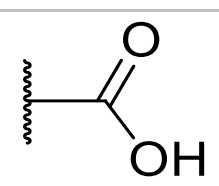

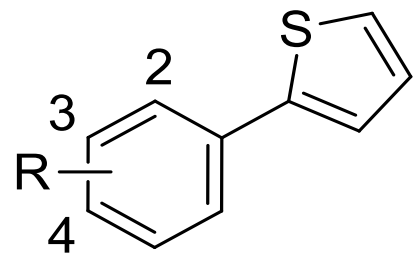

2-		3-		4-	
		**2**[a]	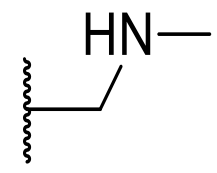		
**5**	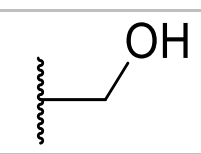	**6**	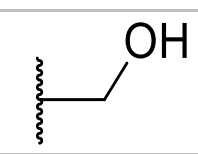	**7**	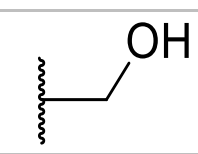
**10**	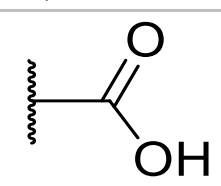	**11**	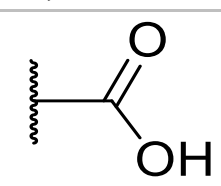	**12**	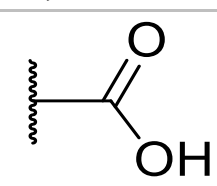
**13**	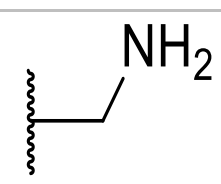	**14**	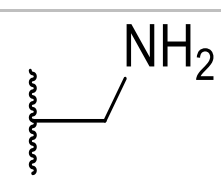	**15**	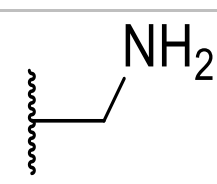
**16**	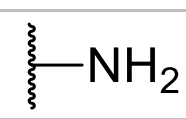	**17**	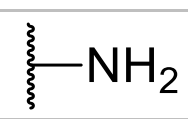		

[a] Initial fragment library hits. All purchased compounds above showed weaker NMR perturbations than the corresponding parent fragments **1** and **2**.

Table S1. HSQC Screening results of synthesized compounds

Compound number	SMILES	Number of Δ δ [a]> 0.02	Average Δ δ [a] of Δ δ > 0.02	Max Δ δ [a]	Rank[b]
**1**	CNCC1=CC(=CC=C1)C1=CSC=C1	26	0.0259	0.0492	1
**18**	CCNCC1=CC=C(C2=CSC=C2)C=C1	40	0.032	0.1398	2
**19**	CC(C)(C)NCC1=CC=C(C2=CSC=C2)C=C1	45	0.030	0.1424	2
**20**	C1(C2=CC=C(CNC3CCCCC3)C=C2)=CSC=C1	57	0.0399	0.1682	2
**21**	C1(C2=CC=C(CNC3(C[C@@H](C4)C5)C[C@@H]5C[C@@H]4C3)C=C2)=CSC=C1	37	0.0305	0.1321	2
**22**	C1(C2=CC=C(CNCC3=CC=CC=C3)C=C2)=CSC=C1	28	0.0321	0.1023	1
**23**	C1(C2=CC=C(CNCCC3=CC=CC=C3)C=C2)=CSC=C1	32	0.0321	0.0933	2
**24**	COC1=CC=C(CNCC2=CC=C(C3=CSC=C3)C=C2)C=C1	8	0.0281	0.0563	0
**25**	C1(C2=CC=C(CN3CCCCC3)C=C2)=CSC=C1	30	0.0327	0.115	1
**26**	C1(C2=CC=C(CN3CCOCC3)C=C2)=CSC=C1	94	0.0296	0.1234	1
**27**	CN(C)CC1=CC=C(C2=CSC=C2)C=C1	51	0.0222	0.0459	1
**28**	NCC1=CC=C(C=C1)C1=CSC=C1	15	0.0223	0.0344	0
**29**	OCCNCC1=CC=C(C=C1)C1=CSC=C1	27	0.0278	0.0603	1
**30**	CCOC(=O)CNCC1=CC=C(C=C1)C1=CSC=C1	11	0.0232	0.0314	1
**31**	C(NCC1=CC=CN=C1)C1=CC=C(C=C1)C1=CSC=C1	26	0.0322	0.0917	1
**32**	NCC1=CC=CC(=C1)C1=CSC=C1	20	0.0228	0.0393	1
**33**	C(NC1CCCCC1)C1=CC(=CC=C1)C1=CSC=C1	37	0.0292	0.085	1
**34**	NC(=O)CNCC1=CC(=CC=C1)C1=CSC=C1	11	0.0235	0.0376	0
**35**	C(N1CCC1)C1=CC=C(C=C1)C1=CSC=C1	26	0.0259	0.0657	1
**36**	COC(=O)CCNCC1=CC(=CC=C1)C1=CSC=C1	35	0.030	0.0821	1
**37**	COC(=O)CCCCCNCC1=CC(=CC=C1)C1=CSC=C1	31	0.0323	0.0948	1
**38**	O=C1CCCN1CC1=CC(=CC=C1)C1=CSC=C1	22	0.0384	0.0983	1
**39**	O=C1CCCCN1CC1=CC(=CC=C1)C1=CSC=C1	32	0.0401	0.1031	2
**40**	[Li]OC(=O)CNCC1=CC(=CC=C1)C1=CSC=C1	10	0.0248	0.0446	0
**41**	[Li]OC(=O)CCNCC1=CC(=CC=C1)C1=CSC=C1	17	0.0227	0.0408	0
**42**	[K]OC(=O)CCCNCC1=CC(=CC=C1)C1=CSC=C1	6	0.0186	0.0243	0
**43**	[K]OC(=O)CCCCNCC1=CC(=CC=C1)C1=CSC=C1	19	0.0265	0.0472	1
**44**	[Li]OC(=O)CCCCCNCC1=CC(=CC=C1)C1=CSC=C1	14	0.0254	0.0392	0
**45**	NC(=O)CCNCC1=CC=CC(=C1)C1=CSC=C1	21	0.0227	0.0383	0

Compound number	SMILES	Number of Δ δ [a]> 0.02	Average Δ δ [a] of Δ δ > 0.02	Max Δ δ [a]	Rank[b]
**46**	OCCNCC1=CC(=CC=C1)C1=CSC=C1	36	0.0294	0.0797	1
**47**	OCCCNCC1=CC(=CC=C1)C1=CSC=C1	23	0.0284	0.0646	1
**48**	OCCCCNCC1=CC=CC(=C1)C1=CSC=C1	22	0.0264	0.0572	1
**49**	OCCCCCNCC1=CC(=CC=C1)C1=CSC=C1	30	0.0257	0.0731	1
**50**	CNCC1=CC(=CC=C1)C1=CC=CN1C	15	0.0266	0.0429	1
**51**	CNCC1=CC(=CC=C1)C1=COC=C1	22	0.0264	0.0485	1
**52**	CNCC1=CC(=CC=C1)C1=CC=CO1	13	0.0283	0.0431	0
**53**	CNCC1=CC(=CC=C1)C1=C(C)ON=C1C	18	0.0261	0.049	1
**54**	CNCC1=CC(=CC=C1)C1=CNN=C1	4	0.0203	0.0242	0
**55**	CNCC1=CC(=CC=C1)C1=CC=NC=C1	19	0.0222	0.0421	1
**56**	CNCC1=CC(=CC=C1)C1=CC=C(O)C=C1	16	0.0269	0.0425	1
**57**	CNCC1=CC(=CC=C1)C1=CC=CC(O)=C1	26	0.0276	0.0526	1
**58**	CNCC1=CC=CC(=C1)C1=CC(=CC=C1)C(F)(F)F	35	0.0258	0.0653	1
**59**	CNCC1=NC(=CC=C1)C1=CSC=C1	12	0.0243	0.039	0
**60**	CNCC1=CN=C(C=C1)C1=CSC=C1	8	0.0201	0.0294	0
**61**	CNCC1=NC=C(C=C1)C1=CSC=C1	9	0.0215	0.0388	0
**62**	CNCC1=CC(=CN=C1)C1=CSC=C1	6	0.0195	0.026	0
**63**	CNCC1=CC(=NC=C1)C1=CSC=C1	8	0.0224	0.0341	0
**64**	CNCC1=CC(=CS1)C1=CC=CC=C1	30	0.025	0.0653	1
**65**	CNCC1=CC=C(S1)C1=CC=CC=C1	21	0.0264	0.0558	0
**66**	NC(=O)C1=CC=CC(=C1)C1=CSC=C1	0	0	0.0124	0
**67**	CNC(=O)C1=CC=CC(=C1)C1=CSC=C1	26	0.0255	0.0519	1
**68**	O=C(NC1CCCCC1)C1=CC=CC(=C1)C1=CSC=C1	0	0	0.0136	0
**69**	CC(=O)NCC1=CC=CC(=C1)C1=CSC=C1	18	0.0236	0.0392	1
**70**	CN(CC1=CC=CC(=C1)C1=CSC=C1)C(C)=O	37	0.034	0.0832	2
**71**	CC(=O)N(CC1=CC=CC(=C1)C1=CSC=C1)C1CCCCC1	17	0.0273	0.0531	1
**72**	COC(=O)[C@H]1CC[C@H](CNCC2=CC(=CC=C2)C2=CSC=C2)CC1	32	0.0257	0.0775	1
**73**	NC(=O)[C@H]1CC[C@H](CNCC2=CC(=CC=C2)C2=CSC=C2)CC1	18	0.0251	0.0432	0
**74**	[Li]OC(=O)[C@H]1CC[C@H](CNCC2=CC=CC(=C2)C2=CSC=C2)CC1	19	0.0207	0.0417	1
**75**	OC[C@H]1CC[C@H](CNCC2=CC(=CC=C2)C2=CSC=C2)CC1	28	0.0279	0.0591	1
**76**	C(NCC1=CC(=CC=C1)C1=CSC=C1)C1CCCCC1	26	0.0346	0.0638	1
**77**	COC(=O)[C@H]1CC[C@@H](CC1)NCC1=CC=CC(=C1)C1=CSC=C1	45	0.0319	0.0899	1
**78**	OC1CCC(CC1)NCC1=CC=CC(=C1)C1=CSC=C1	25	0.0243	0.05	0
**79**	C(NCC1=CC=CC=N1)C1=CC=CC(=C1)C1=CSC=C1	24	0.0293	0.0713	1
**80**	C(NCC1=CC=CC(=C1)C1=CSC=C1)C1=CC=CN=C1	23	0.0287	0.0817	1

Compound number	SMILES	Number of Δ δ [a] > 0.02	Average Δ δ [a] of Δ δ > 0.02	Max Δ δ [a]	Rank[b]
**81**	C(NCC1=CC=CC(=C1)C1=CSC=C1)C1=CC=NC=C1	23	0.0287	0.0855	1
	CN(CC1=CC=CC(=C1)C1=CSC=C1)C(=O)CNC(=O)OC(C)(C)C	12	0.0241	0.0362	1
**83**	CN(CC1=CC=CC(=C1)C1=CSC=C1)C(=O)CCNC(=O)OC(C)(C)C	9	0.0219	0.0298	1
**84**	CN(CC1=CC=CC(=C1)C1=CSC=C1)S(C)(=O)=O	16	0.0245	0.0386	1
**85**	CN(CC1=CC=CC(=C1)C1=CSC=C1)S(=O)(=O)C1=CC=C(C)C=C1	0	0	0.015	0
**86**	CN(CC1=CC=CC(=C1)C1=CSC=C1)C(=O)C(F)(F)F	15	0.0177	0.0222	1
**87**	CN(CC1=CC=CC(=C1)C1=CSC=C1)C(=O)C(C)(C)C	13	0.0289	0.049	1
**88**	CN(CC1=CC=CC(=C1)C1=CSC=C1)C(=O)C1=CC=CC=C1	9	0.0234	0.0421	0
**89**	CN(CC1=CC=CC(=C1)C1=CSC=C1)C(=O)CC1=CC=CC=C1	6	0.0214	0.028	0
**90**	CN(CC1=CC=CC(=C1)C1=CSC=C1)C(=O)CC1=CC=CC=N1	35	0.0397	0.111	2
**S26**	CC1(C)CC(CC(C)(C)N1)NCC1=CC=CC(=C1)C1=CSC=C1	26	0.028	0.061	1

[a] Δ δ is the weighted chemical shift perturbation (CSP) of amides in ^{15}N-^{1}H *Ec*DsbA from the apo sate to the presence of 1 mM compound. [b] Ranking using the previously outlined HSQC NMR ranking method[27] which scores compounds from 0 to 4 based on Δ δ for residues that are not pH or DMSO sensitive and in the active site groove of *Ec*DsbA.

12. X-ray crystallography of compounds

*Ec*DsbA-compound complexes were crystallized and structures were determined as previously described[27].

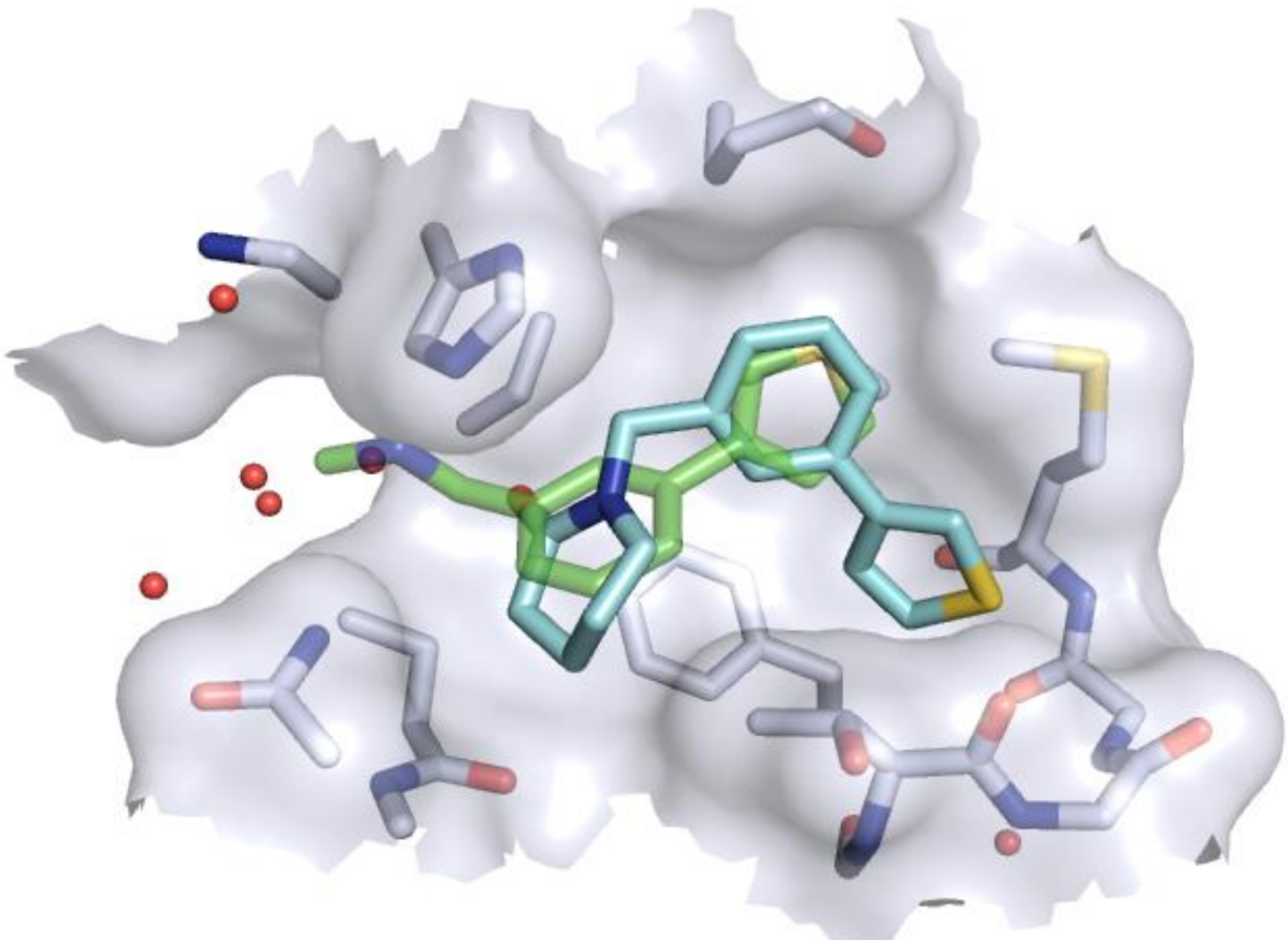

a)

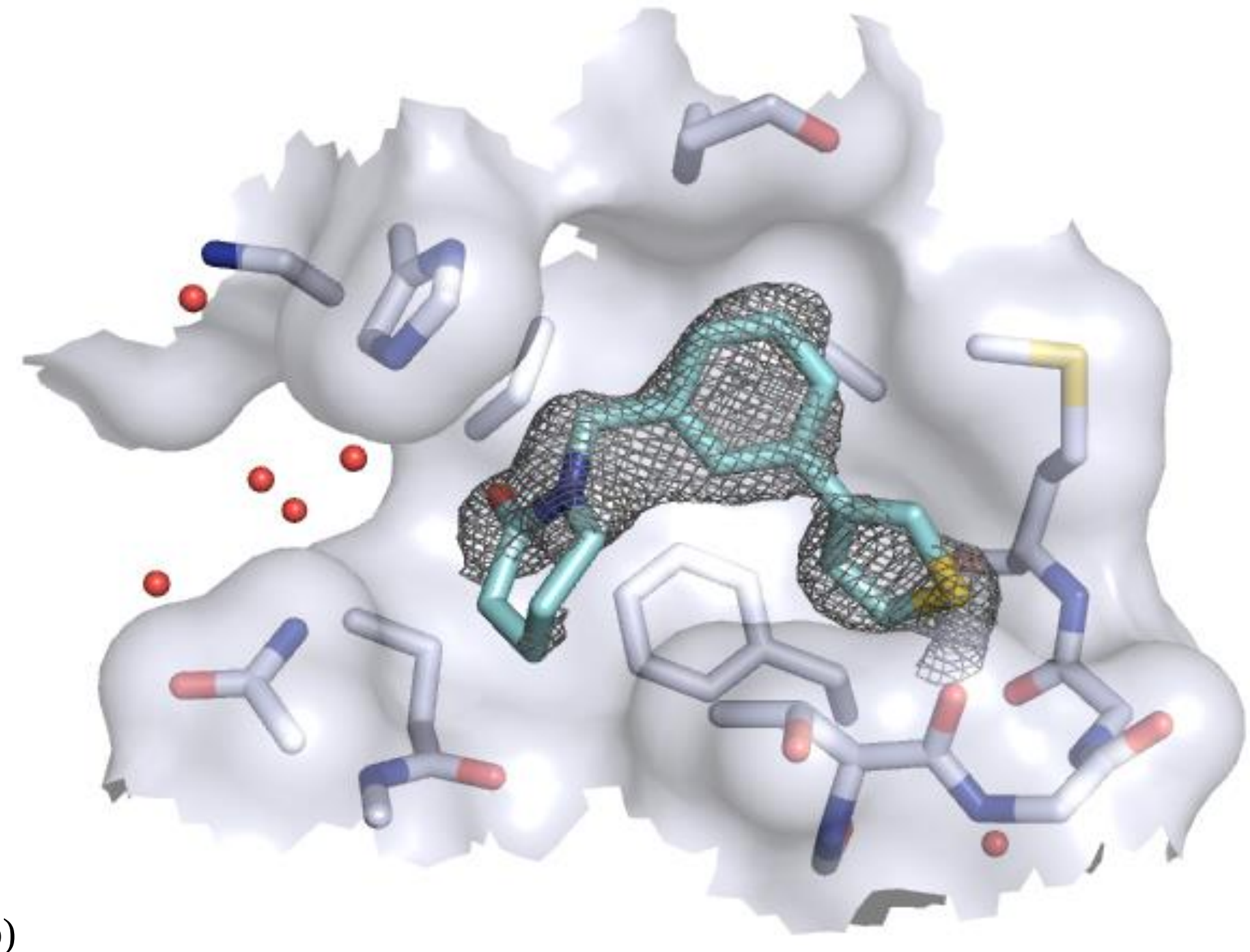

b)

Figure S1 a) Overlay of parent phenylthiophene fragment **1** (green) onto lactam **39** (blue, refined at 1.93 Å) b) lactam **39** (blue) with electron density ($\sigma = 1.1$). Atoms within 5 Å shown as surface and sticks (grey).

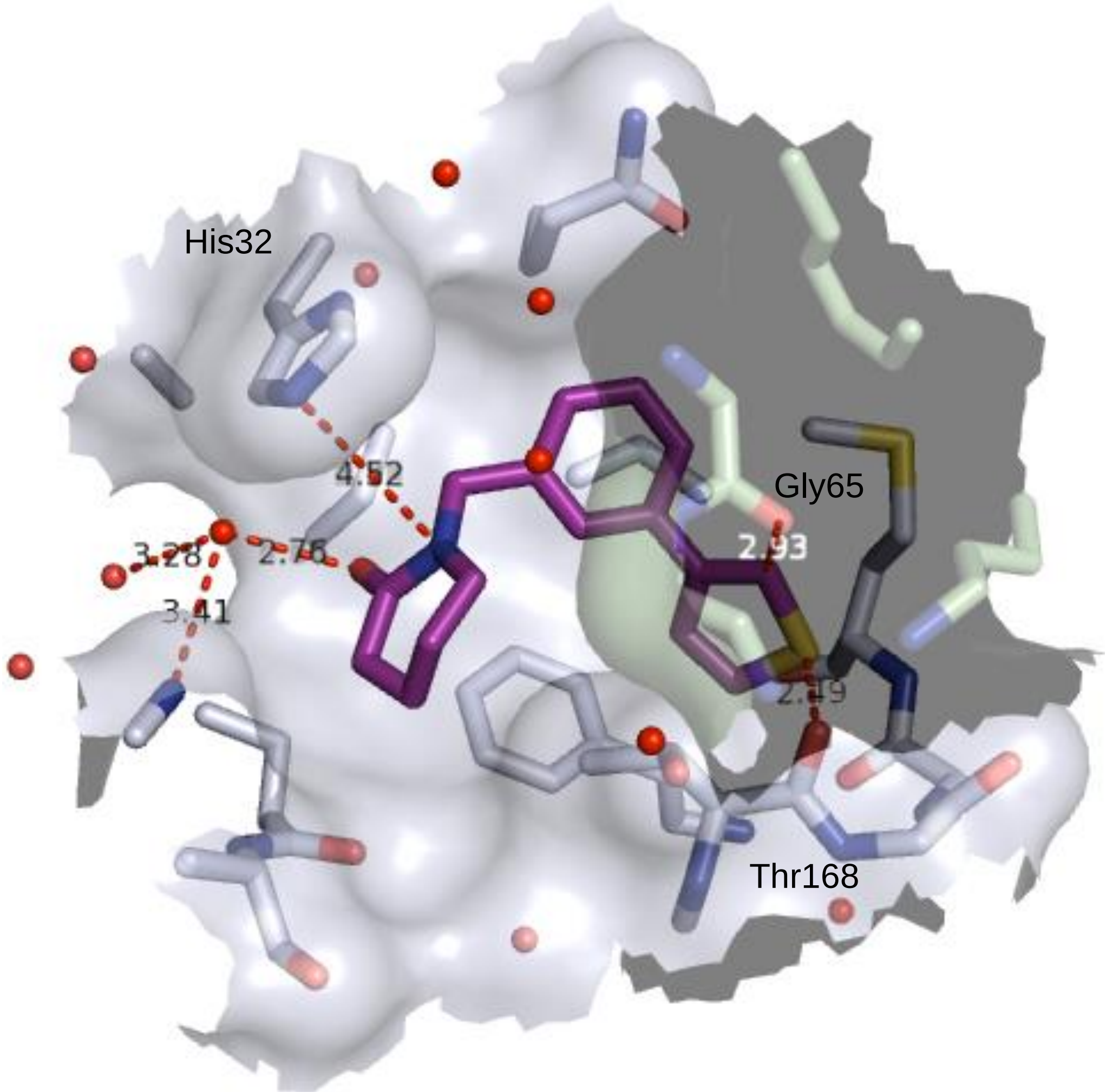

Figure S2 Binding site of lactam **39** (purple) to *Ec*DsbA molecule A (grey) and monomer B (green) (5 Å distance/close contacts from ligand) determined by X-ray crystallography with interactions labelled (Å).

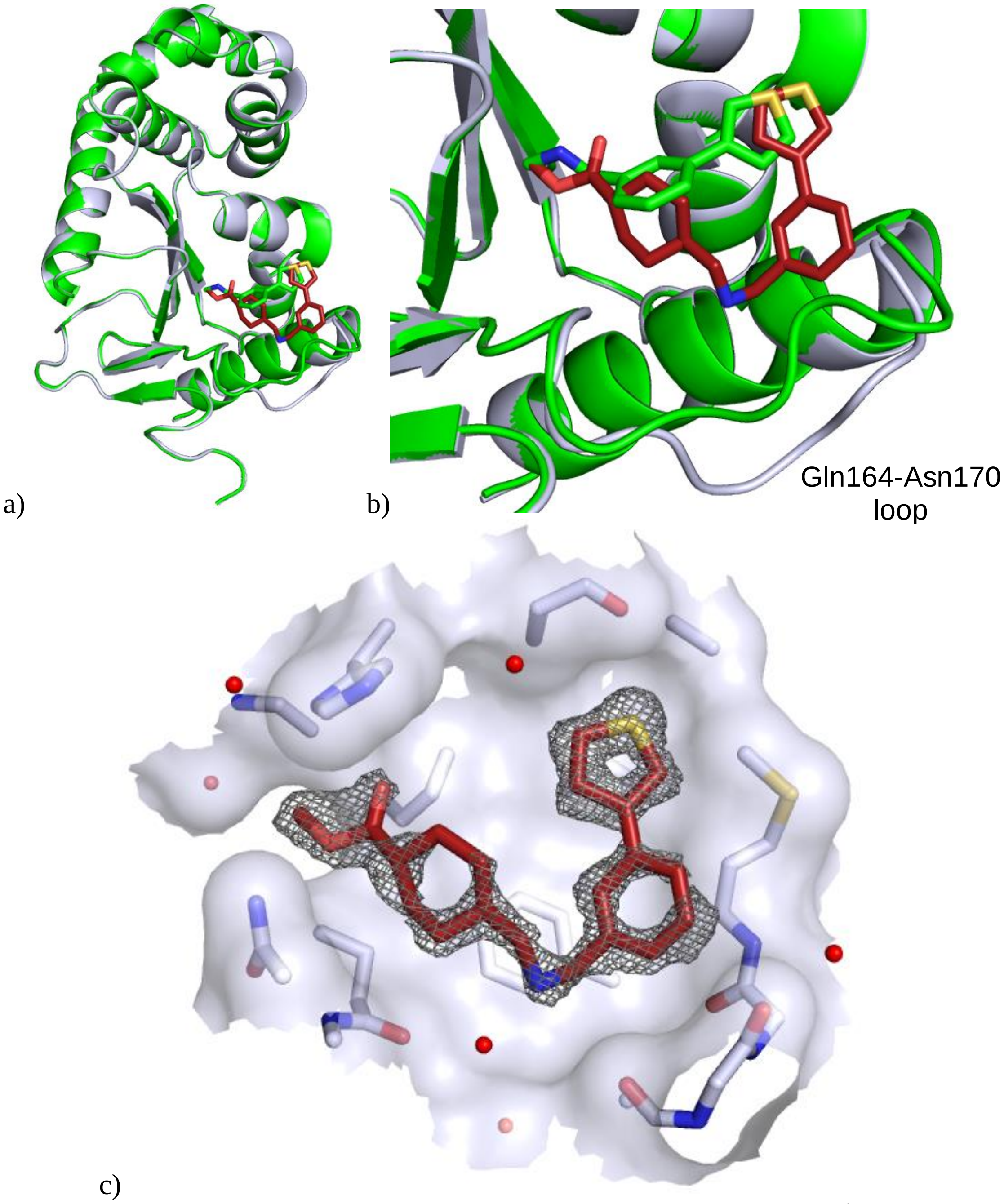

Figure S3 a) Overlay of cyclohexyl ester **72** (red and white, refined at 1.62 Å) and parent phenylthiophene **1** (green) with b) expansion. c) Electron density of cyclohexyl ester **72** (red, σ 1.1) with atoms within 5 Å shown as surface and sticks (white).

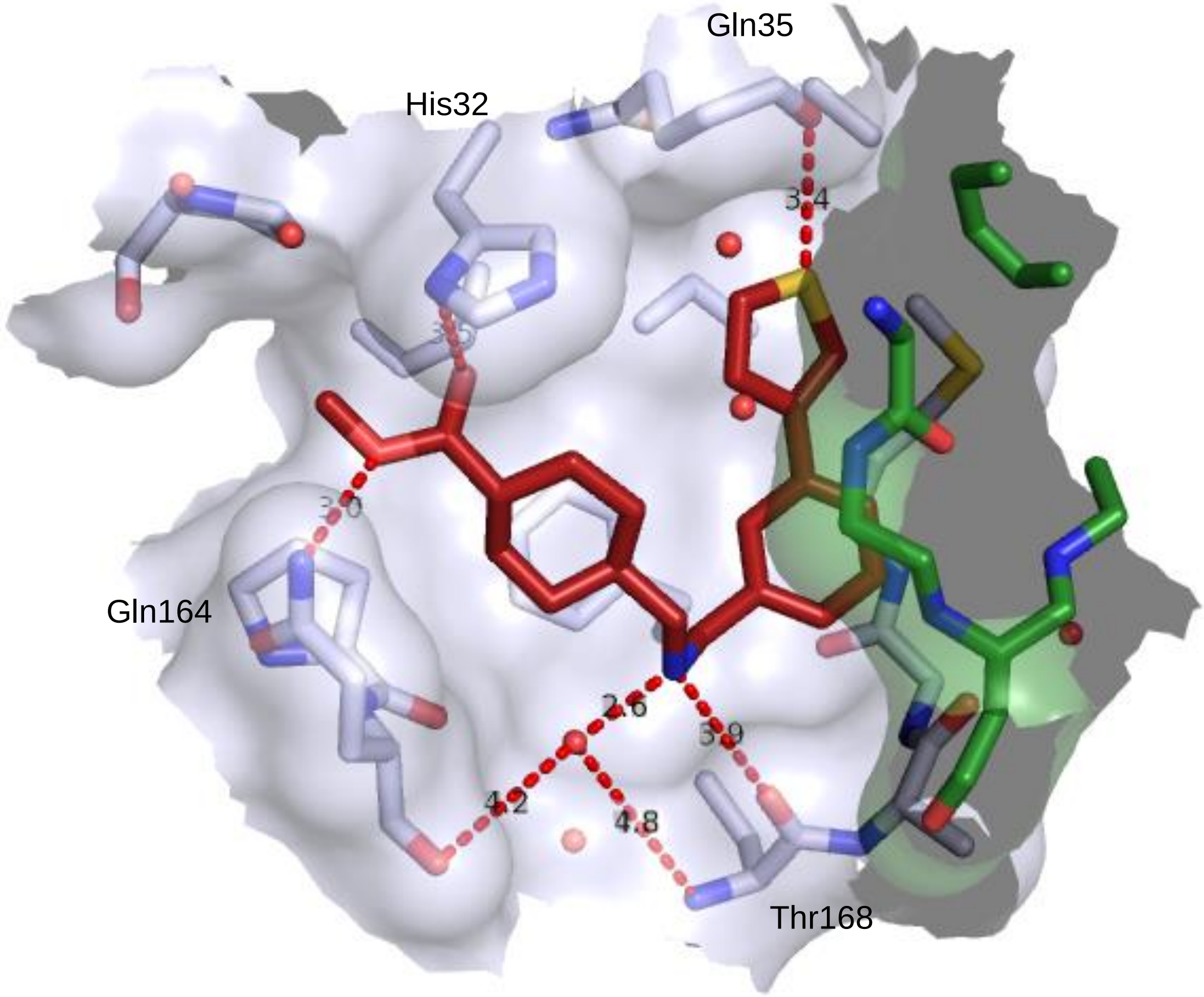

Figure S4 Binding site of cyclohexyl ester **72** (red) to *Ec*DsbA molecule A (grey) and monomer B (green) (atoms within 5 Å distance from ligand shown as surface and sticks) determined by X-ray crystallography with interactions labelled (Å).

Table S2. X-ray crystallography data collection and refinement statistics.

Data collection	39	38	72	1
PDB code	7S1D	7S1F	7S1L	7S1C
Space Group	*C*2	*C*2	*C*2	*C*2
Cell dimensions (Å) (a,b,c)	(115.97, 64.22, 74.36)	(116.13, 63.99, 74.33)	(116.21, 63.94, 74.67)	(116.20, 63.70, 74.26)
Angles	α=γ=90° β=126.04°	α=γ=90° β=126.10°	α=γ=90° β=126.39°	α=γ=90° β=126.11°
Resolution (Å)[a]	56.4 – 1.59 (1.68-1.59)	60.1 − 1.76 (1.86-1.76)	37.26− 1.62 (1.67-1.62)	50.0 − 1.95 (1.98-1.95)
Total number of observations	232207	169127	210687	119589
Number of unique observations	58965	43413	55151	32047
Multiplicity	3.9 (4.0)	3.9 (3.9)	3.8 (3.8)	3.7 (3.7)
Data Completeness (%)	99.3 (98.9)	99.3 (99.8)	99.0 (98.0)	99.9 (100.0)
$\langle I/\sigma_I \rangle$	20.0 (3.4)	20.3 (3.4)	15.6 (2.2)	21.6 (3.0)
R_{merge}(%)[b]	3.8 (40.2)	4.2 (39.7)	5.5 (58.4)	5.3 (39.6)
**Refinement**				
Resolution (Å)	33.0- 1.59 (1.61-1.59)38	56.5-1.76 (1.79-1.76)	34.4- 1.62 (1.65-1.62)	30.0-1.95 (1.98-1.95)
No. reflections	58959	43406	55150	32026
R_{work}[c]/ R_{free}[d]	0.16/0.19 (0.22/ 0.25)	0.16/0.20 (0.27/ 0.35)	0.16/0.19 (0.24/ 0.27)	0.17/0.21 (0.22/0.25)
No. atoms: Protein	3014	3036	3008	2972
No. atoms: Water	490	363	427	313
Wilson B	18.33	21.62	17.00	28.73
B factor (Å^2) – All	26.70	28.64	24.75	35.94
B factor (Å^2) – Water	37.80	36.87	35.20	41.26
B factor (Å^2) – Protein	24.93	27.66	23.26	35.37
R.m.s. deviations				
Bond lengths (Å)	0.011	0.012	0.016	0.007
Bond angles (°)	1.247	1.247	1.534	0.959
Ramachandran plot				
Residues in most favored/additionally allowed regions (%)	98.94/1.06	98.94/1.06	98.67/ 1.33	98.39/1.61
MolProbity Score (percentile)	1.24 (99^{7h})	1.11 (100th)	0.97 (100th)	1.19 (99th)

[a]Values in parentheses refer to the highest resolution shell.

[b]Agreement between intensities of repeated measurements of the same reflections can be defined as: $R_{merge} = \frac{\sum_{hkl}\sum_{i=1}^{n}|I_i(hkl) - \bar{I}(hkl)|}{\sum_{hkl}\sum_{i=1}^{n} I_i(hkl)}$ where $I_i(hkl)$ are individual values and $\bar{I}(hkl)$ is the mean value of the intensity of reflection *hkl*.

[c]$R_{fac} = \sum_h |F_o - F_c| / \sum_h|F_o|$, where F_o and F_c are the observed and calculated structure-factor amplitudes for each reflection "h".

[d]R_{free} was calculated with 5% of the diffraction data selected randomly and excluded from refinement.

13. References

1. Fulmer, G. R.; Miller, A. J. M.; Sherden, N. H.; Gottlieb, H. E.; Nudelman, A.; Stoltz, B. M.; Bercaw, J. E.; Goldberg, K. I. NMR chemical shifts of trace impurities: Common laboratory solvents, organics, and gases in deuterated solvents relevant to the organometallic chemist. *Organometallics* **2010,** 29, 2176-2179.
2. Studier, F. W. Protein production by auto-induction in high-density shaking cultures. *Protein Expression Purif.* **2005,** 41, 207-234.
3. Rathore, A. S.; Bilbrey, R. E.; Steinmeyer, D. E. Optimization of an osmotic shock procedure for isolation of a protein product expressed in E-coli. *Biotech. Prog.* **2003,** 19, 1541-1546.
4. Goddard, T. D.; Kneller, D. G. SPARKY 3. *University of California, San Francisco*.
5. Dubreuil, D. M.; Pipelier, M. G.; Pradere, J. P.; Bakkali, H.; Lepape, P.; Delaunay, T.; Tabatchnik, A. Pyridazine and pyrrole compounds, particularly nonlinear oligopyridazines, pyridazinylpyrroles and oligopyrroles, processes for their preparation and uses in medicine, materials, environmental, electronic and optics field. WO2008012440A2, 2008.
6. Querner, C.; Benedetto, A.; Demadrille, R.; Rannou, P.; Reiss, P. Carbodithioate-Containing Oligo- and Polythiophenes for Nanocrystals' Surface Functionalization. *Chemistry of Materials* **2006,** 18, 4817-4826.
7. Jordan, A. D.; Orsini, M. J.; Middleton, S. A.; Connolly, P. J.; Brenneman, D. E.; Pan, K.; Reitz, A. B. 8-(Heteroaryl)phenalkyl-1-Phenyl-1,3,8-triazaspiro4.5decan-4-ones as Opioid Receptor Modulators. *Medicinal Chemistry* **2005,** 1, 601-610.
8. Gros, L.; Lorente, S. O.; Jimenez; Yardley, V.; Rattray, L.; Wharton, H.; Little, S.; Croft, S. L.; Ruiz-Perez, L. M.; Gonzalez-Pacanowska, D.; Gilbert, I. H. Evaluation of Azasterols as Anti-Parasitics. *Journal of Medicinal Chemistry* **2006,** 49, 6094-6103.
9. McCubbin, J. A.; Maddess, M. L.; Lautens, M. Total Synthesis of Cryptophycin Analogues via a Scaffold Approach. *Organic Letters* **2006,** 8, 2993-2996.
10. Schneider, T. L.; Walsh, C. T.; O'Connor, S. E. Utilization of Alternate Substrates by the First Three Modules of the Epothilone Synthetase Assembly Line. *Journal of the American Chemical Society* **2002,** 124, 11272-11273.
11. Kohda, I.; Iwai, M.; Watanabe, M.; Arakawa, Y.; Fukaya, C.; Yokoyama, K.; Kohama, Y.; Mimura, T. Inhibitory effect of 2-(E-2-alkenoylamino)ethyl alkyl sulfides on gastric ulceration in rats. II. Structure and activity relationships of 2-(E-n or Z-n-decenoylamino)ethyl alkyl sulfides. *Chemical & Pharmaceutical Bulletin* **1991,** 39, 1546-50.
12. Kleemann, A.; Leuchtenberger, W.; Martens, J.; Weigel, H. Ein neuer Weg zu 4-Amino-buttersäureamid. *Angewandte Chemie* **1980,** 92, 640-640.
13. Salmon-Chemin, L.; Buisine, E.; Yardley, V.; Kohler, S.; Debreu, M.-A.; Landry, V.; Sergheraert, C.; Croft, S. L.; Krauth-Siegel, R. L.; Davioud-Charvet, E. 2- and 3-Substituted 1,4-Naphthoquinone Derivatives as Subversive Substrates of Trypanothione Reductase and Lipoamide Dehydrogenase from Trypanosoma cruzi: Synthesis and Correlation between Redox Cycling Activities and in Vitro Cytotoxicity. *Journal of Medicinal Chemistry* **2001,** 44, 548-565.
14. Houssin, R.; Bernier, J.-L.; Hénichart, J.-P. A Convenient and General Method for the Preparation of tert-Butoxycarbonylaminoalkanenitriles and Their Conversion to Mono-tert-butoxycarbonylalkanediamines. *Synthesis* **1988,** 1988, 259-261.
15. Hille, U. E.; Zimmer, C.; Haupenthal, J.; Hartmann, R. W. Optimization of the First Selective Steroid-11 beta-hydroxylase (CYP11B1) Inhibitors for the Treatment of Cortisol Dependent Diseases. *Acs Medicinal Chemistry Letters* **2011,** 2, 559-564.
16. Gallasch, D. P.; Woodhouse, S. L.; Rendina, L. M. Synthesis and redistribution reactions of asymmetric sigma-arylplatinum(II) complexes containing 4,7-phenanthroline. *Journal of Organometallic Chemistry* **2004,** 689, 1288-1294.
17. DeGrazia, M. J.; Thompson, J.; Vanden Heuvel, J. P.; Peterson, B. R. Synthesis of a high-affinity fluorescent PPAR gamma ligand for high-throughput fluorescence polarization assays. *Bioorganic & Medicinal Chemistry* **2003,** 11, 4325-4332.
18. Yen, C.-F.; Yuan, J.; King, C.-H. R. Lyophilization formulation comprising pyrimidine compounds and their preparation thereof. US20100120719A1, 2010.

19. Li, L. T.; Mayer, M.; Schneider, E.; Schreiber, E.; Bernhardt, G.; Peng, S. Q.; Buschauer, A. Preparation of fluorescent nonpeptidic neuropeptide Y receptor ligands: Analogues of the quinazoline-type anti-obesity Y-5 antagonist CGP 71683A. *Archiv Der Pharmazie* **2003,** 336, 585-590.
20. Li, J. B.; Sha, Y. W. A convenient synthesis of amino acid methyl esters. *Molecules* **2008,** 13, 1111-1119.
21. Moffat, D. F. C.; Day, F. A.; Patel, S. R.; Belfield, A. J.; Donald, A. D. G.; Davidson, A. H.; Drummond, A. H. Hydroxamates as inhibitors of histone deacetylase. ZA2009002824A, 2010.
22. Bode, C. M.; Cheng, A. C.; Choquette, D.; Lewis, R. T.; Potashman, M. H.; Romero, K.; Stellwagen, J. C.; Whittington, D. A. Preparation of azabenzimidazole derivatives and analogs for use as anaplastic lymphoma kinase inhibitors. WO2012018668A1, 2012.
23. Shin, H. I.; Choi, H. W.; Lee, J. H.; Lee, K. W. A novel process for preparing 3-amino-5-fluoro-4-dialkoxypentanoic acid ester. *WO 2008/020691 A1* **2008**.
24. Ghosh, A. K.; Banerjee, S.; Sinha, S.; Kang, S. B.; Zajc, B. α-Fluorovinyl Weinreb Amides and α-Fluoroenones from a Common Fluorinated Building Block. *J. Org. Chem.* **2009,** 74, 3689-3697.
25. Oster, T. A.; Harris, T. M. Acetylations of strongly basic and nucleophilic enolate anions with N-methoxy-N-methylacetamide. *Tet. Lett.* **1983,** 24, 1851-1854.
26. Marshall, J. A.; Eidam, P.; Eidam, H. S. (R)- and (S)-4-TIPS-3-butyn-2-ol. Useful precursors of chiral allenylzinc and indium reagents. *J. Org. Chem.* **2006,** 71, 4840-4844.
27. Adams, L. A.; Sharma, P.; Mohanty, B.; Ilyichova, O. V.; Mulcair, M. D.; Williams, M. L.; Gleeson, E. C.; Totsika, M.; Doak, B. C.; Caria, S.; Rimmer, K.; Horne, J.; Shouldice, S. R.; Vazirani, M.; Headey, S. J.; Plumb, B. R.; Martin, J. L.; Heras, B.; Simpson, J. S.; Scanlon, M. J. Application of Fragment-Based Screening to the Design of Inhibitors of Escherichia coli DsbA. *Angew Chem Int Ed Engl* **2015,** 54, 2179-84.

14. NMR and HPLC of compounds S1, 46, 91-100,

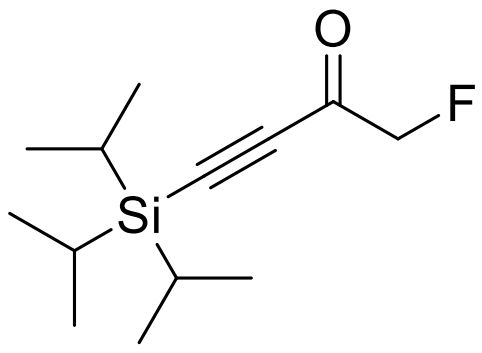

(91)

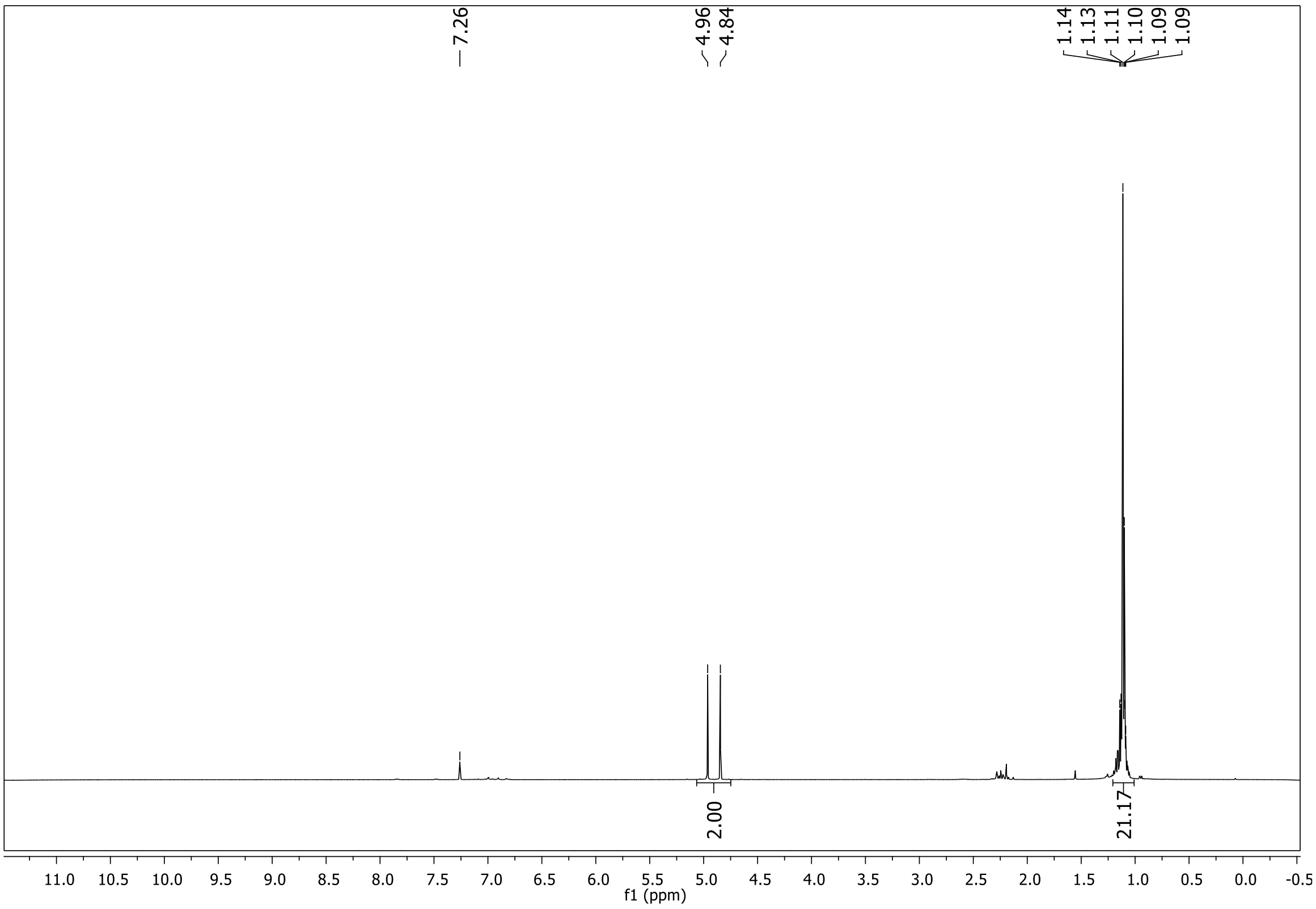

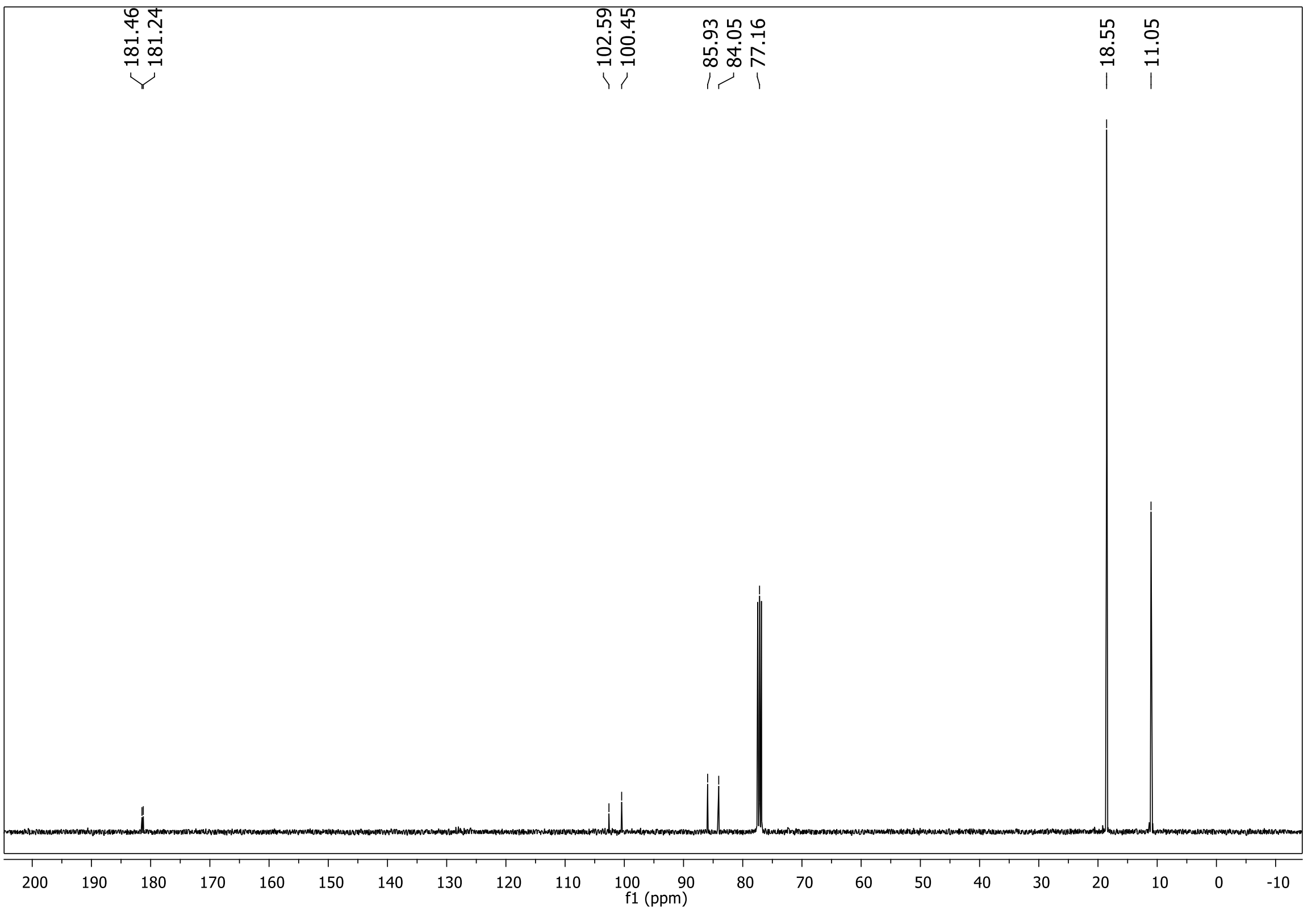

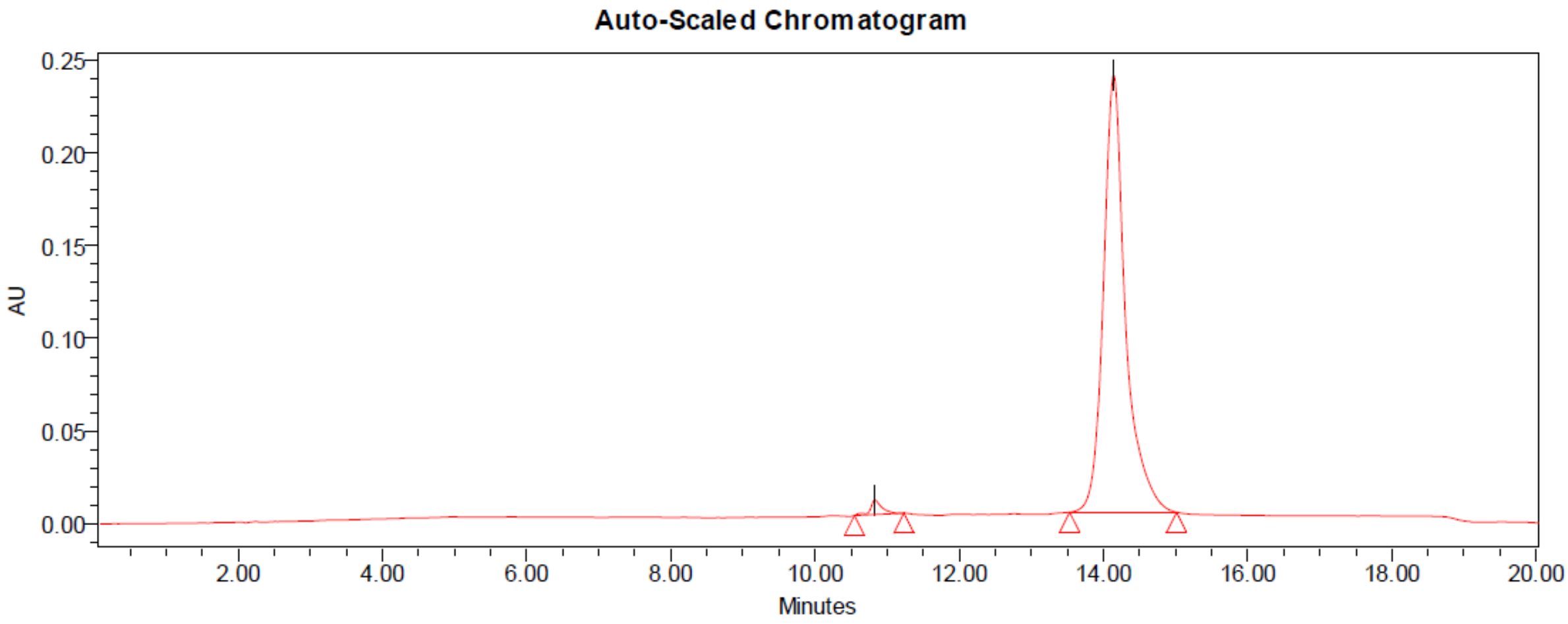

Processed Channel: PDA 254.0 nm

	Processed Channel	Retention Time (min)	Area	% Area	Height
1	PDA 254.0 nm	10.835	88890	1.74	7903
2	PDA 254.0 nm	14.142	5030818	98.26	235908

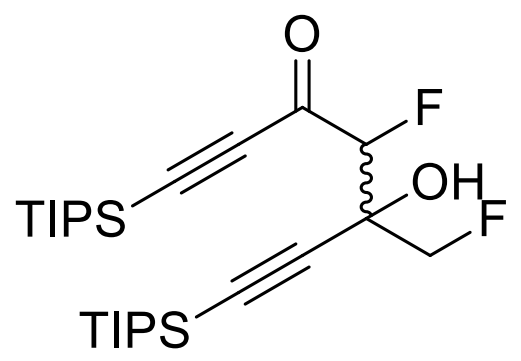
O
F
OH
F
TIPS
TIPS

(**92a**)

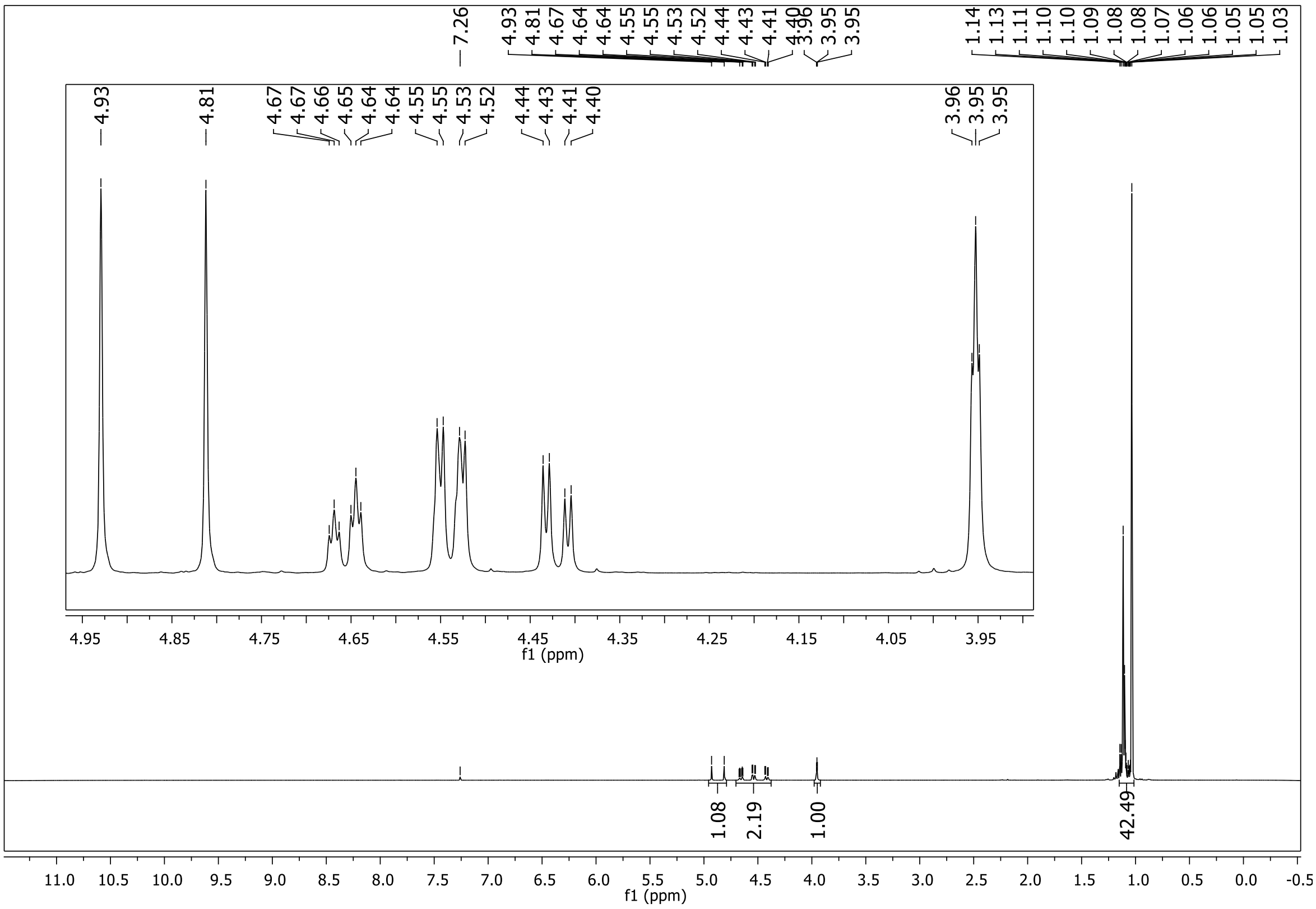
7.26
4.93
4.81
4.67
4.64
4.64
4.55
4.55
4.53
4.52
4.44
4.43
4.41
4.40
3.96
3.95
3.95
1.14
1.13
1.11
1.10
1.09
1.08
1.08
1.07
1.06
1.06
1.05
1.05
1.03
4.93
4.81
4.67
4.67
4.66
4.65
4.64
4.64
4.55
4.55
4.53
4.52
4.44
4.43
4.41
4.40
3.96
3.95
3.95
1.08
2.19
1.00
42.49
f1 (ppm)
f1 (ppm)

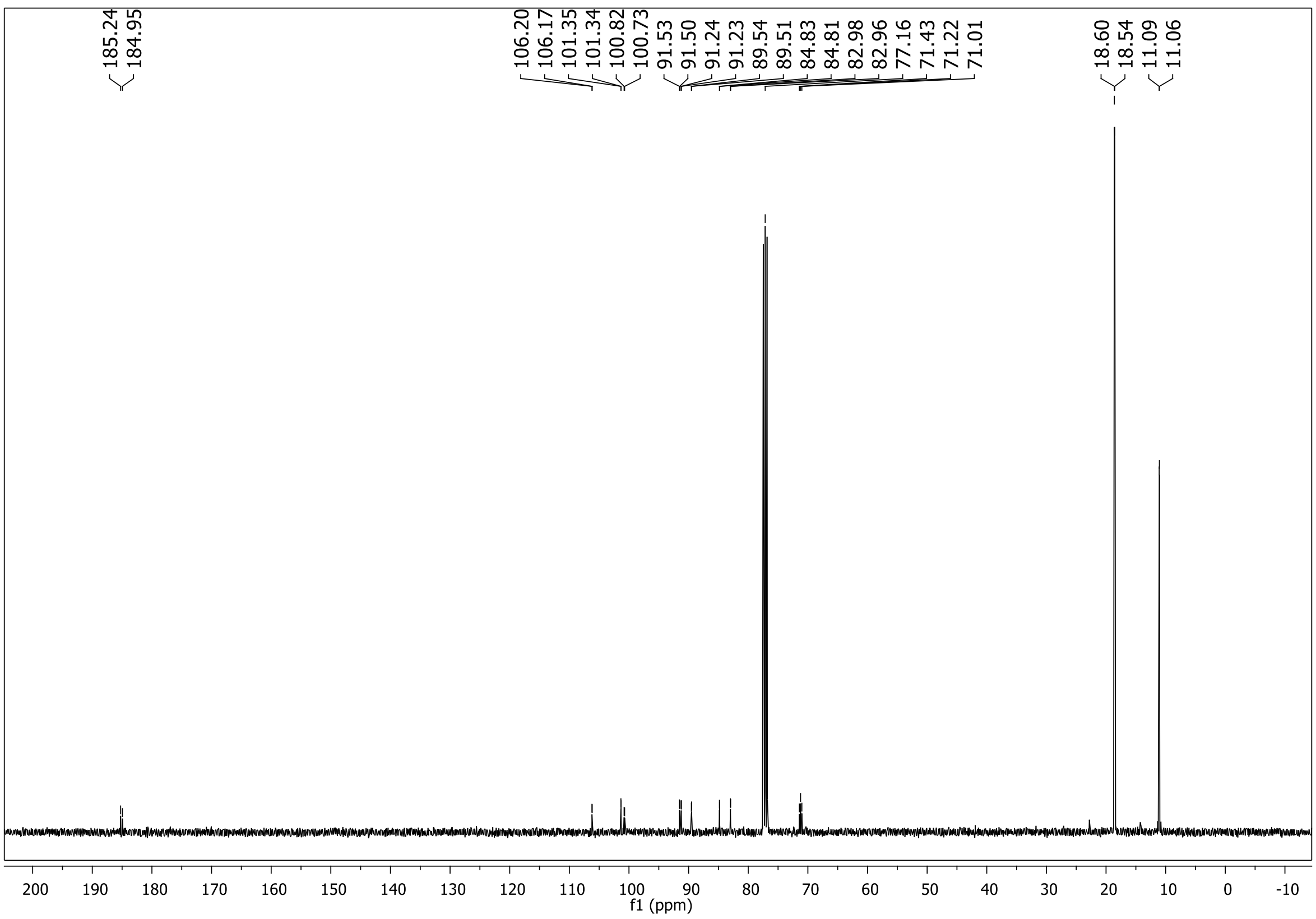
185.24
184.95
106.20
106.17
101.35
101.34
100.82
100.73
91.53
91.50
91.24
91.23
89.54
89.51
84.83
84.81
82.98
82.96
77.16
71.43
71.22
71.01
18.60
18.54
11.09
11.06
f1 (ppm)

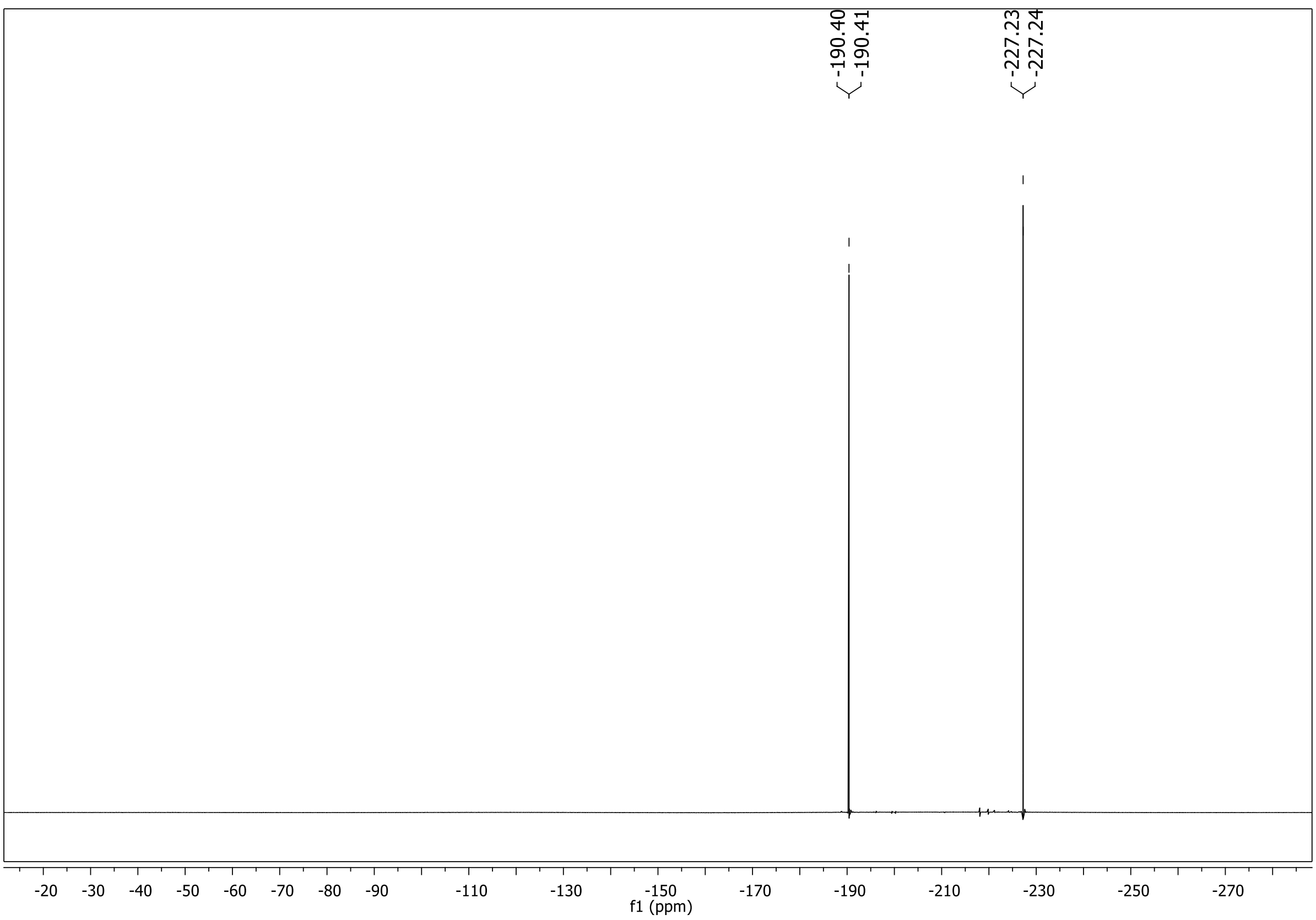
-190.40
-190.41
-227.23
-227.24
-20
-30
-40
-50
-60
-70
-80
-90
-110
-130
-150
-170
-190
-210
-230
-250
-270
f1 (ppm)

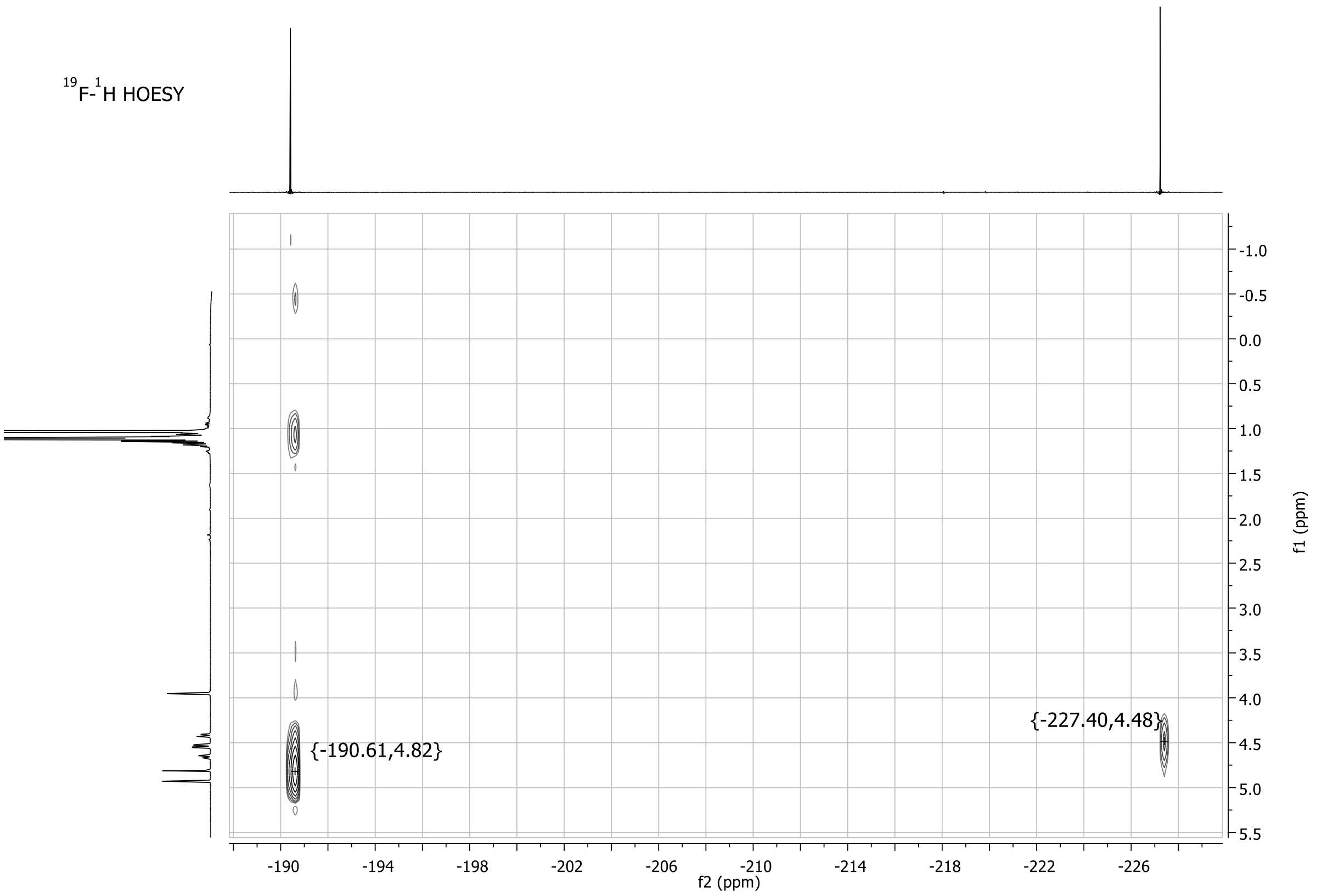
19F-1H HOESY
{-190.61,4.82}
{-227.40,4.48}
-1.0
-0.5
0.0
0.5
1.0
1.5
2.0
2.5
3.0
3.5
4.0
4.5
5.0
5.5
f1 (ppm)
-190
-194
-198
-202
-206
-210
-214
-218
-222
-226
f2 (ppm)

HSQC

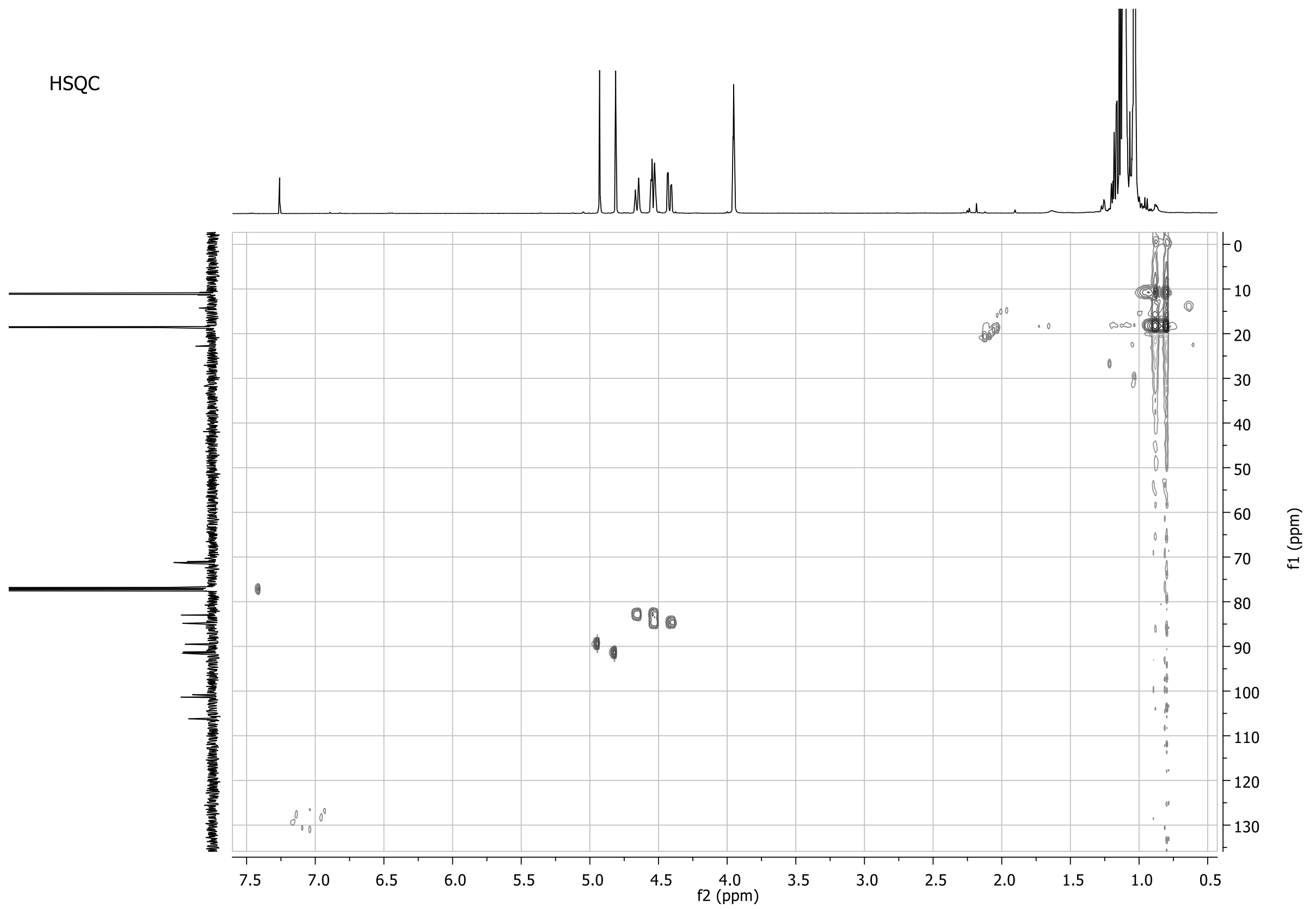

HMBC

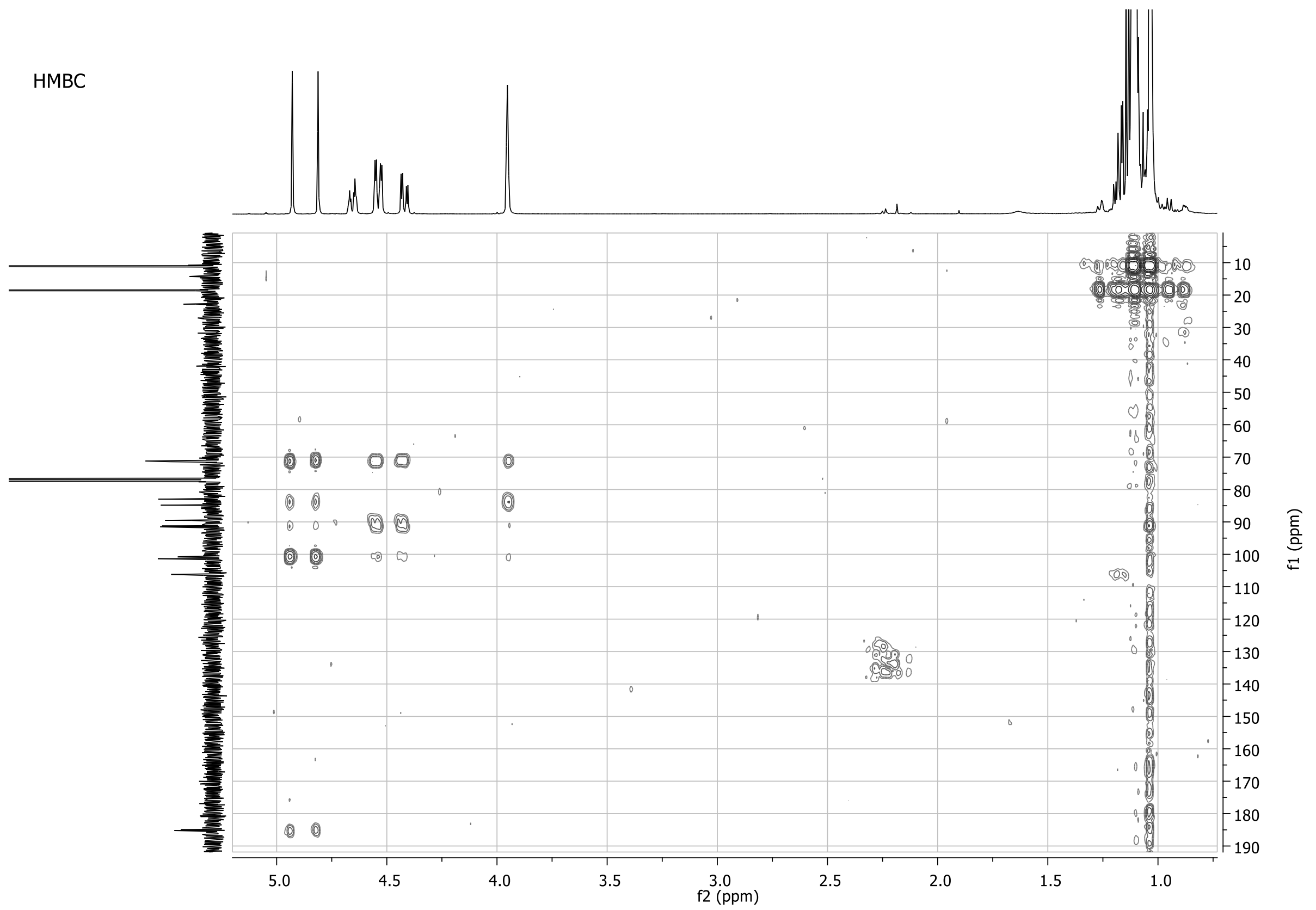

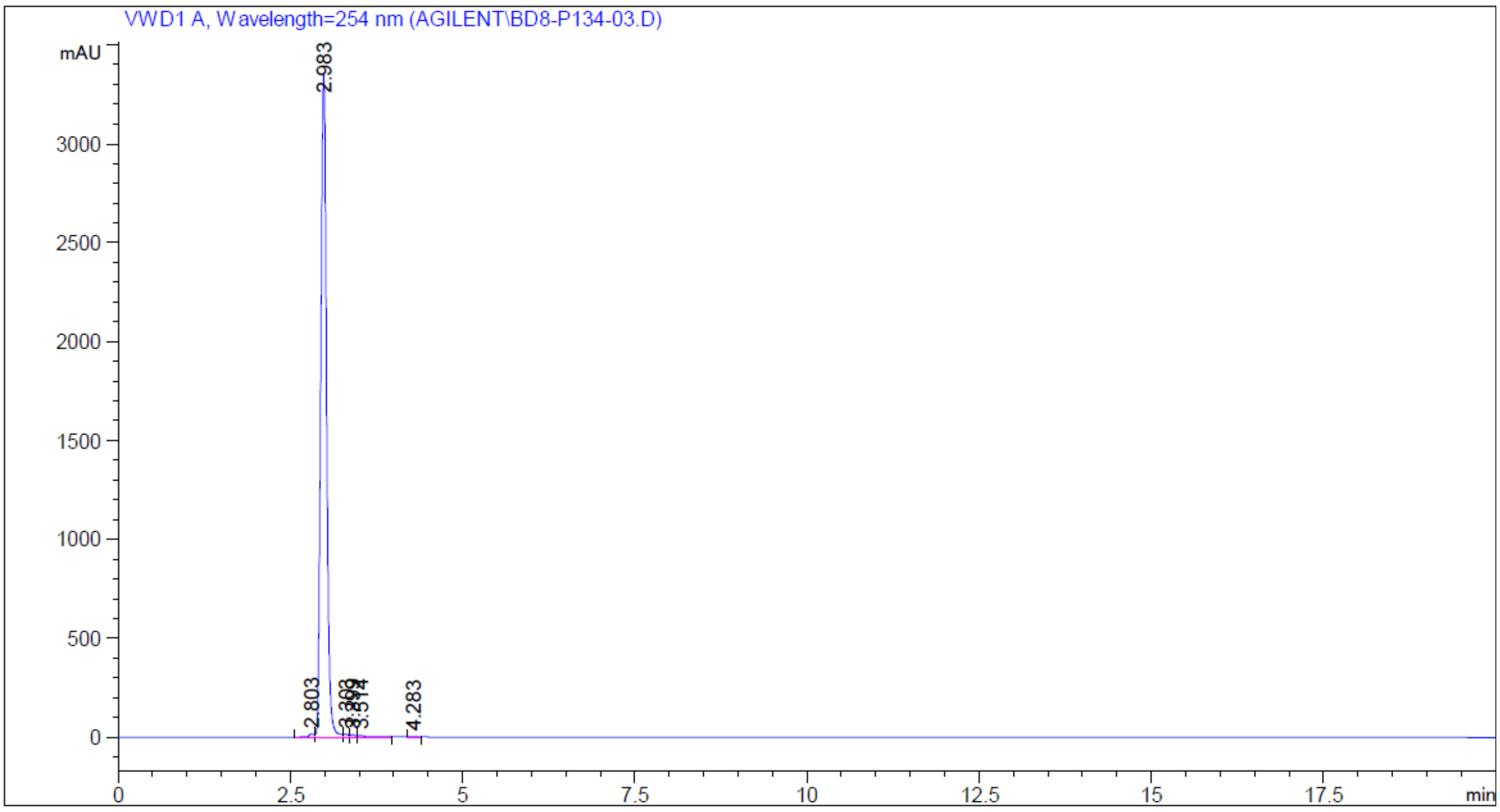

Peak #	RetTime [min]	Type	Width [min]	Area [mAU*s]	Area %
1	2.803	BV	0.0663	73.35941	0.3614
2	2.983	VV	0.0927	2.00207e4	98.6176
3	3.303	VV	0.0632	65.61765	0.3232

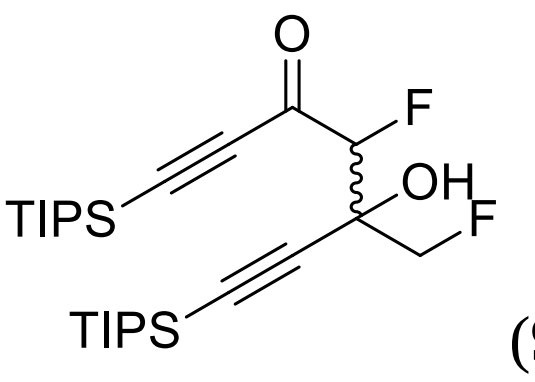
O
F
OH
F
TIPS
TIPS

(**92b**)

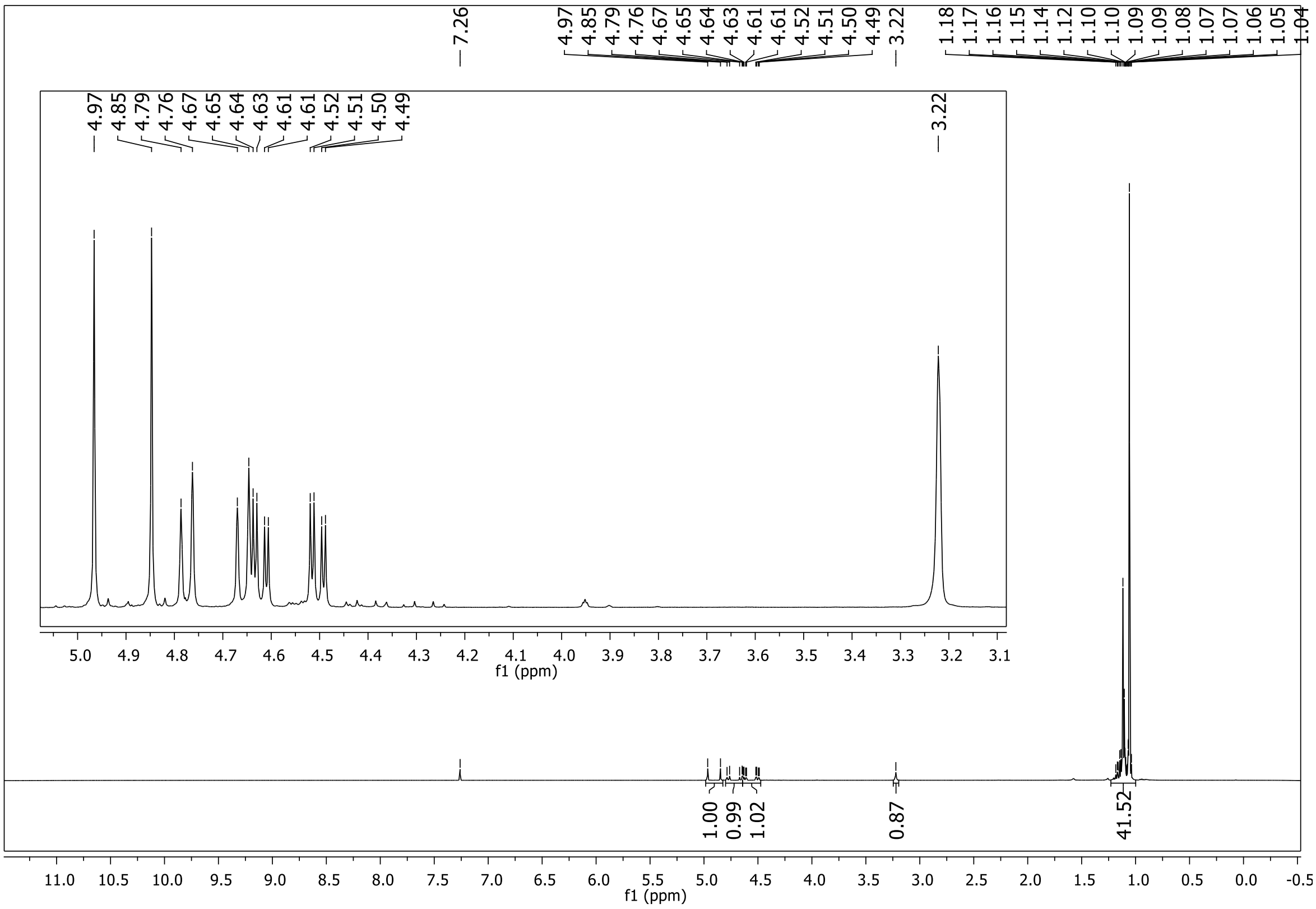
7.26
4.97
4.85
4.79
4.76
4.67
4.65
4.64
4.63
4.61
4.61
4.52
4.51
4.50
4.49
3.22
1.18
1.17
1.16
1.15
1.14
1.12
1.10
1.10
1.09
1.09
1.08
1.07
1.07
1.06
1.05
1.04
1.00
0.99
1.02
0.87
41.52
f1 (ppm)

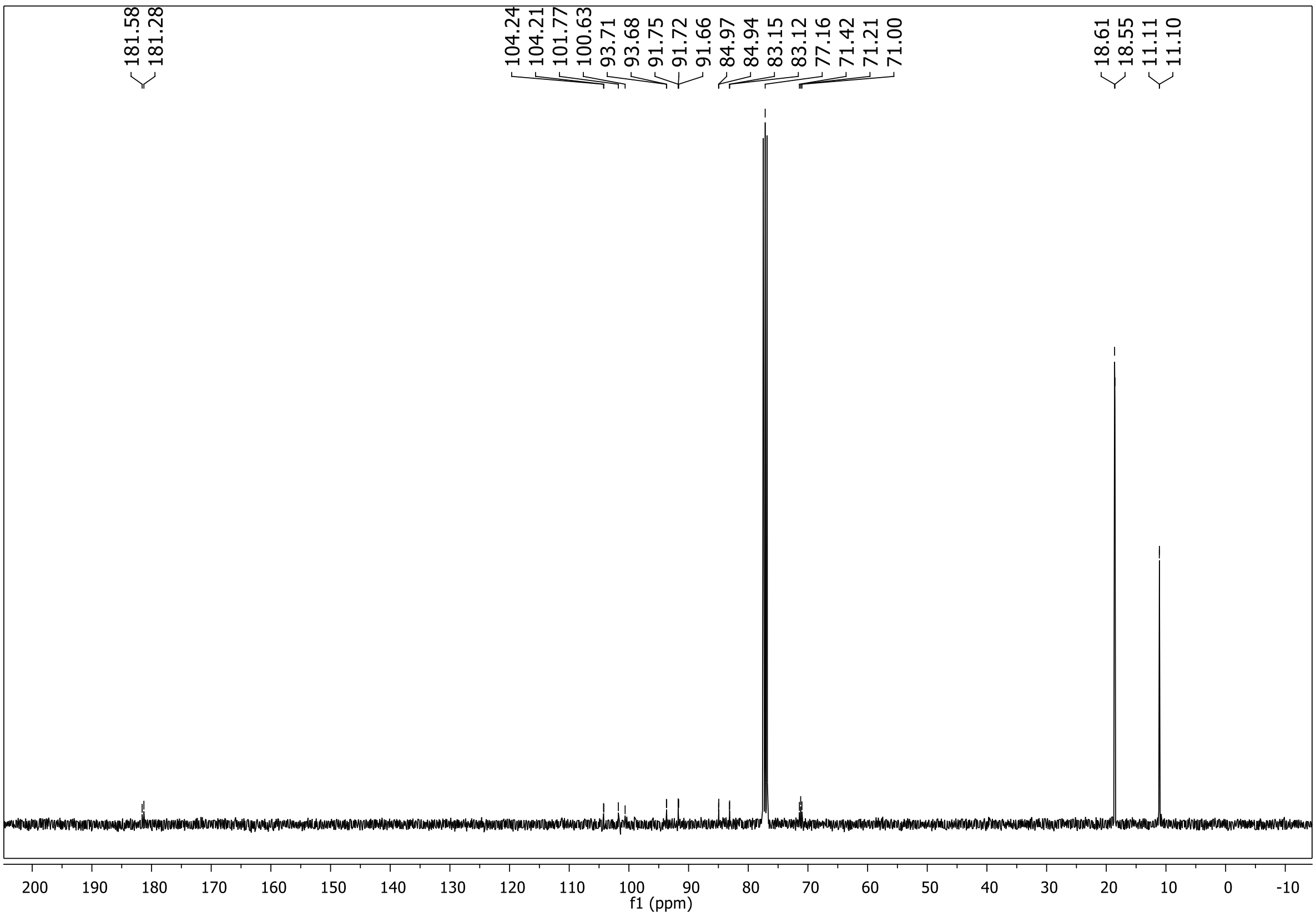
181.58
181.28
104.24
104.21
101.77
100.63
93.71
93.68
91.75
91.72
91.66
84.97
84.94
83.15
83.12
77.16
71.42
71.21
71.00
18.61
18.55
11.11
11.10
f1 (ppm)

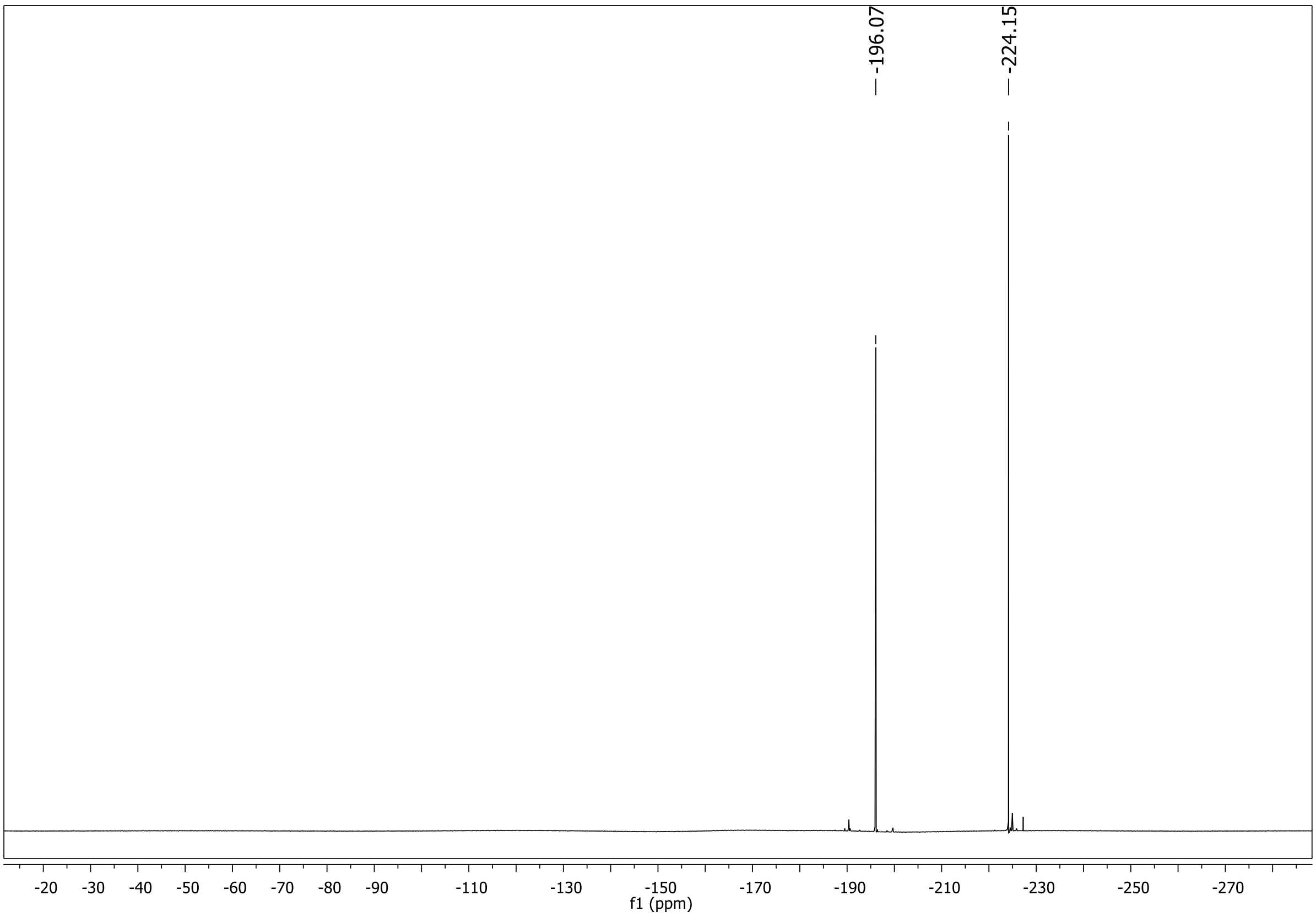

-196.07
-224.15
-20 -30 -40 -50 -60 -70 -80 -90 -110 -130 -150 -170 -190 -210 -230 -250 -270
f1 (ppm)

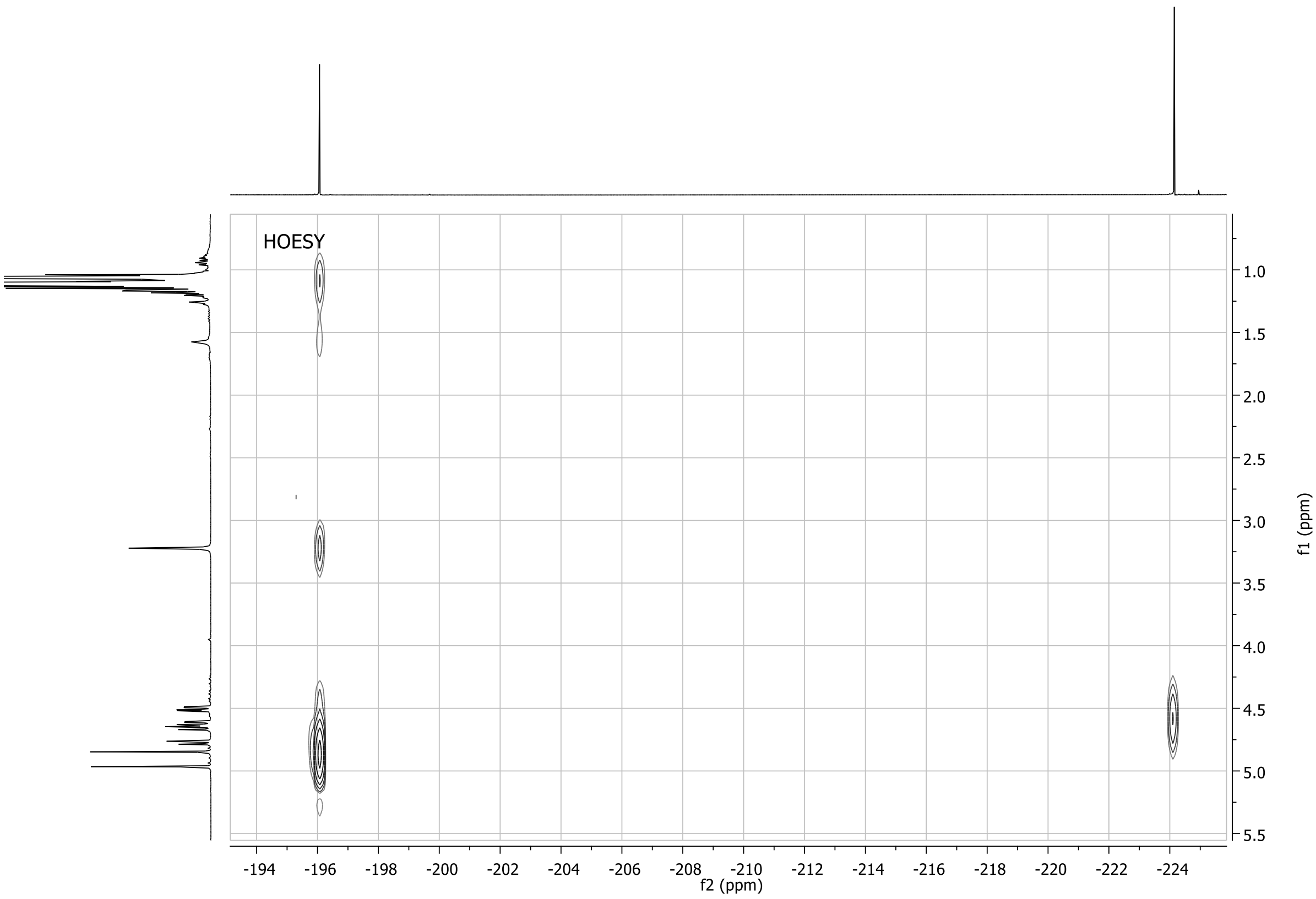

HOESY
1.0 1.5 2.0 2.5 3.0 3.5 4.0 4.5 5.0 5.5
f1 (ppm)
-194 -196 -198 -200 -202 -204 -206 -208 -210 -212 -214 -216 -218 -220 -222 -224
f2 (ppm)

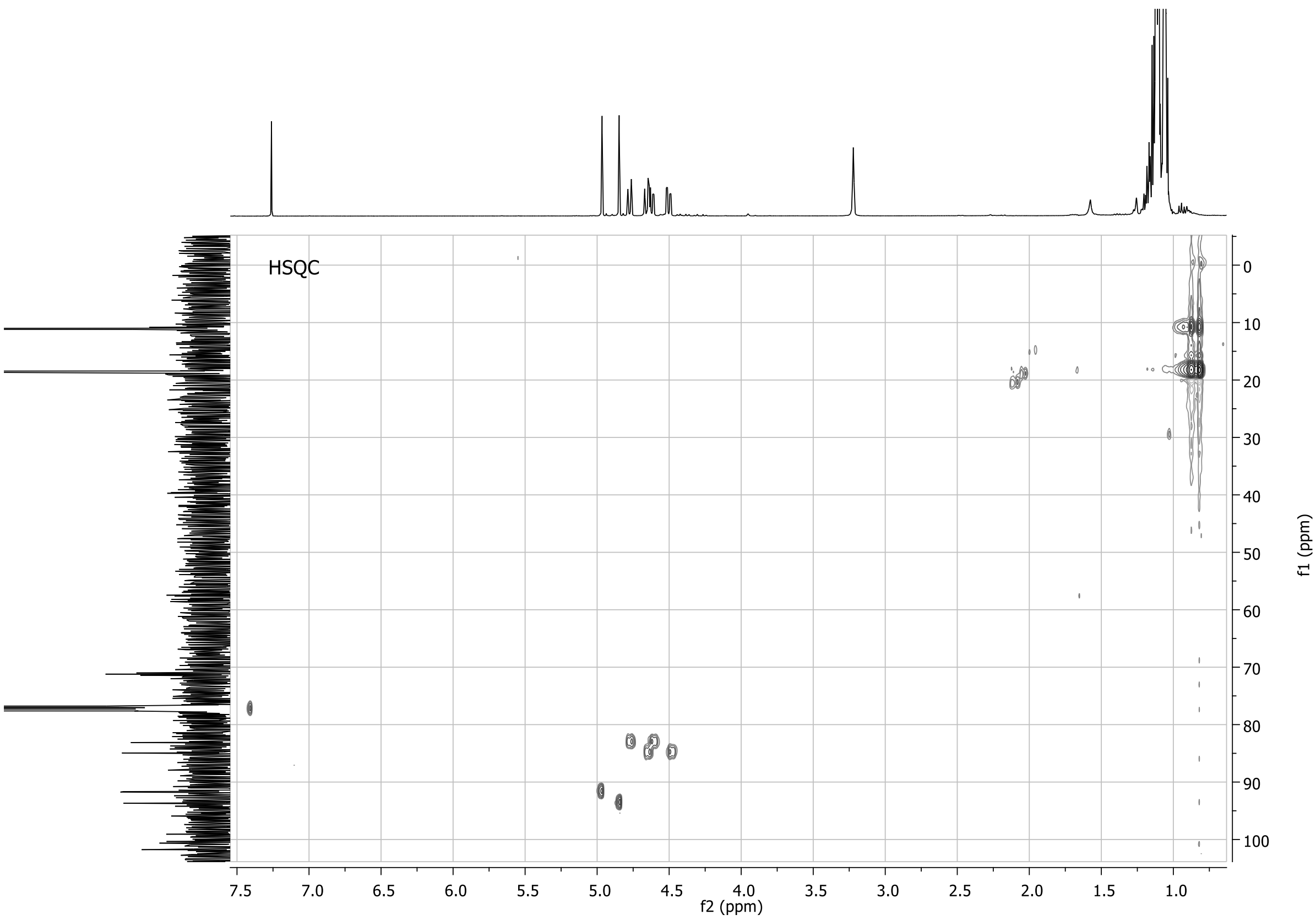
HSQC
f2 (ppm)
f1 (ppm)

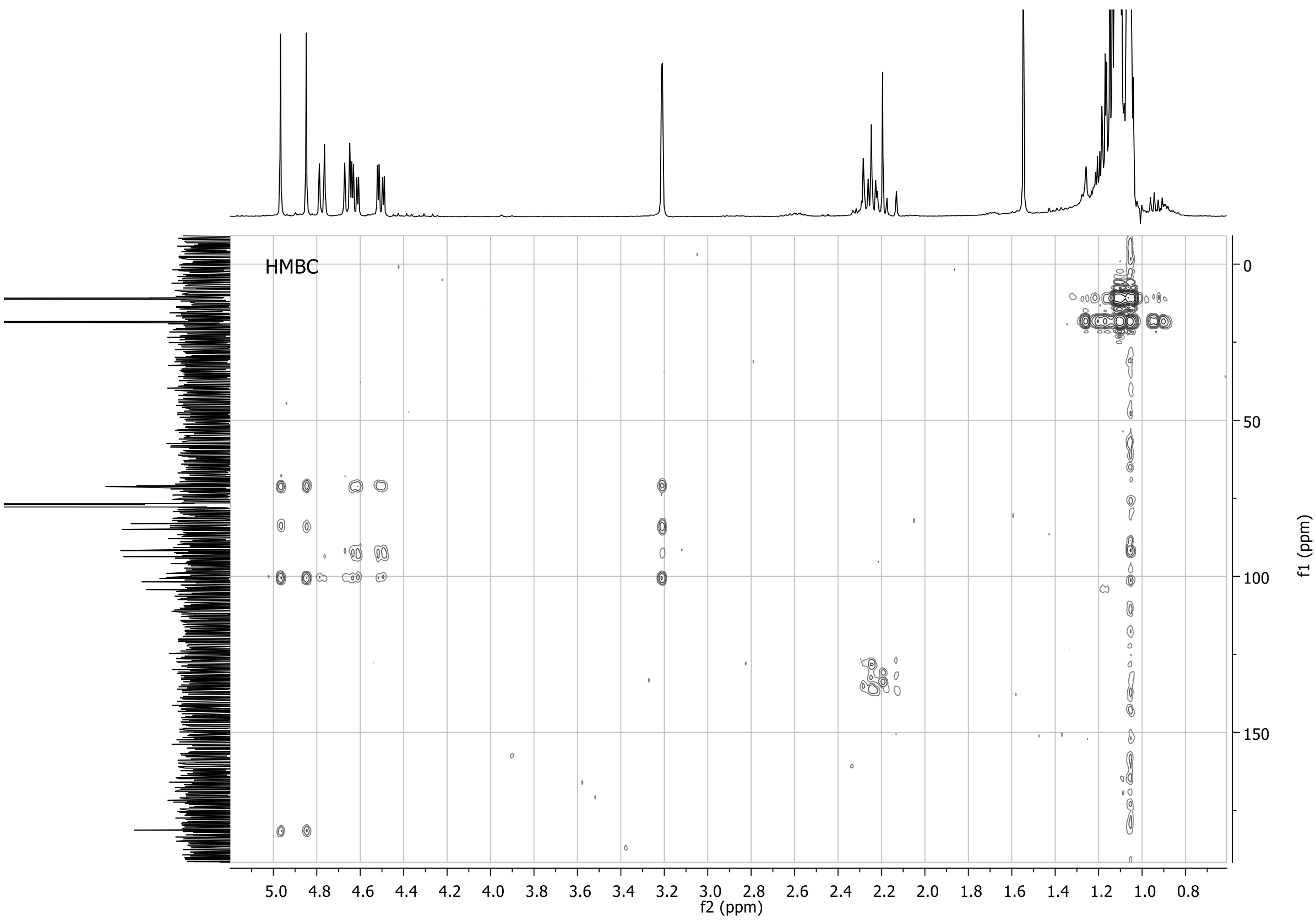
HMBC
f2 (ppm)
f1 (ppm)

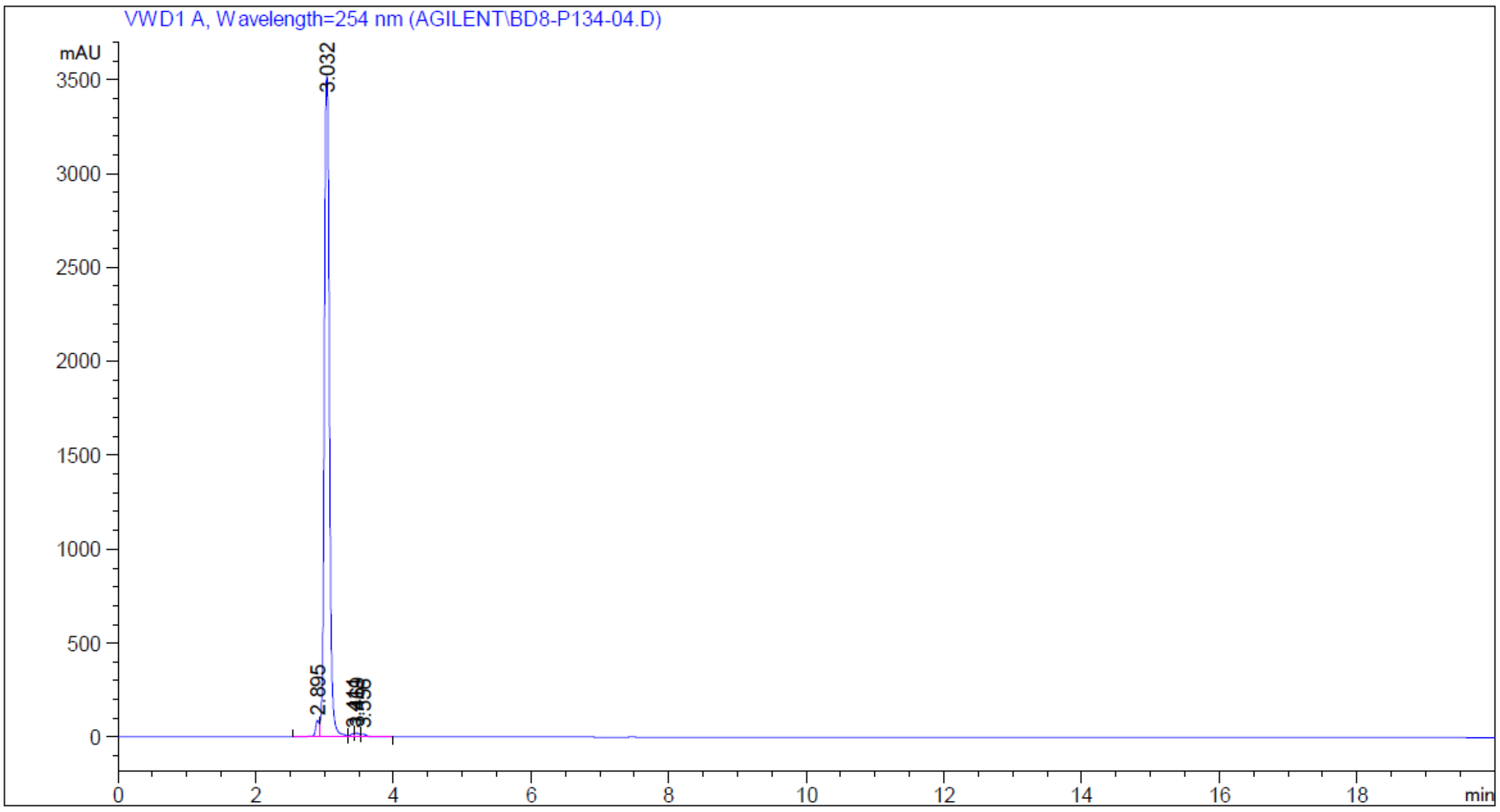

Peak #	RetTime [min]	Type	Width [min]	Area [mAU*s]	Area %
1	2.895	BV	0.0555	333.60568	1.7729
2	3.032	VV	0.0813	1.82167e4	96.8075
3	3.411	VV	0.0570	65.13184	0.3461

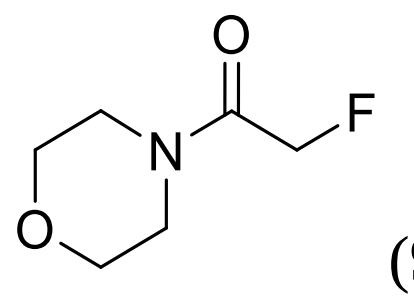
O
N
F
O

(93)

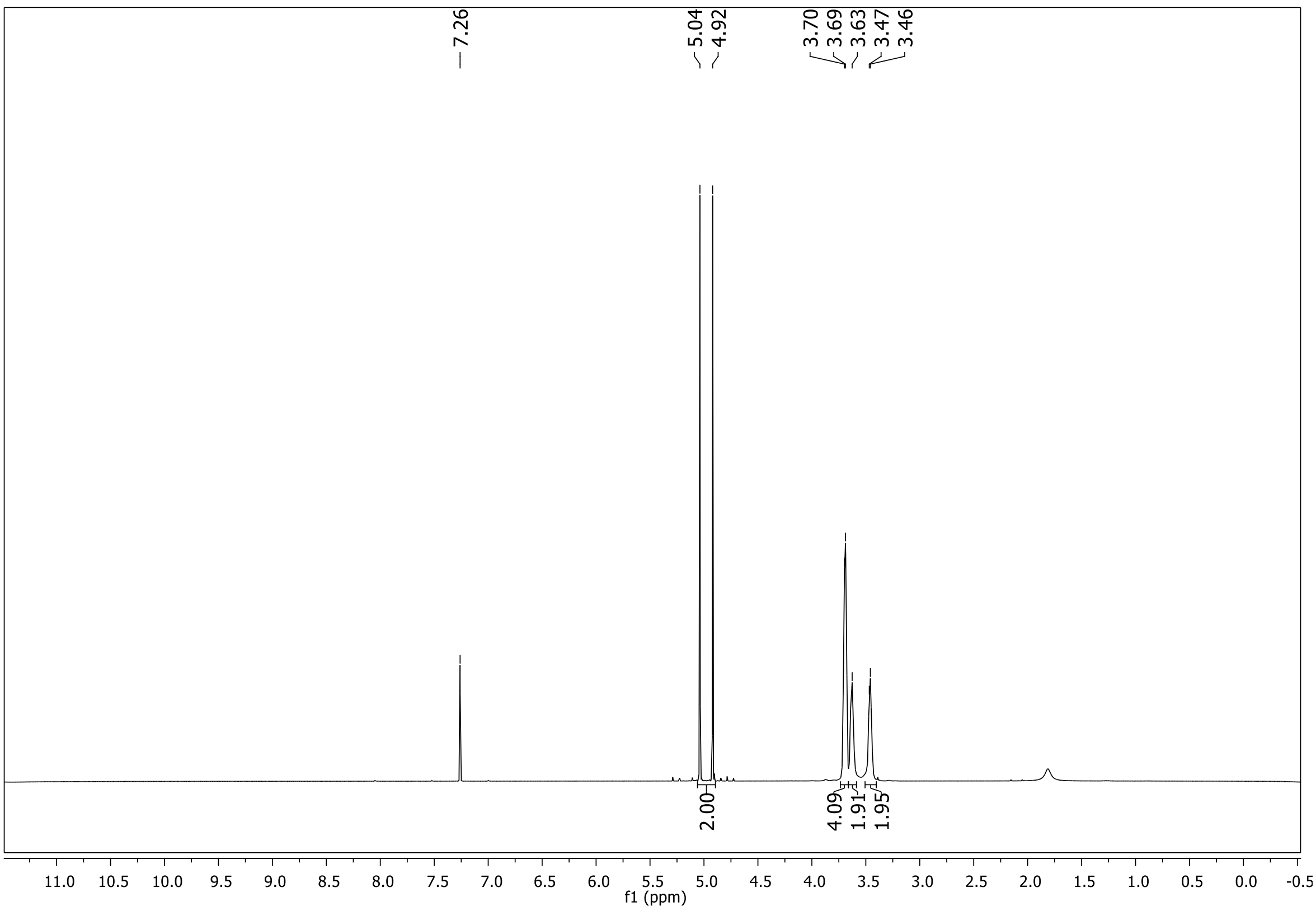
7.26
5.04
4.92
3.70
3.69
3.63
3.47
3.46
2.00
4.09
1.91
1.95
11.0 10.5 10.0 9.5 9.0 8.5 8.0 7.5 7.0 6.5 6.0 5.5 5.0 4.5 4.0 3.5 3.0 2.5 2.0 1.5 1.0 0.5 0.0 -0.5
f1 (ppm)

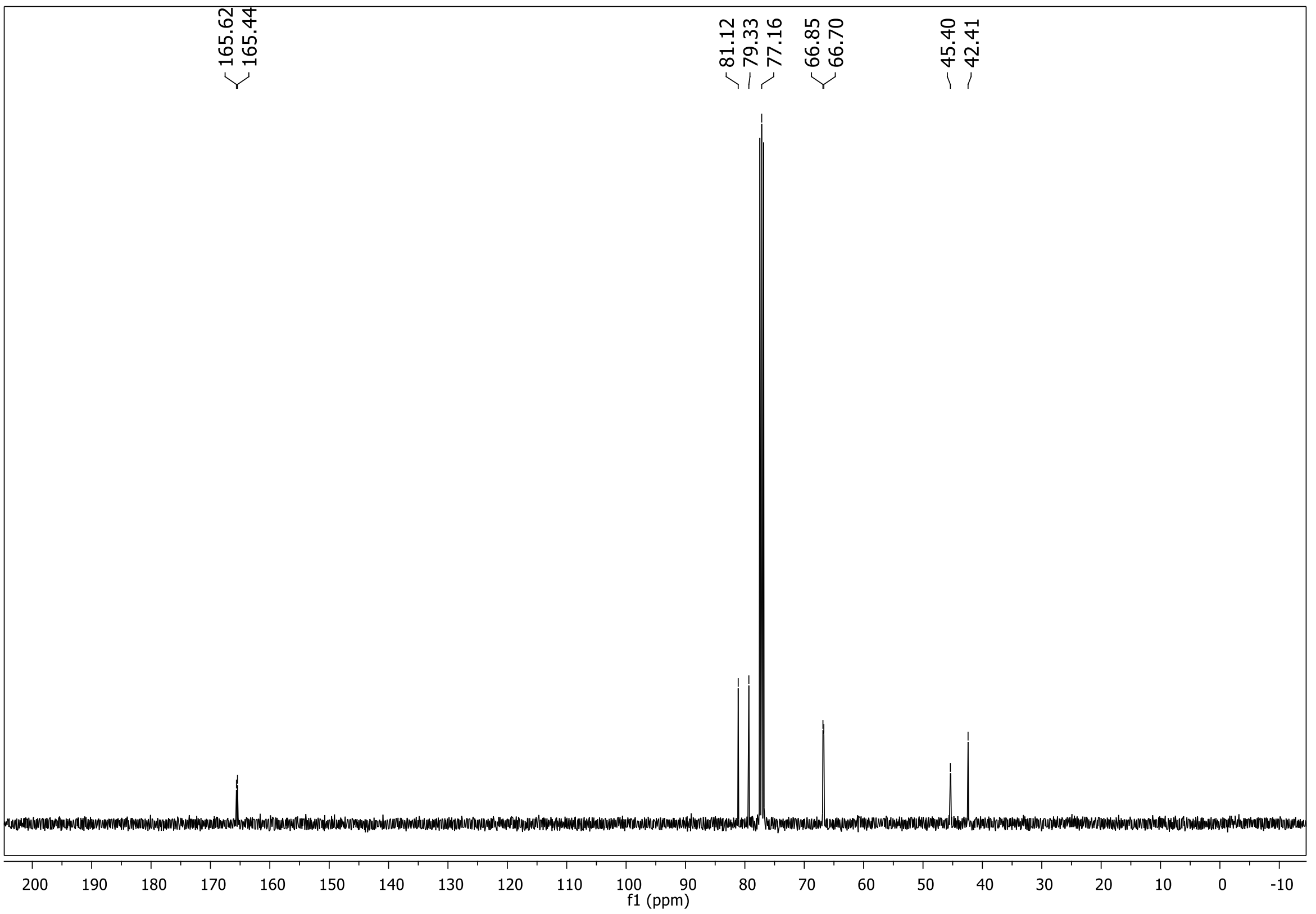
165.62
165.44
81.12
79.33
77.16
66.85
66.70
45.40
42.41
200 190 180 170 160 150 140 130 120 110 100 90 80 70 60 50 40 30 20 10 0 -10
f1 (ppm)

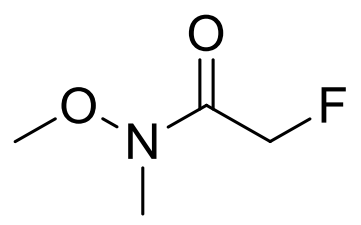

(95)

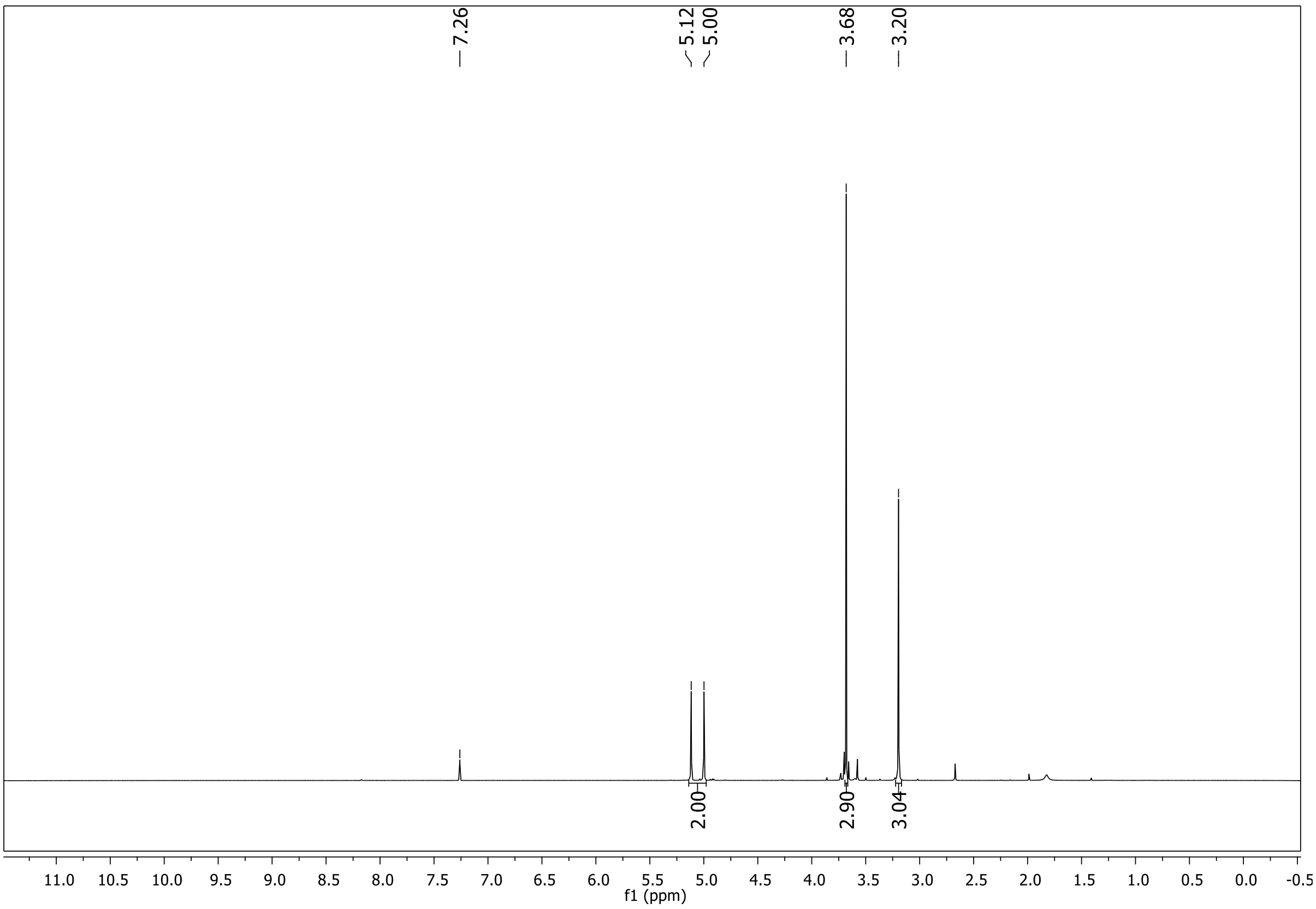
7.26
5.12
5.00
3.68
3.20
2.00
2.90
3.04
11.0 10.5 10.0 9.5 9.0 8.5 8.0 7.5 7.0 6.5 6.0 5.5 5.0 4.5 4.0 3.5 3.0 2.5 2.0 1.5 1.0 0.5 0.0 -0.5
f1 (ppm)

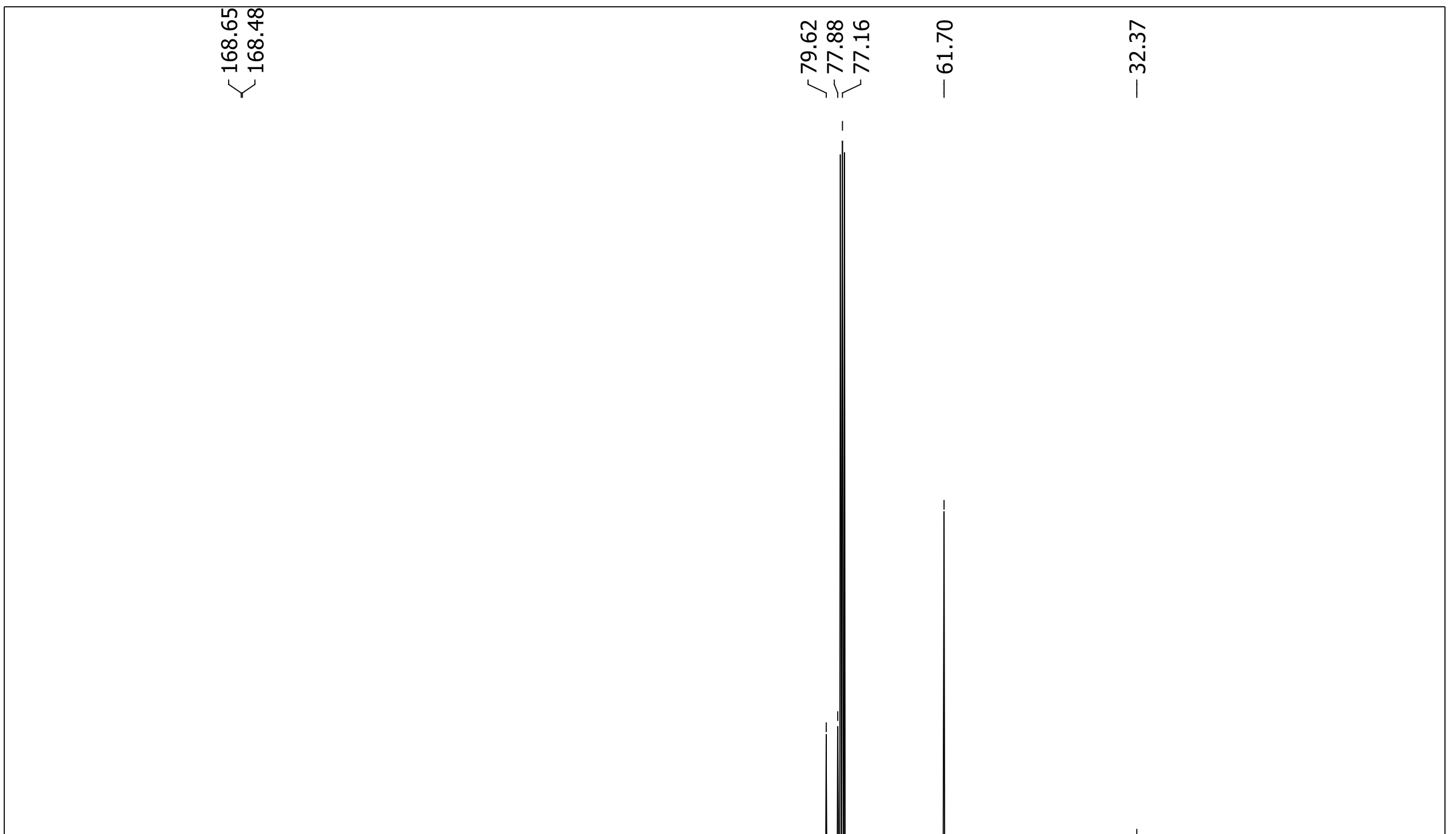
168.65
168.48
79.62
77.88
77.16
61.70
32.37
200 190 180 170 160 150 140 130 120 110 100 90 80 70 60 50 40 30 20 10 0 -10
f1 (ppm)

Auto-Scaled Chromatogram

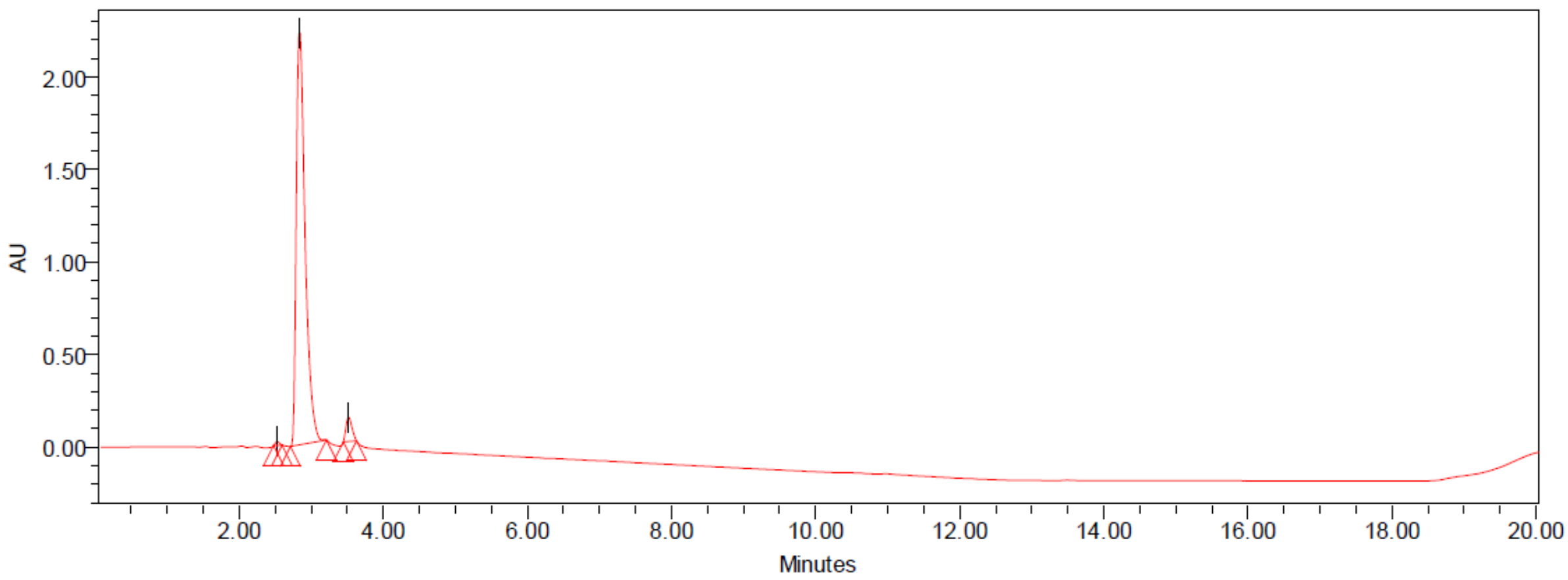

Processed Channel: PDA 214.0 nm

	Processed Channel	Retention Time (min)	Area	% Area	Height
1	PDA 214.0 nm	2.530	77524	0.40	19791
2	PDA 214.0 nm	2.841	18747752	95.75	2234324
3	PDA 214.0 nm	3.520	753641	3.85	131661

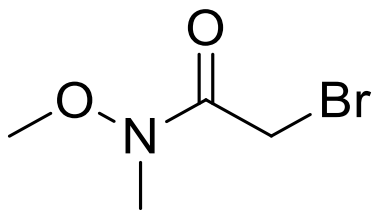

(**94**)

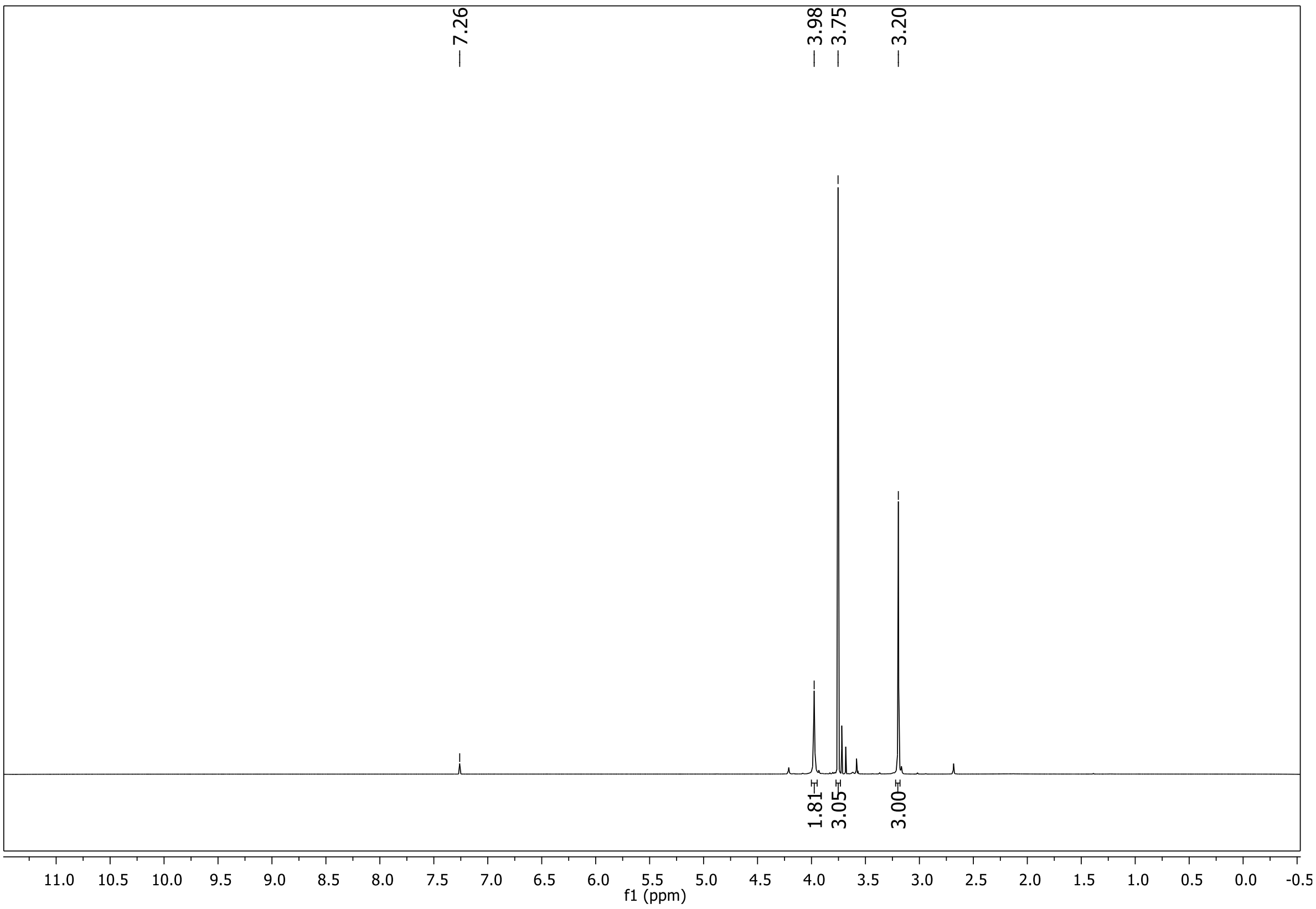

7.26
3.98
3.75
3.20
1.81
3.05
3.00
11.0
10.5
10.0
9.5
9.0
8.5
8.0
7.5
7.0
6.5
6.0
5.5
5.0
4.5
4.0
3.5
3.0
2.5
2.0
1.5
1.0
0.5
0.0
-0.5
f1 (ppm)

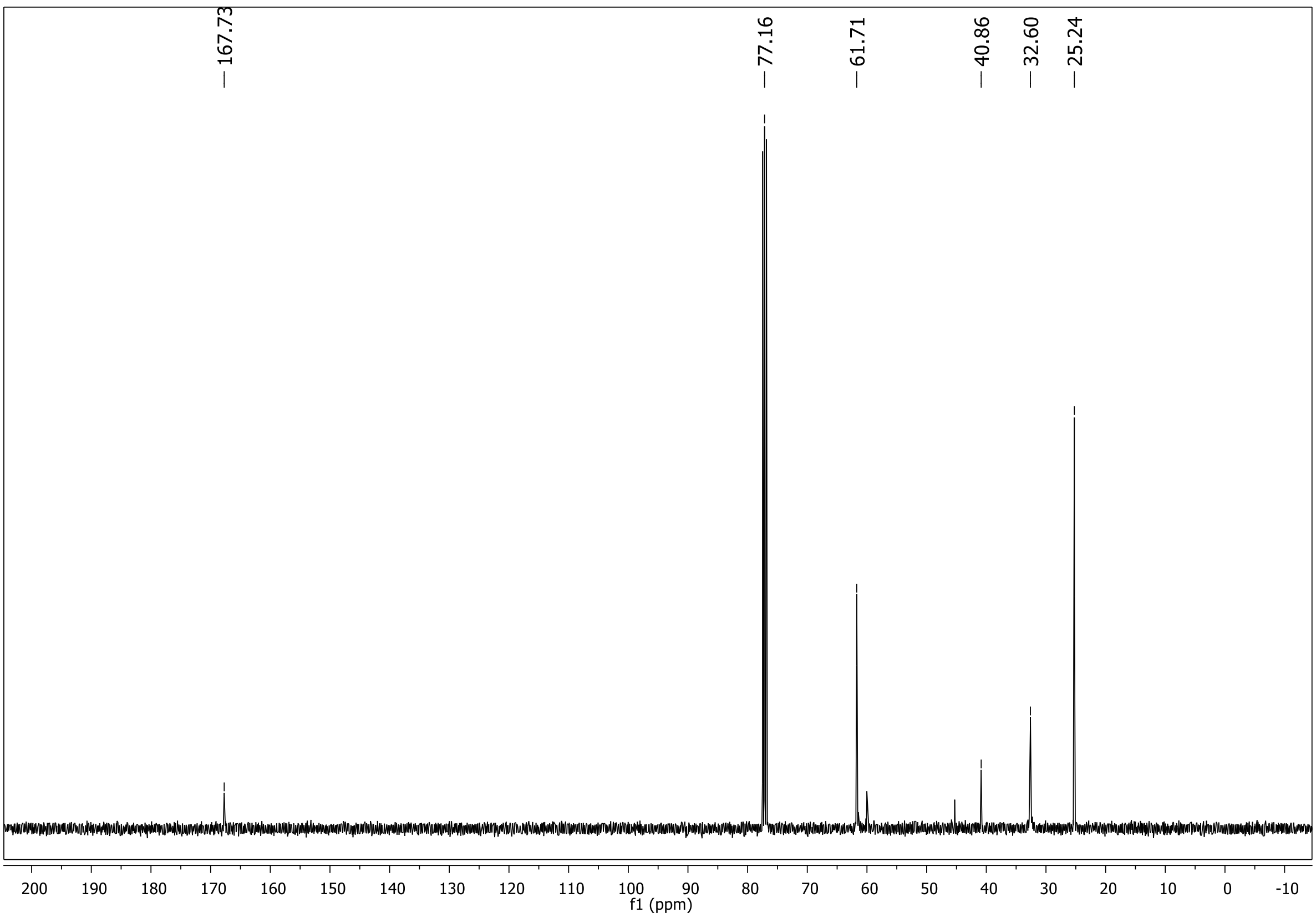

167.73
77.16
61.71
40.86
32.60
25.24
200
190
180
170
160
150
140
130
120
110
100
90
80
70
60
50
40
30
20
10
0
-10
f1 (ppm)

Auto-Scaled Chromatogram

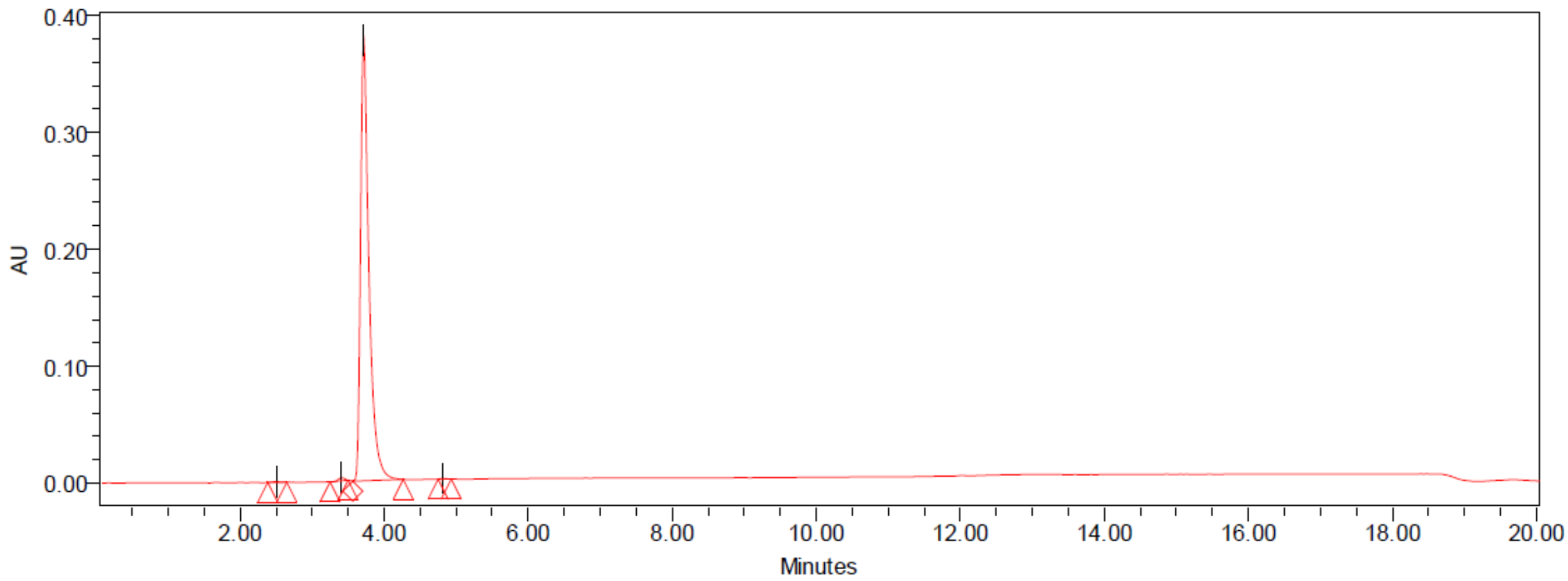

Processed Channel: PDA 254.0 nm

	Processed Channel	Retention Time (min)	Area	% Area	Height
1	PDA 254.0 nm	2.504	2375	0.08	327
2	PDA 254.0 nm	3.410	15608	0.50	2758
3	PDA 254.0 nm	3.720	3086572	99.34	380120
4	PDA 254.0 nm	4.815	2384	0.08	433

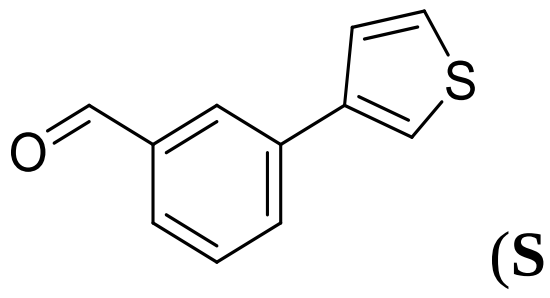
O
S

(**S1**)

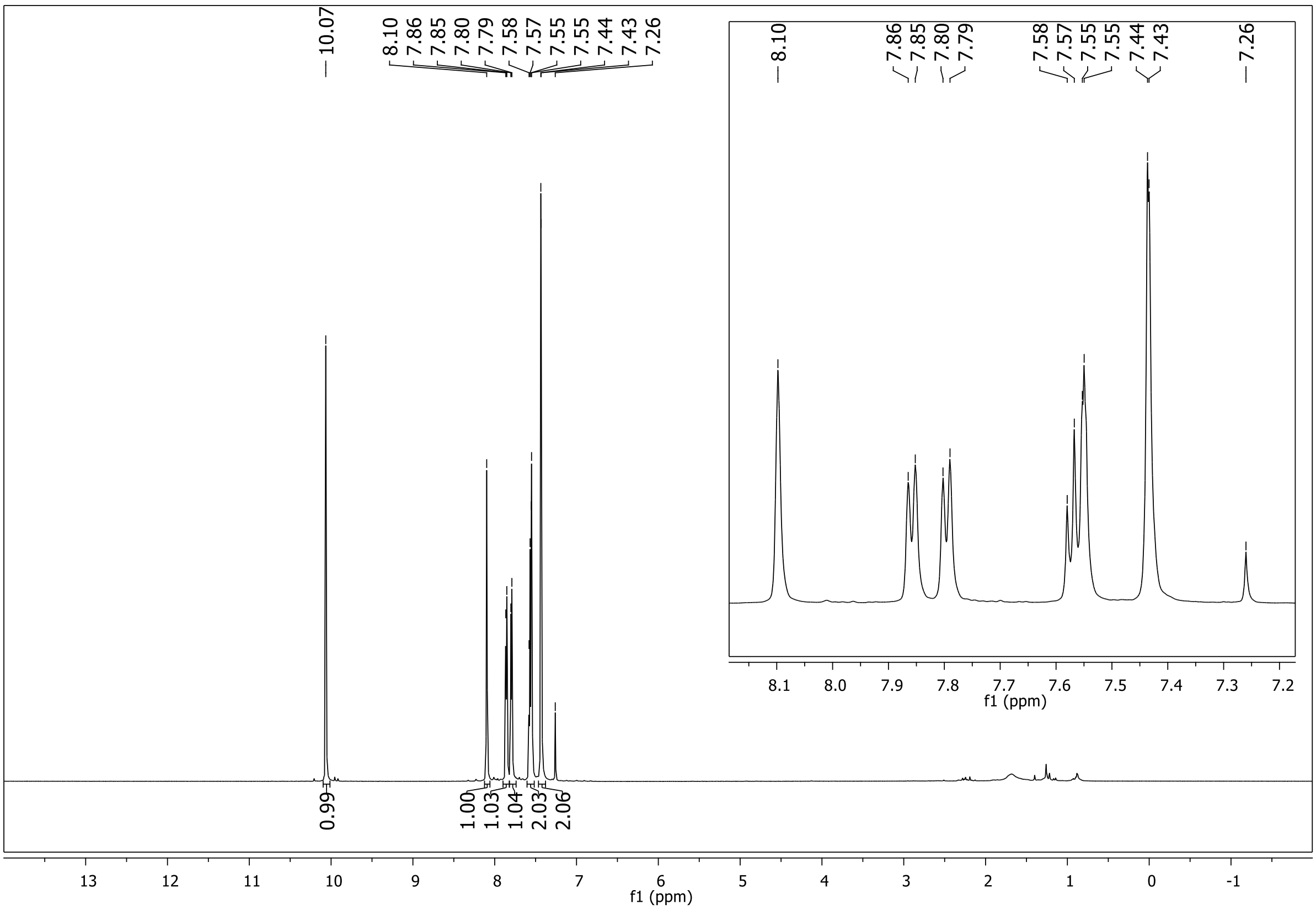
10.07
8.10 7.86 7.85 7.80 7.79 7.58 7.57 7.55 7.55 7.44 7.43 7.26
0.99
1.00 1.03 1.04 2.03 2.06
f1 (ppm)

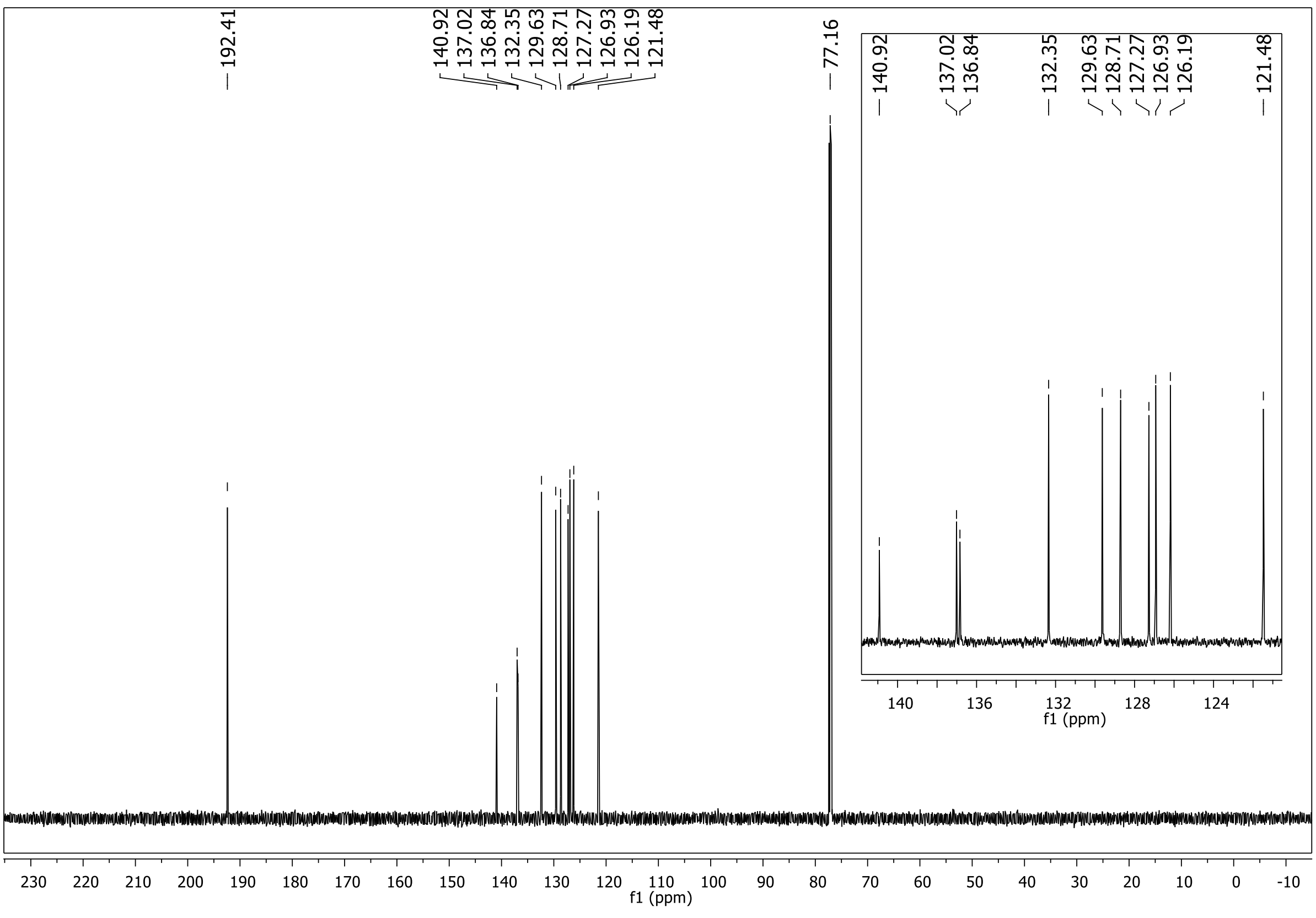
192.41
140.92 137.02 136.84 132.35 129.63 128.71 127.27 126.93 126.19 121.48
77.16
f1 (ppm)

Auto-Scaled Chromatogram

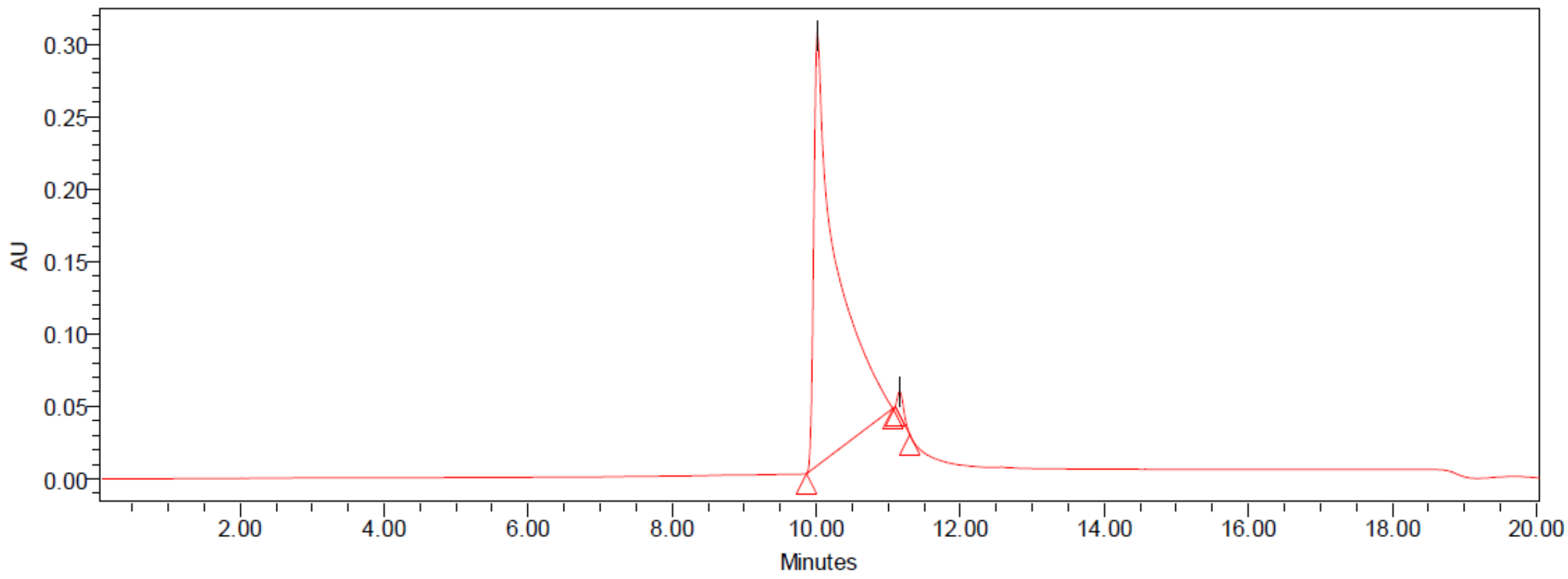

Processed Channel: PDA 254.0 nm

	Processed Channel	Retention Time (min)	Area	% Area	Height
1	PDA 254.0 nm	10.022	6633667	98.62	300006
2	PDA 254.0 nm	11.159	93029	1.38	16023

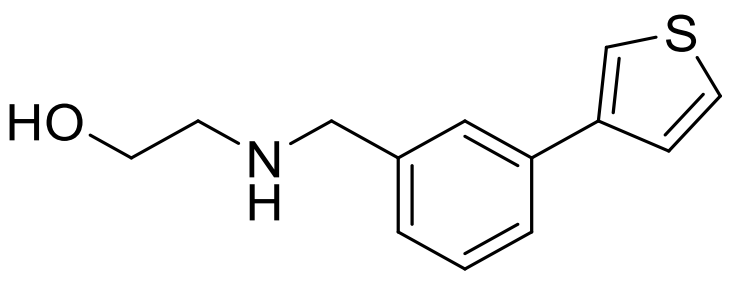

(46)

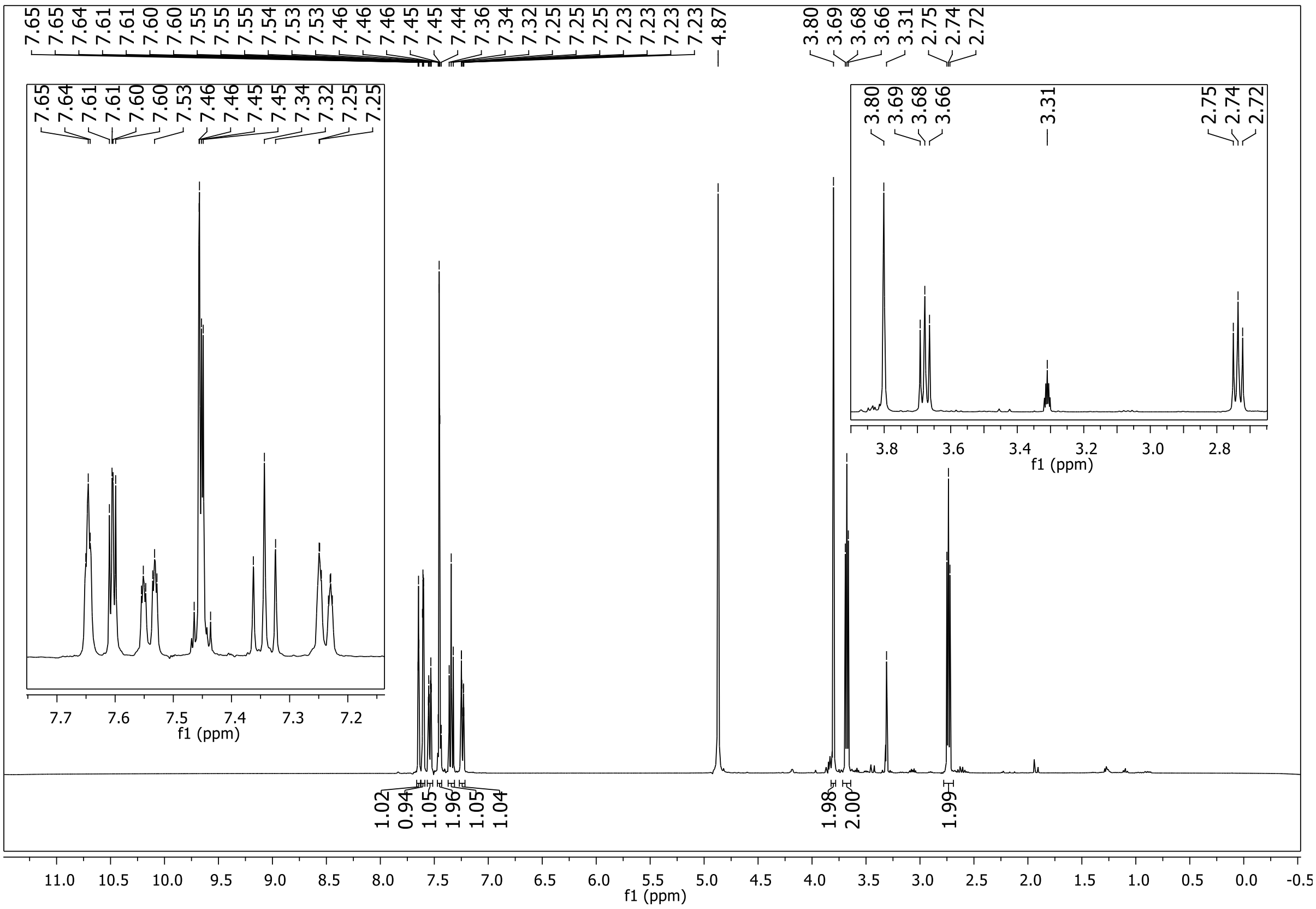

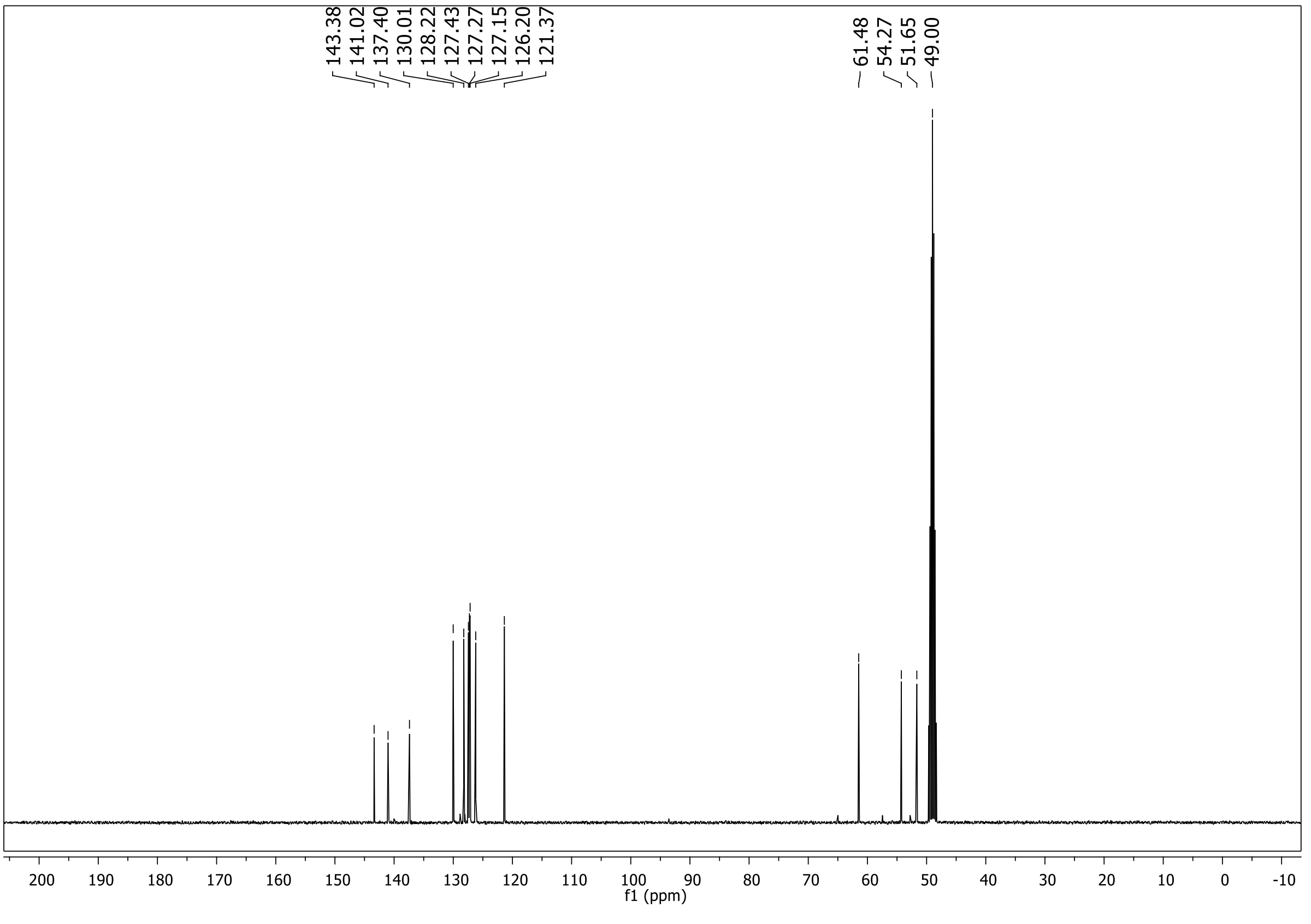

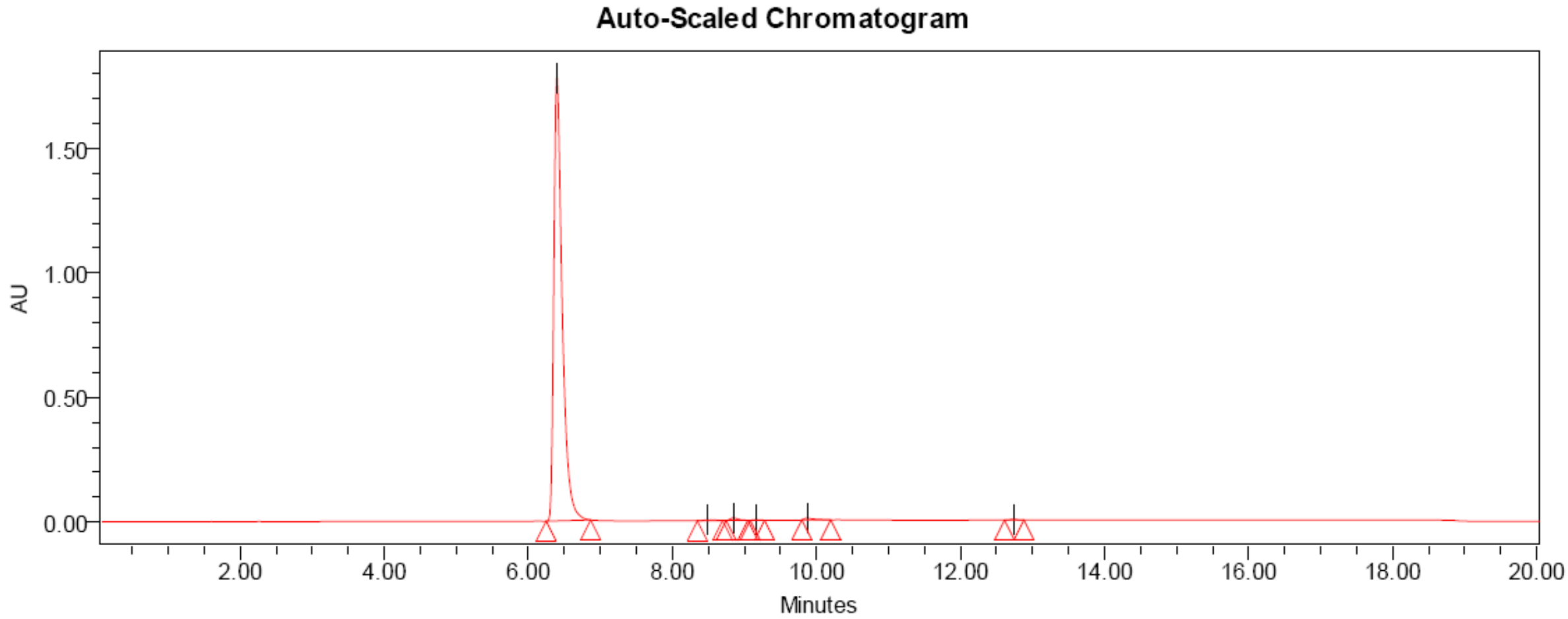

Processed Channel: PDA 254.0 nm

	Processed Channel	Retention Time (min)	Area	% Area	Height
1	PDA 254.0 nm	6.399	14513595	98.58	1793292
2	PDA 254.0 nm	8.482	32749	0.22	3230
3	PDA 254.0 nm	8.850	76049	0.52	10205
4	PDA 254.0 nm	9.168	16307	0.11	2664
5	PDA 254.0 nm	9.873	61849	0.42	6693
6	PDA 254.0 nm	12.734	22650	0.15	3515

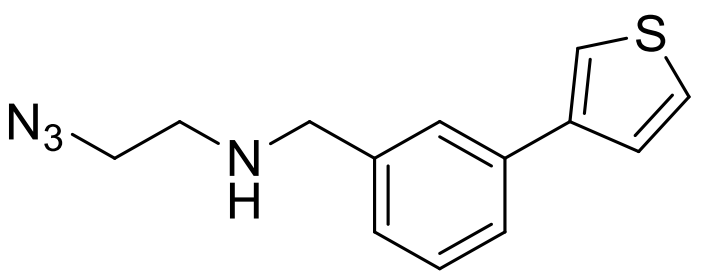

(96)

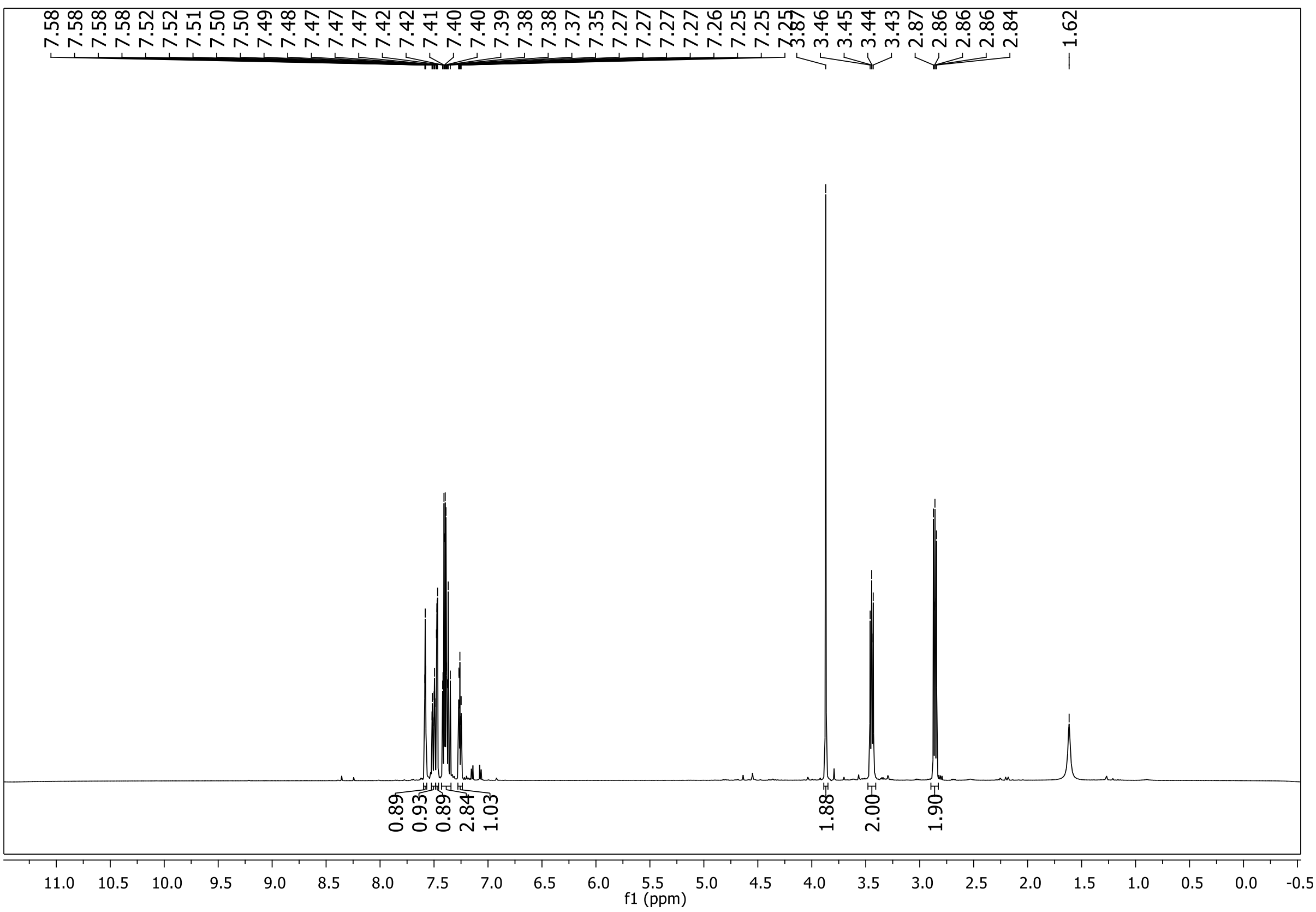
7.58 7.58 7.58 7.58 7.52 7.52 7.51 7.50 7.50 7.49 7.48 7.47 7.47 7.47 7.42 7.42 7.41 7.40 7.40 7.39 7.38 7.38 7.37 7.35 7.27 7.27 7.27 7.27 7.26 7.25 7.25 7.25 3.87 3.46 3.45 3.44 3.43 2.87 2.86 2.86 2.86 2.84 1.62
0.89 0.93 0.89 2.84 1.03 1.88 2.00 1.90
11.0 10.5 10.0 9.5 9.0 8.5 8.0 7.5 7.0 6.5 6.0 5.5 5.0 4.5 4.0 3.5 3.0 2.5 2.0 1.5 1.0 0.5 0.0 -0.5
f1 (ppm)

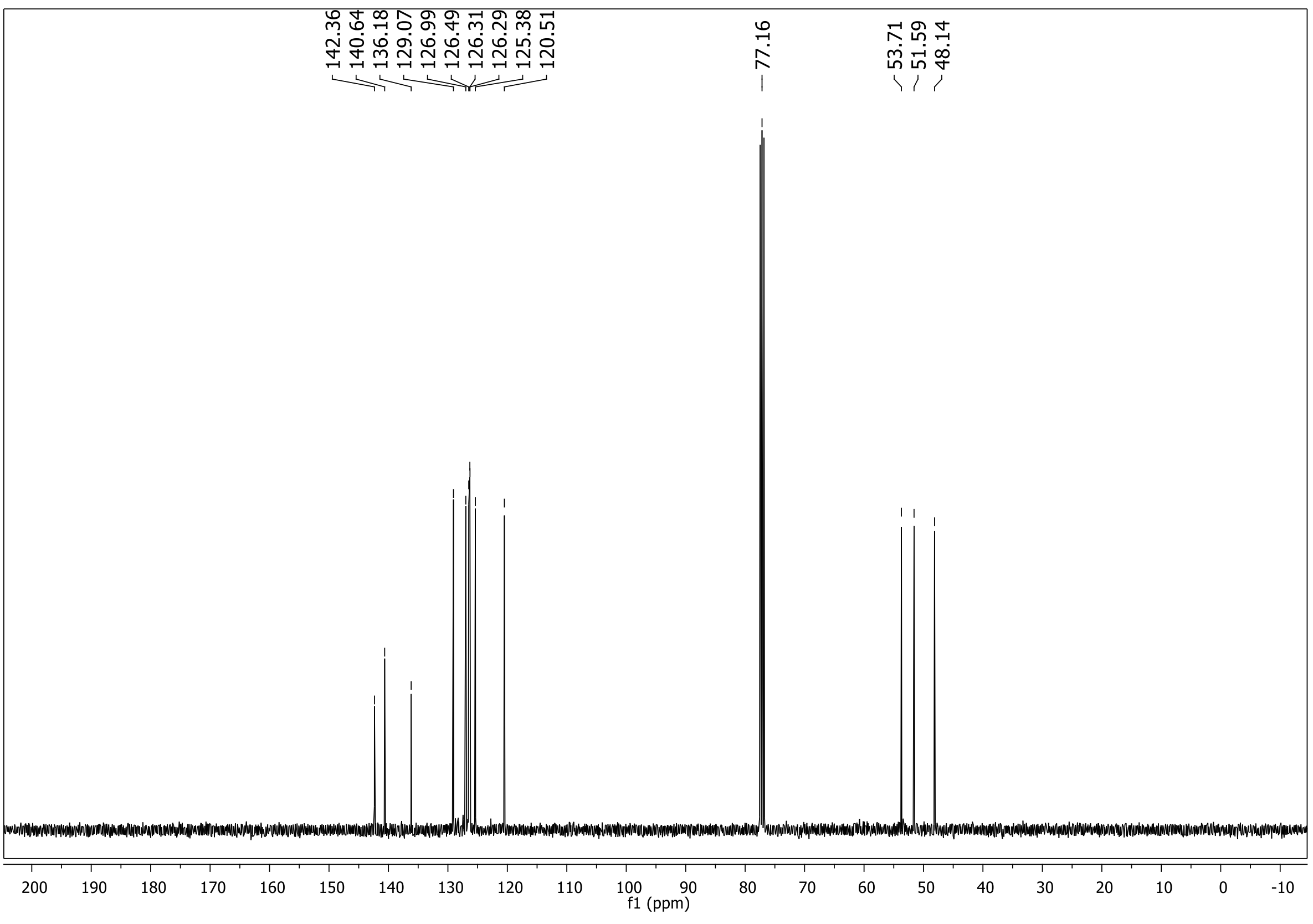
142.36 140.64 136.18 129.07 126.99 126.49 126.31 126.29 125.38 120.51
77.16
53.71 51.59 48.14
200 190 180 170 160 150 140 130 120 110 100 90 80 70 60 50 40 30 20 10 0 -10
f1 (ppm)

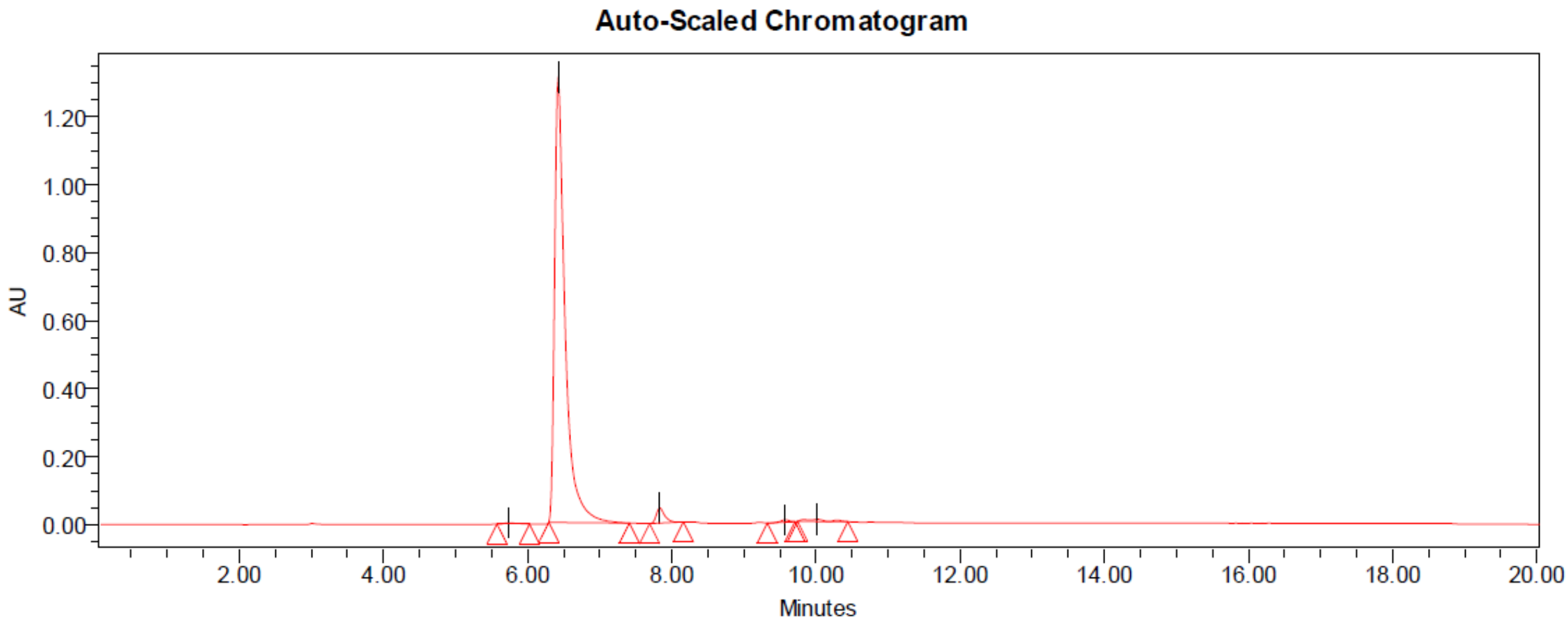

Processed Channel: PDA 254.0 nm

	Processed Channel	Retention Time (min)	Area	% Area	Height
1	PDA 254.0 nm	5.729	39106	0.28	3709
2	PDA 254.0 nm	6.427	13330498	95.78	1308525
3	PDA 254.0 nm	7.834	352412	2.53	44194

	Processed Channel	Retention Time (min)	Area	% Area	Height
4	PDA 254.0 nm	9.572	60633	0.44	7022
5	PDA 254.0 nm	10.015	134939	0.97	6635

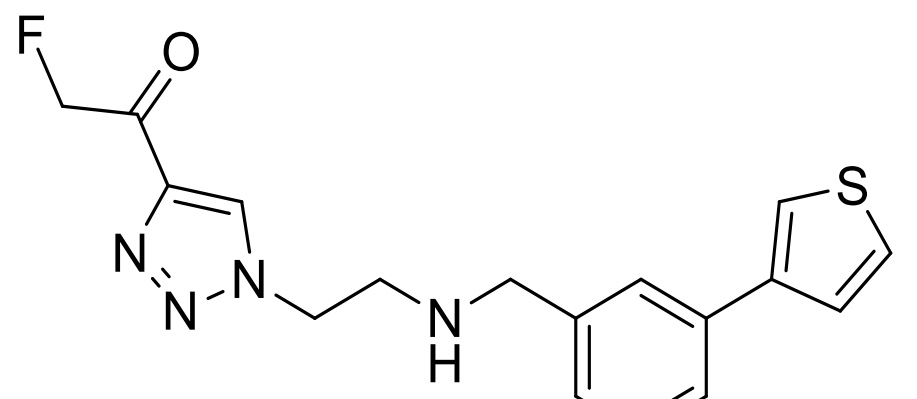

(97)

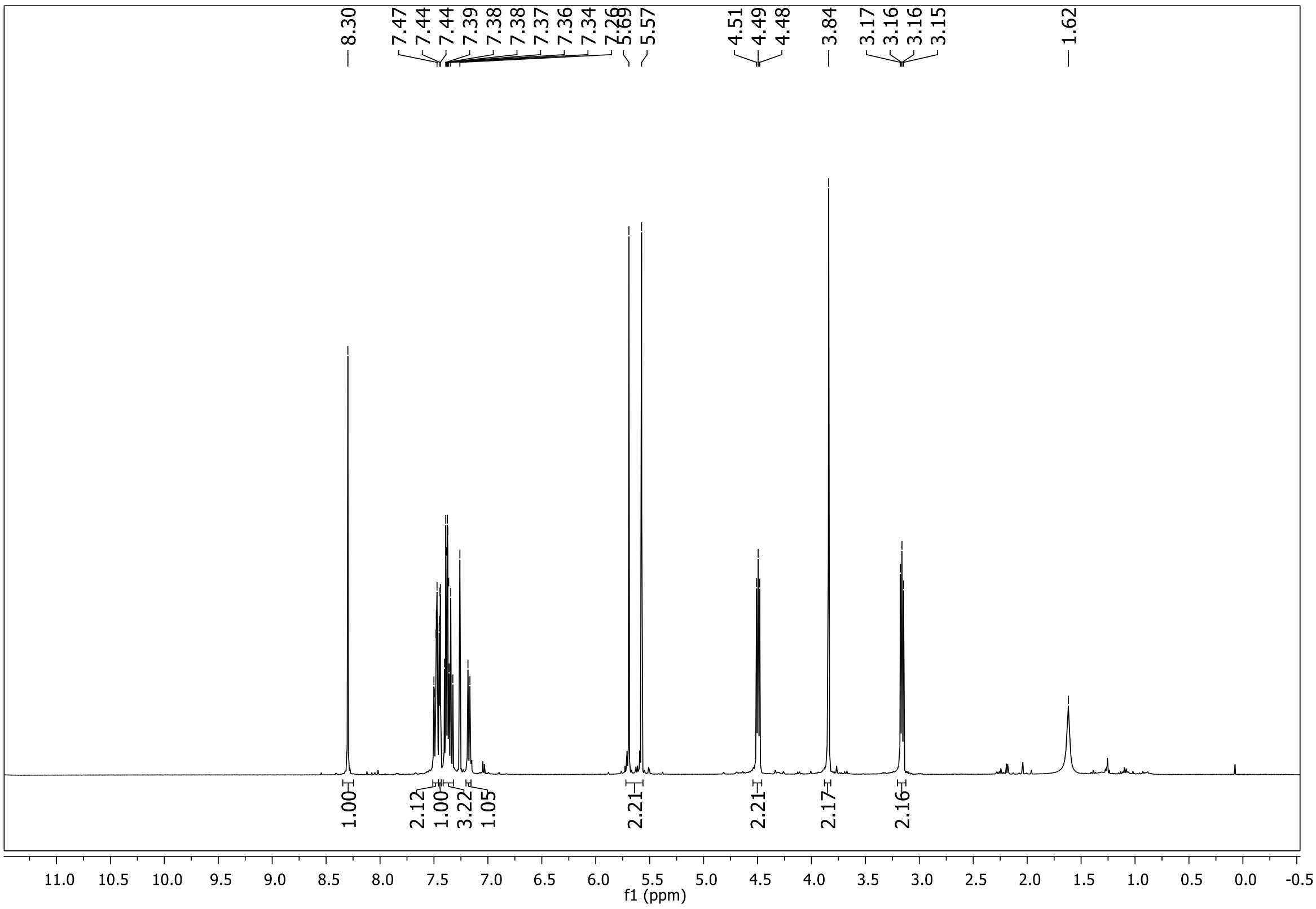

8.30
7.47
7.44
7.44
7.39
7.38
7.38
7.37
7.36
7.34
7.26
5.69
5.57
4.51
4.49
4.48
3.84
3.17
3.16
3.16
3.15
1.62
1.00
2.12
1.00
3.22
1.05
2.21
2.21
2.17
2.16
11.0
10.5
10.0
9.5
9.0
8.5
8.0
7.5
7.0
6.5
6.0
5.5
5.0
4.5
4.0
3.5
3.0
2.5
2.0
1.5
1.0
0.5
0.0
-0.5
f1 (ppm)

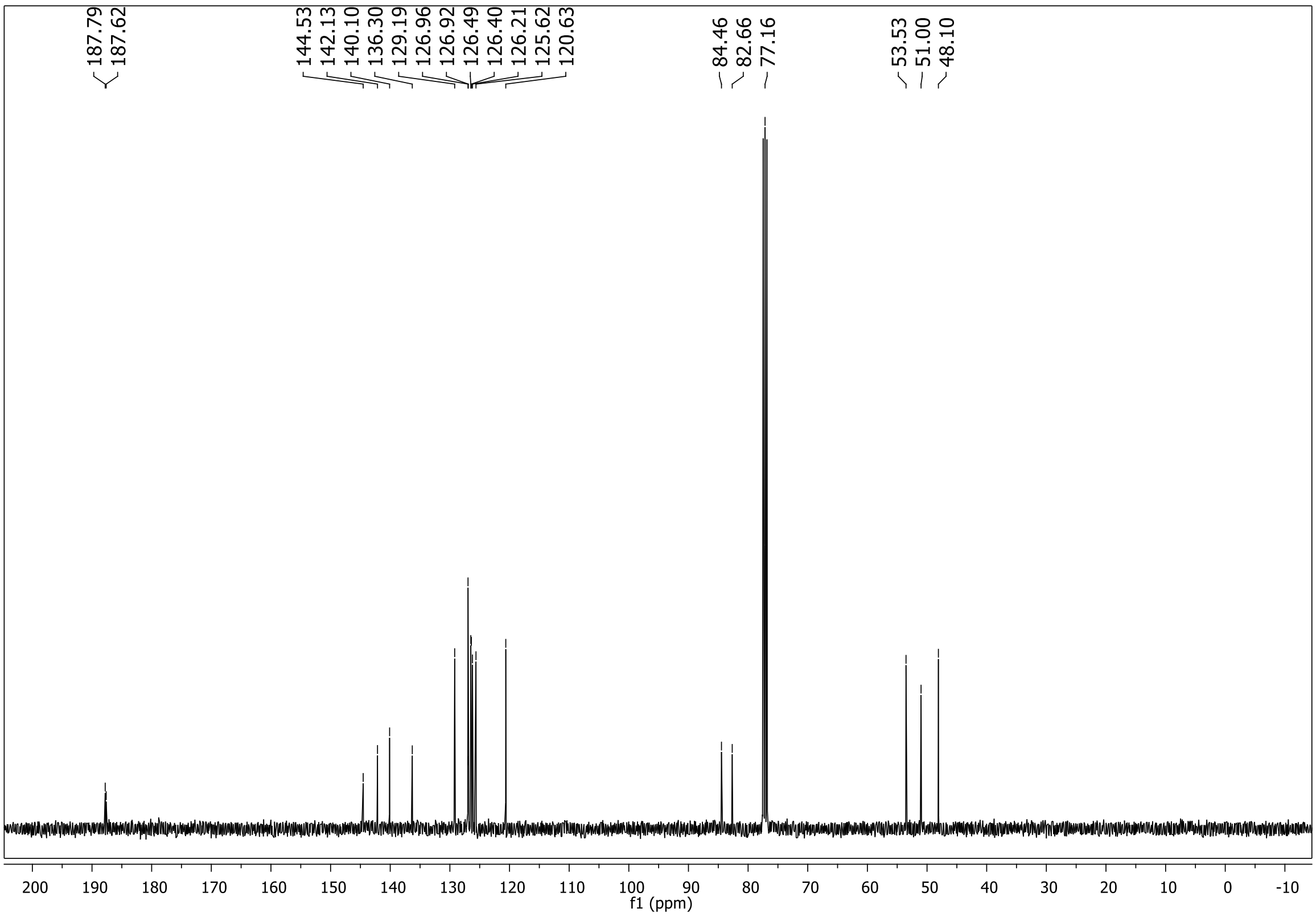

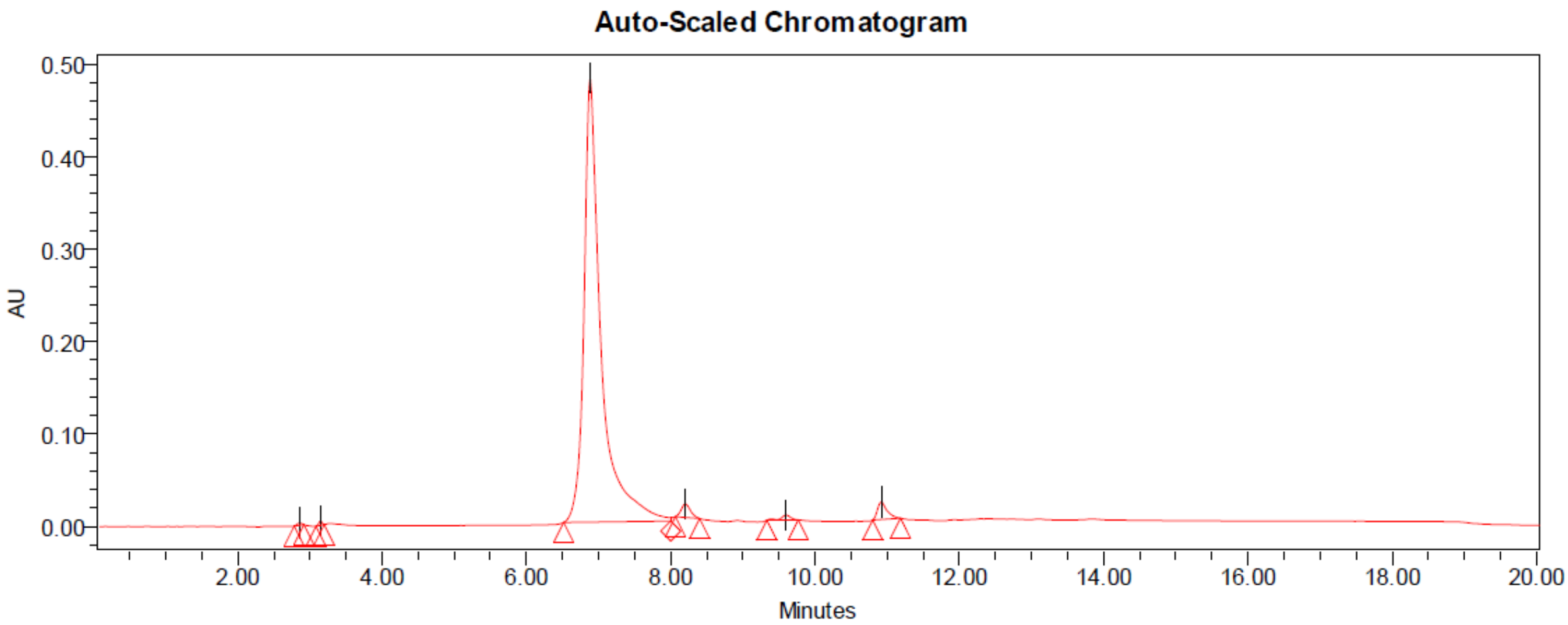

Processed Channel: PDA 254.0 nm

	Processed Channel	Retention Time (min)	Area	% Area	Height
1	PDA 254.0 nm	2.861	11024	0.13	2505
2	PDA 254.0 nm	3.150	15146	0.18	4697
3	PDA 254.0 nm	6.884	7910344	95.35	479830
4	PDA 254.0 nm	8.205	130406	1.57	14665
5	PDA 254.0 nm	9.599	52320	0.63	5398
6	PDA 254.0 nm	10.919	176825	2.13	18990

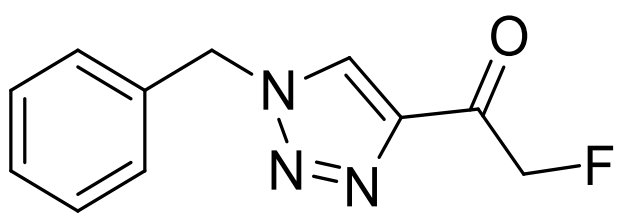
O
N
N
N
F

(98)

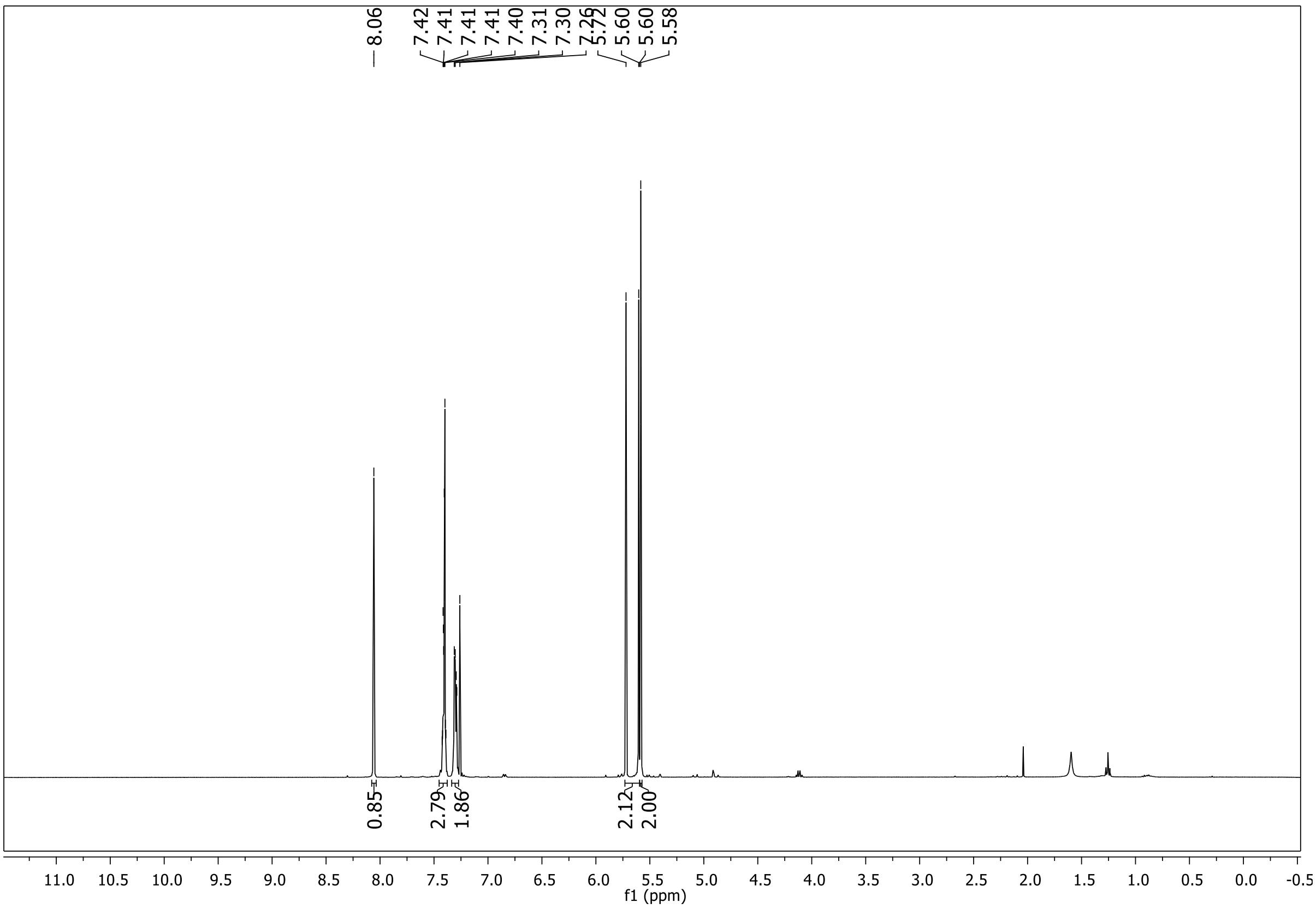
8.06
7.42
7.41
7.41
7.41
7.40
7.31
7.30
7.26
5.72
5.60
5.60
5.58
0.85
2.79
1.86
2.12
2.00
11.0 10.5 10.0 9.5 9.0 8.5 8.0 7.5 7.0 6.5 6.0 5.5 5.0 4.5 4.0 3.5 3.0 2.5 2.0 1.5 1.0 0.5 0.0 -0.5
f1 (ppm)

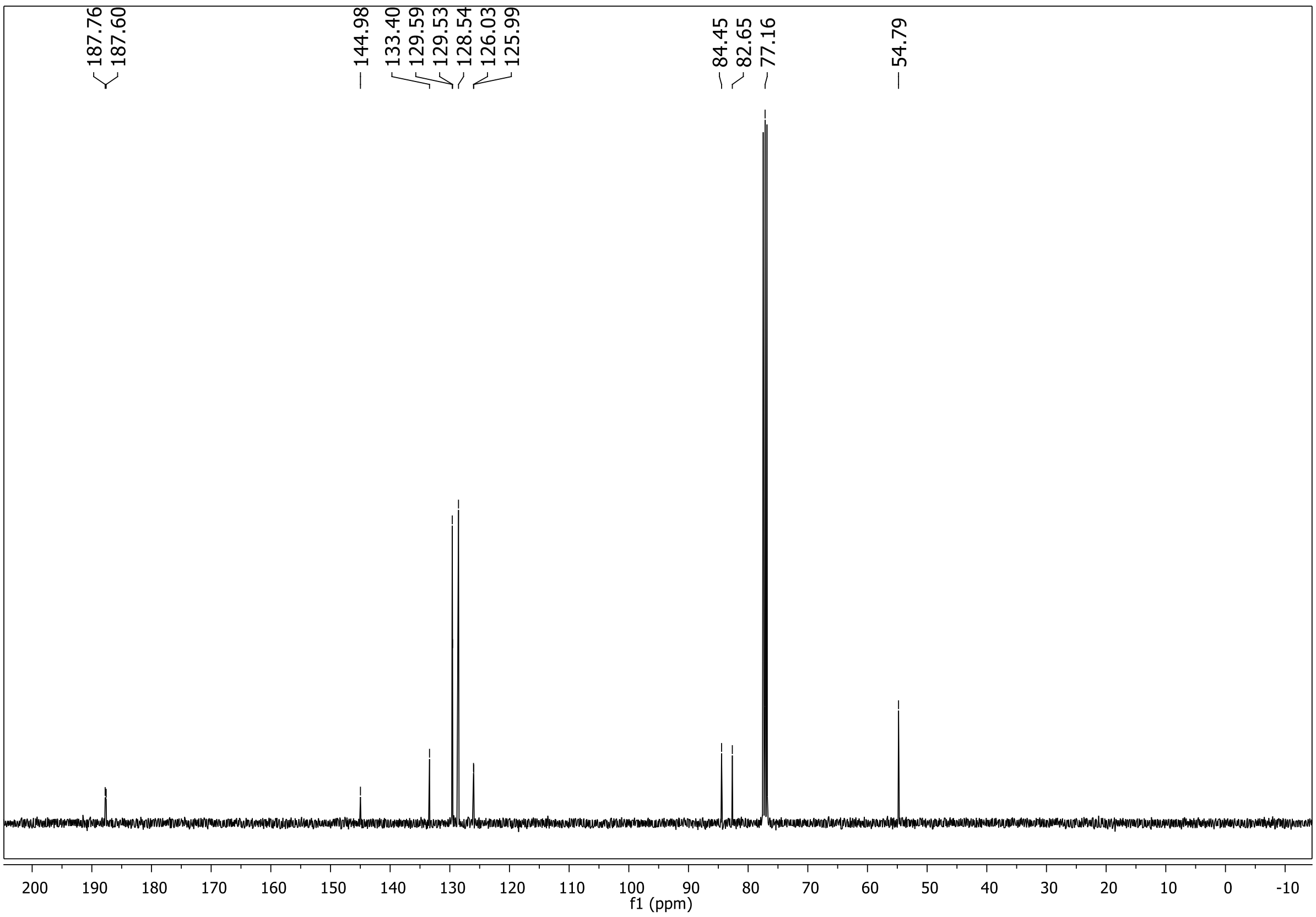
187.76
187.60
144.98
133.40
129.59
129.53
128.54
126.03
125.99
84.45
82.65
77.16
54.79
200 190 180 170 160 150 140 130 120 110 100 90 80 70 60 50 40 30 20 10 0 -10
f1 (ppm)

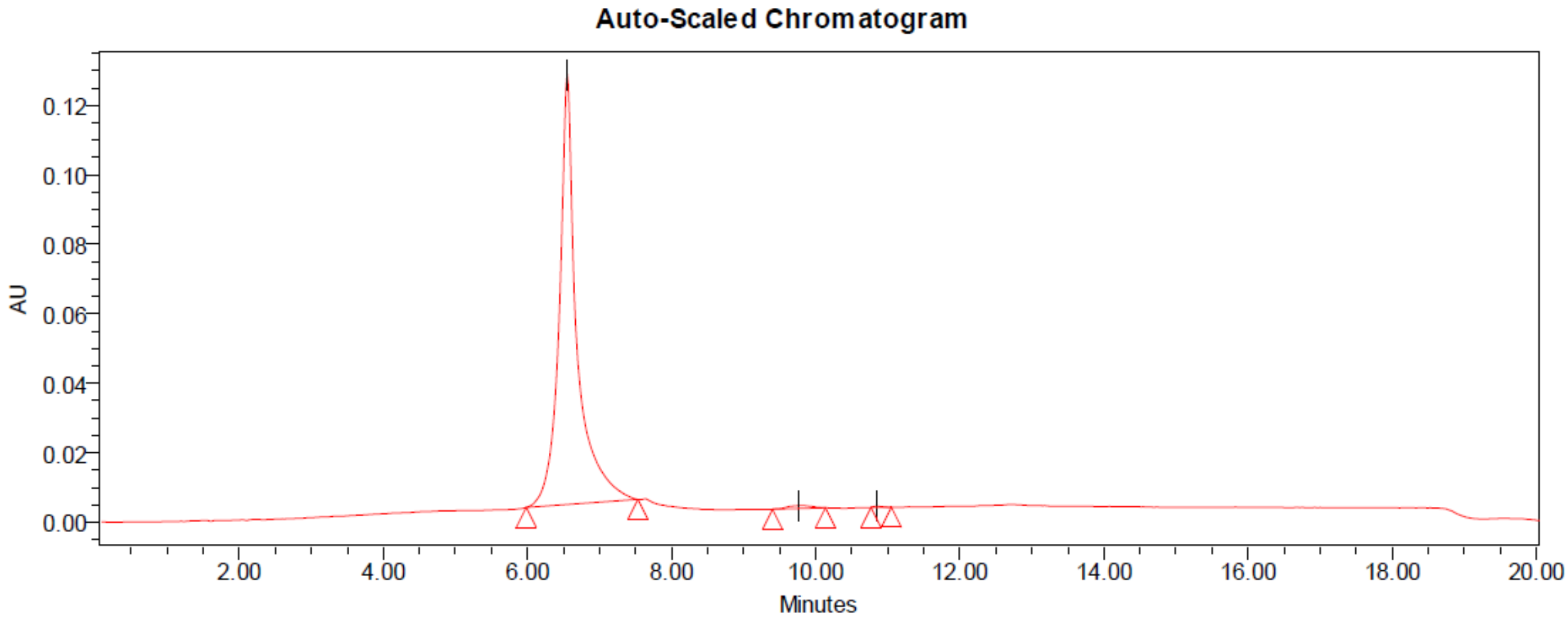

Processed Channel: PDA 254.0 nm

	Processed Channel	Retention Time (min)	Area	% Area	Height
1	PDA 254.0 nm	6.551	2124060	98.99	123849
2	PDA 254.0 nm	9.770	18984	0.88	806
3	PDA 254.0 nm	10.844	2743	0.13	325

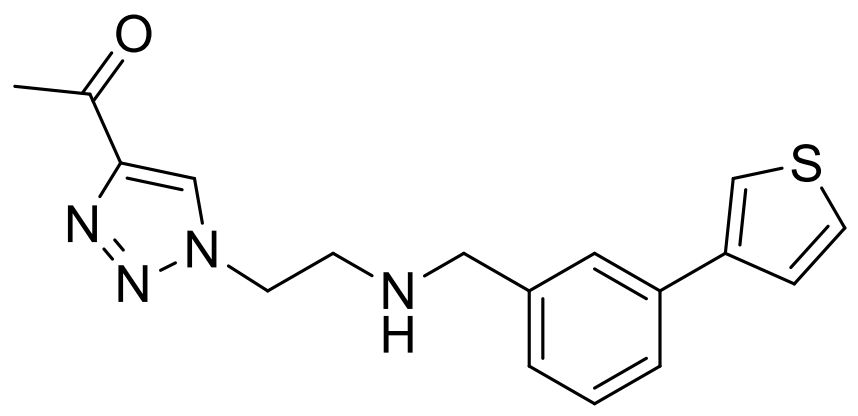
O
N
N
N
N
H
S

(**99**)

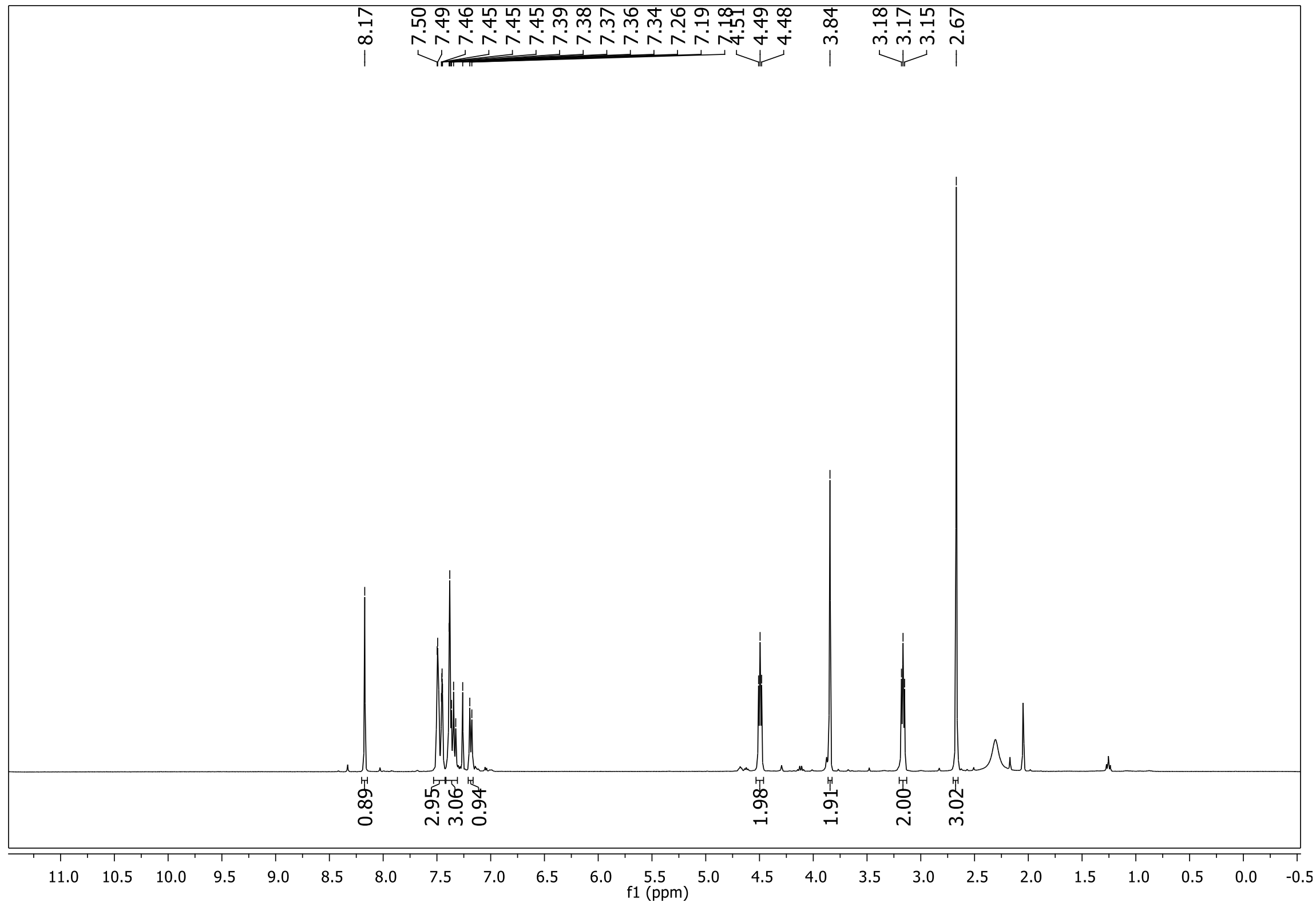
8.17
7.50
7.49
7.46
7.45
7.45
7.45
7.39
7.38
7.37
7.36
7.34
7.26
7.19
7.18
4.51
4.49
4.48
3.84
3.18
3.17
3.15
2.67
0.89
2.95
3.06
0.94
1.98
1.91
2.00
3.02
11.0 10.5 10.0 9.5 9.0 8.5 8.0 7.5 7.0 6.5 6.0 5.5 5.0 4.5 4.0 3.5 3.0 2.5 2.0 1.5 1.0 0.5 0.0 -0.5
f1 (ppm)

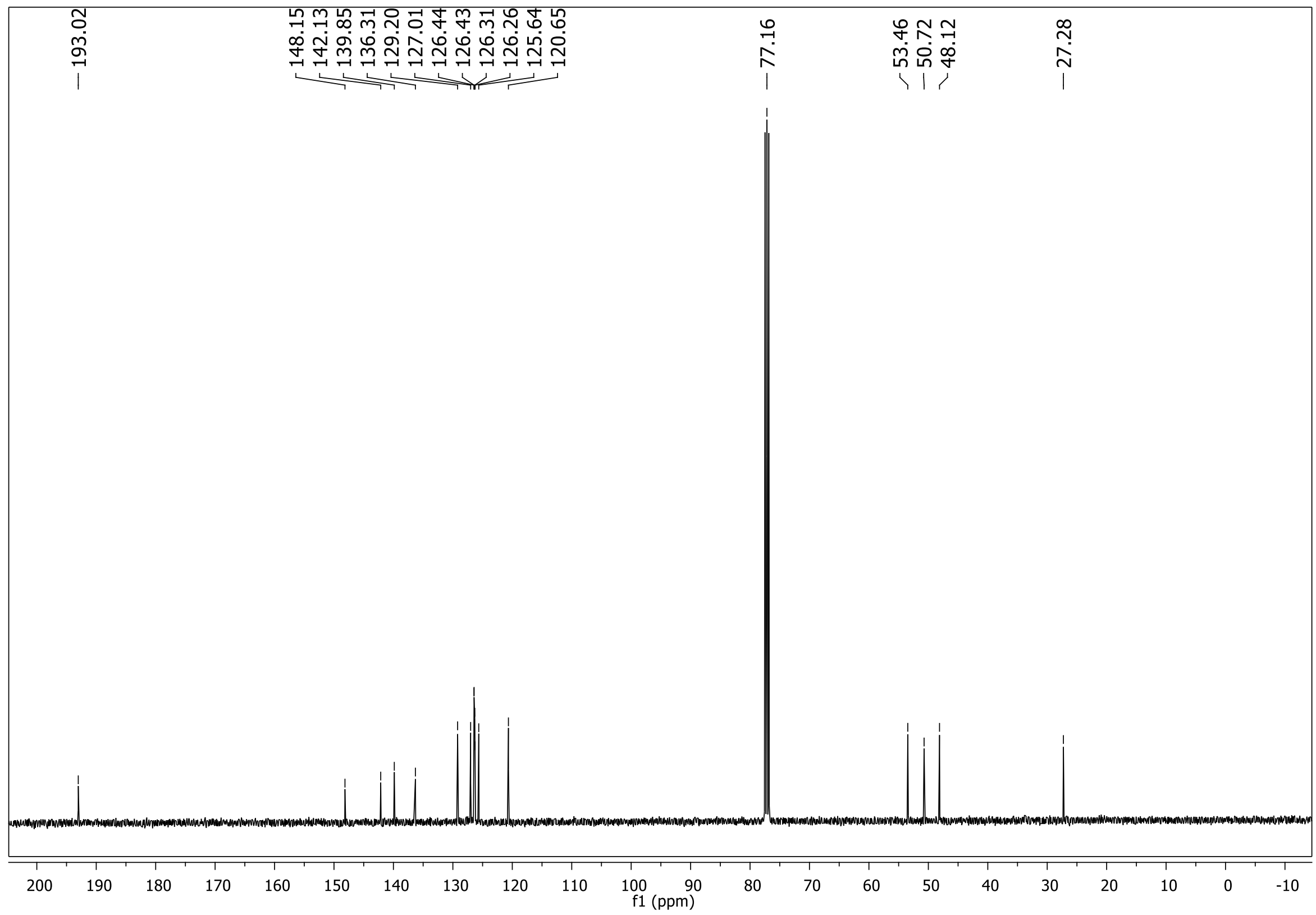
193.02
148.15
142.13
139.85
136.31
129.20
127.01
126.44
126.43
126.31
126.26
125.64
120.65
77.16
53.46
50.72
48.12
27.28
200 190 180 170 160 150 140 130 120 110 100 90 80 70 60 50 40 30 20 10 0 -10
f1 (ppm)

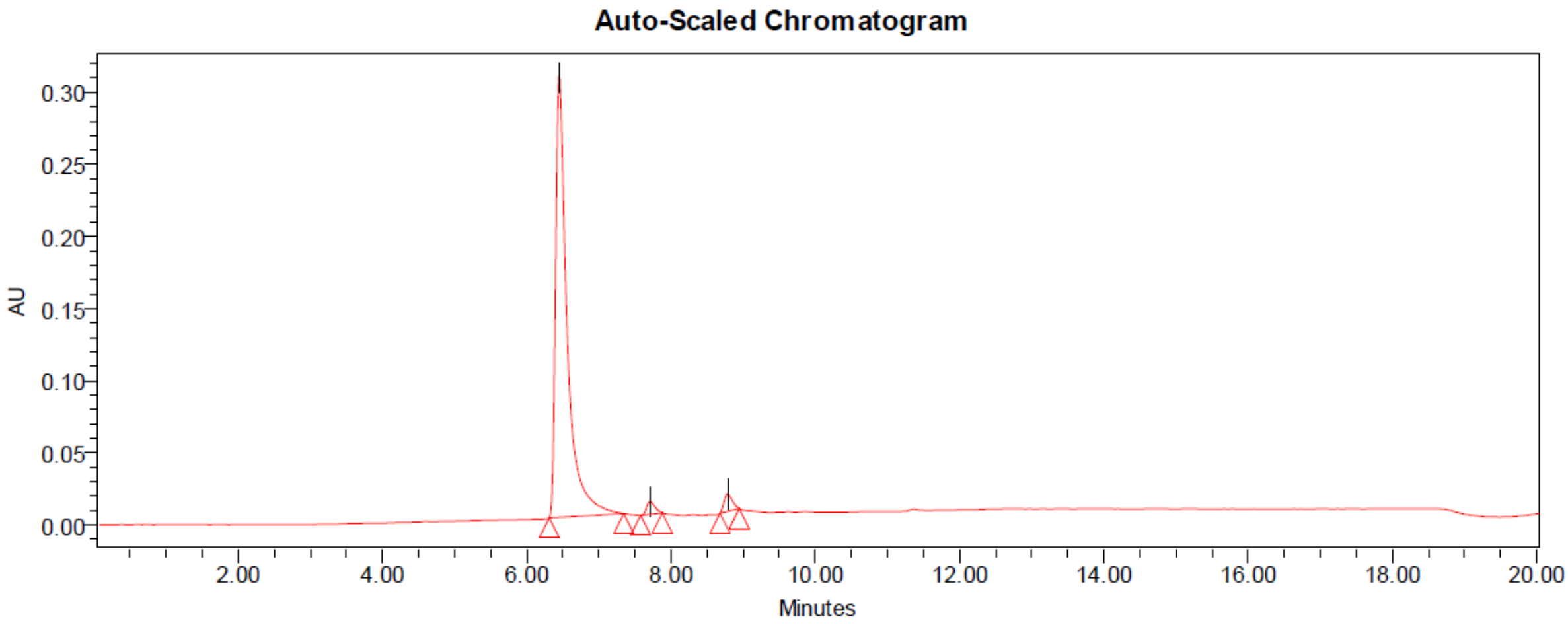

Processed Channel: PDA 254.0 nm

	Processed Channel	Retention Time (min)	Area	% Area	Height
1	PDA 254.0 nm	6.454	3416535	95.39	308910
2	PDA 254.0 nm	7.713	69259	1.93	8951
3	PDA 254.0 nm	8.787	95902	2.68	12161

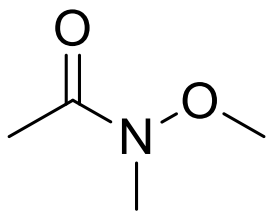
O
N
O

(**100**)

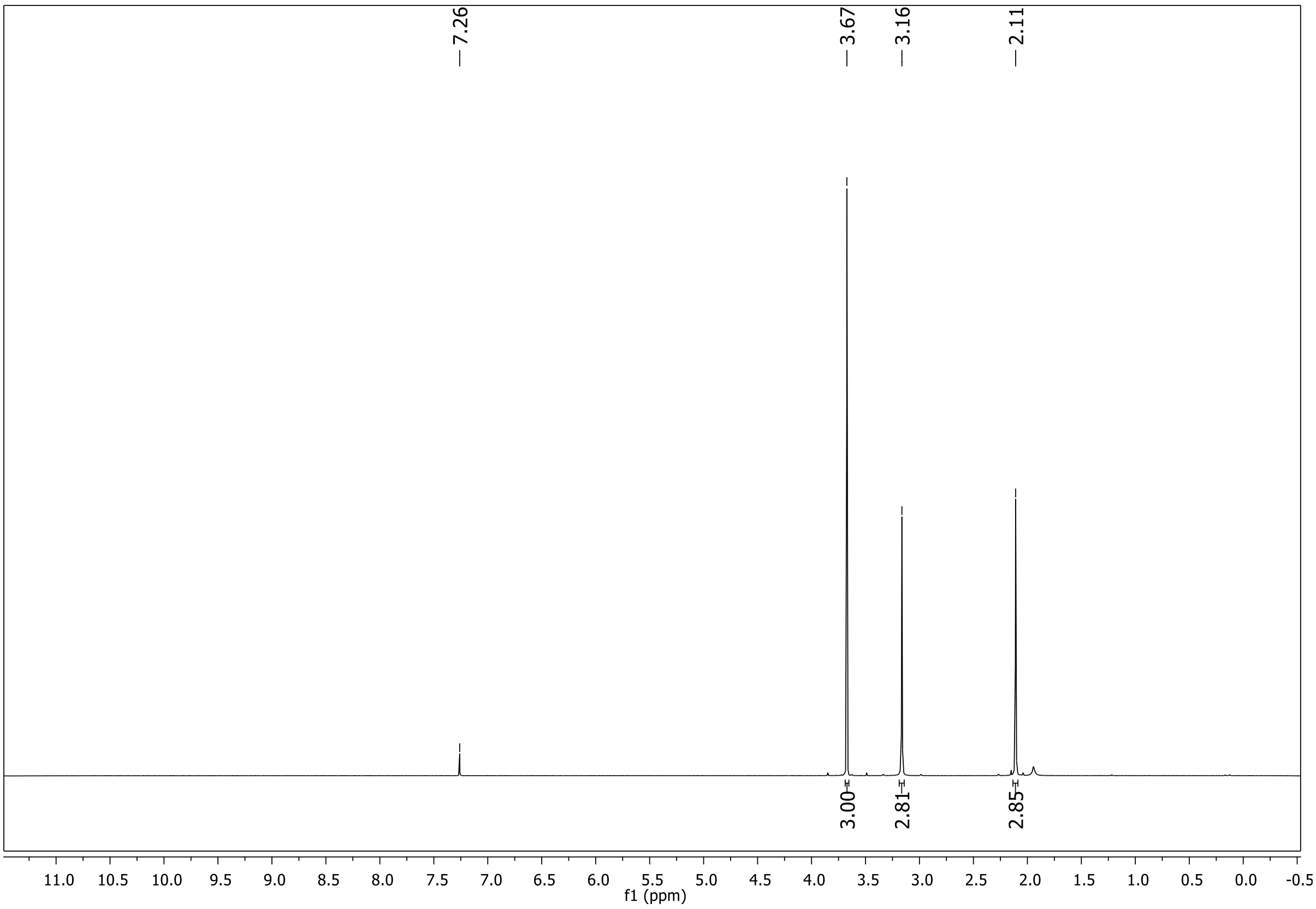
7.26
3.67
3.16
2.11
3.00
2.81
2.85
11.0 10.5 10.0 9.5 9.0 8.5 8.0 7.5 7.0 6.5 6.0 5.5 5.0 4.5 4.0 3.5 3.0 2.5 2.0 1.5 1.0 0.5 0.0 -0.5
f1 (ppm)

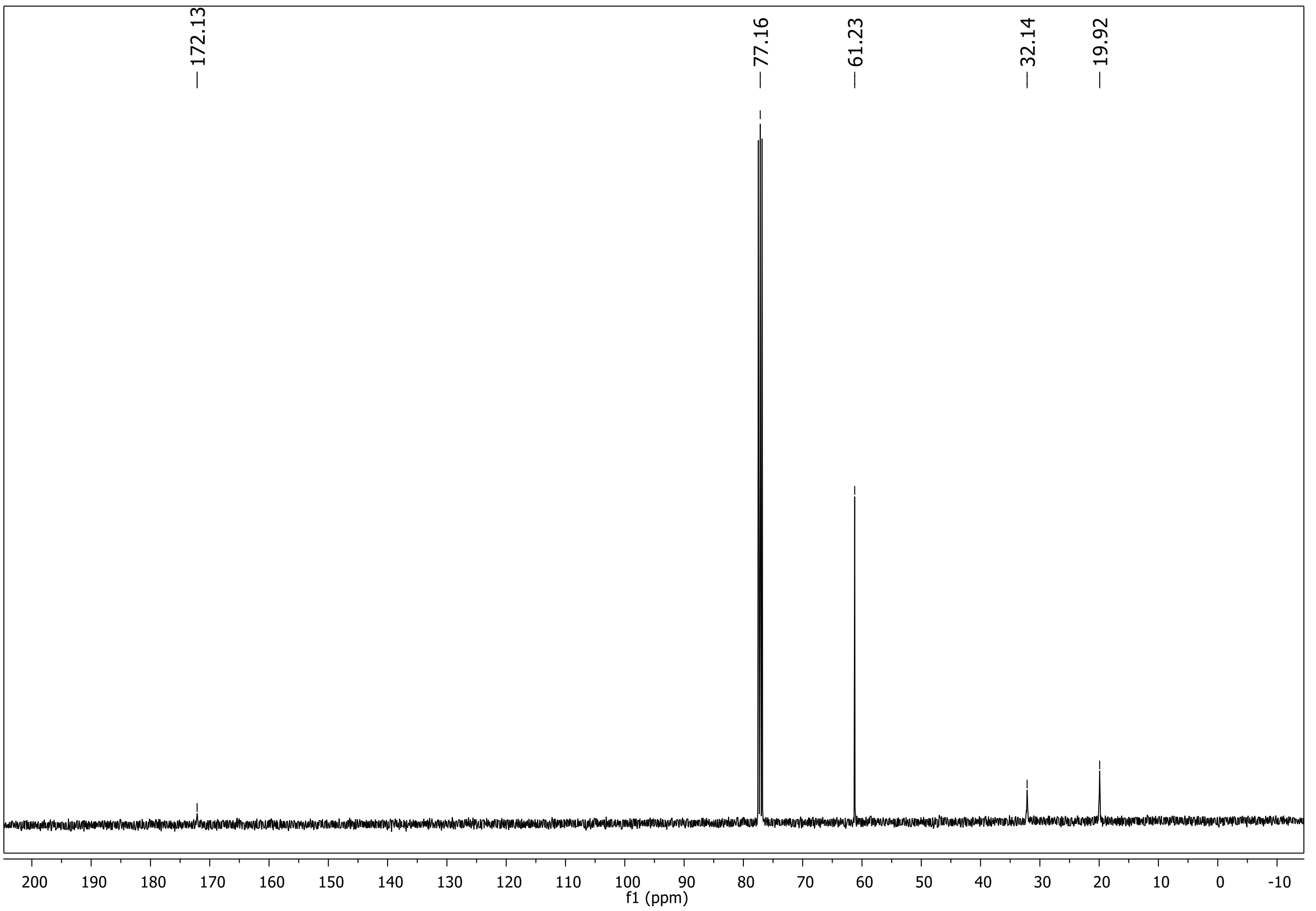
172.13
77.16
61.23
32.14
19.92
200 190 180 170 160 150 140 130 120 110 100 90 80 70 60 50 40 30 20 10 0 -10
f1 (ppm)

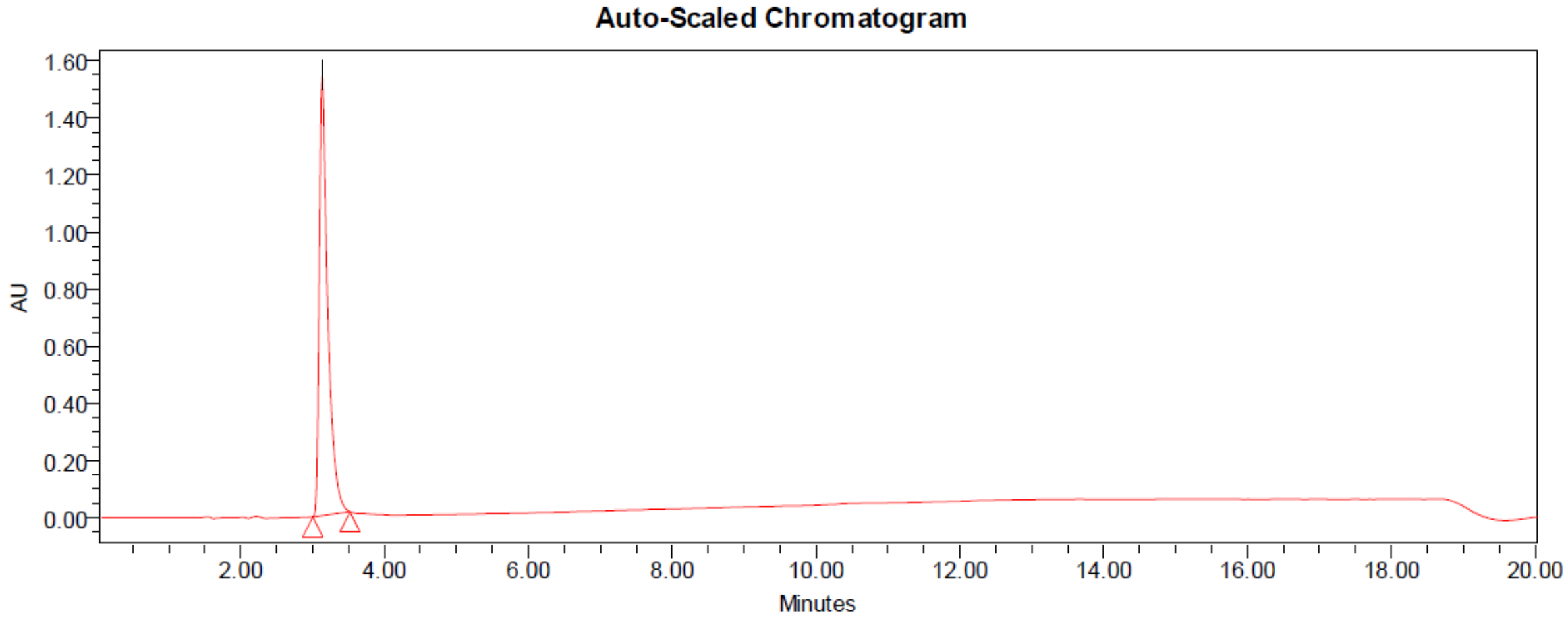

Processed Channel: PDA 214.0 nm

	Processed Channel	Retention Time (min)	Area	% Area	Height
1	PDA 214.0 nm	3.137	12881501	100.00	1542793

15. ^{15}N-^{1}H HSQC titration and residue K_D fits of compound 39

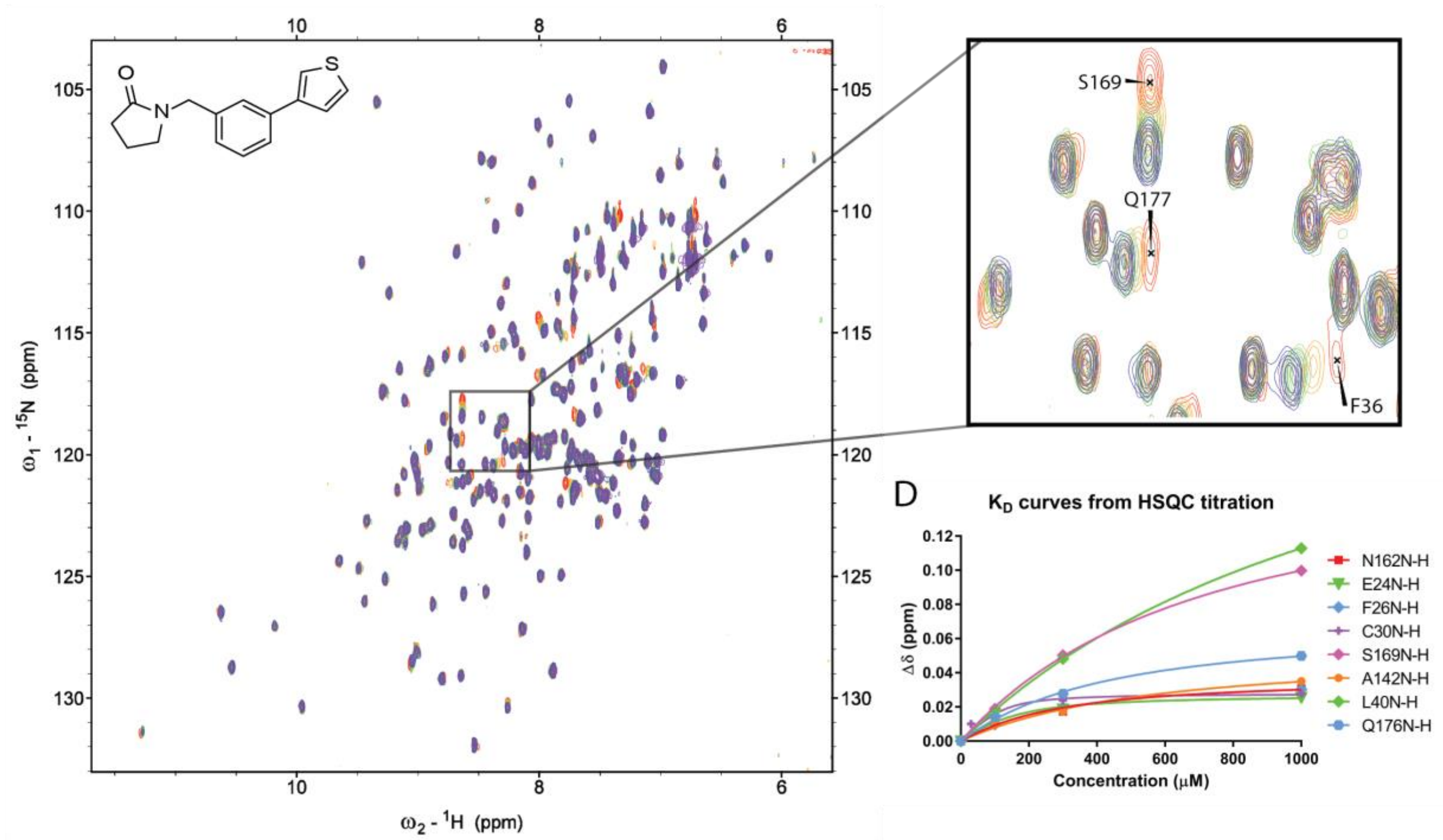

Supplement: Supplementary file 1 [file CMDC-19-e202300684-s001.pdf]
